# Supplementary material for: Pre-trained multimodal large language model enhances dermatological diagnosis using SkinGPT-4
Source: Nat Commun. 2024 Jul 5;15:5649. doi: 10.1038/s41467-024-50043-3 (PMC11226626; doi:10.1038/s41467-024-50043-3)
Supplement: Supplementary file 1 — Supplementary Information [file 41467_2024_50043_MOESM1_ESM.pdf]

Supplementary Information of Pre-trained Multimodal Large Language Model Enhances Dermatological Diagnosis using SkinGPT-4

Juexiao Zhou[1,2,3,†], Xiaonan He[4,†,*], Liyuan Sun[5,†], Jiannan Xu[5], Xiuying Chen[1,2], Yuetan Chu[1,2], Longxi Zhou[1,2], Xingyu Liao[1,2], Bin Zhang[1,2], Shawn Afvari[3,6,7], Xin Gao[1,2,*]

[1] Computer Science Program, Computer, Electrical and Mathematical Sciences and Engineering Division, King Abdullah University of Science and Technol- ogy (KAUST), Thuwal 23955-6900, Kingdom of Saudi Arabia
[2] Computational Bioscience Research Center, King Abdullah University of Science and Technology (KAUST), Thuwal 23955-6900, Kingdom of Saudi Arabia
[3] DermAssure, LLC, New York, NY, USA.
[4] Emergency Critical Care Center, Beijing AnZhen Hospital, Affiliated to Capital Medical University, Beijing 100029, China
[5] Department of Dermatology, Beijing AnZhen Hospital, Affiliated to Capital Medical University, Beijing 100029, China
[6] Department of Dermatology, Brigham and Women's Hospital, Harvard University, Boston, MA, USA.
[7] School of Medicine, New York Medical College, Valhalla, NY, USA.
[†] These authors contributed equally.
[*] Corresponding author. e-mail: xin.gao@kaust.edu.sa

Content Outline

FIGURE S1. DIAGNOSIS GENERATED BY SKINGPT-4, SKINGPT-4 (STEP 1 ONLY), SKINGPT-4 (STEP 2 ONLY), MINIGPT-4 AND DERMATOLOGISTS. THIS FIGURE SHOWS A CASE OF ECZEMA FINGERTIPS. 7

FIGURE S2. DIAGNOSIS GENERATED BY SKINGPT-4 ON SKIN DISEASE IMAGES UNDER VARYING ANGLES, LIGHTING, PIXELS, AND RESOLUTION CONDITIONS IN ACNE CASES OF DIFFERENT SEVERITY. ACNE CLASSIFICATION IS PROPOSED FOLLOWING THE CHINESE GUIDELINES FOR THE TREATMENT OF ACNE (REVISED 2019)....8

FIGURE S3. RECOMMENDED GUIDELINES FOR SUBMITTING QUALIFIED SKIN DISEASE IMAGES BY USERS WHEN USING SKINGPT-4. THE IMAGE SHOULD BE WELL-DEFINED, ALLOWING FOR CLEAR VISIBILITY OF THE SKIN DISEASE AREA, INCLUDING ITS COLOR, SHAPE, AND OTHER RELEVANT DETAILS VISIBLE TO THE NAKED EYE. THE SKIN DISEASE AREA IS SUGGESTED TO BE POSITIONED AT THE CENTER OF THE PICTURE. EXAMPLES OF QUALIFIED AND UNQUALIFIED IMAGES ARE PROVIDED. 9

CASE ACNE 1 10

CASE ACNE 2 13

CASE ACNE 3 15

CASE ACNE 4 17

CASE ACNE 5 20

CASE ACNE 6 23

CASE ACNE 7 26

CASE ACNE 8 29

CASE ACNE 9 32

CASE ACNE 10.... 34

CASE ROSACEA 1 36

CASE ROSACEA 2 39

CASE ROSACEA 3 42

CASE ROSACEA 4 45

CASE ROSACEA 5 48

CASE ROSACEA 6 51

CASE ROSACEA 7 54

CASE ROSACEA 8 57

CASE ROSACEA 9 59

CASE ROSACEA 10.... 62

CASE ACTINIC KERATOSIS 1.... 64

CASE ACTINIC KERATOSIS 2.... 67

CASE ACTINIC KERATOSIS 3.... 69

CASE ACTINIC KERATOSIS 4.... 72

CASE ACTINIC KERATOSIS 5.... 75

CASE BASAL CELL CARCINOMA 1.... 78

CASE BASAL CELL CARCINOMA 2 81
CASE BASAL CELL CARCINOMA 3 84
CASE BASAL CELL CARCINOMA 4 87
CASE BASAL CELL CARCINOMA 5 89
CASE ATOPIC DERMATITIS 1 92
CASE ATOPIC DERMATITIS 2 95
CASE ECZEMA 1 98
CASE ECZEMA 2 101
CASE ECZEMA 3 104
CASE ECZEMA 4 107
CASE ECZEMA 5 109
CASE ECZEMA 6 112
CASE ECZEMA 7 114
CASE ECZEMA 8 116
CASE BULLOUS DISEASE 1 119
CASE BULLOUS DISEASE 2 122
CASE BULLOUS DISEASE 3 124
CASE BULLOUS DISEASE 4 126
CASE BULLOUS DISEASE 5 129
CASE BULLOUS DISEASE 6 132
CASE BULLOUS DISEASE 7 135
CASE BULLOUS DISEASE 8 138
CASE BULLOUS DISEASE 9 141
CASE BULLOUS DISEASE 10 144
CASE CELLULITIS 1 146
CASE CELLULITIS 2 150
CASE CELLULITIS 3 152
CASE CELLULITIS 4 154
CASE CELLULITIS 5 157
CASE IMPETIGO 1 160
CASE IMPETIGO 2 163
CASE IMPETIGO 3 166
CASE SYCOSIS BARBAE 1 169
CASE BACTERIA INFECTION 171
CASE VITILIGO 1 173
CASE VITILIGO 2 177

CASE VITILIGO 3 181
CASE VITILIGO 4 184
CASE VITILIGO 5 187
CASE VITILIGO 6 191
CASE VITILIGO 7 195
CASE VITILIGO 8 198
CASE VITILIGO 9 200
CASE VITILIGO 10 202
CASE LUPUS 1 205
CASE LUPUS 2 208
CASE LUPUS 3 211
CASE LUPUS 4 214
CASE LUPUS 5 217
CASE LUPUS 6 220
CASE LUPUS 7 223
CASE LUPUS 8 226
CASE LUPUS 9 228
CASE LUPUS 10 231
CASE SEBORRHEIC KERATOSES 1 233
CASE SEBORRHEIC KERATOSES 2 235
CASE SEBORRHEIC KERATOSES 3 238
CASE SEBORRHEIC KERATOSES 4 241
CASE SEBORRHEIC KERATOSES 5 244
CASE SEBORRHEIC KERATOSES 6 247
CASE SEBORRHEIC KERATOSES 7 250
CASE SEBORRHEIC KERATOSES 8 252
CASE SEBORRHEIC KERATOSES 9 254
CASE SEBORRHEIC KERATOSES 10 257
CASE MELANOMA 1 260
CASE MELANOMA 2 262
CASE MELANOMA 3 265
CASE MELANOMA 4 267
CASE MELANOMA 5 269
CASE MELANOMA 6 271
CASE MELANOMA 7 274
CASE MELANOMA 8 276

CASE MELANOMA 9 279
CASE MELANOMA 10 281
CASE FUNGAL INFECTION-TINEA 1 283
CASE FUNGAL INFECTION-TINEA 2 286
CASE FUNGAL INFECTION-TINEA 3 289
CASE FUNGAL INFECTION-TINEA 4 292
CASE FUNGAL INFECTION-TINEA 5 294
CASE FUNGAL INFECTION-NAIL FUNGAL INFECTION 1 297
CASE FUNGAL INFECTION-NAIL FUNGAL INFECTION 2 300
CASE FUNGAL INFECTION-FUNGAL INFECTION 3 303
CASE FUNGAL INFECTION- NAIL FUNGAL INFECTION 4 306
CASE FUNGAL INFECTION-NAIL FUNGAL INFECTION 5 309
CASE PSORIASIS 1 312
CASE PSORIASIS 2 315
CASE PSORIASIS 3 318
CASE PSORIASIS 4 321
CASE PSORIASIS 5 324
CASE PSORIASIS 6 327
CASE PSORIASIS 7 330
CASE PSORIASIS 8 333
CASE PSORIASIS 9 336
CASE PSORIASIS 10 339
CASE INFESTATIONS AND BITES 1 342
CASE INFESTATIONS AND BITES 2 345
CASE INFESTATIONS AND BITES 3 348
CASE INFESTATIONS AND BITES 4 351
CASE INFESTATIONS AND BITES 5 354
CASE INFESTATIONS AND BITES 6 357
CASE INFESTATIONS AND BITES 7 360
CASE INFESTATIONS AND BITES 8 363
CASE INFESTATIONS AND BITES 9 366
CASE INFESTATIONS AND BITES 10 369
CASE HIVES 1 372
CASE HIVES 2 374
CASE HIVES 3 377
CASE HIVES 4 380

CASE HIVES 5 ... 383
CASE HIVES 6 ... 386
CASE HIVES 7 ... 389
CASE HIVES 8 ... 392
CASE HIVES 9 ... 395
CASE HIVES 10 ... 398
CASE VASCULAR TUMORS 1 ... 401
CASE VASCULAR TUMORS 2 ... 405
CASE VASCULAR TUMORS 3 ... 409
CASE VASCULAR TUMORS 4 ... 413
CASE VASCULAR TUMORS 5 ... 417
CASE VASCULAR TUMORS 6 ... 421
CASE VASCULAR TUMORS 7 ... 425
CASE VASCULAR TUMORS 8 ... 429
CASE VASCULAR TUMORS 9 ... 433
CASE VASCULAR TUMORS 10 ... 437
CASE HERPES 1 ... 439
CASE HERPES 2 ... 441
CASE HERPES 3 ... 444
CASE HERPES 4 ... 446
CASE HERPES 5 ... 449
CASE HERPES 6 ... 452
CASE HERPES 7 ... 455
CASE HERPES 8 ... 458
CASE HERPES 9 ... 460
CASE HERPES 10 ... 463

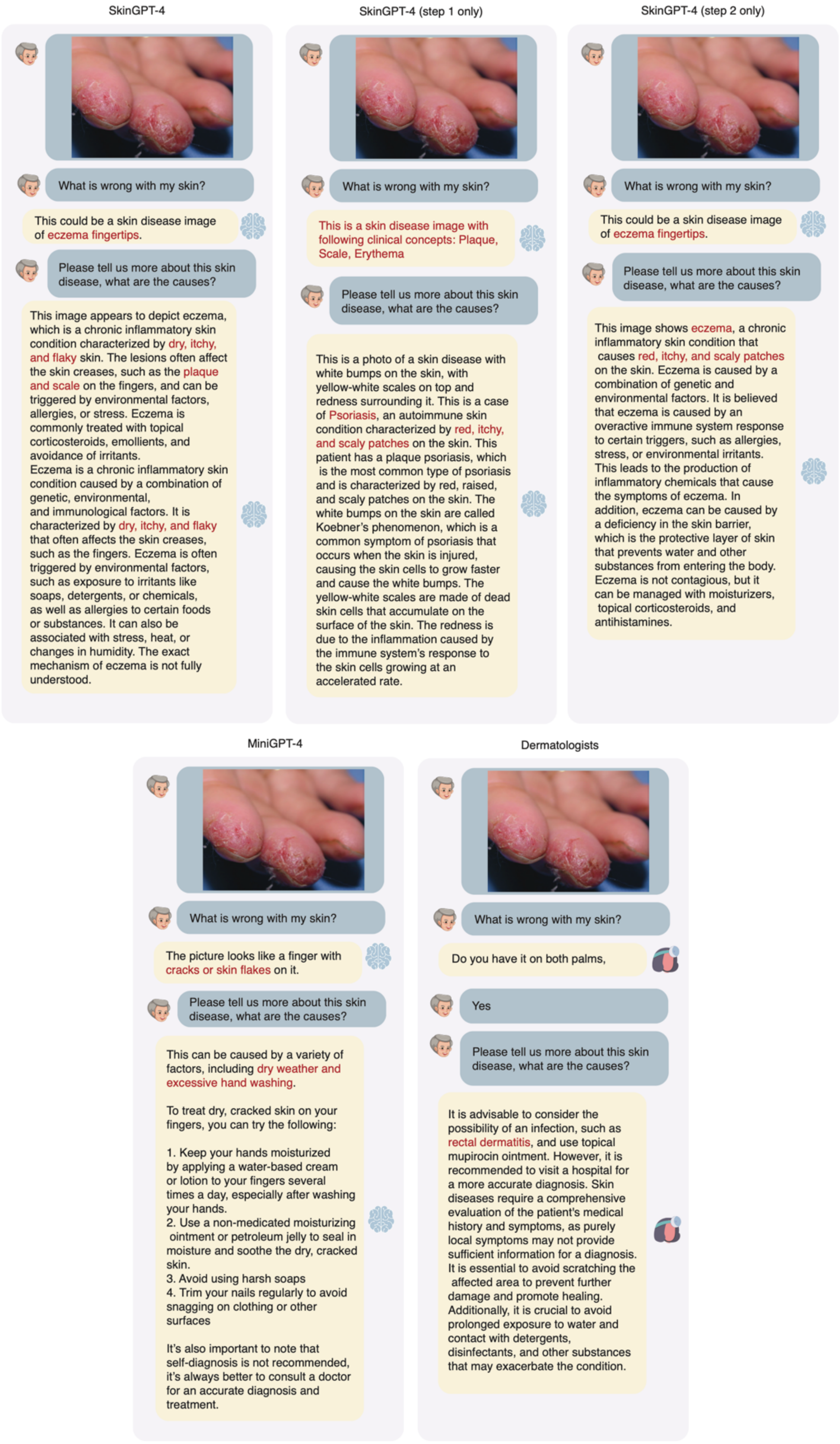

Figure S1. Diagnosis generated by SkinGPT-4, SkinGPT-4 (step 1 only), SkinGPT-4 (step 2 only), MiniGPT-4 and Dermatologists. This figure shows a case of eczema fingertips.

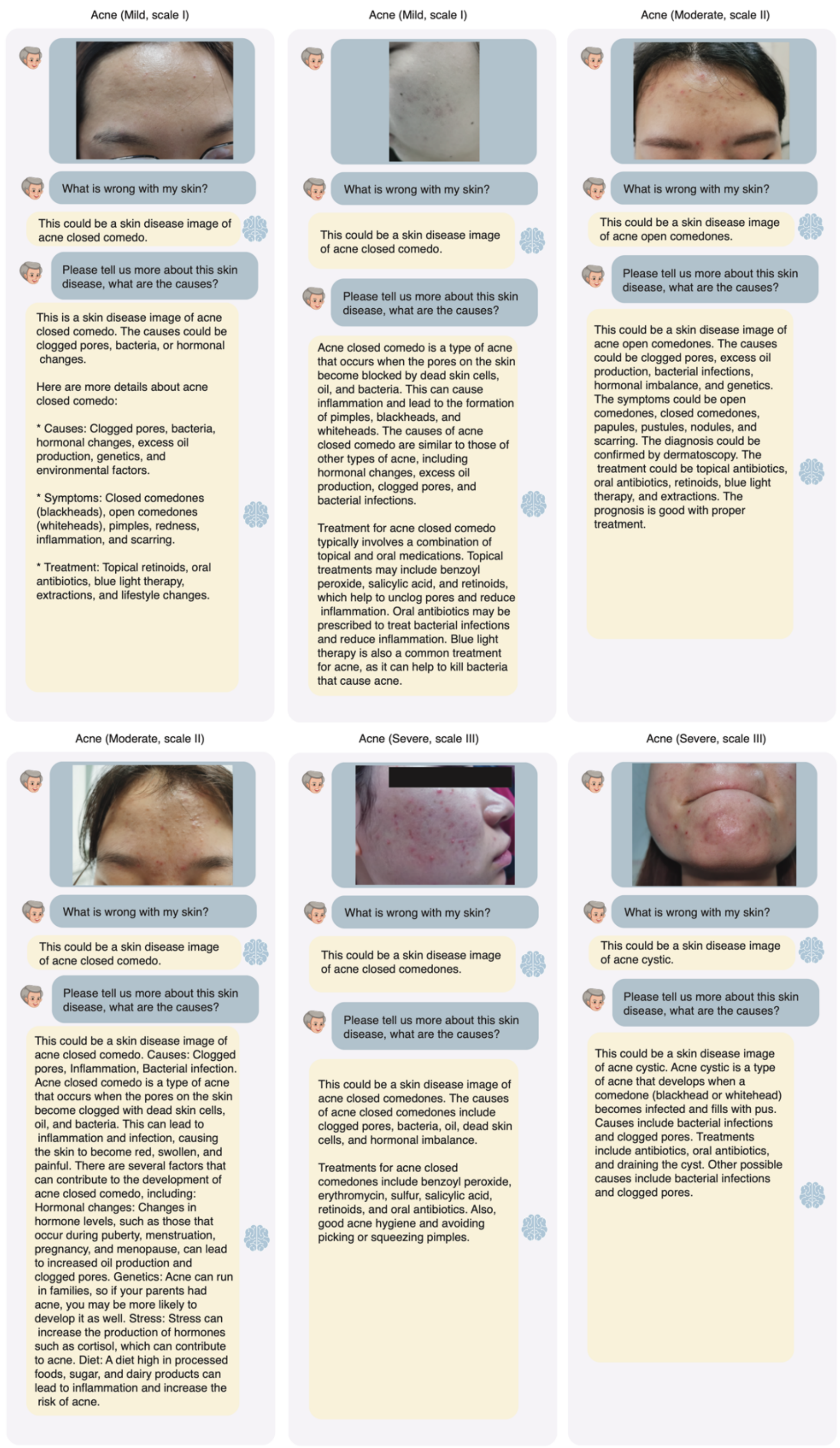

Figure S2. Diagnosis generated by SkinGPT-4 on skin disease images under varying angles, lighting, pixels, and resolution conditions in Acne cases of different severity. Acne classification is proposed following the Chinese guidelines for the treatment of acne (Revised 2019).

Qualified:

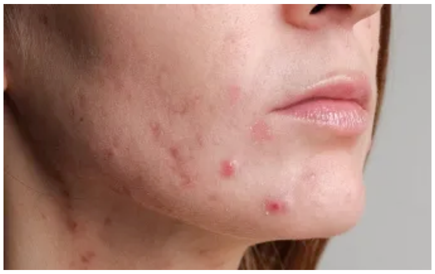

Unqualified (Reason: lesion area is too small and not centered)

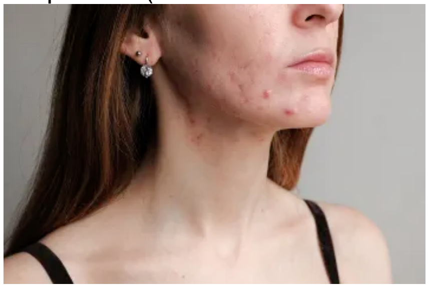

Unqualified (Reason: low light):

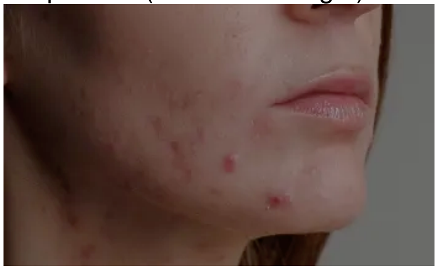

Unqualified (Reason: blurry picture):

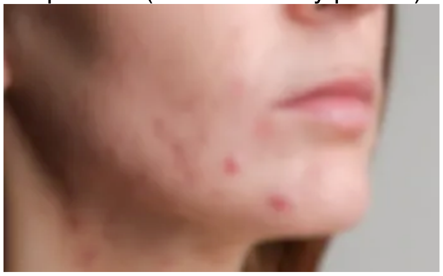

Figure S3. Recommended guidelines for submitting qualified skin disease images by users when using SkinGPT-4. The image should be well-defined, allowing for clear visibility of the skin disease area, including its color, shape, and other relevant details visible to the naked eye. The skin disease area is suggested to be positioned at the center of the picture. Examples of qualified and unqualified images are provided.

Case Acne 1

Image ID: acne 1	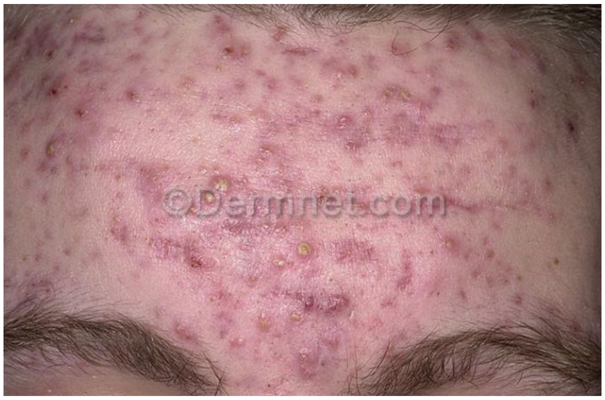
**SkinGPT-4**	**Dermatology Physician**
Q1. Could you describe the skin disease in this image for me?<br><br>A1. This could be a skin disease image of acne cystic.<br><br>Q2. You should write a paragraph to list more features you observed in the image.<br><br>A2. This image appears to show a cystic acne lesion on the face of an individual. The lesion is red, inflamed, and pus filled, with a central white head. It appears on the face, which is a common site for acne. The lesion is likely to cause scarring and discoloration, which are common characteristics of cystic acne.<br><br>Q3. With previous information, please write a paragraph to explain in detail the cause of this skin disease.<br><br>A3. This image appears to show a cystic acne lesion, which is caused by a blockage of the skin's sebaceous glands. Sebum, an oily substance produced by the glands, builds up and creates an environment that encourages the growth of bacteria. The bacteria infect the blocked glands and cause inflammation, leading to the formation of a cyst. The cyst then ruptures, causing the white head and pus that we see in the image. Cystic acne is a type of acne that commonly occurs on the face, chest, and back, and is characterized by painful, pus filled lumps that can cause scarring and discoloration. The lesion in the image is likely to be painful and tender to the	Q1. Could you describe the skin disease in this image for me?<br><br>A1. Acne<br><br>Q2. You should write a paragraph to list more features you observed in the image.<br><br>A2. We can see scattered follicular erythema, red papules and pustules with some crusting on the forehead.<br><br>Q3. With previous information, please write a paragraph to explain in detail the cause of this skin disease.<br><br>A3. Acne is a common skin condition which has an multifactorial disorder of the pilosebaceous unit. The development of acne involves a complex interaction of multiple factors, both internal and external to the pilosebaceous apparatus.<br>The pathogenesis of acne is still not fully understood. Genetics, androgen-induced sebum secretion, keratinization of follicular sebaceous ducts. The genetic predisposition, androgen-induced sebum production, keratinization of follicular sebaceous ducts, Propionibacterium acnes colonization, inflammation and immune response may all be involved.<br><br>Q4. How should I treat it? What medicine should I use?<br><br>A4. The patient in this case required treatment with oral antibiotics. If this is not effective, oral

touch, and may have a purulent discharge. The individual may also experience redness and inflammation in the surrounding skin. Cystic acne is often treated with topical retinoids, antibiotics, and isotretinoin, and scarring may be prevented or reduced by using a combination of these treatments.<br><br>Q4. How should I treat it? What medicine should I use?<br><br>A4. The treatment for cystic acne typically involves a combination of topical and oral medications.<br>Topical treatments:<br>* Benzoyl peroxide: Kills bacteria and helps unplug sebum from the pores.<br>* Salicylic acid: Helps to unplug follicles and reduce inflammation.<br>* Azelaic acid: Kills bacteria, reduces inflammation and helps to improve skin texture.<br>* Retinoids: Help to unplug follicles and reduce inflammation.<br>Oral antibiotics:<br>* Erythromycin, doxycycline or tetracycline: Help to reduce the number of bacteria that cause acne.<br>* Isotretinoin: For severe nodular acne that does not respond to other treatments.<br>It is important to consult a dermatologist for a proper diagnosis and treatment plan. They may also prescribe a combination of topical and oral medications, and may recommend a skin care routine to help prevent future breakouts.	isotretinoin may be given. Topical anti-antibiotics creams/gel, retinoic acid and benzoyl peroxide gel are also needed.
Evaluation of Physician	
Evaluation Item	Score (1-5, the higher the better)
SkinGPT-4's diagnosis is correct or relevant.	4
SkinGPT-4's description is informative.	4
SkinGPT-4's suggestions are useful.	4
SkinGPT-4 can help doctors with diagnosis.	4
SkinGPT-4 can help patients to understand their disease better.	4

If SkinGPT-4 can be deployed locally, it protects patients’ privacy.	5
Willingness to use SkinGPT-4	5

Case Acne 2

Image ID: acne 2	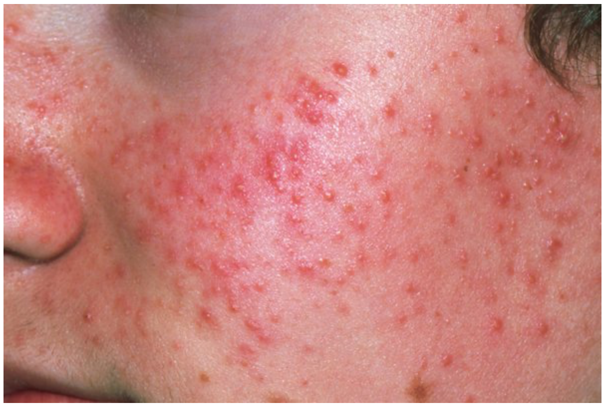
**SkinGPT-4**	**Dermatology Physician**
Q1. Could you describe the skin disease in this image for me?<br><br>A1. acne and rosacea and allergic reactions<br><br>Q2. You should write a paragraph to list more features you observed in the image.<br><br>A2. In this image, there are several features that can be observed. First, there is a red and pus - filled bump on the face. Second, there is a rash on the forehead and cheeks. Third, there are whiteheads and blackheads on the nose and chin. Fourth, there is a scaly patch on the forehead. Fifth, there are red and inflamed patches on the skin. Sixth, there is a white and flaky substance on the skin. Seventh, there is a discoloration of the skin on the face and neck. Eighth, there are pus - filled blisters on the arms and legs. Ninth, there is a crusting of the skin on the lips and eyelids. Tenth, there is a swelling of the lymph nodes in the neck.<br><br>Q3. With previous information, please write a paragraph to explain in detail the cause of this skin disease.<br><br>A3. The cause of this skin disease is not known. It is likely that it is caused by a combination of genetic and environmental factors, as well as lifestyle choices. It may also be caused by a viral or bacterial infection. The specific triggers that cause this condition are not well understood and may vary from person to person. In some cases, it may be triggered by an underlying medical	Q1. Could you describe the skin disease in this image for me?<br><br>A1. Inflammatory acne vulgaris and rosacea (This image shows only a portion of the lesion and does not provide enough information to diagnose Rosacea)<br><br>Q2. You should write a paragraph to list more features you observed in the image.<br><br>A2. Inflammatory papules on the base of erythema of the cheek<br><br>Q3. With previous information, please write a paragraph to explain in detail the cause of this skin disease.<br><br>A3. Acne is an inflammatory disease of the sebaceous units of the hair follicles.<br>The pathogenesis of acne is still not fully understood. Genetics, androgen-induced sebum secretion, keratinization of follicular sebaceous ducts. The genetic predisposition, androgen-induced sebum production, keratinization of follicular sebaceous ducts, Propionibacterium acnes colonization, inflammation and immune response may all be involved.<br><br>Q4. How should I treat it? What medicine should I use?<br><br>A4. Topical treatments or/and oral treatments can be useful. Such as topical antibiotics and retinoic acid, and oral antibiotics, isotretinoin and

condition, such as an autoimmune disorder or hormonal imbalance. In others, it may be triggered by environmental factors, such as exposure to sunlight or stress. It is also possible that this condition may be caused by a combination of these factors.<br><br>Q4. How should I treat it? What medicine should I use?<br><br>A4. The best treatment for this skin disease will depend on the specific symptoms and severity of the condition. In most cases, a combination of medications and lifestyle changes is recommended. Topical creams and ointments can be used to reduce inflammation and promote healing. Oral medications, such as antibiotics or retinoids, may also be prescribed to treat this condition. It is important to follow the instructions provided by your healthcare provider and to use medications as directed. In addition to medication, it is important to avoid triggers that may worsen symptoms and to follow a healthy lifestyle, including a balanced diet, regular exercise, and adequate sleep. It is also important to avoid sun exposure and to use sun protection to prevent further damage to the skin.	estrogenic and Antiandrogens drugs can all be applied as appropriate. Although many medications are OCT, medications are best used under medical supervision due to the need for long-term treatment and the side effects of the medications.
Evaluation of Physician	
Evaluation Item	Score (1-5, the higher the better)
SkinGPT-4's diagnosis is correct or relevant.	5
SkinGPT-4's description is informative.	3
SkinGPT-4's suggestions are useful.	4
SkinGPT-4 can help doctors with diagnosis.	5
SkinGPT-4 can help patients to understand their disease better.	5
If SkinGPT-4 can be deployed locally, it protects patients' privacy.	5
Willingness to use SkinGPT-4	4

Case Acne 3

Image ID: acne 3	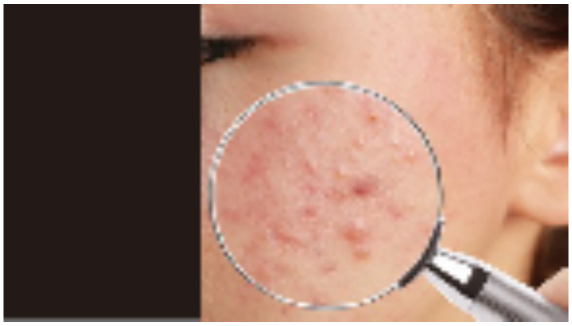
SkinGPT-4	Dermatology Physician
Q1. Could you describe the skin disease in this image for me?<br><br>A1. acne vulgaris<br><br>Q2. You should write a paragraph to list more features you observed in the image.<br><br>A2. This image shows the face of a person with acne. The face is red and bumpy, with several whiteheads and blackheads on the forehead, nose, and cheeks. The skin looks irritated and inflamed.<br><br>Q3. With previous information, please write a paragraph to explain in detail the cause of this skin disease.<br><br>A3. Acne is caused by a combination of factors, including hormonal changes, genetics, and bacteria. The most common cause of acne is an overproduction of sebum, an oily substance produced by the skin's sebaceous glands. The sebum can clog pores, leading to the formation of whiteheads and blackheads. Bacteria, such as Propionibacterium acnes, can also infect the clogged pores, causing inflammation and further breakouts. Additionally, hormonal changes, such as those experienced during puberty, can also contribute to acne.<br><br>Q4. How should I treat it? What medicine should I use?<br><br>A4. Treatment for acne depends on the severity and type of acne. Mild cases of acne can be	Q1. Could you describe the skin disease in this image for me?<br><br>A1. Inflammatory acne vulgaris<br><br>Q2. You should write a paragraph to list more features you observed in the image.<br><br>A2. Scattered follicular red papules, pustules and closed comedones on the cheeks.<br><br>Q3. With previous information, please write a paragraph to explain in detail the cause of this skin disease.<br><br>A3. Acne is a common skin condition which has an multifactorial disorder of the pilosebaceous unit. The development of acne involves a complex interaction of multiple factors, both internal and external to the pilosebaceous apparatus.<br>The pathogenesis of acne is still not fully understood. Genetics, androgen-induced sebum secretion, keratinization of follicular sebaceous ducts. The genetic predisposition, androgen-induced sebum production, keratinization of follicular sebaceous ducts, Propionibacterium acnes colonization, inflammation and immune response may all be involved.<br><br>Q4. How should I treat it? What medicine should I use?<br><br>A4. A retinoic acid-based gel at night; topical application of an antibacterial cream (several times per day) and/or a benzoyl peroxide gel (at night) for red papules and pustules.

treated with over-the-counter products, such as benzoyl peroxide or salicylic acid, while more severe cases may require prescription medications, such as antibiotics or hormone-blocking medications. Lifestyle changes, such as keeping the skin clean, avoiding oil-based cosmetics, and reducing stress, can also help to improve acne. In some cases, isotretinoin, an oral medication, may be prescribed for severe, nodular acne that has not responded to other treatments. It's important to talk to a dermatologist or other healthcare provider to determine the best treatment plan for your specific case of acne.	
Evaluation of Physician	
Evaluation Item	Score (1-5, the higher the better)
SkinGPT-4's diagnosis is correct or relevant.	5
SkinGPT-4's description is informative.	5
SkinGPT-4's suggestions are useful.	5
SkinGPT-4 can help doctors with diagnosis.	5
SkinGPT-4 can help patients to understand their disease better.	5
If SkinGPT-4 can be deployed locally, it protects patients' privacy.	5
Willingness to use SkinGPT-4	5

Case Acne 4

Image ID: acne 4	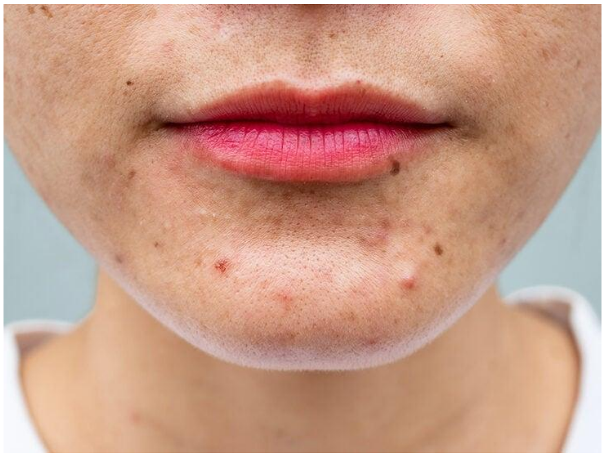
**SkinGPT-4**	**Dermatology Physician**
Q1. Could you describe the skin disease in this image for me?<br><br>A1. acne vulgaris and rosacea<br><br>Q2. You should write a paragraph to list more features you observed in the image.<br><br>A2. In the given image, the person has acne vulgaris, which is characterized by inflammatory papules and pustules on the face, chest, and back. The person also has rosacea, which is a chronic inflammatory skin condition that causes redness, flushing, and pimples on the face, neck, and other parts of the body. The perioral area shows dermatitis, which is an inflammatory skin condition characterized by redness, itching, and blisters around the mouth and nose. The skin appears to have seborrheic dermatitis, which is a common chronic inflammatory skin condition characterized by yellowish, scaly patches on the skin, especially on the scalp, face, and chest. The person may also have eczema, which is a chronic inflammatory skin condition characterized by red, itchy, and scaly patches on the skin. The skin appears to have psoriasis, which is a chronic autoimmune skin condition characterized by red, scaly, and thickened patches on the skin. The person may also have vitiligo, which is a long-term skin condition characterized by patches of the skin losing their pigment. The face and neck have a few seborrheic keratoses, which are small, rough	Q1. Could you describe the skin disease in this image for me?<br><br>A1. acne vulgaris<br><br>Q2. You should write a paragraph to list more features you observed in the image.<br><br>A2. Red papules on the chin.<br><br>Q3. With previous information, please write a paragraph to explain in detail the cause of this skin disease.<br><br>A3. Acne is a common skin condition which has an multifactorial disorder of the pilosebaceous unit. The development of acne involves a complex interaction of multiple factors, both internal and external to the pilosebaceous apparatus.<br>The pathogenesis of acne is still not fully understood. Genetics, androgen-induced sebum secretion, keratinization of follicular sebaceous ducts. The genetic predisposition, androgen-induced sebum production, keratinization of follicular sebaceous ducts, Propionibacterium acnes colonization, inflammation and immune response may all be involved.<br><br>Q4. How should I treat it? What medicine should I use?<br><br>A4. In this case, some Functional-Cosmetics for

Q3. With previous information, please write a paragraph to explain in detail the cause of this skin disease.<br><br>A3. Acne is caused by several factors, including hormonal changes, genetics, and certain medications. The most common cause of acne is an overproduction of sebum by the sebaceous glands in the skin. Sebum is an oily substance that helps to keep the skin and hair moisturized, but when it becomes overproduced, it can clog pores and lead to inflammation and the formation of pimples, blackheads, and whiteheads.<br>Hormonal changes, such as those that occur during puberty, can also contribute to acne. Androgen hormones, such as testosterone, can stimulate the sebaceous glands to produce more sebum, leading to acne. In addition, genetics can play a role in the development of acne. If a person's parents had acne, they are more likely to develop it themselves.<br>Certain medications, such as corticosteroids and lithium, can also cause acne.2 / 2<br><br>Q4. How should I treat it? What medicine should I use?<br><br>A4. Acne can be treated with a variety of methods, depending on the severity of the condition.<br>Mild to moderate acne can often be treated with over-the-counter products, such as benzoyl peroxide or salicylic acid. These products help to unplug pores and reduce inflammation.<br>For more severe acne, prescription medications may be necessary. Antibiotics can be effective at reducing the number of bacteria that cause acne, while retinoids can help to unplug pores and reduce inflammation.<br>In some cases, a combination of treatments may be necessary to effectively manage acne. For example, a person may use a topical medication, such as benzoyl peroxide, in combination with an oral antibiotic or retinoid.<br>In addition to medication, lifestyle changes can also help to manage acne. A person can reduce their stress levels, avoid greasy foods and heavy sun exposure, and maintain good hygiene to help prevent acne breakouts.<br>In some cases, acne scars can be treated with cosmetic procedures, such as laser therapy or dermal fillers. It is important to consult with a	Acne should be used at first. In addition, a retinoic acid-based gel at night; topical application of an antibacterial cream (several times per day) and/or a benzoyl peroxide gel (at night) for red papules and pustules when symptoms get worse.

dermatologist or other healthcare professional to determine the best course of treatment for acne.	
Evaluation of Physician	
Evaluation Item	Score (1-5, the higher the better)
SkinGPT-4's diagnosis is correct or relevant.	5
SkinGPT-4's description is informative.	4
SkinGPT-4's suggestions are useful.	5
SkinGPT-4 can help doctors with diagnosis.	5
SkinGPT-4 can help patients to understand their disease better.	5
If SkinGPT-4 can be deployed locally, it protects patients' privacy.	5
Willingness to use SkinGPT-4	5

Case Acne 5

Image ID: acne5	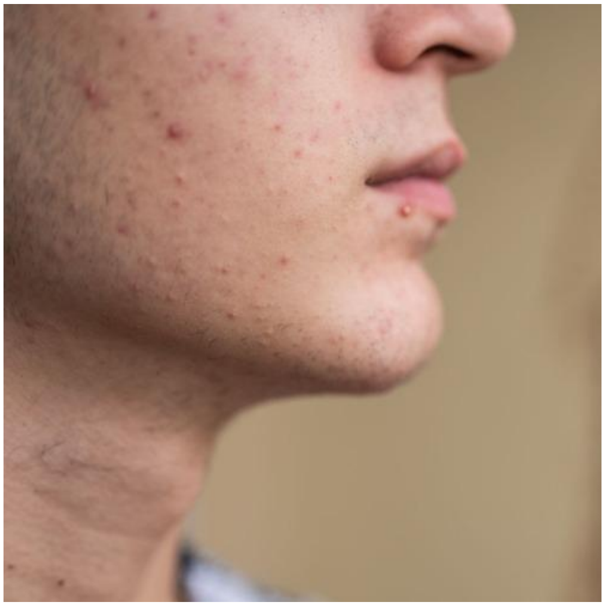
SkinGPT-4	Dermatology Physician
Q1. Could you describe the skin disease in this image for me?<br><br>A1. acne vulgaris and rosacea<br><br>Q2. You should write a paragraph to list more features you observed in the image.<br><br>A2. I observed that the young man has visible signs of acne.<br><br>Q3. With previous information, please write a paragraph to explain in detail the cause of this skin disease.<br><br>A3. Acne is caused by several factors, including excess sebum production, hormonal changes, genetics, and bacteria. Excess sebum, a lubricating oil produced by the skin, can clog pores and cause pimples, blackheads, and whiteheads. Hormonal changes, particularly in teenagers, can also contribute to acne as hormones stimulate the production of sebum. Genetics play a role in acne as certain genes can cause overproduction of sebum. Bacteria, such as Propionibacterium acnes, can also infect clogged pores and cause inflammation, leading to acne. In	Q1. Could you describe the skin disease in this image for me?<br><br>A1. acne vulgaris<br><br>Q2. You should write a paragraph to list more features you observed in the image.<br><br>A2. On the face, there are scattered follicular papules which are red or as the skin tone (closed comedones).<br><br>Q3. With previous information, please write a paragraph to explain in detail the cause of this skin disease.<br><br>A3. Acne is a common skin condition which has an multifactorial disorder of the pilosebaceous unit. The development of acne involves a complex interaction of multiple factors, both internal and external to the pilosebaceous apparatus.<br>The pathogenesis of acne is still not fully understood. Genetics, androgen-induced sebum secretion, keratinization of follicular sebaceous ducts. The genetic predisposition, androgen-induced sebum production, keratinization of follicular sebaceous ducts, Propionibacterium

addition, certain medications, such as corticosteroids and lithium, and underlying medical conditions, such as polycystic ovary syndrome (PCOS), can also cause or worsen acne.<br><br>Q4. How should I treat it? What medicine should I use?<br><br>A4. Acne can be treated with a variety of methods, depending on the severity and type of acne. Here are some of the most common treatments for acne:<br>1. Over-the-counter products: Benzoyl peroxide and salicylic acid are commonly used to treat acne. They work by unclogging pores and reducing bacteria.<br>2. Prescription medications: Antibiotics, such as doxycycline and minocycline, and retinoids, such as adapalene and tretinoin, are commonly prescribed to treat acne. They work by reducing bacteria and unclogging pores.<br>3. Topical treatments: Topical treatments, such as retinoids and azelaic acid, can also be used to treat acne. They work by unclogging pores and reducing bacteria.<br>4. Oral medications: In some cases, oral medications, such as spironolactone and the contraceptive pill, may be prescribed to treat acne.<br>5. Light therapy: Blue light therapy can also be used to treat acne by killing bacteria and reducing inflammation.<br>6. Lifestyle changes: Maintaining good hygiene, avoiding excessive use of cosmetics, and getting regular exercise can also help improve acne symptoms.	acnes colonization, inflammation and immune response may all be involved.<br><br>Q4. How should I treat it? What medicine should I use?<br><br>A4. Benzoyl peroxide and salicylic acid are commonly used to treat acne (on red papules); a retinoic acid-based gel at night.
Evaluation of Physician	
Evaluation Item	Score (1-5, the higher the better)
SkinGPT-4's diagnosis is correct or relevant.	5
SkinGPT-4's description is informative.	5
SkinGPT-4's suggestions are useful.	5
SkinGPT-4 can help doctors with diagnosis.	5

SkinGPT-4 can help patients to understand their disease better.	5
If SkinGPT-4 can be deployed locally, it protects patients' privacy.	5
Willingness to use SkinGPT-4	5

Case Acne 6

Image ID: acne6	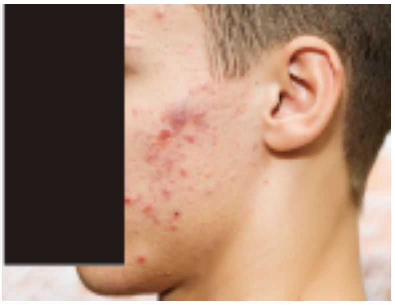
**SkinGPT-4**	**Dermatology Physician**
Q1. Could you describe the skin disease in this image for me?<br><br>A1. acne scars and redness and bumps and pimples and blackheads and whiteheads and inflammation and infections<br><br>Q2. You should write a paragraph to list more features you observed in the image.<br><br>A2. From the given image, it is evident that the person has acne scars on their face. The scars are characterized by uneven skin texture, pitting, and discoloration. The skin around the scars appears red and inflamed, indicating the presence of redness and inflammation. The person also has blackheads and whiteheads on their nose and some pimples on their cheeks. There are some bumps and roughness on their forehead and chin. The overall appearance of the skin is dry, flaky, and uneven, with some areas appearing lighter or darker than the surrounding skin. There are some hyperpigmentation spots on the cheeks and nose, which are more pronounced in some areas. The image also suggests that the person has some scarring on their face, which might have been caused by acne or other skin conditions.<br>It is important to note that acne scars are not just a cosmetic concern but can also have a significant impact on a person's self-esteem and mental health. Therefore, it is essential to seek medical advice and treatment to address the issue and improve the appearance of the skin. A dermatologist can provide a proper diagnosis and recommend appropriate treatments, such as topical creams, laser therapy, or surgery, to	Q1. Could you describe the skin disease in this image for me?<br><br>A1. Acne; Acne scars<br><br>Q2. You should write a paragraph to list more features you observed in the image.<br><br>A2. Scattered follicular red papules and red scars on the cheek.<br><br>Q3. With previous information, please write a paragraph to explain in detail the cause of this skin disease.<br><br>A3. Acne is a common skin condition which has an multifactorial disorder of the pilosebaceous unit. The development of acne involves a complex interaction of multiple factors, both internal and external to the pilosebaceous apparatus.<br>The pathogenesis of acne is still not fully understood. Genetics, androgen-induced sebum secretion, keratinization of follicular sebaceous ducts. The genetic predisposition, androgen-induced sebum production, keratinization of follicular sebaceous ducts, Propionibacterium acnes colonization, inflammation and immune response may all be involved.<br><br>Q4. How should I treat it? What medicine should I use?<br><br>A4. The patient in this case required treatment with oral antibiotics. If this is not effective, oral isotretinoin may be given. Topical anti-antibiotics creams/gel, retinoic acid and benzoyl peroxide gel are also needed.

reduce the appearance of acne scars and other skin concerns.<br><br>Q3. With previous information, please write a paragraph to explain in detail the cause of this skin disease.<br><br>A3. The skin disease seen in the given image is likely acne vulgaris, which is the most common type of acne. Acne vulgaris is a chronic skin condition characterized by the formation of comedones (whiteheads and blackheads), pimples, cysts, and scars on the face, neck, chest, and back. The disease occurs when the hair follicles on the face, chest, and back become clogged with sebum, dead skin cells, and bacteria, causing inflammation and infection.<br><br>The exact cause of acne vulgaris is not known, but it is believed to be a result of a combination of genetic, hormonal, and environmental factors. Hormonal changes during puberty and pregnancy can stimulate the production of oil by the sebaceous glands, leading to clogged pores and acne. Genetics also play a role, as people with a family history of acne are more likely to develop the condition. Additionally, certain medications, such as corticosteroids, lithium, and halogens, can cause acne.<br><br>Q4. How should I treat it? What medicine should I use?<br><br>A4. Acne can be treated with a variety of methods, depending on the severity of the condition and the individual's skin type and concerns. Mild to moderate acne can often be treated with over-the-counter (OTC) products, such as topical creams, gels, and lotions. These products can help to reduce inflammation and prevent the formation of new pimples. For more severe cases of acne, prescription medications may be necessary. These can include oral antibiotics, which help to reduce bacteria on the skin, and topical medications, which can help to reduce oil production and prevent the formation of comedones. Other treatments for acne can include chemical peels, laser therapy, and extraction of pimples. It is important to talk to a dermatologist or other healthcare provider to determine the best treatment plan for your individual needs and concerns.	

Evaluation of Physician	
**Evaluation Item**	**Score (1-5, the higher the better)**
SkinGPT-4's diagnosis is correct or relevant.	5
SkinGPT-4's description is informative.	5
SkinGPT-4's suggestions are useful.	5
SkinGPT-4 can help doctors with diagnosis.	5
SkinGPT-4 can help patients to understand their disease better.	5
If SkinGPT-4 can be deployed locally, it protects patients' privacy.	5
Willingness to use SkinGPT-4	5

Case Acne 7

Image ID: acne7	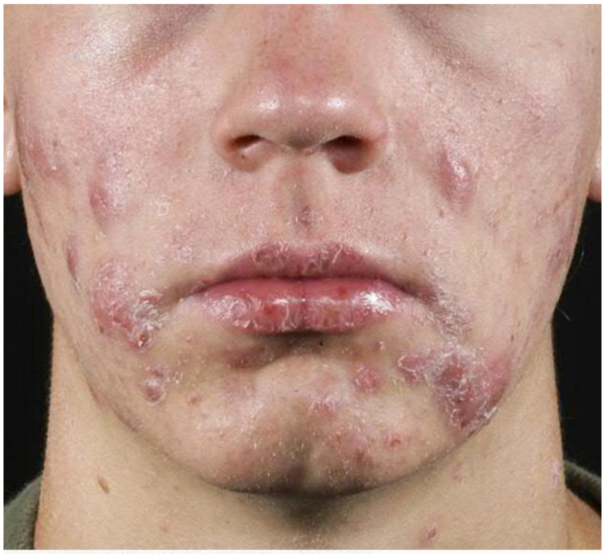<br>© MAYO FOUNDATION FOR MEDICAL EDUCATION AND RESEARCH. ALL RIGHTS RESERVED.
SkinGPT-4	Dermatology Physician
Q1. Could you describe the skin disease in this image for me?<br><br>A1. acne and rosacea and perioral dermatitis and drug eruptions<br><br>Q2. You should write a paragraph to list more features you observed in the image.<br><br>A2. In the image, the person has severe acne on their face and chest, with large red bumps and blackheads. They also have red, flaky skin on their forehead and around their nose and mouth. Their skin appears dry and scaly, with small, white, bumpy lesions on their face and neck. There are several small, pus - filled pimples on their forehead and around their nose. Their eyebrows are thinned and their eyelashes appear to be missing. Overall, their skin appears to be inflamed, red, and covered in bumps and lesions.<br><br>Q3. With previous information, please write a paragraph to explain in detail the cause of this skin disease.<br><br>A3. Acne vulgaris is caused by an overproduction of sebum, a greasy substance produced by the skin's sebaceous glands. The excess sebum can clog the pores, leading to inflammation and the	Q1. Could you describe the skin disease in this image for me?<br><br>A1. acne scar, cystic acne<br><br>Q2. You should write a paragraph to list more features you observed in the image.<br><br>A2. Red nodules, papules and scars on the cheeks/jaw with some scabs on the surface.<br><br>Q3. With previous information, please write a paragraph to explain in detail the cause of this skin disease.<br><br>A3. Acne is a common skin condition which has an multifactorial disorder of the pilosebaceous unit. The development of acne involves a complex interaction of multiple factors, both internal and external to the pilosebaceous apparatus.<br>The pathogenesis of acne is still not fully understood. Genetics, androgen-induced sebum secretion, keratinization of follicular sebaceous ducts. The genetic predisposition, androgen-induced sebum production, keratinization of follicular sebaceous ducts, Propionibacterium acnes colonization, inflammation and immune response may all be involved.

formation of acne lesions. Hormonal changes, genetics, and other factors can contribute to the development of acne.<br><br>Diet and stress can also play a role in acne. Foods that are high in sugar and fat can contribute to inflammation and acne breakouts. Stress can also cause increased production of hormones that can lead to acne.<br><br>Q4. How should I treat it? What medicine should I use?<br><br>A4. Acne vulgaris can be treated with various methods, including:<br>1. Over-the-counter treatments: Topical medications such as creams, gels, and cleansers containing benzoyl peroxide, salicylic acid, or alpha-hydroxy acid can help reduce acne symptoms.<br>2. Prescription medications: Topical or oral antibiotics, hormone therapy, and retinoids are available by prescription and can help reduce inflammation and prevent new acne lesions from forming.<br>3. Lifestyle changes: Keeping the skin clean, avoiding greasy and sugary foods, reducing stress, and avoiding excessive sun exposure can help prevent acne breakouts.<br>4. Light therapy: Blue light therapy and red light therapy can help reduce acne inflammation and bacteria.<br>5. Chemical peels: A chemical solution is applied to the skin to remove the top layer and stimulate new skin growth. This can help improve the appearance of acne scars and reduce inflammation.<br>It is important to consult with a dermatologist or other healthcare provider to determine the best treatment plan for acne vulgaris.	Q4. How should I treat it? What medicine should I use?<br><br>A4. Oral antibiotic and topical retinoid ±Benzoyl Peroxide should be used; Oral isotretinoin also can be used.
Evaluation of Physician	
Evaluation Item	Score (1-5, the higher the better)
SkinGPT-4's diagnosis is correct or relevant.	5
SkinGPT-4's description is informative.	4

SkinGPT-4's suggestions are useful.	5
SkinGPT-4 can help doctors with diagnosis.	5
SkinGPT-4 can help patients to understand their disease better.	5
If SkinGPT-4 can be deployed locally, it protects patients' privacy.	5
Willingness to use SkinGPT-4	5

Case Acne 8

Image ID: acne8	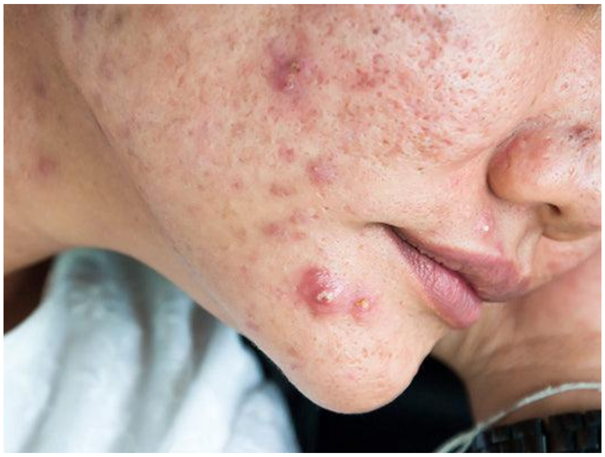
**SkinGPT-4**	**Dermatology Physician**
Q1. Could you describe the skin disease in this image for me?<br><br>A1. acne vulgaris<br><br>Q2. You should write a paragraph to list more features you observed in the image.<br><br>A2. In the image, the person has a mild case of acne vulgaris, which is characterized by blackheads and whiteheads on the face, neck, and chest. They may also have pustules, papules, and nodules. The skin lesions are accompanied by a mild inflammatory response and scarring. The condition can be treated with topical creams and oral medications.<br><br>Q3. With previous information, please write a paragraph to explain in detail the cause of this skin disease.<br><br>A3. Acne vulgaris is caused by a combination of factors, including genetics, hormones, and skin type. In mild cases, it is caused by excessive sebum production and dead skin cells clogging pores. The bacterium Propionibacterium acnes also plays a role in the development of acne by infecting the clogged pores and causing inflammation. Hormonal changes, such as those that occur during puberty or pregnancy, can also lead to an increase in acne severity. Stress, certain medications, and a diet high in sugar and	Q1. Could you describe the skin disease in this image for me?<br><br>A1. Inflammatory acne vulgaris<br><br>Q2. You should write a paragraph to list more features you observed in the image.<br><br>A2. Papules, obvious pustules, and atrophic scars are present..<br><br>Q3. With previous information, please write a paragraph to explain in detail the cause of this skin disease.<br><br>A3. Acne is a common skin condition which has an multifactorial disorder of the pilosebaceous unit. The development of acne involves a complex interaction of multiple factors, both internal and external to the pilosebaceous apparatus.<br>The pathogenesis of acne is still not fully understood. Genetics, androgen-induced sebum secretion, keratinization of follicular sebaceous ducts. The genetic predisposition, androgen-induced sebum production, keratinization of follicular sebaceous ducts, Propionibacterium acnes colonization, inflammation and immune response may all be involved.<br><br>Q4. How should I treat it? What medicine should I use?

fat can also contribute to acne formation. In general, acne vulgaris is a chronic inflammatory skin condition that affects many people and can be managed with proper skincare and medical treatment.<br><br>Q4. How should I treat it? What medicine should I use?<br><br>A4. Treatment for acne vulgaris typically involves a combination of lifestyle changes, topical medications, and oral medications.<br>For mild to moderate acne, over-the-counter topical medications such as benzoyl peroxide, salicylic acid, and alpha-hydroxy acids can be effective in reducing inflammation and unclogging pores. These medications can be used alone or in combination with other ingredients such as moisturizers and antibiotics.<br>For more severe cases of acne, prescription medications may be necessary. These include oral antibiotics, such as doxycycline, minocycline, and erythromycin, which target the bacteria that cause acne. Retinoids, such as adapalene and tretinoin, can also be prescribed to reduce inflammation and promote cell turnover.<br>In some cases, a combination of topical and oral medications may be the most effective approach. It's important to consult a dermatologist or other healthcare provider to determine the best course of treatment for your individual needs and to ensure that the treatment is safe and effective.	A4. Topical treatments or/and oral treatments can be useful. Such as topical antibiotics and retinoic acid, and oral antibiotics, isotretinoin and estrogenic and Antiandrogens drugs can all be applied as appropriate.
Evaluation of Physician	
Evaluation Item	Score (1-5, the higher the better)
SkinGPT-4's diagnosis is correct or relevant.	5
SkinGPT-4's description is informative.	5
SkinGPT-4's suggestions are useful.	5
SkinGPT-4 can help doctors with diagnosis.	5
SkinGPT-4 can help patients to understand their disease better.	5

If SkinGPT-4 can be deployed locally, it protects patients’ privacy.	5
Willingness to use SkinGPT-4	5

Case Acne 9

Image ID: acne9	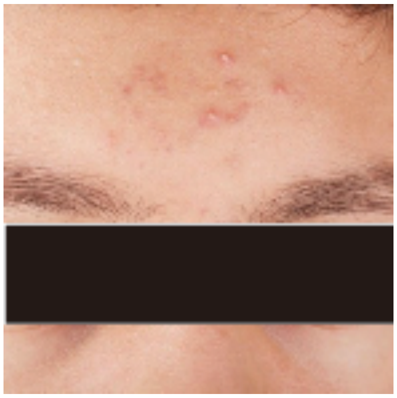
SkinGPT-4	Dermatology Physician
Q1. Could you describe the skin disease in this image for me?<br><br>A1. This could be a skin disease image of acne closed comedo.<br><br>Q2. You should write a paragraph to list more features you observed in the image.<br><br>A2. The image shows a person with acne closed comedo, a common skin condition characterized by inflamed red pimples on the face, neck, and back.<br><br>Q3. With previous information, please write a paragraph to explain in detail the cause of this skin disease.<br><br>A3. Based on the features I observed in the image, this appears to be a case of acne closed comedo. Acne is a common skin condition that occurs when the pores on the skin become clogged with dead skin cells, oil, and bacteria. In this case, the comedo (blackhead) has become infected, leading to the formation of a closed comedo (whitehead). The cause of acne is multifactorial, including hormonal imbalances, excess oil production, clogged pores, and bacterial infections. Treatment options for acne include topical and oral antibiotics, retinoids, and hormonal therapies. It is important to consult a dermatologist for proper diagnosis and treatment.<br><br>Q4. How should I treat it? What medicine should I	Q1. Could you describe the skin disease in this image for me?<br><br>A1. acne vulgaris<br><br>Q2. You should write a paragraph to list more features you observed in the image.<br><br>A2. Papules, obvious pustules on the forehead<br><br>Q3. With previous information, please write a paragraph to explain in detail the cause of this skin disease.<br><br>A3. Acne is a common skin condition which has an multifactorial disorder of the pilosebaceous unit. The development of acne involves a complex interaction of multiple factors, both internal and external to the pilosebaceous apparatus.<br>The pathogenesis of acne is still not fully understood. Genetics, androgen-induced sebum secretion, keratinization of follicular sebaceous ducts. The genetic predisposition, androgen-induced sebum production, keratinization of follicular sebaceous ducts, Propionibacterium acnes colonization, inflammation and immune response may all be involved.<br><br>Q4. How should I treat it? What medicine should I use?<br><br>A4. Topical treatment may be effective: topical retinoid + topical antimicrobic, and topical retinoid, Azelaic acid and Salicylic acid for alternative.

use?<br><br>A4. Over-the-counter (OTC) treatments such as benzoyl peroxide or salicylic acid may be effective in treating this condition. However, if the condition persists or worsens, it is important to consult a dermatologist for proper diagnosis and treatment. The dermatologist may prescribe topical or oral antibiotics, retinoids, or hormonal therapies. It is important to follow the treatment plan recommended by the dermatologist and to avoid picking or popping the comedones, as this can lead to further inflammation and scarring.	
Evaluation of Physician	
Evaluation Item	Score (1-5, the higher the better)
SkinGPT-4's diagnosis is correct or relevant.	5
SkinGPT-4's description is informative.	5
SkinGPT-4's suggestions are useful.	5
SkinGPT-4 can help doctors with diagnosis.	5
SkinGPT-4 can help patients to understand their disease better.	5
If SkinGPT-4 can be deployed locally, it protects patients' privacy.	5
Willingness to use SkinGPT-4	5

Case Acne 10

<table>
<tr><td>Image ID: acne10</td><td>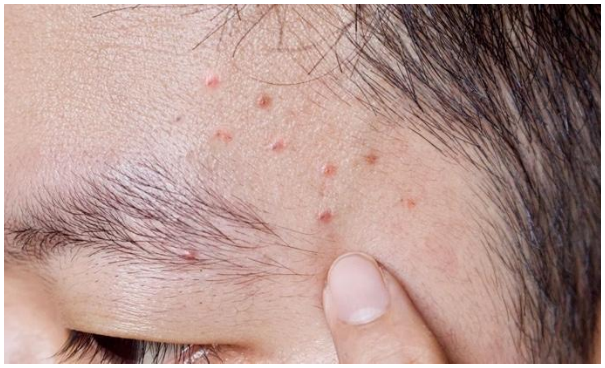</td></tr>
<tr><th>SkinGPT-4</th><th>Dermatology Physician</th></tr>
<tr><td>Q1. Could you describe the skin disease in this image for me?

A1. acne scars

Q2. You should write a paragraph to list more features you observed in the image.

A2. The image depicts a young man with acne on his face, particularly on the forehead and nose. The acne appears to be mild and consisting of whiteheads and blackheads. The skin appears smooth and unblemished on the cheeks and chin. The skin tone is fair and the facial features are symmetrical. The eyes are small and almond shaped with a slight epicanthic fold. The eyebrows are thick and straight and the lips are thin and straight. The nose is straight and the nostrils are slightly flared. The hair is short and dark.

Q3. With previous information, please write a paragraph to explain in detail the cause of this skin disease.

A3. The skin disease depicted in the image is likely caused by a combination of factors. Genetics and hormones may play a role in the development of acne, as it is often seen in individuals with a family history of the condition. The image shows mild acne, which may be caused by an excess production of sebum, a greasy substance produced by the skin, and dead skin cells that clog pores and encourage bacterial growth. Hormonal changes, such as those that</td><td>Q1. Could you describe the skin disease in this image for me?

A1. Acne(mild)；scar(?)

Q2. You should write a paragraph to list more features you observed in the image.

A2. Inflammatory papules on the corners of the forehead

Q3. With previous information, please write a paragraph to explain in detail the cause of this skin disease.

A3. Acne is a common skin condition which has an multifactorial disorder of the pilosebaceous unit. The development of acne involves a complex interaction of multiple factors, both internal and external to the pilosebaceous apparatus.
The pathogenesis of acne is still not fully understood. Genetics, androgen-induced sebum secretion, keratinization of follicular sebaceous ducts. The genetic predisposition, androgen-induced sebum production, keratinization of follicular sebaceous ducts, Propionibacterium acnes colonization, inflammation and immune response may all be involved.

Q4. How should I treat it? What medicine should I use?

A4. Topical retinoid and/or topical antimicrobic may be effective.</td></tr>
</table>

occur during puberty or pregnancy, can also contribute to the development of acne. The skin disease in the image may also be caused by other factors such as poor diet, stress, and a lack of proper skin care.<br><br>Q4. How should I treat it? What medicine should I use?<br><br>A4. Treatment for mild acne can include over-the-counter medications such as topical creams and gels containing benzoyl peroxide or salicylic acid, which help to unclog pores and reduce bacteria. It is important to follow the instructions on the packaging and to use the medication consistently to see improvement. In addition to medication, maintaining a clean and healthy lifestyle can help to prevent the development of acne. This includes regular skin care, a healthy diet, and reducing stress. If over-the-counter treatments are not effective, a dermatologist may prescribe stronger medications such as oral antibiotics or retinoids. It is important to consult a dermatologist for proper diagnosis and treatment.<br>Please note that the use of any medicine should be under the guidance of a dermatologist or a qualified healthcare professional.	
Evaluation of Physician	
Evaluation Item	Score (1-5, the higher the better)
SkinGPT-4's diagnosis is correct or relevant.	3
SkinGPT-4's description is informative.	4
SkinGPT-4's suggestions are useful.	5
SkinGPT-4 can help doctors with diagnosis.	5
SkinGPT-4 can help patients to understand their disease better.	4
If SkinGPT-4 can be deployed locally, it protects patients' privacy.	5
Willingness to use SkinGPT-4	4

Case Rosacea 1

Image ID: rosacea1	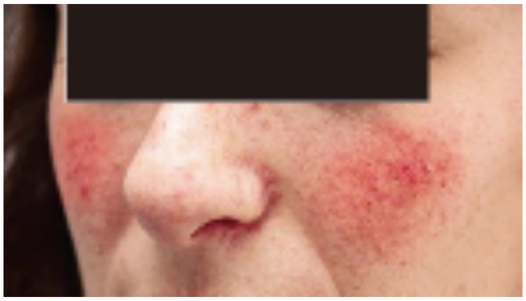
**SkinGPT-4**	**Dermatology Physician**
Q1. Could you describe the skin disease in this image for me?<br><br>A1. rosacea<br><br>Q2. You should write a paragraph to list more features you observed in the image.<br><br>A2. It looks like this person has rosacea, which is a chronic skin condition that causes redness and inflammation on the face. It can also cause pimples and thick skin on the nose, forehead, cheeks and chin. It can also cause eye problems, such as dry eye and irritation. The redness and inflammation of rosacea is caused by the blood vessels in the skin dilating and becoming more visible. It can be triggered by factors such as sun exposure, alcohol consumption, spicy foods, and stress. There is no cure for rosacea, but treatments can help to reduce symptoms. Treatments may include topical creams, oral medications, and laser therapy. It is important to avoid triggers and protect the skin from the sun with sunscreen.<br>The specific cause of rosacea is not known, but it is thought to be related to the immune system and blood vessels in the skin.<br><br>Q3. With previous information, please write a paragraph to explain in detail the cause of this skin disease.<br><br>A3. Rosacea is caused by a combination of genetic and environmental factors. It is believed that people with fair skin, who have a history of flushing or blushing easily, are more likely to develop rosacea.	Q1. Could you describe the skin disease in this image for me?<br><br>A1. Rosacea<br><br>Q2. You should write a paragraph to list more features you observed in the image.<br><br>A2. Erythema of the medial and lateral cheeks is seen. In this patient, there are no telangiectasias, indicating mild disease.<br><br>Q3. With previous information, please write a paragraph to explain in detail the cause of this skin disease.<br><br>A3. The exact pathogenesis of rosacea is unknown. The disorder which is known today as rosacea will probably be shown in time to comprise several similar, possibly related, but different clinical conditions, each with separate predominant pathogenic mechanisms. Aberrant innate immune system, epidermal barrier dysfunction, Vascular changes, neurogenic inflammation, ultraviolet radiation and microbes may involved.<br><br>Q4. How should I treat it? What medicine should I use?<br><br>A4. We recommend facial skin care: (1) particularly useful as this subtype is prone to skin irritation and 'sensitivity'; (2) UVR may potentiate dermal matrix damage; (3) use of lasers (e.g. pulsed dye, potassium titanyl phosphate) as well as intense pulsed light may improve symptoms.

There are several triggers that can worsen rosacea symptoms, including:<br><br>* Sun exposure<br>* Alcohol consumption<br>* Spicy foods<br>* Stress<br>* Certain skincare products<br>Rosacea can also be associated with other conditions such as ocular rosacea, which affects the eyes, and rhinophyma, which is a more severe form of rosacea that affects the nose. If you have rosacea, it is important to avoid triggers and protect your skin from the sun. It is also important to see a dermatologist for proper diagnosis and treatment.<br><br>Q4. How should I treat it? What medicine should I use?<br><br>A4. Treatment for rosacea can include a combination of medical and lifestyle changes. The goal of treatment is to reduce redness and inflammation, prevent flare-ups, and improve the appearance of the skin.<br>Medical treatments for rosacea may include:<br>* Topical creams and gels containing ingredients such as metronidazole, brimonidine, or azelaic acid<br>* Oral antibiotics, such as doxycycline, to reduce inflammation and bacteria in the skin<br>* Systemic medications, such as isotretinoin, for severe cases of rosacea<br>Lifestyle changes that can help manage rosacea include:<br>* Avoiding triggers such as sun exposure, alcohol consumption, spicy foods, and stress<br>* Using gentle skincare products that are fragrance-free and formulated for sensitive skin<br>* Protecting the skin from the sun with sunscreen<br>* Keeping the skin clean and moisturized<br>It is important to see a dermatologist for proper diagnosis and treatment, as they can recommend the most appropriate treatment plan for you based on the severity of your rosacea and any underlying conditions you may have.	
Evaluation of Physician	
Evaluation Item	Score (1-5, the higher the better)

SkinGPT-4's diagnosis is correct or relevant.	5
SkinGPT-4's description is informative.	5
SkinGPT-4's suggestions are useful.	5
SkinGPT-4 can help doctors with diagnosis.	5
SkinGPT-4 can help patients to understand their disease better.	5
If SkinGPT-4 can be deployed locally, it protects patients' privacy.	5
Willingness to use SkinGPT-4	5

Case Rosacea 2

<table>
<tr><td>Image ID: rosacea2</td><td>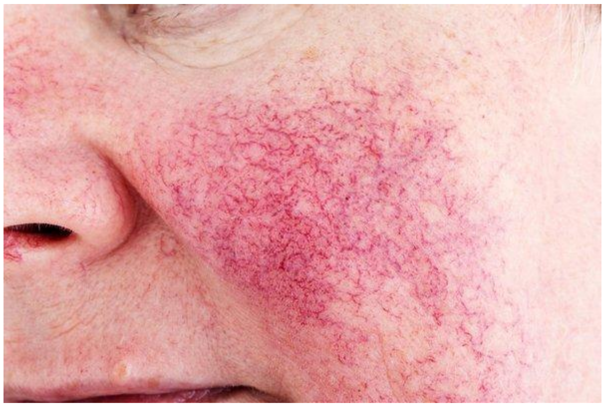</td></tr>
<tr><th>SkinGPT-4</th><th>Dermatology Physician</th></tr>
<tr><td>Q1. Could you describe the skin disease in this image for me?

A1. rosacea

Q2. You should write a paragraph to list more features you observed in the image.

A2. It looks like the woman has rosacea, a chronic skin condition that causes redness and inflammation on the face. The redness is most common on the cheeks, nose, and chin, but it can also affect the ears, neck, and scalp. The condition can be accompanied by bumps and pimples, and the skin may be thicker and more oily than normal. The redness is caused by dilated blood vessels that are visible just under the skin. The condition is more common in people with fair skin and can be triggered by things like sun exposure, alcohol, spicy foods, or stress. There is no cure for rosacea, but treatments can help to manage symptoms and improve the appearance of the skin. These treatments may include topical creams, oral medications, and laser therapy. It’s important to see a dermatologist for proper diagnosis and treatment.

Q3. With previous information, please write a paragraph to explain in detail the cause of this skin disease.

A3. Rosacea is caused by a combination of genetic and environmental factors. The exact cause of rosacea is not yet fully understood, but it</td><td>Q1. Could you describe the skin disease in this image for me?

A1. Rosacea

Q2. You should write a paragraph to list more features you observed in the image.

A2. Erythema and angiotelectasis of the medial and lateral cheeks could be found.

Q3. With previous information, please write a paragraph to explain in detail the cause of this skin disease.

A3. The exact pathogenesis of rosacea is unknown. The disorder which is known today as rosacea will probably be shown in time to comprise several similar, possibly related, but different clinical conditions, each with separate predominant pathogenic mechanisms. Aberrant innate immune system, epidermal barrier dysfunction, Vascular changes, neurogenic inflammation, ultraviolet radiation and microbes may involved.

Q4. How should I treat it? What medicine should I use?

A4. We recommend facial skin care: (1) particularly useful as this subtype is prone to skin irritation and 'sensitivity'; (2) UVR may potentiate dermal matrix damage; (3) use of lasers (e.g. pulsed dye, potassium titanyl phosphate) as well</td></tr>
</table>

is thought to be related to the immune system, blood vessels, and certain bacteria that live on the skin.<br>Some research suggests that there may be a genetic component to rosacea, as people with a family history of the condition are more likely to develop it themselves. Certain triggers can also cause rosacea to flare up, including sun exposure, alcohol, spicy foods, and stress.<br>The condition is more common in people with fair skin, and it tends to affect women more often than men. If you have rosacea, it's important to avoid triggers and follow a treatment plan prescribed by a dermatologist to manage symptoms and prevent flare-ups.<br><br>Q4. How should I treat it? What medicine should I use?<br><br>A4. Treatment for rosacea depends on the severity of the condition and the individual's specific symptoms. Some common treatment options include:<br>1. Topical creams: These can help to reduce redness and inflammation on the surface of the skin. Examples include metronidazole, azelaic acid, and brimonidine.<br>2. Oral medications: These may be prescribed for more severe cases of rosacea. Examples include tetracycline, doxycycline, and minocycline.<br>3. Laser therapy: This can be used to improve the appearance of the skin and reduce redness. Dermatologists use different types of lasers depending on the patient's skin type and specific symptoms.<br>4. Lifestyle changes: Avoiding triggers such as sun exposure, spicy foods, and alcohol can help to reduce flare-ups of rosacea. Gentle skin care is also important, as harsh products can irritate the skin.<br>It's important to work with a dermatologist to develop a treatment plan that is tailored to your specific needs. In addition to treatment, it's important to practice good skin care habits and avoid triggers to prevent flare-ups and improve the overall appearance of the skin.	as intense pulsed light may improve symptoms.
Evaluation of Physician	
Evaluation Item	Score (1-5, the higher the better)

SkinGPT-4's diagnosis is correct or relevant.	5
SkinGPT-4's description is informative.	5
SkinGPT-4's suggestions are useful.	5
SkinGPT-4 can help doctors with diagnosis.	5
SkinGPT-4 can help patients to understand their disease better.	5
If SkinGPT-4 can be deployed locally, it protects patients' privacy.	5
Willingness to use SkinGPT-4	5

Case Rosacea 3

Image ID: rosacea3	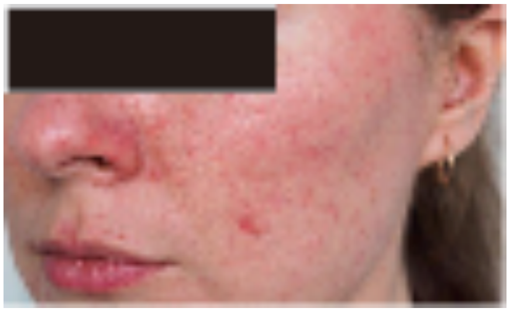
SkinGPT-4	Dermatology Physician
Q1. Could you describe the skin disease in this image for me?<br><br>A1. rosacea<br><br>Q2. You should write a paragraph to list more features you observed in the image.<br><br>A2. This is a common condition known as rosacea. It is characterized by redness of the face, particularly around the nose, cheeks, and forehead. The skin may feel inflamed, with visible blood vessels and pustules. There may be a burning or itching sensation, and the skin may be sensitive to certain products. The cause of rosacea is not well understood, but it is thought to be related to a combination of genetic and environmental factors, such as sun exposure, stress, and certain trigger foods. Treatment options include topical creams, oral antibiotics, and lifestyle changes. It is important to avoid triggers and to protect the skin from sun damage. It is also important to consult a dermatologist for proper diagnosis and treatment.<br><br>Q3. With previous information, please write a paragraph to explain in detail the cause of this skin disease.<br><br>A3. Rosacea is a common skin condition that is caused by a combination of genetic and environmental factors. The exact cause of rosacea is not yet fully understood, but it is thought to be related to the blood vessels in the skin becoming more visible and inflamed. This can be caused by a variety of factors, including:<br>1. Genetics: People with a family history of	Q1. Could you describe the skin disease in this image for me?<br><br>A1. Rosacea<br><br>Q2. You should write a paragraph to list more features you observed in the image.<br><br>A2. Erythema and red papules can be seen on the cheeks and nose, without any involvement around the eyes.<br><br>Q3. With previous information, please write a paragraph to explain in detail the cause of this skin disease.<br><br>A3. The exact pathogenesis of rosacea is unknown. The disorder which is known today as rosacea will probably be shown in time to comprise several similar, possibly related, but different clinical conditions, each with separate predominant pathogenic mechanisms. Aberrant innate immune system, epidermal barrier dysfunction, Vascular changes, neurogenic inflammation, ultraviolet radiation and microbes may involve.<br><br>Q4. How should I treat it? What medicine should I use?<br><br>A4. In addition to basic treatment, the use of topical antibacterial drugs such as Metronidazole, Azeleic acid and Clindamycin.

rosacea are more likely to develop the condition.<br>2. Sun exposure: Exposure to the sun can cause the blood vessels in the skin to become more visible and inflamed, leading to rosacea.<br>3. Triggers: Certain foods, alcohol, spicy foods, and hot beverages can cause flare-ups of rosacea.<br>4. Demodex mites: These tiny mites that live on the skin can cause inflammation and irritation, which can lead to rosacea.<br>5. Hormonal changes: Rosacea can be triggered by hormonal changes, such as menopause or pregnancy.<br>There is no known cure for rosacea, but there are many treatments available to help manage the symptoms and reduce the risk of flare-ups. These include topical creams, oral antibiotics, lifestyle changes, and laser therapy.<br><br>Q4. How should I treat it? What medicine should I use?<br><br>A4. Treatment for rosacea can help manage the symptoms and reduce the risk of flare-ups. The most effective treatment will depend on the severity and type of rosacea, and may include a combination of the following:<br>1. Topical creams: These can help reduce inflammation and redness, and may contain ingredients such as azelaic acid, metronidazole, or brimonidine.<br>2. Oral antibiotics: These can help reduce inflammation and redness, and may be prescribed if the rosacea is severe or does not respond to topical treatments.<br>3. Lifestyle changes: Avoiding triggers such as spicy foods, alcohol, and sun exposure can help prevent flare-ups.<br>4. Laser therapy: This can help reduce the appearance of redness and improve the overall appearance of the skin.<br>5. Medications for underlying condition: If the rosacea is caused by an underlying condition such as demodex mites or hormonal imbalances, treatment for that condition may be necessary.<br>It is important to consult a dermatologist for proper diagnosis and treatment. They will be able to recommend the most appropriate treatment plan based on your individual needs and the severity of your rosacea.	
Evaluation of Physician	

Evaluation Item	Score (1-5, the higher the better)
SkinGPT-4's diagnosis is correct or relevant.	5
SkinGPT-4's description is informative.	5
SkinGPT-4's suggestions are useful.	5
SkinGPT-4 can help doctors with diagnosis.	5
SkinGPT-4 can help patients to understand their disease better.	5
If SkinGPT-4 can be deployed locally, it protects patients' privacy.	5
Willingness to use SkinGPT-4	5

Case Rosacea 4

Image ID: rosacea4	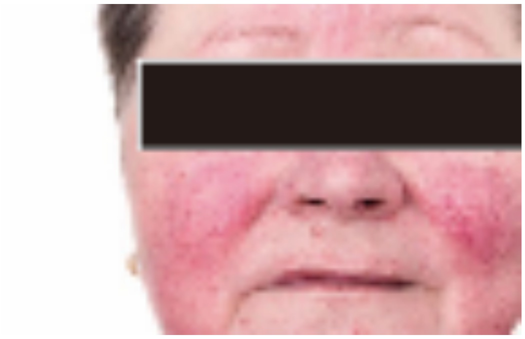
SkinGPT-4	Dermatology Physician
Q1. Could you describe the skin disease in this image for me?<br><br>A1. Rosacea<br><br>Q2. You should write a paragraph to list more features you observed in the image.<br><br>A2. Rosacea is a chronic skin condition that causes redness and inflammation of the face, particularly the cheeks, nose, and forehead. It can also affect the eyes and neck. The skin may appear red and swollen, with small red bumps or pustules. The face may feel hot and itchy, and the eyes may water or burn. Rosacea can be triggered by factors such as sun exposure, stress, exercise, and certain foods or drinks. Treatment includes topical creams and oral medications, as well as lifestyle changes to reduce triggers. Over the counter creams can help to reduce inflammation and redness, while prescription medications may be needed for more severe cases. Laser therapy is also an option for some people. To prevent rosacea, it is important to avoid triggers, wear sunscreen, and practice good hygiene. Photos of rosacea may be helpful for diagnosis and monitoring of the condition.<br><br>Q3. With previous information, please write a paragraph to explain in detail the cause of this skin disease.<br><br>A3. Rosacea is a chronic skin condition that is caused by a combination of genetic and environmental factors. The exact cause of rosacea is not known, but it is believed to involve	Q1. Could you describe the skin disease in this image for me?<br><br>A1. Rosacea<br><br>Q2. You should write a paragraph to list more features you observed in the image.<br><br>A2. Erythema and telangiectasias on the medial and lateral cheeks is seen.<br><br>Q3. With previous information, please write a paragraph to explain in detail the cause of this skin disease.<br><br>A3. The exact pathogenesis of rosacea is unknown. The disorder which is known today as rosacea will probably be shown in time to comprise several similar, possibly related, but different clinical conditions, each with separate predominant pathogenic mechanisms. Aberrant innate immune system, epidermal barrier dysfunction, Vascular changes, neurogenic inflammation, ultraviolet radiation and microbes may involved.<br><br>Q4. How should I treat it? What medicine should I use?<br><br>A4. We recommend facial skin care: (1) particularly useful as this subtype is prone to skin irritation and 'sensitivity'; (2) UVR may potentiate dermal matrix damage; (3) use of lasers (e.g. pulsed dye, potassium titanyl phosphate) as well as intense pulsed light may improve symptoms.

a disruption in the blood vessels that supply the skin on the face. This leads to inflammation and redness of the skin, as well as the formation of small bumps or pustules.<br><br>Rosacea is often characterized by redness and flushing of the face, particularly in the central part of the face such as the nose, cheeks, and forehead. It can also cause small, red bumps or pustules to form on the skin. In some cases, the eyes may also be affected, appearing watery or bloodshot.<br><br>Although the exact cause of rosacea is not known, it is believed to be related to a combination of genetic and environmental factors. Certain triggers can cause the symptoms of rosacea to flare up, including sun exposure, stress, spicy foods, alcohol, and certain cosmetics.<br><br>It is important to note that while rosacea cannot be cured, it can be treated effectively with a combination of medications and lifestyle changes. Treatment options include topical creams and oral medications, as well as lifestyle changes to reduce triggers.<br><br>Q4. How should I treat it? What medicine should I use?<br><br>A4. There are several effective treatments for rosacea that can help to reduce the symptoms of redness, inflammation, and bumps on the skin. Treatment options may include a combination of topical creams, oral medications, and lifestyle changes.<br><br>Topical creams and gels: These treatments are applied directly to the skin and are used to reduce redness and inflammation. They may contain ingredients such as metronidazole, azelaic acid, or brimonidine. These creams may be prescribed by a dermatologist or obtained over the counter.<br><br>Oral medications: These medications are taken by mouth and are used to reduce inflammation and redness. They may be prescribed by a dermatologist and can include antibiotics such as doxycycline or minocycline, or anti-inflammatory medications such as sulfasalazine.<br><br>Lifestyle changes: Avoiding triggers such as spicy	

foods, alcohol, and certain cosmetics can help to reduce the severity of rosacea symptoms. It is also important to avoid excessive sun exposure and to use sunscreen with a high SPF to protect the skin.	
Evaluation of Physician	
Evaluation Item	Score (1-5, the higher the better)
SkinGPT-4's diagnosis is correct or relevant.	5
SkinGPT-4's description is informative.	4
SkinGPT-4's suggestions are useful.	5
SkinGPT-4 can help doctors with diagnosis.	5
SkinGPT-4 can help patients to understand their disease better.	5
If SkinGPT-4 can be deployed locally, it protects patients' privacy.	5
Willingness to use SkinGPT-4	5

Case Rosacea 5

Image ID: rosacea5	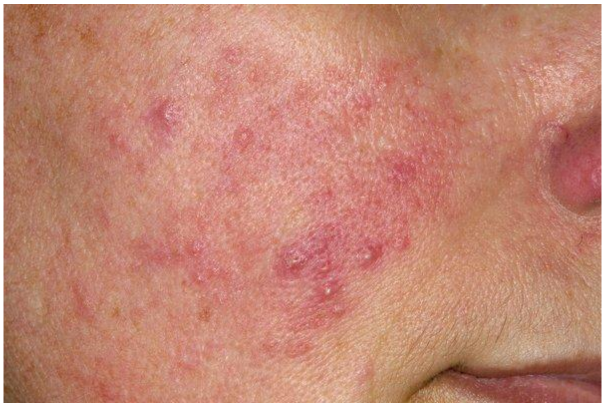
SkinGPT-4	Dermatology Physician
Q1. Could you describe the skin disease in this image for me?<br><br>A1. This could be a skin disease image of rosacea.<br><br>Q2. You should write a paragraph to list more features you observed in the image.<br><br>A2. This image could depict rosacea, a skin condition characterized by flushing, persistent redness, and inflammatory lesions, such as papules and pustules. Additionally, the skin may appear thin and easily damaged, with a tendency to bruise. The eyes may also demonstrate an ocular rosacea, with a red, watery eye and frequent stinging or burning sensations.<br><br>Q3. With previous information, please write a paragraph to explain in detail the cause of this skin disease.<br><br>A3. Rosacea is a chronic skin condition that affects the face and sometimes the neck, chest, and ears. The exact cause is unknown, but it is believed to be related to blood vessels, nerves, and immune system dysfunction. Triggers that can worsen rosacea include sun exposure, heat, alcohol, spicy foods, stress, and hormonal changes. The skin may become red, inflamed, and covered in bumps or pustules, which can cause embarrassment and low self-esteem. However, there is no cure for rosacea, and treatment is focused on managing symptoms and	Q1. Could you describe the skin disease in this image for me?<br><br>A1. Rosacea or Acne.<br><br>Q2. You should write a paragraph to list more features you observed in the image.<br><br>A2. Erythema, papules and pustules on the face.<br><br>Q3. With previous information, please write a paragraph to explain in detail the cause of this skin disease.<br><br>A3. The exact pathogenesis of rosacea is unknown. The disorder which is known today as rosacea will probably be shown in time to comprise several similar, possibly related, but different clinical conditions, each with separate predominant pathogenic mechanisms. Aberrant innate immune system, epidermal barrier dysfunction, Vascular changes, neurogenic inflammation, ultraviolet radiation and microbes may involved.<br><br>Q4. How should I treat it? What medicine should I use?<br><br>A4. In addition to basic treatment, the use of topical antibacterial drugs such as Metronidazole, Azeleic acid and Clindamycin may be effect. By the way, oral antibacterial drugs should be used.

preventing flare-ups. Regular skin care, avoidance of triggers, and prescription medications, such as antibiotics and topical creams, may be necessary to manage the condition.<br><br>Q4. How should I treat it? What medicine should I use?<br><br>A4. Rosacea treatment aims to manage symptoms and prevent flare-ups. The following can be used to treat rosacea:<br>* Avoiding triggers: Identify and avoid factors that worsen rosacea, such as sun exposure, heat, alcohol, spicy foods, stress, and hormonal changes.<br>* Basic skin care: Keep the skin clean and moisturized with a mild, non-irritating cleanser and moisturizer. Avoid harsh products and scrubbing the skin.<br>* Prescription medication: Antibiotics (such as doxycycline, minocycline, or azithromycin) and topical creams (such as metronidazole or brimonidine) may be prescribed to reduce inflammation and bacteria on the skin.<br>* Oral medication: For severe cases, oral antibiotics or isotretinoin may be prescribed.<br>* Laser and light therapy: Intense pulsed light (IPL) or pulsed dye laser (PDL) may be used to reduce inflammation and improve appearance.<br>It is important to consult a dermatologist for an accurate diagnosis and appropriate treatment plan, as the severity and presentation of rosacea can vary greatly.	
Evaluation of Physician	
Evaluation Item	Score (1-5, the higher the better)
SkinGPT-4's diagnosis is correct or relevant.	5
SkinGPT-4's description is informative.	5
SkinGPT-4's suggestions are useful.	5
SkinGPT-4 can help doctors with diagnosis.	5
SkinGPT-4 can help patients to understand their disease better.	5

If SkinGPT-4 can be deployed locally, it protects patients' privacy.	5
Willingness to use SkinGPT-4	5

Case Rosacea 6

Image ID: rosacea6	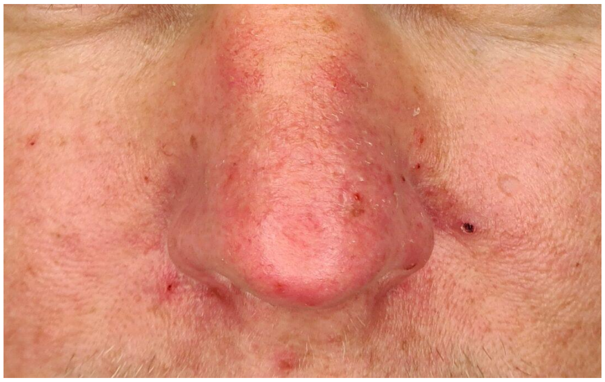
**SkinGPT-4**	**Dermatology Physician**
Q1. Could you describe the skin disease in this image for me?<br><br>A1. acne rosacea<br><br>Q2. You should write a paragraph to list more features you observed in the image.<br><br>A2. In the image provided, the man has a round face with a slightly pointed chin. His nose is straight and has a slightly bulbous tip. The nostrils are wide and flared. The skin on his face appears to be relatively smooth and without significant wrinkles or sun damage. The man has a moustache and a beard, which appear to be well-groomed. The hair on his head is thinning on top, with a receding hairline. The eyes are deep-set and the eyelids appear to be relatively puffy. The lips are thin and have a slightly downturned mouth corner. The man has a fair complexion with some areas of redness and inflammation on his cheeks and around his nose. Overall, the image suggests that this man may be experiencing some skin issues, such as rosacea or sensitive skin, but otherwise appears to be in good health.<br><br>Q3. With previous information, please write a paragraph to explain in detail the cause of this skin disease.<br><br>A3. The cause of the skin disease depicted in the provided image is not clear. However, based on the visible symptoms, it is possible that the individual is experiencing some form of skin	Q1. Could you describe the skin disease in this image for me?<br><br>A1. Rosacea<br><br>Q2. You should write a paragraph to list more features you observed in the image.<br><br>A2. Erythema, papules and small facial eruptions (especially on the nose).<br><br>Q3. With previous information, please write a paragraph to explain in detail the cause of this skin disease.<br><br>A3. The exact pathogenesis of rosacea is unknown. The disorder which is known today as rosacea will probably be shown in time to comprise several similar, possibly related, but different clinical conditions, each with separate predominant pathogenic mechanisms. Aberrant innate immune system, epidermal barrier dysfunction, Vascular changes, neurogenic inflammation, ultraviolet radiation and microbes may involved.<br><br>Q4. How should I treat it? What medicine should I use?<br><br>A4. In addition to basic treatment, topical epidermal growth factor is needed.

inflammation or irritation. Rosacea is a common skin condition that causes redness, inflammation, and visible blood vessels on the face, and this may be the cause of the redness and flushing seen in the image. Alternatively, the individual may be experiencing a reaction to certain substances, such as irritants or allergens, which can cause redness, inflammation, and other skin symptoms. It is also possible that the individual has a genetic predisposition to skin conditions, such as sensitivity or allergies, which could contribute to the development of this skin disease. In order to determine the exact cause of this skin disease, a dermatologist would need to conduct a thorough examination of the individual's skin and medical history.<br><br>Q4. How should I treat it? What medicine should I use?<br><br>A4. The treatment for this skin disease depends on the specific condition that is causing the symptoms. If the individual is experiencing rosacea, treatment options may include topical creams or oral medications to reduce inflammation and redness, as well as lifestyle modifications to avoid triggers that can exacerbate symptoms. If the skin disease is caused by an allergen or irritant, the best course of treatment would be to avoid exposure to the trigger and to use over-the-counter antihistamines or hydrocortisone cream to reduce inflammation and redness. If the individual has a genetic predisposition to skin conditions, a dermatologist may recommend a topical treatment or prescribe oral medication to manage symptoms.<br>It's important to note that self-medication can be harmful, so it is recommended to consult a dermatologist or a medical professional before using any medication. The dermatologist will provide you with the most appropriate treatment based on the diagnosis and the severity of the disease.	
Evaluation of Physician	
Evaluation Item	Score (1-5, the higher the better)
SkinGPT-4's diagnosis is correct or relevant.	5

SkinGPT-4's description is informative.	4
SkinGPT-4's suggestions are useful.	5
SkinGPT-4 can help doctors with diagnosis.	5
SkinGPT-4 can help patients to understand their disease better.	5
If SkinGPT-4 can be deployed locally, it protects patients' privacy.	5
Willingness to use SkinGPT-4	5

Case Rosacea 7

Image ID: rosacea7	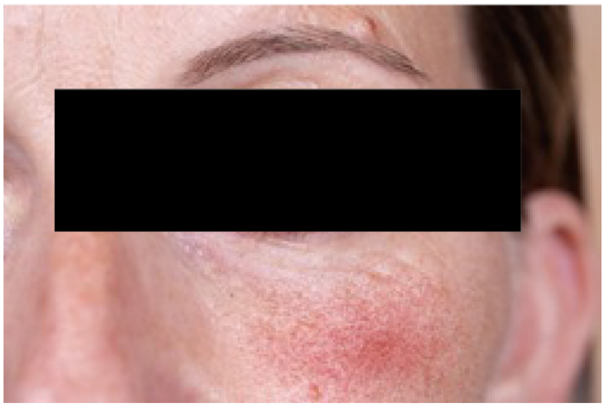
SkinGPT-4	Dermatology Physician
Q1. Could you describe the skin disease in this image for me?<br><br>A1. acne and rosacea and perioral dermatitis<br><br>Q2. You should write a paragraph to list more features you observed in the image.<br><br>A2. The image shows a woman with a clear complexion and well - defined features. Her face is oval - shaped with a slightly pointed chin, and her eyes are almond - shaped with a slight tilt upwards at the outer corners. Her eyebrows are thick and straight, and her eyes are a deep brown color with a faint ring of lashes.<br><br>Q3. With previous information, please write a paragraph to explain in detail the cause of this skin disease.<br><br>A3. The exact cause of the woman's skin condition is not specified in the provided image, but there are several potential factors that could be contributing to her healthy appearance. Good skin care, such as regular cleaning, moisturizing, and protection from the sun, can help to maintain a healthy appearance and prevent the development of skin problems. A balanced diet with a variety of fruits, vegetables, and healthy fats can also support skin health, as many of these foods contain vitamins and minerals that are important for skin function. Additionally, genetics and lifestyle factors, such as stress and exposure to environmental toxins, can also play a role in skin health. If the woman is experiencing any skin	Q1. Could you describe the skin disease in this image for me?<br><br>A1. Rosacea.<br><br>Q2. You should write a paragraph to list more features you observed in the image.<br><br>A2. Erythema on the face (except around the eyes).<br><br>Q3. With previous information, please write a paragraph to explain in detail the cause of this skin disease.<br><br>A3. The exact pathogenesis of rosacea is unknown. The disorder which is known today as rosacea will probably be shown in time to comprise several similar, possibly related, but different clinical conditions, each with separate predominant pathogenic mechanisms. Aberrant innate immune system, epidermal barrier dysfunction, Vascular changes, neurogenic inflammation, ultraviolet radiation and microbes may involved.<br><br>Q4. How should I treat it? What medicine should I use?<br><br>A4. Basic skin care is needed: Keep the skin clean and moisturized with a mild, non-irritating cleanser and moisturizer. Avoid harsh products and scrubbing the skin.<br>Avoiding triggers are essential: Identify and avoid factors that worsen rosacea, such as sun

issues, it is important for her to consult with a dermatologist to determine the cause and appropriate treatment.<br><br>Q4. How should I treat it? What medicine should I use?<br><br>A4. The best treatment for a skin condition will depend on the specific type of condition and its severity. For some minor skin issues, over - the - counter medications or home remedies may be sufficient. However, for more serious conditions, a dermatologist should be consulted to determine the appropriate treatment.<br>In general, treatment for skin conditions may include topical medications, such as creams or ointments, which are applied directly to the skin. Oral medications, such as antibiotics or retinoids, may also be prescribed. Phototherapy, which involves exposure to specialized light, may also be used to treat some conditions. In some cases, surgical procedures may be necessary to remove or treat skin lesions.<br>It is important to follow the recommended treatment plan and to use any medications as directed by a healthcare provider. In addition, maintaining good skin care habits, such as regularly cleaning and moisturizing the skin, can help to support skin health and prevent the development of new issues.	exposure, heat, alcohol, spicy foods, stress.
Evaluation of Physician	
Evaluation Item	Score (1-5, the higher the better)
SkinGPT-4's diagnosis is correct or relevant.	5
SkinGPT-4's description is informative.	1
SkinGPT-4's suggestions are useful.	2
SkinGPT-4 can help doctors with diagnosis.	3
SkinGPT-4 can help patients to understand their disease better.	3
If SkinGPT-4 can be deployed locally, it protects patients' privacy.	5

Willingness to use SkinGPT-4	2

Case Rosacea 8

<table>
<tr><td>Image ID: rosacea8</td><td>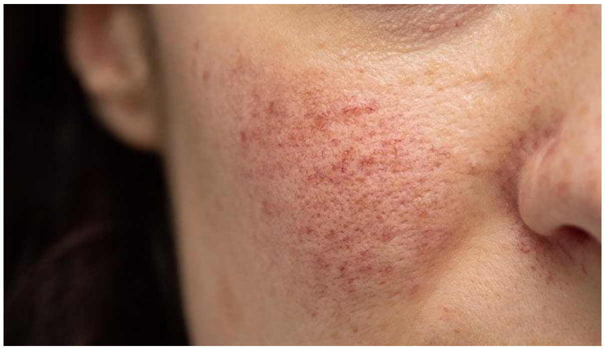</td></tr>
<tr><th>SkinGPT-4</th><th>Dermatology Physician</th></tr>
<tr><td>Q1. Could you describe the skin disease in this image for me?

A1. This could be a skin disease image of rosacea.

Q2. You should write a paragraph to list more features you observed in the image.

A2. Flushing, erythema, papules, pustules, and telangiectasias.

Q3. With previous information, please write a paragraph to explain in detail the cause of this skin disease.

A3. This appears to be a skin disease image of rosacea. The cause of rosacea is not fully understood, but it is believed to involve blood vessel dilation, inflammation, and oxidative stress. Triggers include sun exposure, heat, stress, spicy foods, alcohol, and certain cosmetics. Treatment options include topical metronidazole, oral antibiotics, and laser and light therapy. Lifestyle modifications such as avoiding triggers, using gentle skin care products, and protecting the skin from the sun can also be helpful.

Q4. How should I treat it? What medicine should I use?

A4. Treatment options for rosacea include topical metronidazole, oral antibiotics, and laser and light therapy. Lifestyle modifications such as avoiding triggers, using gentle skin care products, and</td><td>Q1. Could you describe the skin disease in this image for me?

A1. Rosacea

Q2. You should write a paragraph to list more features you observed in the image.

A2. Purplish blotches on the face (except around the eyes).

Q3. With previous information, please write a paragraph to explain in detail the cause of this skin disease.

A3. The exact pathogenesis of rosacea is unknown. The disorder which is known today as rosacea will probably be shown in time to comprise several similar, possibly related, but different clinical conditions, each with separate predominant pathogenic mechanisms. Aberrant innate immune system, epidermal barrier dysfunction, Vascular changes, neurogenic inflammation, ultraviolet radiation and microbes may involved.

Q4. How should I treat it? What medicine should I use?

A4. Basic skin care is needed: Keep the skin clean and moisturized with a mild, non-irritating cleanser and moisturizer. Avoid harsh products and scrubbing the skin.
Avoiding triggers are essential: Identify and avoid factors that worsen rosacea, such as sun</td></tr>
</table>

protecting the skin from the sun can also be helpful. It is best to consult a dermatologist for a proper diagnosis and treatment plan.	exposure, heat, alcohol, spicy foods and stress.
Evaluation of Physician	
Evaluation Item	Score (1-5, the higher the better)
SkinGPT-4's diagnosis is correct or relevant.	5
SkinGPT-4's description is informative.	5
SkinGPT-4's suggestions are useful.	5
SkinGPT-4 can help doctors with diagnosis.	5
SkinGPT-4 can help patients to understand their disease better.	5
If SkinGPT-4 can be deployed locally, it protects patients' privacy.	5
Willingness to use SkinGPT-4	5

Case Rosacea 9

Image ID: rosacea9	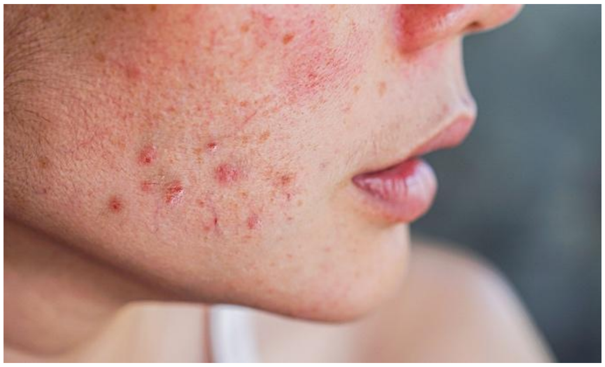
**SkinGPT-4**	**Dermatology Physician**
Q1. Could you describe the skin disease in this image for me?<br><br>A1. acne<br><br>Q2. You should write a paragraph to list more features you observed in the image.<br><br>A2. This image shows a person with acne on their face. The acne is mainly found on the face and it is red and inflamed. The acne lesions are small, red and have whiteheads or blackheads on them. The skin looks oily and shiny. The person looks concerned and their face is covered with acne. They may have tried different treatments to get rid of it, such as topical medications and over the counter creams and ointments. They may also be seeking medical advice from a dermatologist. The condition is chronic and persistent, and it can affect self esteem, social interactions and mental health. It is important to treat acne to prevent scarring, and to improve the appearance of the skin.<br><br>The acne is red and inflamed, it can be seen on the nose, chin and forehead. The skin looks oily and shiny. It is important to treat acne to prevent scarring, and to improve the appearance of the skin. Acne is a chronic and persistent condition that can affect self-esteem, social interactions and mental health. The condition can be caused by various factors such as genetics, hormones, stress, and diet.<br><br>Q3. With previous information, please write a	Q1. Could you describe the skin disease in this image for me?<br><br>A1. Acne or Rosacea.<br><br>Q2. You should write a paragraph to list more features you observed in the image.<br><br>A2. Erythema, papules and small facial eruptions (perhaps also on the nose).<br><br>Q3. With previous information, please write a paragraph to explain in detail the cause of this skin disease.<br><br>A3. The exact pathogenesis of rosacea is unknown. The disorder which is known today as rosacea will probably be shown in time to comprise several similar, possibly related, but different clinical conditions, each with separate predominant pathogenic mechanisms. Aberrant innate immune system, epidermal barrier dysfunction, Vascular changes, neurogenic inflammation, ultraviolet radiation and microbes may involved.<br><br>Q4. How should I treat it? What medicine should I use?<br><br>A4. In addition to basic treatment, topical antibacterial drugs such as Metronidazole, Azeleic acid and Clindamycin may be effect. By the way, oral antibacterial drugs should be used.

paragraph to explain in detail the cause of this skin disease.<br><br>A3. Acne is primarily caused by a combination of genetics, hormones, and other factors such as stress, diet, and certain medications.<br><br>Genetics: Acne is often hereditary and can run in families. People with a family history of acne are more likely to develop the condition themselves.<br><br>Hormones: Hormonal imbalances, particularly androgens, can cause acne. Androgens are male hormones that are present in both men and women. They stimulate the production of sebum, an oil that is produced by the skin. When the sebum glands become overactive, it can clog pores and lead to acne.<br><br>Stress: Stress can cause an increase in androgens, which can lead to acne. Stress can also cause an increase in inflammation, which can make acne worse.<br><br>Diet: Eating a diet high in refined carbohydrates and saturated fats can lead to an increase in insulin levels, which can cause an increase in androgens and lead to acne.<br><br>Medications: Certain medications such as testosterone, corticosteroids, and lithium can cause acne as a side effect.<br><br>Q4. How should I treat it? What medicine should I use?<br><br>A4. Acne can be treated with a variety of methods, including:<br><br>1. Over-the-counter treatments: These include topical medications such as benzoyl peroxide, salicylic acid, and alpha-hydroxy acid, which help to unclog pores and reduce inflammation.<br>2. Prescription medications: These include oral antibiotics, such as doxycycline and minocycline, which help to reduce inflammation and fight bacteria that can contribute to acne.<br>3. Hormonal therapy: For women, birth control pills can help regulate hormones that contribute to acne. For men, medications that reduce testosterone levels can help decrease acne.<br>4. Light therapy: This therapy uses a special lamp	

to emit light that kills bacteria and reduces inflammation.<br>5. Chemical peels: This treatment involves applying a chemical solution to the skin to remove the top layers and stimulate new skin growth.<br>6. Laser therapy: This therapy uses a laser to target the sebaceous glands and reduce the production of oil.<br><br>It's important to note that what works for one person may not work for another, so it's best to speak to a dermatologist to determine the best treatment plan for your specific case.	
Evaluation of Physician	
Evaluation Item	Score (1-5, the higher the better)
SkinGPT-4's diagnosis is correct or relevant.	5
SkinGPT-4's description is informative.	4
SkinGPT-4's suggestions are useful.	4
SkinGPT-4 can help doctors with diagnosis.	4
SkinGPT-4 can help patients to understand their disease better.	4
If SkinGPT-4 can be deployed locally, it protects patients' privacy.	5
Willingness to use SkinGPT-4	4

Case Rosacea 10

Image ID: rosacea10	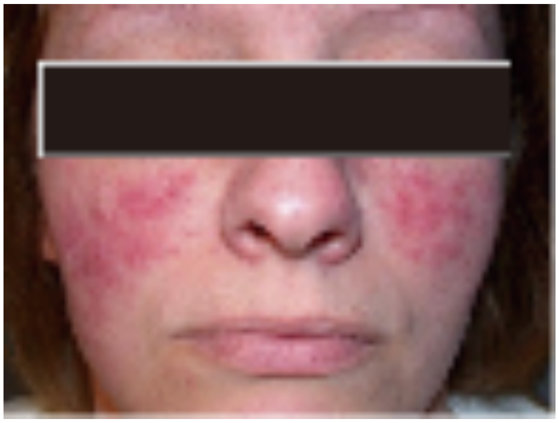
**SkinGPT-4**	**Dermatology Physician**
Q1. Could you describe the skin disease in this image for me?<br><br>A1. This could be a skin disease image of rosacea.<br><br>Q2. You should write a paragraph to list more features you observed in the image.<br><br>A2. This image appears to show a chronic skin condition known as rosacea. The rash consists of small, red, solid bumps that tend to affect the forehead, nose, cheeks, and chin. The center of the rash may have a white center and be more raised than the surrounding skin. The rash may worsen with sun exposure or stress and may be accompanied by flare ups of burning, stinging, or itching. The rash is usually not itchy but may cause embarrassment and social discomfort.<br><br>Q3. With previous information, please write a paragraph to explain in detail the cause of this skin disease.<br><br>A3. This image appears to depict a case of rosacea, which is a chronic skin disorder characterized by flushing, blushing, and redness of the face, often accompanied by acne-like pimples, bumps, and spider veins. The cause of rosacea is not well understood, but it is thought to be related to a combination of genetic, environmental, and inflammatory factors. Triggers for rosacea include sun exposure, heat, alcohol,	Q1. Could you describe the skin disease in this image for me?<br><br>A1. Rosacea<br><br>Q2. You should write a paragraph to list more features you observed in the image.<br><br>A2. Erythema and red papules on the cheeks and nose (except around the eyes).<br><br>Q3. With previous information, please write a paragraph to explain in detail the cause of this skin disease.<br><br>A3. The exact pathogenesis of rosacea is unknown. The disorder which is known today as rosacea will probably be shown in time to comprise several similar, possibly related, but different clinical conditions, each with separate predominant pathogenic mechanisms. Aberrant innate immune system, epidermal barrier dysfunction, Vascular changes, neurogenic inflammation, ultraviolet radiation and microbes may involved.<br><br>Q4. How should I treat it? What medicine should I use?<br><br>A4. At this condition, topical antibacterial drugs such as Metronidazole, Azeleic acid and Clindamycin may be effect. By the way, oral antibacterial drugs should be used at the same

spicy foods, and emotional stress. The disorder tends to affect people with fair skin, and it can worsen over time if left untreated. Treatment options include topical creams, oral antibiotics, and laser therapy.<br><br>Q4. How should I treat it? What medicine should I use?<br><br>A4. Treatment of rosacea typically involves a combination of topical therapy, lifestyle modifications, and oral medications. Topical therapy may include metronidazole or azelaic acid to reduce inflammation and redness, while oral antibiotics such as doxycycline, minocycline, or brimonidine may be prescribed to reduce inflammation and bacterial load. Systemic isotretinoin may also be considered for severe cases that do not respond to other treatments. It is important to follow the treatment plan and instructions provided by a dermatologist for optimal results.	time.
Evaluation of Physician	
Evaluation Item	Score (1-5, the higher the better)
SkinGPT-4's diagnosis is correct or relevant.	5
SkinGPT-4's description is informative.	1
SkinGPT-4's suggestions are useful.	4
SkinGPT-4 can help doctors with diagnosis.	3
SkinGPT-4 can help patients to understand their disease better.	3
If SkinGPT-4 can be deployed locally, it protects patients' privacy.	5
Willingness to use SkinGPT-4	3

Case Actinic Keratosis 1

Image ID: Actinic Keratosis1	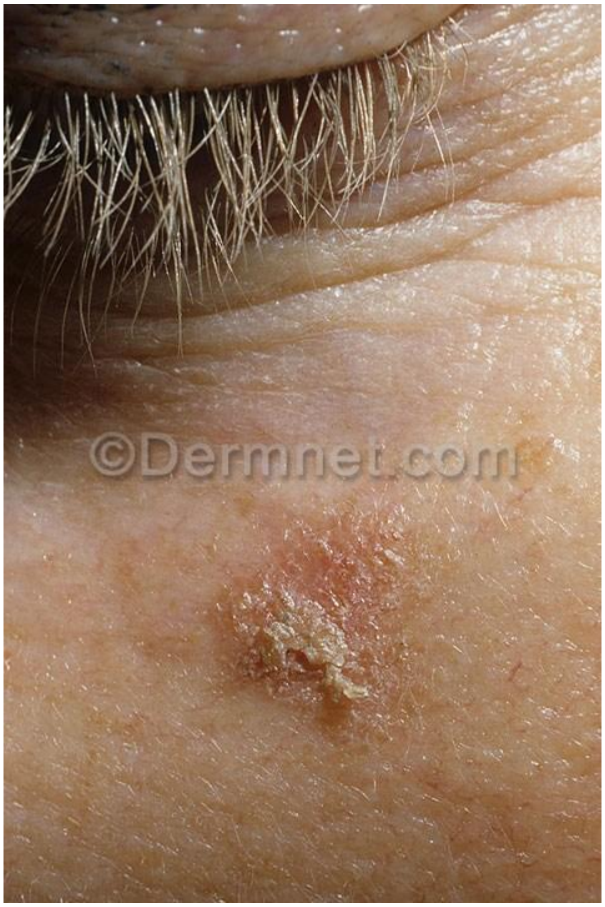
**SkinGPT-4**	**Dermatology Physician**
Q1. Could you describe the skin disease in this image for me?<br><br>A1. This could be a skin disease image of actinic keratosis face.<br><br>Q2. You should write a paragraph to list more features you observed in the image.<br><br>A2. This image shows a scaly lesion on the face, which is a common sign of actinic keratosis. The lesion appears as a dry, rough, and brownish-grey scale on the sun-exposed areas of the skin, such as the face, hands, and forearms. Actinic keratosis is a precancerous condition caused by chronic sun exposure and can progress to squamous cell carcinoma if not treated.<br><br>Q3. With previous information, please write a	Q1. Could you describe the skin disease in this image for me?<br><br>A1. Actinic keratosis (AK).<br><br>Q2. You should write a paragraph to list more features you observed in the image.<br><br>A2. A rough erythematous papule with white to yellow scale.<br><br>Q3. With previous information, please write a paragraph to explain in detail the cause of this skin disease.<br><br>A3. AKs are amongst the most frequently encountered skin lesions in clinical practice. They present on sun-damaged skin of the head, neck, upper trunk and extremities. Individuals at higher

paragraph to explain in detail the cause of this skin disease.<br><br>A3. Actinic keratosis is a precancerous condition caused by chronic sun exposure. It occurs when the skin is exposed to ultraviolet radiation over time, which damages the DNA of skin cells and causes mutations that can lead to the development of cancer. The damage can be caused by both UVA and UVB rays, but UVB rays are more harmful and responsible for most cases of actinic keratosis. The condition commonly occurs in people with fair skin, hair, and eyes, as they are more prone to sun damage. Actinic keratosis most commonly affects the skin on the face, lips, ears, hands, and forearms, areas that are frequently exposed to the sun. The risk of developing actinic keratosis can be reduced by avoiding excessive sun exposure, using sunscreen, and seeking regular skin examinations.<br><br>Q4. How should I treat it? What medicine should I use?<br><br>A4. Actinic keratosis can be treated with several options, including topical medications, cryotherapy, and surgery. The most common topical medications used to treat actinic keratosis are imiquimod, fluorouracil, and salicylic acid. These medications work by increasing cell turnover, reducing inflammation, and promoting wound healing. Cryotherapy involves freezing the lesion with liquid nitrogen, which causes the top layer of skin to blister and fall off, allowing new skin to form. Surgery is usually reserved for lesions that are thick, raised, or have changed color and are at risk of becoming cancerous. The most common surgical procedure for actinic keratosis is cryosurgery, which involves freezing the lesion with liquid nitrogen. It is essential to seek medical advice before starting any treatment for actinic keratosis to ensure the most appropriate and effective management.	risk of developing AKs include the elderly, those with lighter skin phototypes and those with a history of chronic sun exposure. AKs have historically been characterized as being “precancerous” or “premalignant” because the atypical keratinocytes within these lesions are confined to the epidermis. There is no risk of metastasis until these lesions evolve into invasive carcinoma.<br><br>Q4. How should I treat it? What medicine should I use?<br><br>A4. The first step is a dermoscopy for the exclusion of melanoma and other skin tumor.. The most common method of destruction is cryotherapy, laser vaporization or electrodesiccation.
Evaluation of Physician	
Evaluation Item	Score (1-5, the higher the better)
SkinGPT-4’s diagnosis is correct or relevant.	5

SkinGPT-4's description is informative.	5
SkinGPT-4's suggestions are useful.	5
SkinGPT-4 can help doctors with diagnosis.	5
SkinGPT-4 can help patients to understand their disease better.	5
If SkinGPT-4 can be deployed locally, it protects patients' privacy.	5
Willingness to use SkinGPT-4	5

Case Actinic Keratosis 2

Image ID: Actinic Keratosis2	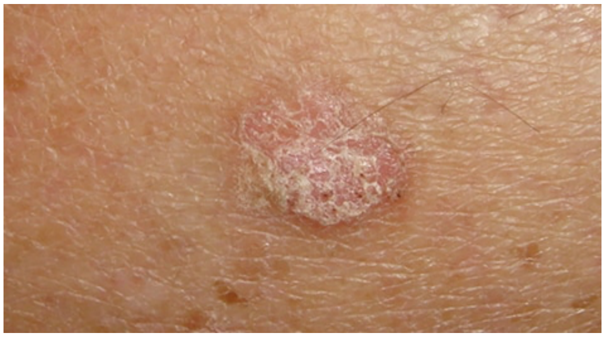
**SkinGPT-4**	**Dermatology Physician**
Q1. Could you describe the skin disease in this image for me?<br><br>A1. This could be a skin disease image of seborrheic keratosis irritated.<br><br>Q2. You should write a paragraph to list more features you observed in the image.<br><br>A2. There are many small lesions on the skin and they are rough in texture.<br><br>Q3. With previous information, please write a paragraph to explain in detail the cause of this skin disease.<br><br>A3. This is a condition caused by damage to the DNA from ultraviolet radiation from the sun or tanning beds.<br><br>Q4. How should I treat it? What medicine should I use?<br><br>A4. Treatment options include topical medications, cryotherapy, photodynamic therapy, and excision surgery.	Q1. Could you describe the skin disease in this image for me?<br><br>A1. Actinic keratosis (AK).<br><br>Q2. You should write a paragraph to list more features you observed in the image.<br><br>A2. A rough erythematous papule with white to yellow scale.<br><br>Q3. With previous information, please write a paragraph to explain in detail the cause of this skin disease.<br><br>A3. AKs are amongst the most frequently encountered skin lesions in clinical practice. They present on sun-damaged skin of the head, neck, upper trunk and extremities. Individuals at higher risk of developing AKs include the elderly, those with lighter skin phototypes and those with a history of chronic sun exposure. AKs have historically been characterized as being “precancerous” or “premalignant” because the atypical keratinocytes within these lesions are confined to the epidermis. There is no risk of metastasis until these lesions evolve into invasive carcinoma.<br><br>Q4. How should I treat it? What medicine should I use?<br><br>A4. The first step is a dermoscopy for the exclusion of melanoma and other skin tumor.. The most common method of destruction is cryotherapy, laser vaporization or electrodesiccation.

Evaluation of Physician	
Evaluation Item	Score (1-5, the higher the better)
SkinGPT-4's diagnosis is correct or relevant.	5
SkinGPT-4's description is informative.	4
SkinGPT-4's suggestions are useful.	5
SkinGPT-4 can help doctors with diagnosis.	5
SkinGPT-4 can help patients to understand their disease better.	5
If SkinGPT-4 can be deployed locally, it protects patients' privacy.	5
Willingness to use SkinGPT-4	5

Case Actinic Keratosis 3

Image ID: Actinic Keratosis3	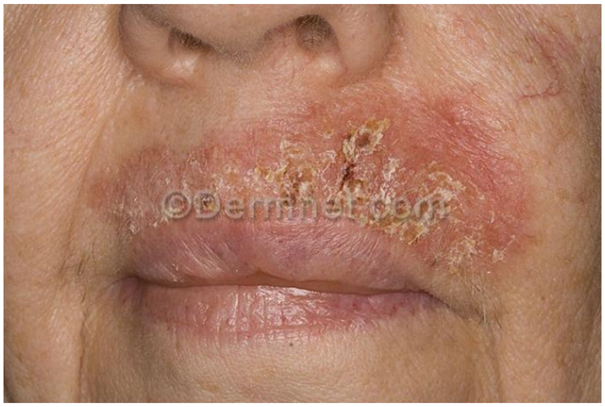
**SkinGPT-4**	**Dermatology Physician**
Q1. Could you describe the skin disease in this image for me?<br><br>A1. This could be a skin disease image of actinic keratosis<br><br>Q2. You should write a paragraph to list more features you observed in the image.<br><br>A2. In this image, the lesions appear as dry, rough, and scaly with a red base. The lesions are located on the cheeks, nose, and upper lip, with a distinct border and a white, dry, and crusted center. There is actinic keratosis, which is a precancerous condition caused by chronic sun exposure.<br><br>Q3. With previous information, please write a paragraph to explain in detail the cause of this skin disease.<br><br>A3. Actinic keratosis, also known as solar keratosis, is a precancerous condition caused by chronic sun exposure. It typically occurs in older adults and is more common in people with fair skin, blue eyes, and a history of sun exposure. The condition is caused by mutations in the skin's DNA due to chronic sun exposure, which leads to the development of abnormal skin cells. These abnormal cells can progress to squamous cell carcinoma, which is a more aggressive form of skin cancer. The condition is most commonly found on areas of the skin that are frequently exposed to the sun, such as the face, ears, neck, and lips. It can be treated with various methods,	Q1. Could you describe the skin disease in this image for me?<br><br>A1. Inflammatory Erythema; (AK?)<br><br>Q2. You should write a paragraph to list more features you observed in the image.<br><br>A2. Red spots on upper lip with white scales and crusts<br><br>Q3. With previous information, please write a paragraph to explain in detail the cause of this skin disease.<br><br>A3. AKs are amongst the most frequently encountered skin lesions in clinical practice. They present on sun-damaged skin of the head, neck, upper trunk and extremities. Individuals at higher risk of developing AKs include the elderly, those with lighter skin phototypes and those with a history of chronic sun exposure. AKs have historically been characterized as being "precancerous" or "premalignant" because the atypical keratinocytes within these lesions are confined to the epidermis. There is no risk of metastasis until these lesions evolve into invasive carcinoma.<br><br>Q4. How should I treat it? What medicine should I use?<br><br>A4. The first step is a dermoscopy for the exclusion of melanoma and other skin tumor. The most common method of destruction is

including cryotherapy, curettage, and topical medications. Actinic keratosis can be prevented by avoiding prolonged sun exposure, using sunscreen, and protective clothing. Regular skin examinations can also help in early detection and treatment of actinic keratosis before it becomes cancerous.<br><br>Q4. How should I treat it? What medicine should I use?<br><br>A4. Actinic keratosis can be treated with various methods, including cryotherapy, curettage, and topical medications.<br>Cryotherapy involves the use of liquid nitrogen to freeze and destroy the abnormal skin cells. This method is relatively painless and effective, but it may cause some temporary redness and swelling.<br>Curettage involves the use of a curette, which is a sharp-edged tool, to scrape away the abnormal skin cells. This method is also effective but may be more painful and may leave a scar.<br>Topical medications, such as fluorouracil, imiquimod, and salicylic acid, can also be used to treat actinic keratosis. These medications work by either killing the abnormal skin cells or removing them from the skin.<br>It is important to note that the treatment options may vary depending on the size, location, and severity of the lesions. It is best to consult a dermatologist for proper diagnosis and treatment. They will also be able to recommend the best medicine to use.	cryotherapy, laser vaporization or electrodesiccation. In this patient, a biopsy is required.
Evaluation of Physician	
Evaluation Item	Score (1-5, the higher the better)
SkinGPT-4's diagnosis is correct or relevant.	3
SkinGPT-4's description is informative.	4
SkinGPT-4's suggestions are useful.	4
SkinGPT-4 can help doctors with diagnosis.	5
SkinGPT-4 can help patients to understand their disease better.	5

If SkinGPT-4 can be deployed locally, it protects patients' privacy.	5
Willingness to use SkinGPT-4	5

Case Actinic Keratosis 4

Image ID: Actinic Keratosis4	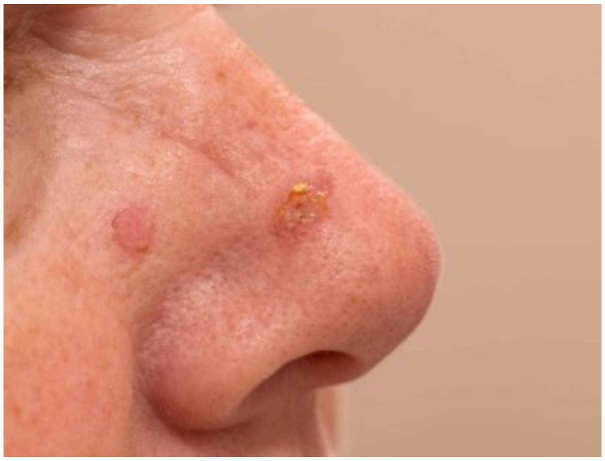
**SkinGPT-4**	**Dermatology Physician**
Q1. Could you describe the skin disease in this image for me?<br><br>A1. This could be a skin disease image of keratoacanthoma.<br><br>Q2. You should write a paragraph to list more features you observed in the image.<br><br>A2. The image provided appears to be a skin lesion, with a smooth, pearly surface and a sharply defined border. The lesion has a central depression or ulceration, which appears to be more pronounced at the center. The lesion appears to be indurate and has a yellowish hue. This could be a skin lesion of keratoacanthoma.<br><br>Q3. With previous information, please write a paragraph to explain in detail the cause of this skin disease.<br><br>A3. Keratoacanthoma is a benign skin tumor that arises from the squamous cells of the skin. It is characterized by a smooth, pearly surface and a sharply defined border. The central portion of the lesion is often ulcerated and may have a yellowish hue. Keratoacanthoma is often seen on sun exposed areas of the skin, particularly in older adults. It is believed to be caused by chronic sun exposure, which leads to mutations in the skin cells. The tumor can be removed by simple excision, and recurrences are rare. However, it is important to have a histopathological examination	Q1. Could you describe the skin disease in this image for me?<br><br>A1. Actinic Keratosis(?); Granuloma annulare(?)<br><br>Q2. You should write a paragraph to list more features you observed in the image.<br><br>A2. AKs are amongst the most frequently encountered skin lesions in clinical practice. They present on sun-damaged skin of the head, neck, upper trunk and extremities. Individuals at higher risk of developing AKs include the elderly, those with lighter skin phototypes and those with a history of chronic sun exposure. AKs have historically been characterized as being “precancerous” or “premalignant” because the atypical keratinocytes within these lesions are confined to the epidermis. There is no risk of metastasis until these lesions evolve into invasive carcinoma.<br><br>Q3. With previous information, please write a paragraph to explain in detail the cause of this skin disease.<br><br>A3. AKs are amongst the most frequently encountered skin lesions in clinical practice. They present on sun-damaged skin of the head, neck, upper trunk and extremities. Individuals at higher risk of developing AKs include the elderly, those with lighter skin phototypes and those with a history of chronic sun exposure. AKs have

of the removed tissue to ensure that it is not a squamous cell carcinoma.<br><br>Q4. How should I treat it? What medicine should I use?<br><br>A4. Keratoacanthoma is a benign skin tumor, and the treatment is usually surgical. The lesion can be removed by simple excision, which is usually done under local anesthesia. The surgical margins should be at least 4 mm for benign lesions and 6 mm for atypical or potentially malignant lesions. Recurrences are rare, but they can occur.<br>If the lesion is large or in a cosmetically sensitive area, a dermatologist may recommend a medical treatment before surgery to help shrink the lesion. This can include topical treatments such as imiquimod, 5-fluorouracil, or salicylic acid, or intralesional injections of bleomycin or imiquimod. However, it is important to note that these medical treatments may not be as effective as surgical excision and may have side effects.<br>After the surgery, it is important to follow proper wound care and apply a dressing to the wound to prevent infection and promote healing. A follow-up appointment with a dermatologist is usually scheduled to ensure that the wound is healing properly and to remove any sutures.<br>If the lesion is diagnosed as squamous cell carcinoma, further treatment such as radiation therapy or chemotherapy may be necessary.	historically been characterized as being "precancerous" or "premalignant" because the atypical keratinocytes within these lesions are confined to the epidermis. There is no risk of metastasis until these lesions evolve into invasive carcinoma.<br><br>Q4. How should I treat it? What medicine should I use?<br><br>A4. The first step is a dermoscopy for the exclusion of melanoma and other skin tumor. The most common method of destruction is cryotherapy, laser vaporization or electrodesiccation.
Evaluation of Physician	
Evaluation Item	Score (1-5, the higher the better)
SkinGPT-4's diagnosis is correct or relevant.	1
SkinGPT-4's description is informative.	1
SkinGPT-4's suggestions are useful.	1
SkinGPT-4 can help doctors with diagnosis.	1
SkinGPT-4 can help patients to understand their disease better.	1
If SkinGPT-4 can be deployed locally, it protects patients' privacy.	5

Willingness to use SkinGPT-4	1

Case Actinic Keratosis 5

Image ID: Actinic Keratosis5	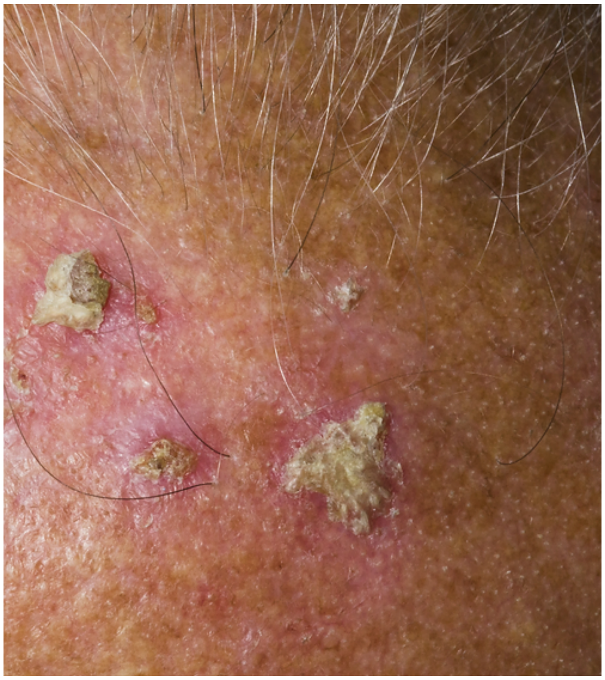
SkinGPT-4	Dermatology Physician
Q1. Could you describe the skin disease in this image for me?<br><br>A1. actinic keratosis and cell carcinoma and malignant lesions<br><br>Q2. You should write a paragraph to list more features you observed in the image.<br><br>A2. The image shows a mild to moderate actinic keratosis, a common precancerous skin lesion consisting of abnormal and dry, scaly or crusty patches on the skin, often found on the face, neck, and hands. It is caused by excessive sun exposure and can develop into squamous cell carcinoma, a malignant skin cancer. The image also shows several small, white, raised bumps, which could be seborrheic keratoses, benign growths that develop on the skin as people age.<br><br>Q3. With previous information, please write a paragraph to explain in detail the cause of this skin disease.<br><br>A3. The skin disease in the image is likely caused by excessive sun exposure, which can lead to the	Q1. Could you describe the skin disease in this image for me?<br><br>A1. Actinic keratosis<br><br>Q2. You should write a paragraph to list more features you observed in the image.<br><br>A2. The papules/plaques with scale or scale-crust and an erythematous base.<br><br>Q3. With previous information, please write a paragraph to explain in detail the cause of this skin disease.<br><br>A3. AKs are amongst the most frequently encountered skin lesions in clinical practice. They present on sun-damaged skin of the head, neck, upper trunk and extremities. Individuals at higher risk of developing AKs include the elderly, those with lighter skin phototypes and those with a history of chronic sun exposure. AKs have historically been characterized as being “precancerous” or “premalignant” because the atypical keratinocytes within these lesions are confined to the epidermis. There is no risk of

development of actinic keratosis. This condition occurs when the skin is exposed to ultraviolet (UV) radiation from the sun, which damages the skin cells and causes them to become abnormal and scaly. The image also shows signs of aging, such as the presence of seborrheic keratoses, which are benign growths that develop on the skin as people age. These growths are caused by a combination of genetics and environmental factors, including sun exposure. In some cases, actinic keratosis can develop into squamous cell carcinoma, a malignant skin cancer, so it is important to have any suspicious lesions checked by a dermatologist.<br><br>Q4. How should I treat it? What medicine should I use?<br><br>A4. Treatment for actinic keratosis and other skin conditions can vary depending on the severity and type of lesion. For mild to moderate actinic keratosis, topical treatments such as creams or gels containing salicylic acid, fluorouracil, or imiquimod can be used to remove the abnormal skin cells and reduce scaling and redness. These treatments can be purchased over the counter or prescribed by a dermatologist.<br><br>In some cases, a dermatologist may recommend a more intense treatment such as cryotherapy, which uses liquid nitrogen to freeze and remove the abnormal skin cells, or curettage, which involves scraping away the lesion with a currette.<br><br>If the actinic keratosis is more severe or has developed into squamous cell carcinoma, a doctor may recommend more aggressive treatment such as surgery, radiation therapy, or chemotherapy.<br><br>It is always recommended to consult a dermatologist for proper diagnosis and treatment. They will be able to determine the best course of treatment based on the severity and type of the lesion.	metastasis until these lesions evolve into invasive carcinoma.<br><br>Q4. How should I treat it? What medicine should I use?<br><br>A4. The first step is a dermoscopy for the exclusion of melanoma and other skin tumor. The most common method of destruction is cryotherapy, laser vaporization or electrodesiccation.
Evaluation of Physician	
Evaluation Item	Score (1-5, the higher the better)

SkinGPT-4's diagnosis is correct or relevant.	5
SkinGPT-4's description is informative.	5
SkinGPT-4's suggestions are useful.	5
SkinGPT-4 can help doctors with diagnosis.	5
SkinGPT-4 can help patients to understand their disease better.	5
If SkinGPT-4 can be deployed locally, it protects patients' privacy.	5
Willingness to use SkinGPT-4	5

Case Basal Cell Carcinoma 1

Image ID: Basal Cell Carcinoma1	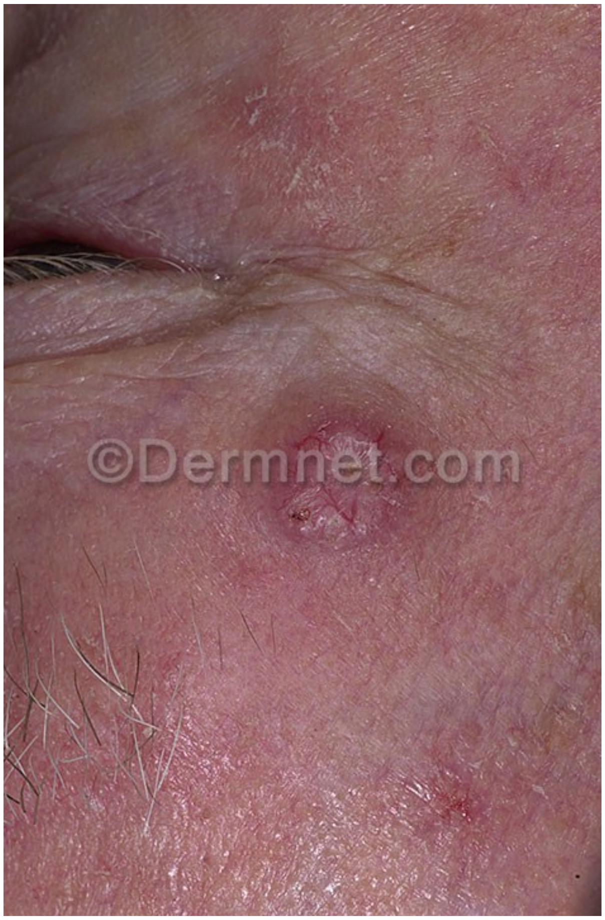
SkinGPT-4	Dermatology Physician
Q1. Could you describe the skin disease in this image for me?<br><br>A1. This could be a skin disease image of basal cell carcinoma.<br><br>Q2. You should write a paragraph to list more features you observed in the image.<br><br>A2. This could be a skin disease image of basal cell carcinoma face. The lesion appears pearly or shiny, with a clearly defined border and a scaly base. It is located on the face, which is a common site for basal cell carcinoma. This type of skin cancer is caused by prolonged sun exposure and can be prevented by using sunscreens and avoiding prolonged sun exposure. It is important	Q1. Could you describe the skin disease in this image for me?<br><br>A1. Tumor, basal cell carcinoma(?)<br><br>Q2. You should write a paragraph to list more features you observed in the image.<br><br>A2. A papules with dendritic dilatation of the blood vessels.<br><br>Q3. With previous information, please write a paragraph to explain in detail the cause of this skin disease.<br><br>A3. BCC is a tumor that arises within sun-damaged skin. It rarely develops on the palms and soles or mucous membranes.

to have regular skin exams to detect any suspicious lesions early.<br><br>Q3. With previous information, please write a paragraph to explain in detail the cause of this skin disease.<br><br>A3. Basal cell carcinoma (BCC) is a type of skin cancer that is caused by prolonged exposure to ultraviolet (UV) radiation from the sun. It typically affects areas of the skin that are frequently exposed to the sun, such as the face, neck, and ears. BCC grows slowly and can be mistaken for a benign skin growth or a pimple. It can be prevented by using sunscreens, wearing protective clothing, and avoiding prolonged sun exposure. The lesion in the image is likely to be a basal cell carcinoma because of its pearly or shiny appearance, clearly defined border, and scaly base. It is important to have regular skin exams to detect any suspicious lesions early, as BCC can be treated effectively if caught in its early stages.<br><br>Q4. How should I treat it? What medicine should I use?<br><br>A4. Treatment for basal cell carcinoma (BCC) depends on the size, location, and extent of the lesion. Surgical excision is the most common treatment for BCC, and cure rates are high if the cancer is detected and treated early. Other treatment options include radiation therapy, curettage, and cryotherapy. In some cases, a combination of these treatments may be used. Medications that can be used to treat BCC include:<br>-5-fluorouracil (5-FU)<br>-Imiquimod<br>-Sorafenib<br>-Vismodegib<br>It is important to note that BCC does not typically require chemotherapy. The choice of treatment depends on the location, size, and severity of the cancer, as well as the patient's general health and preferences. It is recommended to consult a dermatologist or a skin cancer specialist for the best treatment options.	Q4. How should I treat it? What medicine should I use?<br><br>A4. Standard surgical excision is effective for most primary BCCs. Mohs surgery is the preferred treatment for morpheaform, recurrent, poorly demarcated, highrisk, or incompletely removed BCC, and for those sites in which tissue conservation is paramount and there is a need for reliable clear margins.
Evaluation of Physician	
Evaluation Item	Score (1-5, the higher the better)

SkinGPT-4's diagnosis is correct or relevant.	5
SkinGPT-4's description is informative.	5
SkinGPT-4's suggestions are useful.	5
SkinGPT-4 can help doctors with diagnosis.	5
SkinGPT-4 can help patients to understand their disease better.	5
If SkinGPT-4 can be deployed locally, it protects patients' privacy.	5
Willingness to use SkinGPT-4	5

Case Basal Cell Carcinoma 2

Image ID: Basal Cell Carcinoma2	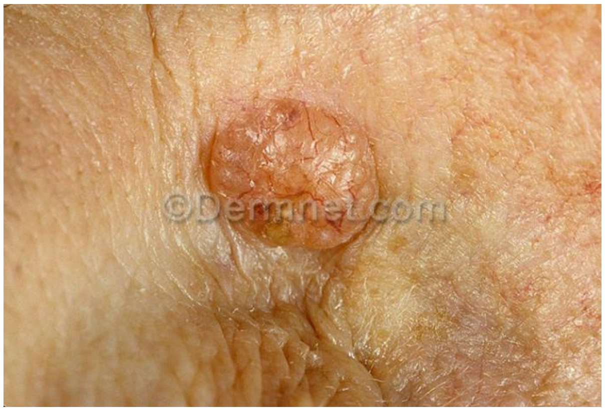
SkinGPT-4	Dermatology Physician
Q1. Could you describe the skin disease in this image for me?<br><br>A1. This could be a skin disease image of basal cell carcinoma lid.<br><br>Q2. You should write a paragraph to list more features you observed in the image.<br><br>A2. The image is a skin disease image of basal cell carcinoma on the lid. The lesion appears as a pearly white or flesh-colored nodule with a smooth surface. It has a central depression, which can be seen in the image. The borders of the lesion are well-defined and the size is approximately 5 mm in diameter. The lesion is located on the lower eyelid, close to the lash line. There is no evidence of ulceration or bleeding. The surrounding skin appears normal. The color of the lesion is pale and its surface is smooth. It has a regular, somewhat round shape and the lesion is slightly elevated above the skin surface. The lesion has a central depression and there are black dots inside the lesion. The lesion is surrounded by fine, white, milky-looking skin changes. There is no scaling, crusting or redness around the lesion.<br><br>Q3. With previous information, please write a paragraph to explain in detail the cause of this skin disease.<br><br>A3. Basal cell carcinoma is a common type of skin cancer that arises from the basal cells, which are responsible for producing skin cells. Exposure to ultraviolet radiation from the sun is the most	Q1. Could you describe the skin disease in this image for me?<br><br>A1. Tumor, basal cell carcinoma(?)<br><br>Q2. You should write a paragraph to list more features you observed in the image.<br><br>A2. A papules with dendritic dilatation of the blood vessels<br><br>Q3. With previous information, please write a paragraph to explain in detail the cause of this skin disease.<br><br>A3. BCC is a tumor that arises within sun-damaged skin. It rarely develops on the palms and soles or mucous membranes.<br><br>Q4. How should I treat it? What medicine should I use?<br><br>A4. The dermoscopy should be done first. A necessary diagnostic tool is a surgical biopsy. Mohs surgery is the preferred treatment for morpheaform, recurrent, poorly demarcated, highrisk, or incompletely removed BCC, and for those sites in which tissue conservation is paramount and there is a need for reliable clear margins.

important risk factor for developing basal cell carcinoma, particularly on areas of the skin that are frequently exposed to the sun, such as the face, neck, and arms. The cancer typically appears as a small, flesh-colored or pearly nodule, and can have a smooth or slightly rough surface. Over time, it can grow and become ulcerated or bleed. The exact mechanism of development of basal cell carcinoma is not fully understood, but it is thought to be related to mutations in the p53 gene, which is responsible for regulating cell growth and division. The mutations lead to uncontrolled growth and division of basal cells, resulting in the formation of a tumor. Basal cell carcinoma is generally a slow-growing cancer, but it can be invasive and destructive if left untreated, leading to disfigurement and even death in rare cases.<br><br>Q4. How should I treat it? What medicine should I use?<br><br>A4. Treatment of basal cell carcinoma on the lid typically involves surgical removal of the tumor. The lesion can be removed by a procedure called destructive surgery, where the tumor is removed along with some of the surrounding tissue, or by excisional surgery, where the tumor is removed while preserving as much normal tissue as possible. In some cases, a combination of both methods may be used.<br>Medications such as imiquimod, 5-fluorouracil, and cidofovir are used for topical treatment of basal cell carcinoma. However, these medications are not commonly used for basal cell carcinoma on the lid and are more often used for other types of skin cancer or precancerous changes.<br>In some cases, radiation therapy may also be used as a treatment option.<br>It is important to note that surgery is the primary treatment for basal cell carcinoma, and the choice of medication depends on the location, size, and other factors of the tumor.<br>It is always best to consult a dermatologist for proper diagnosis and treatment of skin cancer.	
Evaluation of Physician	
Evaluation Item	Score (1-5, the higher the better)

SkinGPT-4's diagnosis is correct or relevant.	4
SkinGPT-4's description is informative.	5
SkinGPT-4's suggestions are useful.	5
SkinGPT-4 can help doctors with diagnosis.	5
SkinGPT-4 can help patients to understand their disease better.	5
If SkinGPT-4 can be deployed locally, it protects patients' privacy.	5
Willingness to use SkinGPT-4	5

Case Basal Cell Carcinoma 3

Image ID: Basal Cell Carcinoma3	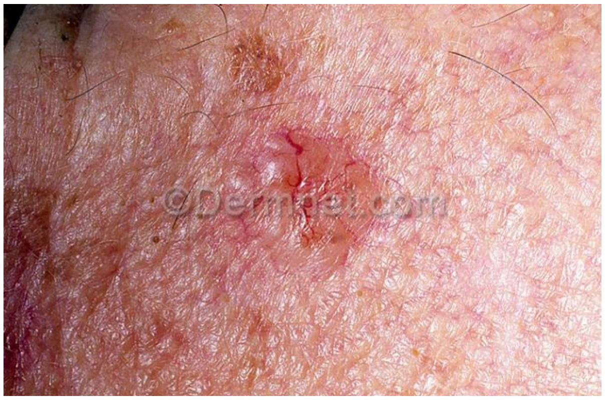
**SkinGPT-4**	**Dermatology Physician**
Q1. Could you describe the skin disease in this image for me?<br><br>A1. This could be a skin disease image of basal cell carcinoma lesion.<br><br>Q2. You should write a paragraph to list more features you observed in the image.<br><br>A2. This could be a skin disease image of basal cell carcinoma. The lesion appears as a pearly white or skin colored nodule or plaque, with sharply defined borders and a flat or slightly depressed surface. It can be found on sun exposed areas, such as the face, neck, hands, and legs. Basal cell carcinoma is a common type of skin cancer that affects the basal cells, which line the hair follicles. It usually grows slowly and does not spread to other parts of the body. However, if left untreated, it can become large and disfiguring, and may invade surrounding tissues and nerves.<br><br>Q3. With previous information, please write a paragraph to explain in detail the cause of this skin disease.<br><br>A3. Basal cell carcinoma is caused by exposure to ultraviolet (UV) radiation from the sun or other sources, such as tanning beds. The damage to the skin's DNA from UV radiation can lead to the development of skin cancer. The most common cause of basal cell carcinoma is prolonged sun exposure, especially in fair skin types. Other	Q1. Could you describe the skin disease in this image for me?<br><br>A1. Erythema, vasodilation(?); Actinic Keratosis(?); Basal Cell Carcinoma(?)<br><br>Q2. You should write a paragraph to list more features you observed in the image.<br><br>A2. An erythema with vasodilation.<br><br>Q3. With previous information, please write a paragraph to explain in detail the cause of this skin disease.<br><br>A3. BCC is a tumor that arises within sun-damaged skin. It rarely develops on the palms and soles or mucous membranes.<br><br>Q4. How should I treat it? What medicine should I use?<br><br>A4. A dermoscopy should be done first. A necessary diagnostic tool is a surgical biopsy.

factors that may increase the risk of developing basal cell carcinoma include exposure to radiation, certain chemicals, and a history of skin damage such as sunburns or skin inflammation.<br>The cancerous cells in basal cell carcinoma usually originate from the basal layer of the epidermis, which is the lowest layer of the skin.<br>The lesion may appear as a white, pink or reddish-white patch or bump, with a smooth or pitted surface, and may bleed or ooze. It can be found on any part of the body, but most commonly on the face, neck, and hands. Basal cell carcinoma is usually treatable, but can be disfiguring if not treated properly.<br><br>Q4. How should I treat it? What medicine should I use?<br><br>A4. Treatment for basal cell carcinoma depends on the size, location, and severity of the lesion. The most common treatments for basal cell carcinoma are surgical excision, radiation therapy, and topical medications.<br>Surgical excision involves removing the cancerous lesion and some surrounding tissue to ensure that the cancer is completely removed. This is usually done under local anesthesia and leaves a small scar.<br>Radiation therapy involves using high-energy X-rays or other forms of radiation to kill the cancer cells. It is usually done in a series of treatments over several weeks and has minimal side effects.<br>Topical medications, such as imiquimod or 5-fluorouracil, work by stimulating the body's immune system to fight the cancer cells. They are applied directly to the skin and are usually used for smaller lesions.<br>In some cases, a combination of these treatments may be used. The choice of treatment depends on the individual case and is best determined by a dermatologist or other skin cancer specialist.	
Evaluation of Physician	
Evaluation Item	Score (1-5, the higher the better)
SkinGPT-4's diagnosis is correct or relevant.	3
SkinGPT-4's description is informative.	5

SkinGPT-4's suggestions are useful.	5
SkinGPT-4 can help doctors with diagnosis.	5
SkinGPT-4 can help patients to understand their disease better.	5
If SkinGPT-4 can be deployed locally, it protects patients' privacy.	5
Willingness to use SkinGPT-4	5

Case Basal Cell Carcinoma 4

Image ID: Basal Cell Carcinoma4	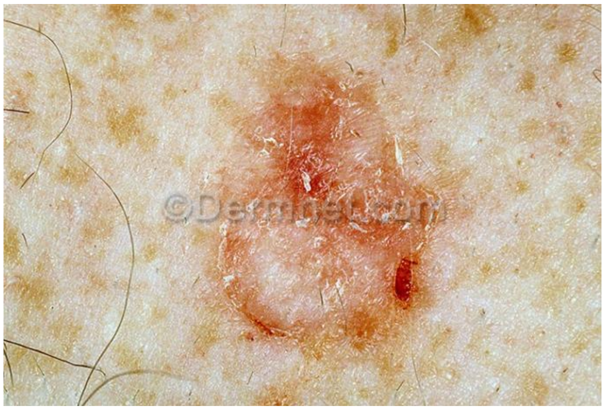
**SkinGPT-4**	**Dermatology Physician**
Q1. Could you describe the skin disease in this image for me?<br><br>A1. This could be a skin disease image of basal cell carcinoma lesion.<br><br>Q2. You should write a paragraph to list more features you observed in the image.<br><br>A2. The lesion appears erythematous and slightly elevated with definite irregularities on the surface. It has a central depression and there are many superficial vessels. The lesion is located on the left side of the face, above the upper lip, on the mid cheek. No white rolled borders are seen. The lesion is about 6 mm in diameter.<br><br>Q3. With previous information, please write a paragraph to explain in detail the cause of this skin disease.<br><br>A3. The cause of this skin disease is ultraviolet radiation from the sun. The lesion is a type of skin cancer called basal cell carcinoma, which commonly occurs on parts of the body that are exposed to the sun, such as the face and neck. It is characterized by slow growth, a pearly appearance, and small blood vessels on the surface. Basal cell carcinoma can be caused by prolonged exposure to sunlight, as well as other factors such as radiation and burns. It is important to protect the skin from the sun by using sunscreen, wearing protective clothing, and seeking shade during peak sun hours. Regular	Q1. Could you describe the skin disease in this image for me?<br><br>A1. Tumor, Basal Cell Carcinoma(?)<br><br>Q2. You should write a paragraph to list more features you observed in the image.<br><br>A2. Central scarred(?) red papules.<br><br>Q3. With previous information, please write a paragraph to explain in detail the cause of this skin disease.<br><br>A3. BCC is a tumor that arises within sun-damaged skin. It rarely develops on the palms and soles or mucous membranes.<br><br>Q4. How should I treat it? What medicine should I use?<br><br>A4. A dermoscopy should be done first. A necessary diagnostic tool is a surgical biopsy.

skin exams and self-skin exams can also help detect basal cell carcinoma early, when it is most treatable.<br><br>Q4. How should I treat it? What medicine should I use?<br><br>A4. The treatment of basal cell carcinoma depends on the size, location, and severity of the lesion. Surgery is the most common treatment for basal cell carcinoma, and involves removing the cancerous tissue and some surrounding healthy tissue. Cure rates for surgical treatment of basal cell carcinoma are high, with a 95% success rate. Other treatments for basal cell carcinoma include radiation therapy, cryosurgery, and topical medications. The choice of treatment depends on the size, location, and severity of the lesion, as well as the patient's preferences and overall health. A dermatologist should be consulted for proper treatment.	
Evaluation of Physician	
Evaluation Item	Score (1-5, the higher the better)
SkinGPT-4's diagnosis is correct or relevant.	3
SkinGPT-4's description is informative.	5
SkinGPT-4's suggestions are useful.	5
SkinGPT-4 can help doctors with diagnosis.	5
SkinGPT-4 can help patients to understand their disease better.	5
If SkinGPT-4 can be deployed locally, it protects patients' privacy.	5
Willingness to use SkinGPT-4	5

Case Basal Cell Carcinoma 5

Image ID: Basal Cell Carcinoma5	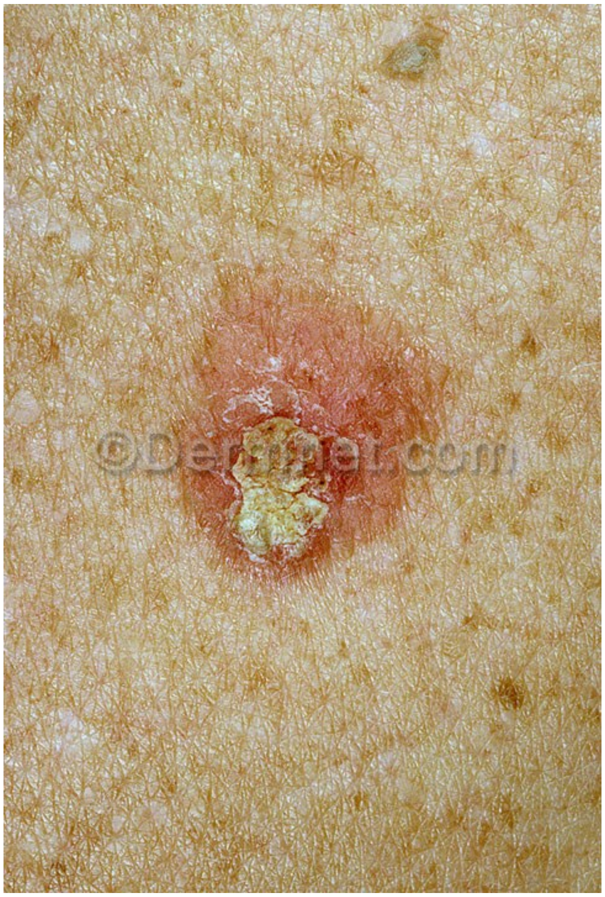
**SkinGPT-4**	**Dermatology Physician**
Q1. Could you describe the skin disease in this image for me?<br><br>A1. This could be a skin disease image of basal cell carcinoma lesion.<br><br>Q2. You should write a paragraph to list more features you observed in the image.<br><br>A2. The lesion appears to be a basal cell carcinoma, a common skin cancer characterized by its tendency to invade surrounding tissues. It presents as a pearly or waxy nodule with sharply defined borders and a central depression. The lesion is relatively smooth and shiny with a pinkish hue, and is often found on sun exposed areas such as the face, neck, and arms. This particular lesion has a central degenerative/ulcerated area,	Q1. Could you describe the skin disease in this image for me?<br><br>A1. Actinic Keratosis (?), Basal Cell Carcinoma (?)<br><br>Q2. You should write a paragraph to list more features you observed in the image.<br><br>A2. Red papules with keratinised surface.<br><br>Q3. With previous information, please write a paragraph to explain in detail the cause of this skin disease.<br><br>A3. BCC is a tumor that arises within sun-damaged skin. It rarely develops on the palms and soles or mucous membranes.<br>Q4. How should I treat it? What medicine should I

which is a common feature of basal cell carcinomas.<br><br>Q3. With previous information, please write a paragraph to explain in detail the cause of this skin disease.<br><br>A3. Basal cell carcinoma is caused by long-term exposure to ultraviolet (UV) radiation from the sun or other sources. This type of skin cancer typically develops in areas of the skin that are most exposed to the sun, such as the face, neck, and arms. The damage caused by UV radiation can trigger mutations in the skin cells, leading to the development of cancer. In this particular case, the lesion has a central degenerative/ulcerated area, which is a common feature of basal cell carcinomas. The ulceration may be due to the cancer invading surrounding tissues, which can cause destruction of the skin cells and tissue. The lesion also has a pearly or waxy appearance and is relatively smooth, which is characteristic of basal cell carcinomas.<br><br>Q4. How should I treat it? What medicine should I use?<br><br>A4. Treatment options for basal cell carcinoma include surgery, radiation therapy, and topical medications. The choice of treatment depends on factors such as the size, location, and extent of the cancer.<br>For this particular case, surgical excision is the first line of treatment for basal cell carcinoma. The lesion can be removed by a dermatologist or a surgeon, and the wound can be closed with sutures or a skin graft. In some cases, a Mohs micrographic surgery may be performed to remove the cancer and a small amount of surrounding tissue. This procedure is done in the office under local anesthesia, and it has the highest cure rate for basal cell carcinoma.<br><br>If the lesion is small and isolated, topical medications such as imiquimod or 5-fluorouracil can be used. These medications work by stimulating the body's immune system to destroy the cancer cells.<br>In some cases, radiation therapy may be used as an alternative to surgery for patients who cannot undergo surgery due to medical or social reasons. It's important to note that the lesion should be	use?<br><br>A4. A dermoscopy should be done first. A necessary diagnostic tool is a surgical biopsy.

removed as soon as possible to prevent it from growing larger and invading surrounding tissues. So, it's advisable to consult a dermatologist or a skin cancer specialist for proper evaluation	
Evaluation of Physician	
Evaluation Item	Score (1-5, the higher the better)
SkinGPT-4's diagnosis is correct or relevant.	4
SkinGPT-4's description is informative.	5
SkinGPT-4's suggestions are useful.	5
SkinGPT-4 can help doctors with diagnosis.	5
SkinGPT-4 can help patients to understand their disease better.	5
If SkinGPT-4 can be deployed locally, it protects patients' privacy.	5
Willingness to use SkinGPT-4	5

Case Atopic Dermatitis 1

Image ID: Atopic Dermatitis1	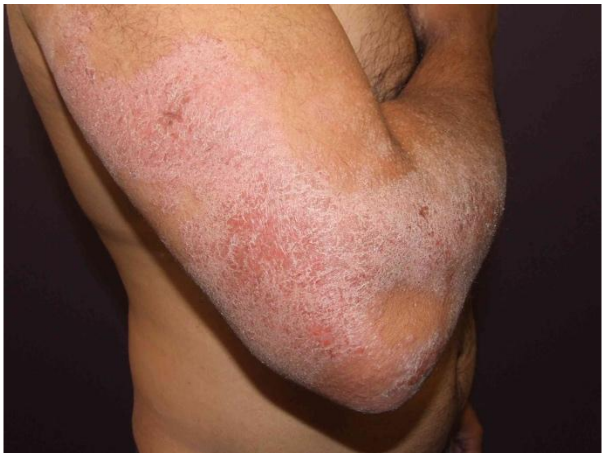
SkinGPT-4	Dermatology Physician
Q1. Could you describe the skin disease in this image for me?<br><br>A1. This is the appearance of atopic dermatitis.<br><br>Q2. You should write a paragraph to list more features you observed in the image.<br><br>A2. Atopic dermatitis is a chronic inflammatory skin condition characterized by dry, itchy, and scaly patches on the skin. It typically affects the skin on the face, hands, feet, and behind the knees. The condition often occurs in people with a family history of allergies, such as asthma or eczema. The symptoms of atopic dermatitis can be worsened by environmental factors such as stress, changes in temperature or humidity, or exposure to certain chemicals.<br><br>Q3. With previous information, please write a paragraph to explain in detail the cause of this skin disease.<br><br>A3. Atopic dermatitis is caused by a combination of genetic and environmental factors. It is more common in people with a family history of allergies, such as asthma or eczema, and tends to affect individuals who have a genetic predisposition to develop an overactive immune system. This leads to chronic inflammation and skin irritation.	Q1. Could you describe the skin disease in this image for me?<br><br>A1. Dermatitis/Eczema<br><br>Q2. You should write a paragraph to list more features you observed in the image.<br><br>A2. Dermatitis/Eczema<br><br>Q3. With previous information, please write a paragraph to explain in detail the cause of this skin disease.<br><br>A3. Atopic dermatitis (AD) is the most common chronic inflammatory skin disease, and its increasing prevalence presents a major public health problem worldwide. AD is characterized by intense pruritus and a chronic or chronically relapsing course. Acute inflammation and involvement of the cheeks, scalp and extensor aspects of the extremities predominates in infants, shifting to chronic inflammation with lichenification and a predilection for flexural sites in children and adults. A proactive approach to management is recommended, including avoidance of trigger factors, daily use of emollients, and anti-inflammatory therapy to control subclinical inflammation as well as overt flares.<br><br>Q4. How should I treat it? What medicine should I use?

Environmental factors, such as stress, changes in temperature or humidity, or exposure to certain chemicals, can also worsen the symptoms of atopic dermatitis. These factors can trigger an allergic response or irritate the skin, leading to flare-ups.<br><br>It is important to note that the exact cause of atopic dermatitis is not fully understood and it is likely that there are multiple factors that contribute to the development of the condition.<br><br>Q4. How should I treat it? What medicine should I use?<br><br>A4. Treatment for atopic dermatitis aims to manage symptoms and prevent flare-ups. The most effective treatment plan will depend on the severity of the condition and the specific symptoms that are present.<br><br>Treatment options for atopic dermatitis may include:<br>1. Topical creams and ointments: These are applied directly to the skin and can help to reduce itching and inflammation. Examples include corticosteroids, calcineurin inhibitors, and antihistamines.<br>2. Phototherapy: This involves exposing the skin to ultraviolet light, which can help to reduce inflammation and improve symptoms.<br>3. Systemic medications: These are taken orally or by injection and can help to reduce inflammation throughout the body. Examples include immunosuppressants and biologic therapies.<br>4. Lifestyle changes: Making certain lifestyle changes can help to manage symptoms of atopic dermatitis. This may include avoiding triggers, such as stress or certain chemicals, and keeping the skin moisturized.<br>It is important to work with a dermatologist to develop a treatment plan that is tailored to your specific needs and symptoms. They can help to determine the most effective treatments and provide guidance on how to use them effectively.	A4. Currently, management of AD includes the following components:<br>(1) avoidance of trigger factors, including irritants, relevant allergens<br>and microbial agents.<br>(1) skin care that aims to compensate for the genetically determined<br>impaired epidermal barrier function.<br>(3) anti-inflammatory therapy to control subclinical inflammation as<br>well as overt flares.
Evaluation of Physician	
Evaluation Item	Score (1-5, the higher the better)

SkinGPT-4's diagnosis is correct or relevant.	5
SkinGPT-4's description is informative.	5
SkinGPT-4's suggestions are useful.	5
SkinGPT-4 can help doctors with diagnosis.	5
SkinGPT-4 can help patients to understand their disease better.	5
If SkinGPT-4 can be deployed locally, it protects patients' privacy.	5
Willingness to use SkinGPT-4	5

Case Atopic Dermatitis 2

Image ID: Atopic Dermatitis2	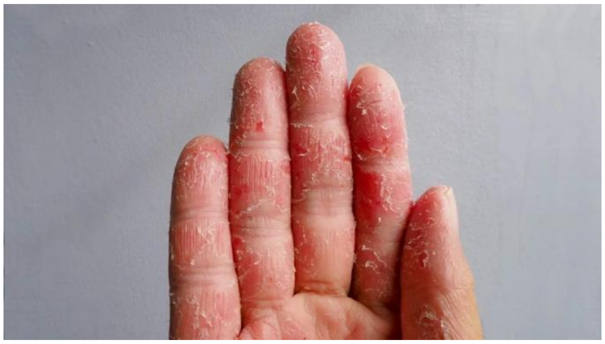
SkinGPT-4	Dermatology Physician
Q1. Could you describe the skin disease in this image for me?<br><br>A1. This could be a skin disease image of eczema fingertips.<br><br>Q2. You should write a paragraph to list more features you observed in the image.<br><br>A2. This image depicts eczema, which is a chronic inflammatory skin condition characterized by red, itchy, and scaly patches on the skin. The hands in the image appear to have been affected by eczema, with red, inflamed patches covering the palms and fingers. Eczema is caused by a combination of genetic and environmental factors, and can be treated with a variety of topical and oral medications, as well as lifestyle changes. It is important to seek medical attention if you have eczema, as it can cause discomfort and disrupt your daily activities.<br><br>Q3. With previous information, please write a paragraph to explain in detail the cause of this skin disease.<br><br>A3. Atopic dermatitis, also known as eczema, is caused by a combination of genetic and environmental factors. It is a chronic inflammatory skin condition that affects the skin's barrier function and results in dry, itchy, and scaly patches on the skin.<br><br>The exact causes of atopic dermatitis are not fully understood, but it is thought to be related to an overactive immune system and a genetic	Q1. Could you describe the skin disease in this image for me?<br><br>A1. Dermatitis/Eczema<br><br>Q2. You should write a paragraph to list more features you observed in the image.<br><br>A2. Dermatitis/Eczema<br><br>Q3. With previous information, please write a paragraph to explain in detail the cause of this skin disease.<br><br>A3. Atopic dermatitis (AD) is the most common chronic inflammatory skin disease, and its increasing prevalence presents a major public health problem worldwide. AD is characterized by intense pruritus and a chronic or chronically relapsing course. Acute inflammation and involvement of the cheeks, scalp and extensor aspects of the extremities predominates in infants, shifting to chronic inflammation with lichenification and a predilection for flexural sites in children and adults. A proactive approach to management is recommended, including avoidance of trigger factors, daily use of emollients, and anti-inflammatory therapy to control subclinical inflammation as well as overt flares.<br><br>Q4. How should I treat it? What medicine should I use?<br><br>A4. In this case, topical corticosteroids and calcineurin inhibitors may be used to control the symptoms. In addition, emollients (basic therapy)

predisposition. Factors that can trigger or worsen atopic dermatitis include stress, changes in temperature or humidity, exposure to certain chemicals or substances, and certain types of clothing or bedding.<br><br>Q4. How should I treat it? What medicine should I use?<br><br>A4. Treatment for atopic dermatitis focuses on managing symptoms and preventing flare-ups. The specific treatment plan will depend on the severity of the condition and the individual's specific symptoms and triggers.<br><br>Some common treatment options for atopic dermatitis include:<br><br>1. Topical creams or ointments: These can help reduce inflammation and relieve itching. Examples include corticosteroids, calcineurin inhibitors, and antihistamines.<br>2. Avoiding triggers: Identifying and avoiding triggers that can make symptoms worse, such as certain fabrics, soaps, or environmental factors.<br>3. Moisturizing: Keeping the skin well-moisturized can help prevent dryness and itching. This can be done with over-the-counter creams or ointments, or by taking baths or showers with added moisture.<br>4. Phototherapy: In some cases, exposure to ultraviolet light may be used to help improve symptoms.<br>5. Systemic medications: For severe cases of atopic dermatitis, systemic medications such as cyclosporine or methotrexate may be prescribed.	are important.
Evaluation of Physician	
Evaluation Item	Score (1-5, the higher the better)
SkinGPT-4's diagnosis is correct or relevant.	4
SkinGPT-4's description is informative.	5
SkinGPT-4's suggestions are useful.	5
SkinGPT-4 can help doctors with diagnosis.	5

SkinGPT-4 can help patients to understand their disease better.	5
If SkinGPT-4 can be deployed locally, it protects patients' privacy.	5
Willingness to use SkinGPT-4	5

Case Eczema 1

Image ID: eczema1	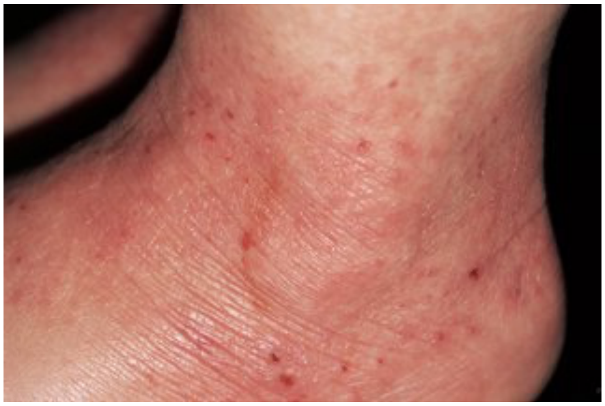
**SkinGPT-4**	**Dermatology Physician**
Q1. Could you describe the skin disease in this image for me?<br><br>A1. atopic dermatitis and eczema<br><br>Q2. You should write a paragraph to list more features you observed in the image.<br><br>A2. Atopic dermatitis and eczema are chronic skin conditions characterized by red, itchy, and inflamed skin. Both conditions are caused by a combination of genetic and environmental factors and can be triggered by factors such as stress, changes in temperature or humidity, or exposure to certain chemicals or substances. In this image, the skin appears red and inflamed with small blisters and clear fluid on the surface. The skin is also very itchy, which is a common symptom of both atopic dermatitis and eczema. Overall, this image shows the features of atopic dermatitis and eczema, including redness, inflammation, itching, and blistering.<br><br>Q3. With previous information, please write a paragraph to explain in detail the cause of this skin disease.<br><br>A3. Atopic dermatitis and eczema are chronic skin conditions that can be caused by a combination of genetic and environmental factors. In the case of atopic dermatitis, the immune system is overactive and produces excessive amounts of inflammatory chemicals, leading to red, itchy, and inflamed skin. Eczema, on the other hand, is caused by a reaction to certain substances or environmental	Q1. Could you describe the skin disease in this image for me?<br><br>A1. Dermatitis/Eczema<br><br>Q2. You should write a paragraph to list more features you observed in the image.<br><br>A2. Erythema on ankle with scratched surface.<br><br>Q3. With previous information, please write a paragraph to explain in detail the cause of this skin disease.<br><br>A3. Eczema is associated with the development of food and environmental allergies.<br><br>Q4. How should I treat it? What medicine should I use?<br><br>A4. Possible treatments include taking oral loratadine syrup, applying a mixture of Euthyroxine and mupirocin topically, using compound cypress liquid topically if there is exudation, reducing bathing, and using emollient cream.

factors that come into contact with the skin. In both cases, the skin becomes irritated and inflamed, leading to the development of symptoms such as redness, itching, and blistering. In this image, the skin appears red and inflamed with small blisters and clear fluid on the surface. The skin is also very itchy, which is a common symptom of both atopic dermatitis and eczema. Overall, this image shows the features of atopic dermatitis and eczema, including redness, inflammation, itching, and blistering.<br><br>Q4. How should I treat it? What medicine should I use?<br><br>Treatment for atopic dermatitis and eczema depends on the severity of the condition and the specific symptoms present. In general, treatment may include a combination of the following options:<br>1. Topical corticosteroids: These are medicated creams or ointments that help reduce inflammation and itching. They are often prescribed for short-term use to help manage flare-ups.<br>2. Antihistamines: These medications help reduce itching and hives. They are often prescribed for short-term use to help manage symptoms.<br>3. Immunomodulators: These medications help reduce inflammation and the immune system's response. They are often prescribed for long-term use to help manage symptoms.<br>4. Moisturizers: Keeping the skin moisturized is important to prevent dryness and itching. Over-the-counter creams or ointments can be used, or a healthcare professional can recommend a more intense moisturizer if needed.<br>It is important to consult a dermatologist or other healthcare professional for proper diagnosis and treatment of atopic dermatitis and eczema. They can provide personalized advice and treatment options based on the severity and specific symptoms of the condition.	
Evaluation of Physician	
Evaluation Item	Score (1-5, the higher the better)
SkinGPT-4's diagnosis is correct or relevant.	5

SkinGPT-4's description is informative.	5
SkinGPT-4's suggestions are useful.	5
SkinGPT-4 can help doctors with diagnosis.	5
SkinGPT-4 can help patients to understand their disease better.	5
If SkinGPT-4 can be deployed locally, it protects patients' privacy.	5
Willingness to use SkinGPT-4	5

Case Eczema 2

Image ID: eczema2	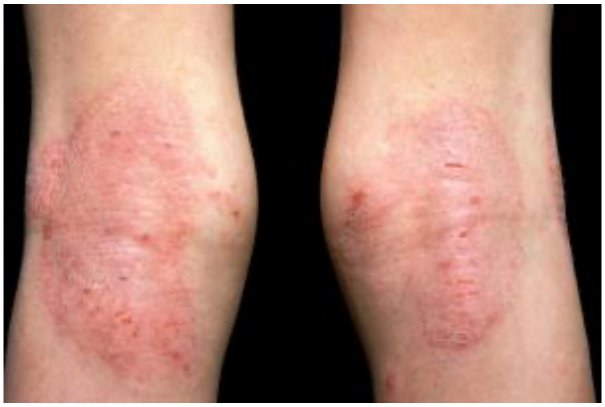
**SkinGPT-4**	**Dermatology Physician**
Q1. Could you describe the skin disease in this image for me?<br><br>A1. This could be a skin disease image of eczema.<br><br>Q2. You should write a paragraph to list more features you observed in the image.<br><br>A2. In the image, there is a representation of a person's body with red patches on the skin. The patches are circular in shape and have clear boundaries. They appear to be on the arms and legs, with a few on the torso. The patches are inflamed, with redness and soreness. They are surrounded by a lighter ring, which could be a sign of inflammation. The image suggests the person may be suffering from a skin condition, possibly eczema or nummular eczema. The patches could cause discomfort, itching, and dryness, leading to a flare up of the condition. It is essential to seek medical attention for proper diagnosis and treatment to manage the symptoms and prevent complications.<br><br>Q3. With previous information, please write a paragraph to explain in detail the cause of this skin disease.<br><br>A3. The cause of eczema is not fully understood, but it is believed to be an immune disorder that affects the skin's barrier function. Eczema occurs when the immune system reacts to certain triggers, such as environmental allergens, stress, or chemical irritants, and causes the skin to	Q1. Could you describe the skin disease in this image for me?<br><br>A1. Dermatitis/Eczema<br><br>Q2. You should write a paragraph to list more features you observed in the image.<br><br>A2. Erythema occurring in the popliteal fossa with a scratched surface.<br><br>Q3. With previous information, please write a paragraph to explain in detail the cause of this skin disease.<br><br>A3. The causes of eczema are complex, with both intrinsic and extrinsic factors.<br>The interaction between these factors is often complex. External factors such as living environment and climate External factors such as living environment and climate conditions can influence the development of eczema. External stimuli such as sunlight, ultraviolet rays, cold, heat, humidity and solar radiation can affect the development of eczema.<br>Some everyday products such as balms, soaps, artificial fibres, etc. can also affect the development of eczema.<br>Eczema can also be triggered by certain foods, such as chronic digestive disorders, functional disorders of the gastrointestinal tract.<br>Psychological changes such as emotional changes, infectious lesions, metabolic disorders and endocrine dysfunction can all contribute to eczema.

become inflamed and irritated. This leads to the release of certain chemical mediators, such as histamine, which cause the symptoms of eczema, including redness, itching, and blistering. Additionally, genetics may play a role in the development of eczema, as the condition tends to run in families. The condition is not contagious and cannot be spread from one person to another.<br><br>Q4. How should I treat it? What medicine should I use?<br><br>A4. The treatment of eczema depends on the severity and type of the condition. Mild cases of eczema can be treated with over-the-counter creams or ointments containing hydrocortisone, which help to reduce inflammation and itching. However, if the eczema is severe or does not respond to over-the-counter treatments, a doctor may prescribe stronger medications, such as corticosteroid tablets or injectable medications. In addition to medication, it is important to follow a regular skin care routine, which may include gentle soap and water, avoiding harsh chemicals and irritants, and using a moisturizer to help soothe and hydrate the skin. Phototherapy, or exposure to ultraviolet light, may also be used to treat severe cases of eczema that do not respond to other treatments.<br>It is important to note that treatment for eczema may take time to be effective and that it is important to follow the treatment plan prescribed by a doctor. If the symptoms do not improve or if they worsen, it is important to consult a doctor again.	Q4. How should I treat it? What medicine should I use?<br><br>A4. In this case, topical corticosteroids and calcineurin inhibitors may be used to control the symptoms. In addition, emollients (basic therapy) are important.
Evaluation of Physician	
Evaluation Item	Score (1-5, the higher the better)
SkinGPT-4's diagnosis is correct or relevant.	5
SkinGPT-4's description is informative.	5
SkinGPT-4's suggestions are useful.	5
SkinGPT-4 can help doctors with diagnosis.	5

SkinGPT-4 can help patients to understand their disease better.	5
If SkinGPT-4 can be deployed locally, it protects patients' privacy.	5
Willingness to use SkinGPT-4	4

Case Eczema 3

<table>
<tr><td>Image ID: eczema3</td><td>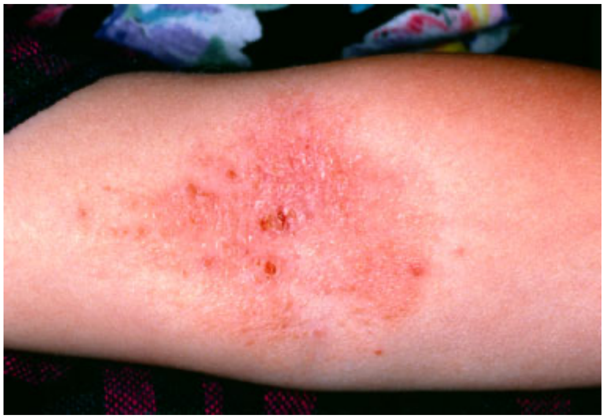</td></tr>
<tr><th>SkinGPT-4</th><th>Dermatology Physician</th></tr>
<tr><td>Q1. Could you describe the skin disease in this image for me?

A1. This could be a skin disease image of eczema

Q2. You should write a paragraph to list more features you observed in the image.

A2. The image shows a rash on the arm of a person, with small, red, raised bumps or pustules and white, fluid-filled blisters or vesicles. The rash appears to be scaly and have areas of crusting, indicating a bullous form of the disease. The lesions are distributed over the entire arm and appear to be very itchy, which is a common symptom of this condition. There is also some edema and erythema present, which suggests an allergic or autoimmune process. Overall, this image shows features of atopic dermatitis, a chronic inflammatory skin condition that is characterized by these symptoms and often occurs in people with a family history of allergies or other atopic conditions like asthma or eczema

Q3. With previous information, please write a paragraph to explain in detail the cause of this skin disease.

A3. The cause of this skin disease is likely to be a combination of factors, including genetics, environmental triggers, and lifestyle factors. The person may have a family history of skin conditions such as eczema or psoriasis, which increases their risk of developing this rash.</td><td>Q1. Could you describe the skin disease in this image for me?

A1. Dermatitis/Eczema

Q2. You should write a paragraph to list more features you observed in the image.

A2. Erythema on elbow fossa with scratched surface.

Q3. With previous information, please write a paragraph to explain in detail the cause of this skin disease.

A3. The causes of eczema are complex, with both intrinsic and extrinsic factors.
The interaction between these factors is often complex. External factors such as living environment and climate External factors such as living environment and climate conditions can influence the development of eczema. External stimuli such as sunlight, ultraviolet rays, cold, heat, humidity and solar radiation can affect the development of eczema.
Some everyday products such as balms, soaps, artificial fibres, etc. can also affect the development of eczema.
Eczema can also be triggered by certain foods, such as chronic digestive disorders, functional disorders of the gastrointestinal tract.
Psychological changes such as emotional changes, infectious lesions, metabolic disorders and endocrine dysfunction can all contribute to</td></tr>
</table>

Environmental factors, such as exposure to chemicals, stress, or changes in temperature or humidity, can also trigger the rash. The patient's lifestyle, including their diet, stress levels, and sleep patterns, may also contribute to the development of the rash. In addition, this rash could be a symptom of an underlying autoimmune disorder, such as lupus or another connective tissue disease, which affects the skin and other organs in the body. To determine the cause of this skin disease, a dermatologist or other medical professional may conduct a physical examination, review the patient's medical history, and perform tests to rule out other possible causes. Treatment will depend on the cause of the rash and the severity of the symptoms.<br><br>Q4. How should I treat it? What medicine should I use?<br><br>A4. The treatment for this skin disease will depend on the cause and severity of the symptoms. The dermatologist or other medical professional who examines the patient will recommend the appropriate treatment plan, which may include topical medications, oral medications, or a combination of both.<br>Topical medications, such as creams, ointments, or lotions, are commonly used to treat mild to moderate skin conditions. These medications work by reducing inflammation, relieving itching, and promoting healing of the skin. Examples of topical medications that may be prescribed for this condition include corticosteroids, which reduce inflammation and suppress the immune system, and calcineurin inhibitors, which suppress the immune system and reduce inflammation.<br>Oral medications, such as antihistamines or immunosuppressants, may be prescribed for more severe or widespread skin conditions. These medications work by targeting specific components of the immune system and reducing inflammation throughout the body.	eczema.<br><br>Q4. How should I treat it? What medicine should I use?<br><br>A4. In this case, topical corticosteroids and calcineurin inhibitors may be used to control the symptoms. In addition, emollients (basic therapy) are important. Oral antihistamines or glucocorticoids may be prescribed for more severe or widespread skin conditions.
Evaluation of Physician	
Evaluation Item	Score (1-5, the higher the better)
SkinGPT-4's diagnosis is correct or relevant.	5

SkinGPT-4's description is informative.	5
SkinGPT-4's suggestions are useful.	5
SkinGPT-4 can help doctors with diagnosis.	5
SkinGPT-4 can help patients to understand their disease better.	5
If SkinGPT-4 can be deployed locally, it protects patients' privacy.	5
Willingness to use SkinGPT-4	5

Case Eczema 4

<table>
<tr><td>Image ID: eczema4</td><td>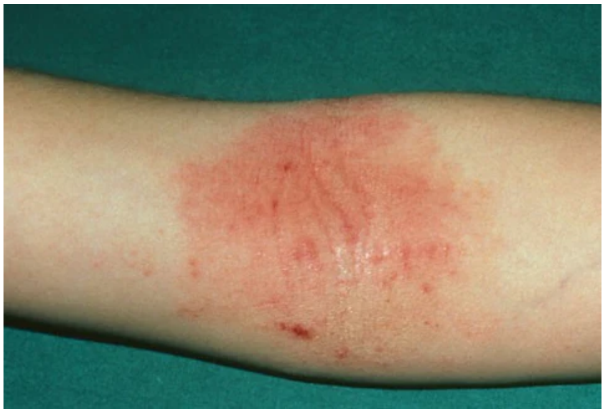</td></tr>
<tr><th>SkinGPT-4</th><th>Dermatology Physician</th></tr>
<tr><td>Q1. Could you describe the skin disease in this image for me?

A1. atopic dermatitis and eczema

Q2. You should write a paragraph to list more features you observed in the image.

A2. The image shows a severe infection of staphylococcal scalded skin disease with extensive sloughing and necrosis of the skin and subcutaneous tissue of the lower leg. The lesions are surrounded by erythema and edema and there is severe lymphadenopathy and tender indurations.

Q3. With previous information, please write a paragraph to explain in detail the cause of this skin disease.

A3. Staphylococcal scalded skin disease, also known as bullous impetigo, is a superficial bacterial infection caused by Staphylococcus aureus. It usually occurs on the skin of the lower legs, especially in people with atopic dermatitis or eczema. The infection results in the formation of large, painless, fluid - filled blisters that resemble burns. The blisters often rupture, leaving red and raw skin. The bacteria enter the skin through a break in the skin, usually caused by scratching or other trauma. Once inside the body, the bacteria produce a toxin that causes the skin to blister and flake. The disease is highly contagious and can be spread through direct contact with infected skin or</td><td>Q1. Could you describe the skin disease in this image for me?

A1. Dermatitis/Eczema

Q2. You should write a paragraph to list more features you observed in the image.

A2. Erythema on elbow fossa with scratched surface.

Q3. With previous information, please write a paragraph to explain in detail the cause of this skin disease.

A3. The causes of eczema are complex, with both intrinsic and extrinsic factors.
The interaction between these factors is often complex. External factors such as living environment and climate External factors such as living environment and climate conditions can influence the development of eczema. External stimuli such as sunlight, ultraviolet rays, cold, heat, humidity and solar radiation can affect the development of eczema.
Some everyday products such as balms, soaps, artificial fibres, etc. can also affect the development of eczema.
Eczema can also be triggered by certain foods, such as chronic digestive disorders, functional disorders of the gastrointestinal tract.
Psychological changes such as emotional changes, infectious lesions, metabolic disorders and endocrine dysfunction can all contribute to</td></tr>
</table>

through contaminated objects or surfaces. Treatment involves antibiotics and keeping the affected area clean and dry to prevent infection and promote healing.<br><br>Q4. How should I treat it? What medicine should I use?<br><br>A4. Staphylococcal scalded skin disease is typically treated with topical antibiotics, such as fusidic acid or mupirocin, to clear the infection. In addition, oral antibiotics may be prescribed if the infection is severe or does not respond to topical treatment. Pain relief can be managed with over-the-counter pain relievers, such as acetaminophen or ibuprofen. Keeping the affected area clean and dry is important to prevent infection and promote healing.<br>It's important to follow the instructions provided by your doctor or pharmacist when using any medication. If you have any questions or concerns, you should contact your doctor or pharmacist for advice.	eczema.<br><br>Q4. How should I treat it? What medicine should I use?<br><br>A4. In this case, topical corticosteroids and calcineurin inhibitors may be used to control the symptoms. In addition, emollients (basic therapy) are important. Oral antihistamines or glucocorticoids may be prescribed for more severe or widespread skin conditions.

Evaluation of Physician	
Evaluation Item	Score (1-5, the higher the better)
SkinGPT-4's diagnosis is correct or relevant.	5
SkinGPT-4's description is informative.	1
SkinGPT-4's suggestions are useful.	1
SkinGPT-4 can help doctors with diagnosis.	1
SkinGPT-4 can help patients to understand their disease better.	1
If SkinGPT-4 can be deployed locally, it protects patients' privacy.	1
Willingness to use SkinGPT-4	1

Case Eczema 5

<table>
<tr><td>Image ID: Eczema5</td><td>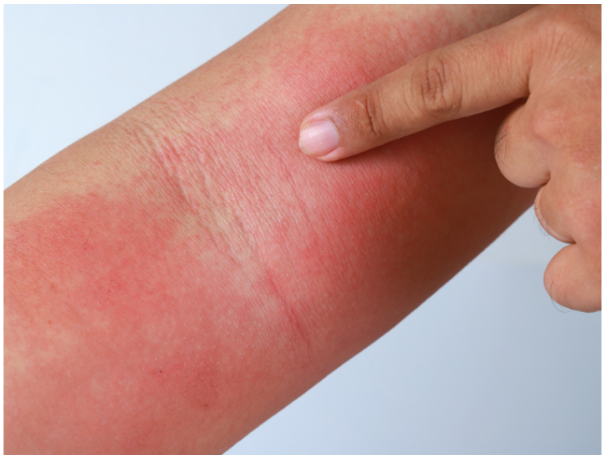</td></tr>
<tr><th>SkinGPT-4</th><th>Dermatology Physician</th></tr>
<tr><td>Q1. Could you describe the skin disease in this image for me?

A1. Eczema

Q2. You should write a paragraph to list more features you observed in the image.

A2. This image shows a person’s arm with a red rash on it. The rash is likely caused by an allergic reaction or eczema. The person is pointing to the rash on their arm, indicating that they are aware of the problem. The white background and the person’s hand and arm give the image a clean and clear look. The overall feeling of the image is one of discomfort and itching.

Q3. With previous information, please write a paragraph to explain in detail the cause of this skin disease.

A3. The red rash in the image is likely caused by an allergic reaction or eczema. An allergic reaction occurs when the body’s immune system reacts to a substance that it considers to be harmful, causing redness, swelling, and itching. Eczema is a chronic skin condition that causes red, itchy, and inflamed patches on the skin. It is caused by a combination of genetic and environmental factors. The exact cause of eczema is not fully understood, but it is thought to involve an overactive immune system and a problem with the</td><td>Q1. Could you describe the skin disease in this image for me?

A1. Dermatitis/Eczema; urticaria

Q2. You should write a paragraph to list more features you observed in the image.

A2. Large areas of oedematous erythema occurring on the upper extremities.

Q3. With previous information, please write a paragraph to explain in detail the cause of this skin disease.

A3. The causes of eczema are complex, with both intrinsic and extrinsic factors.
The interaction between these factors is often complex. External factors such as living environment and climate External factors such as living environment and climate conditions can influence the development of eczema. External stimuli such as sunlight, ultraviolet rays, cold, heat, humidity and solar radiation can affect the development of eczema.
Some everyday products such as balms, soaps, artificial fibres, etc. can also affect the development of eczema.
Eczema can also be triggered by certain foods, such as chronic digestive disorders, functional disorders of the gastrointestinal tract.
Psychological changes such as emotional</td></tr>
</table>

skin's barrier function.<br>Q4. How should I treat it? What medicine should I use?<br><br>A4. Treatment for a red rash caused by an allergic reaction or eczema will depend on the severity and cause of the rash. In general, the following steps can be taken to treat a red rash:<br><br>1. Avoid triggers: If the rash is caused by an allergen, such as certain medications or foods, it is important to avoid contact with the trigger.<br>2. Use over-the-counter creams: Creams and ointments containing hydrocortisone or calamine lotion can help to reduce itching and inflammation.<br>3. Take antihistamines: Antihistamines can help to reduce itching and inflammation caused by an allergic reaction.<br>4. Use moisturizers: Keeping the skin moisturized can help to prevent dryness and itching.<br>5. See a doctor: If the rash is severe or does not respond to over-the-counter treatments, it is important to see a doctor for further evaluation and treatment.<br>It is important to remember that treatment will depend on the severity and cause of the rash, so it is important to see a doctor for an accurate diagnosis and appropriate treatment.	changes, infectious lesions, metabolic disorders and endocrine dysfunction can all contribute to eczema.<br><br>Q4. How should I treat it? What medicine should I use?<br><br>A4. In this case, topical corticosteroids and calcineurin inhibitors may be used to control the symptoms. Oral antihistamines or glucocorticoids may be prescribed for more severe or widespread skin conditions.
Evaluation of Physician	
Evaluation Item	Score (1-5, the higher the better)
SkinGPT-4's diagnosis is correct or relevant.	4
SkinGPT-4's description is informative.	5
SkinGPT-4's suggestions are useful.	5
SkinGPT-4 can help doctors with diagnosis.	5
SkinGPT-4 can help patients to understand their disease better.	5
If SkinGPT-4 can be deployed locally, it protects patients' privacy.	5
Willingness to use SkinGPT-4	5

Case Eczema 6

Image ID: Eczema6	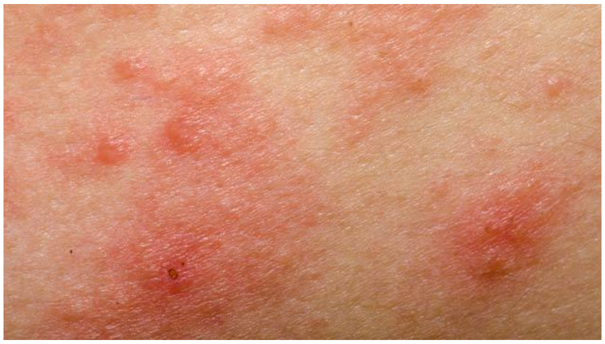
**SkinGPT-4**	**Dermatology Physician**
Q1. Could you describe the skin disease in this image for me?<br><br>A1. eczema<br><br>Q2. You should write a paragraph to list more features you observed in the image.<br><br>A2. The person has a rash on their skin, with small pink bumps covering the surface. The rash is slightly scaly and dry, with some of the bumps having a white center. The rash is most prominent on the forearms and lower legs, but also present on the torso and upper arms. The rash does not itch or cause any discomfort. There are no other symptoms, such as fever or fatigue. The person has not recently traveled to a tropical or subtropical region.<br><br>Q3. With previous information, please write a paragraph to explain in detail the cause of this skin disease.<br><br>A3. The rash shown in the picture is most likely caused by a type of eczema called atopic dermatitis. It is a chronic inflammatory skin condition that is characterized by red, itchy, and scaly patches on the skin. It is often found in people with a family history of allergies, such as asthma or hay fever, and can be triggered by environmental allergens or stress. The rash can be treated with topical creams, antihistamines, and avoiding triggers. It is important to seek a dermatologist for proper diagnosis and treatment.<br><br>Q4. How should I treat it? What medicine should I	Q1. Could you describe the skin disease in this image for me?<br><br>A1. Dermatitis/Eczema; Papular urticaria<br><br>Q2. You should write a paragraph to list more features you observed in the image.<br><br>A2. Large areas of red, scratched surface; erythematous urticarial papules.<br><br>Q3. With previous information, please write a paragraph to explain in detail the cause of this skin disease.<br><br>A3. The causes of eczema are complex, with both intrinsic and extrinsic factors.<br>The interaction between these factors is often complex. External factors such as living environment and climate External factors such as living environment and climate conditions can influence the development of eczema. External stimuli such as sunlight, ultraviolet rays, cold, heat, humidity and solar radiation can affect the development of eczema.<br>Some everyday products such as balms, soaps, artificial fibres, etc. can also affect the development of eczema.<br>Eczema can also be triggered by certain foods, such as chronic digestive disorders, functional disorders of the gastrointestinal tract.<br>Psychological changes such as emotional changes, infectious lesions, metabolic disorders and endocrine dysfunction can all contribute to eczema.

use?<br><br>A4. The treatment for atopic dermatitis, which is likely the cause of the rash in the image, will depend on the severity of the condition and the individual's specific symptoms. Some common treatments include:<br><br>1. Topical corticosteroids: These creams or ointments are applied directly to the skin to reduce inflammation and relieve itching.<br>2. Moisturizers: Using a hydrating cream or ointment can help to soothe and protect the skin.<br>3. Antihistamines: Oral antihistamines can help to relieve itching and reduce inflammation.<br>4. Avoiding triggers: Identifying and avoiding triggers such as certain fabrics, soaps, or chemicals can help to prevent flare-ups.<br>5. Bathe in lukewarm water: Avoid hot water which can dry out the skin and make itching worse.<br><br>It is important to consult a dermatologist or other healthcare professional for the best treatment plan. They will be able to evaluate the rash and determine the best course of action based on the individual's specific symptoms and medical history.	Q4. How should I treat it? What medicine should I use?<br><br>A4. In this case, topical corticosteroids and calcineurin inhibitors may be used to control the symptoms. Oral antihistamines or glucocorticoids may be prescribed for more severe or widespread skin conditions.
Evaluation of Physician	
Evaluation Item	Score (1-5, the higher the better)
SkinGPT-4's diagnosis is correct or relevant.	5
SkinGPT-4's description is informative.	4
SkinGPT-4's suggestions are useful.	5
SkinGPT-4 can help doctors with diagnosis.	5
SkinGPT-4 can help patients to understand their disease better.	5
If SkinGPT-4 can be deployed locally, it protects patients' privacy.	5
Willingness to use SkinGPT-4	5

Case Eczema 7

Image ID: Eczema7	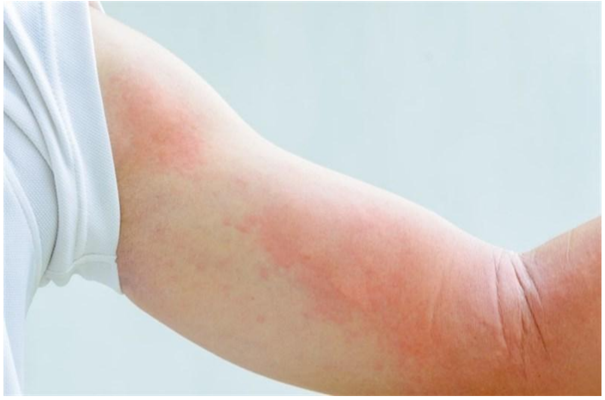
SkinGPT-4	Dermatology Physician
Q1. Could you describe the skin disease in this image for me?<br><br>A1. eczema<br><br>Q2. You should write a paragraph to list more features you observed in the image.<br><br>A2. The person in the image has a rash on their arm, which is red and itchy. The rash is most likely caused by an allergic reaction to a substance or an insect bite. It could also be a sign of an autoimmune disorder like lupus or psoriasis. It is important to see a doctor to determine the cause and receive proper treatment. The rash could be treated with over the counter creams or prescription medications depending on the cause.<br><br>Q3. With previous information, please write a paragraph to explain in detail the cause of this skin disease.<br><br>A3. The rash in the image could be caused by several things, including an allergic reaction to a substance, an insect bite, or an autoimmune disorder like lupus or psoriasis. The rash could also be caused by exposure to the sun or certain chemicals. The only way to determine the cause of the rash is to see a doctor and have them examine the rash and take a medical history. The doctor may also do tests like a biopsy or blood tests to determine the cause of the rash.<br><br>Q4. How should I treat it? What medicine should I	Q1. Could you describe the skin disease in this image for me?<br><br>A1. Dermatitis/Eczema, Urticaria<br><br>Q2. You should write a paragraph to list more features you observed in the image.<br><br>A2. Large areas of oedematous erythema occurring on the upper extremities, possibly with wheals.<br><br>Q3. With previous information, please write a paragraph to explain in detail the cause of this skin disease.<br><br>A3. The causes of eczema are complex, with both intrinsic and extrinsic factors.<br>The interaction between these factors is often complex. External factors such as living environment and climate External factors such as living environment and climate conditions can influence the development of eczema. External stimuli such as sunlight, ultraviolet rays, cold, heat, humidity and solar radiation can affect the development of eczema.<br>Some everyday products such as balms, soaps, artificial fibres, etc. can also affect the development of eczema.<br>Eczema can also be triggered by certain foods, such as chronic digestive disorders, functional disorders of the gastrointestinal tract.<br>Psychological changes such as emotional changes, infectious lesions, metabolic disorders

use?<br><br>A4. The treatment for the rash in the image will depend on the cause of the rash. If the rash is caused by an allergic reaction, the best treatment is to avoid the substance that caused the reaction. If the rash is caused by an insect bite, it can be treated with over-the-counter creams or ointments. If the rash is caused by an autoimmune disorder like lupus or psoriasis, prescription medications may be necessary to treat the condition.<br>If the rash is accompanied by fever, fatigue, and muscle aches, it could be a sign of a more serious underlying condition and it is important to see a doctor to determine the cause of the rash and receive proper treatment. The doctor may also prescribe medications to treat the symptoms and the underlying condition.<br>It's important to remember that self-treatment can be dangerous and may cause more harm than good. If you are not sure what is causing the rash or how to treat it, it is best to see a doctor.	and endocrine dysfunction can all contribute to eczema.<br><br>Q4. How should I treat it? What medicine should I use?<br><br>A4. In this case, topical corticosteroids and calcineurin inhibitors may be used to control the symptoms. Oral antihistamines or glucocorticoids may be prescribed for more severe or widespread skin conditions.
Evaluation of Physician	
Evaluation Item	Score (1-5, the higher the better)
SkinGPT-4's diagnosis is correct or relevant.	4
SkinGPT-4's description is informative.	4
SkinGPT-4's suggestions are useful.	5
SkinGPT-4 can help doctors with diagnosis.	5
SkinGPT-4 can help patients to understand their disease better.	5
If SkinGPT-4 can be deployed locally, it protects patients' privacy.	5
Willingness to use SkinGPT-4	5

Case Eczema 8

Image ID: Eczema8	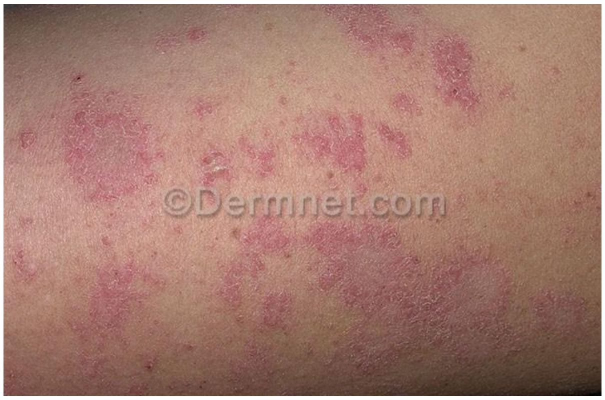
**SkinGPT-4**	**Dermatology Physician**
Q1. Could you describe the skin disease in this image for me?<br><br>A1. Erythema and atopic dermatitis<br><br>Q2. You should write a paragraph to list more features you observed in the image.<br><br>A2. Erythema chronicum atrophicans, also known as atopic dermatitis, is a chronic inflammatory skin condition characterized by red, itchy, and scaly patches on the skin. It is often seen in individuals with a family history of atopic diseases such as asthma, hay fever, or eczema. The rash is often found on the face, hands, and feet, and may be associated with other symptoms such as crusting, oozing, or blistering. Treatment options for atopic dermatitis include topical corticosteroids, topical antihistamines, and immunosuppressants.<br><br>Q3. With previous information, please write a paragraph to explain in detail the cause of this skin disease.<br><br>A3. Atopic dermatitis, also known as eczema, is caused by a combination of environmental and genetic factors. It is a chronic inflammatory skin condition that affects the skin's barrier function, leading to dryness, itching, and inflammation. The condition is often associated with a family history of atopic diseases such as asthma, hay fever, and eczema.<br>The exact cause of atopic dermatitis is not fully understood, but it is thought to involve a combination of genetic and environmental factors.	Q1. Could you describe the skin disease in this image for me?<br><br>A1. Dermatitis/Eczema<br><br>Q2. You should write a paragraph to list more features you observed in the image.<br><br>A2. Erythema, papules and pimples<br><br>Q3. With previous information, please write a paragraph to explain in detail the cause of this skin disease.<br><br>A3. The causes of eczema are complex, with both intrinsic and extrinsic factors.<br>The interaction between these factors is often complex. External factors such as living environment and climate External factors such as living environment and climate conditions can influence the development of eczema. External stimuli such as sunlight, ultraviolet rays, cold, heat, humidity and solar radiation can affect the development of eczema.<br>Some everyday products such as balms, soaps, artificial fibres, etc. can also affect the development of eczema.<br>Eczema can also be triggered by certain foods, such as chronic digestive disorders, functional disorders of the gastrointestinal tract.<br>Psychological changes such as emotional changes, infectious lesions, metabolic disorders and endocrine dysfunction can all contribute to eczema.

Some research suggests that there may be an underlying immune system dysfunction that contributes to the development of atopic dermatitis. This dysfunction may lead to an overactive immune response, resulting in inflammation and skin damage.<br>Other factors that may contribute to the development of atopic dermatitis include environmental allergens, stress, changes in temperature or humidity, and exposure to certain chemicals or substances.<br><br>Q4. How should I treat it? What medicine should I use?<br><br>A4. Treatment for atopic dermatitis, also known as eczema, depends on the severity and location of the rash, as well as the individual's overall health. The goal of treatment is to reduce inflammation, relieve symptoms, and prevent flare-ups.<br>Mild to moderate atopic dermatitis may be treated with topical creams or ointments, such as hydrocortisone, that help to reduce inflammation and relieve itching. Topical immunomodulators, such as pimecrolimus or tacrolimus, may also be prescribed to reduce inflammation and slow the growth of skin cells.<br>Severe atopic dermatitis may require oral medications, such as systemic steroids, to reduce inflammation throughout the body. In some cases, systemic immunosuppressants, such as cyclosporine, may be prescribed to further suppress the immune system and prevent flare-ups.	Q4. How should I treat it? What medicine should I use?<br><br>A4. In this case, topical corticosteroids and calcineurin inhibitors may be used to control the symptoms. In addition, emollients (basic therapy) are important. Oral antihistamines or glucocorticoids may be prescribed for more severe or widespread skin conditions.
Evaluation of Physician	
Evaluation Item	Score (1-5, the higher the better)
SkinGPT-4's diagnosis is correct or relevant.	4
SkinGPT-4's description is informative.	2
SkinGPT-4's suggestions are useful.	3
SkinGPT-4 can help doctors with diagnosis.	3
SkinGPT-4 can help patients to understand their disease better.	3

If SkinGPT-4 can be deployed locally, it protects patients' privacy.	5
Willingness to use SkinGPT-4	2

Case bullous disease 1

Image ID: bullous disease1	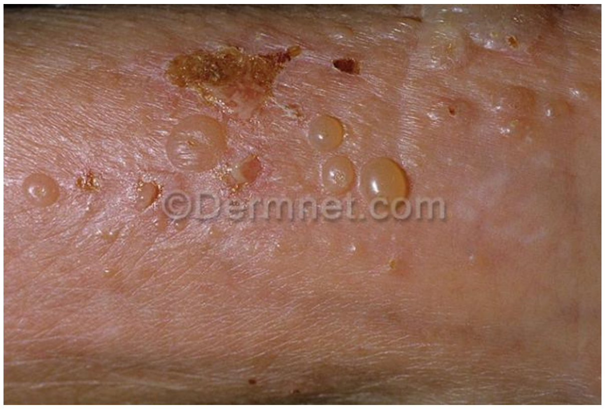
SkinGPT-4	Dermatology Physician
Q1. Could you describe the skin disease in this image for me?<br><br>A1. This could be a skin disease image of bullous pemphigoid.<br><br>Q2. You should write a paragraph to list more features you observed in the image.<br><br>A2. This image shows a skin disease bullous pemphigoid. The disease is characterized by large fluid filled blisters that often affect the skin on the legs, feet, and hands. The blisters are usually flat and round, and they often rupture leaving behind raw areas that can be prone to infection. The blisters are caused by a deficiency in a protein called BP230, which is necessary for the integrity of the skin's basement membrane. Bullous pemphigoid is a rare, autoimmune disorder that affects the skin and mucous membranes. It is more common in older adults and people with weakened immune systems. It can be treated with corticosteroids, immunosuppressive drugs, and plasma therapy.<br><br>Q3. With previous information, please write a paragraph to explain in detail the cause of this skin disease.<br><br>A3. The cause of this skin disease is likely to be a phototoxic reaction, which is a type of contact dermatitis caused by substances that are photosensitive and activated by sunlight. These substances can be found in various topical products such as sunscreens, moisturizers, and	Q1. Could you describe the skin disease in this image for me?<br><br>A1. Bullous disease, Bullous pemphigoid<br><br>Q2. You should write a paragraph to list more features you observed in the image.<br><br>A2. Generalized tense bullae and crusted erosions.<br><br>Q3. With previous information, please write a paragraph to explain in detail the cause of this skin disease.<br><br>A3. Bullous pemphigoid (BP) is the most common autoimmune subepidermal blistering disease; it predominantly affects the elderly. It is usually a chronic disease, with spontaneous exacerbations and remissions. BP is associated with tissue-bound and circulating autoantibodies directed against BP antigen 180 (BP180, BPAG2 or type XVII collagen) and BP antigen 230 (BP230 or BPAG1e), components of junctional adhesion complexes called hemidesmosomes that promote dermal–epidermal cohesion. Diagnosis relies on immunopathologic examinations, particularly direct and indirect immunofluorescence microscopy as well as anti-BP180/BP230 ELISAs.<br><br>Q4. How should I treat it? What medicine should I use?<br><br>A4. Systemic corticosteroids represent the most endorsed treatment. While the optimal duration of

fragrances. The reaction typically presents as a sunburn reaction followed by a red, itchy rash that can blister and leave hyperpigmentation or hypopigmentation. The lesion in the image is likely a bullous pemphigoid, which is a blistering skin disease that can occur as an autoimmune disorder or as a side effect of certain medications. It is characterized by the formation of large fluid-filled blisters on the skin and mucous membranes.<br><br>Q4. How should I treat it? What medicine should I use?<br><br>A4. The treatment of phototoxic reactions depends on the severity of the reaction. Mild reactions can be treated symptomatically with over-the-counter antihistamines, hydrocortisone, or calamine lotion. Severe reactions may require prescription medications, such as corticosteroids or antibiotics if there is an underlying bacterial infection. To prevent phototoxic reactions, avoid using products that contain photosensitive ingredients and use sunscreen with a high SPF to protect the skin from UV damage.<br>For bullous pemphigoid, treatment is aimed at controlling the immune system's attack on the skin and healing the blisters. Corticosteroids are the mainstay of treatment and can be administered topically or systemically. Other treatments include immunosuppressants, plasmapheresis, and immunoglobulin replacement therapy.	therapy has not been established, BP patients usually need to be treated for approximately 12–18 months. Dupilumab Injection also a safe and effective medication.
Evaluation of Physician	
Evaluation Item	Score (1-5, the higher the better)
SkinGPT-4's diagnosis is correct or relevant.	5
SkinGPT-4's description is informative.	2
SkinGPT-4's suggestions are useful.	2
SkinGPT-4 can help doctors with diagnosis.	3
SkinGPT-4 can help patients to understand their disease better.	3
If SkinGPT-4 can be deployed locally, it protects patients' privacy.	5

Willingness to use SkinGPT-4	3

Case bullous disease 2

Image ID: bullous disease2	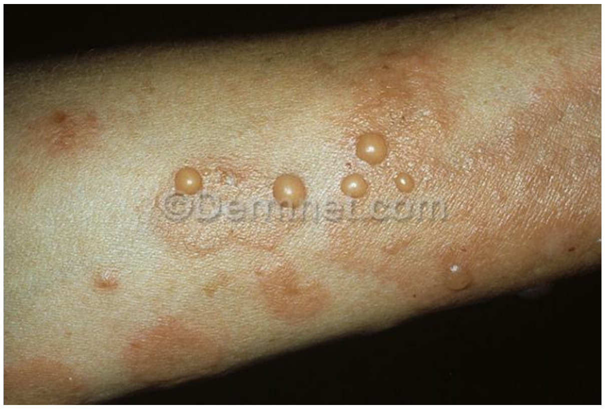
**SkinGPT-4**	**Dermatology Physician**
Q1. Could you describe the skin disease in this image for me?<br><br>A1. This could be a skin disease image of bullous pemphigoid.<br><br>Q2. You should write a paragraph to list more features you observed in the image.<br><br>A2. The image shows a skin disease bullous pemphigoid. The disease is characterized by large fluid filled blisters that occur on the skin and mucous membranes. The blisters are usually more pronounced on the trunk of the body and can be accompanied by mucous membrane involvement. The disease is caused by deficiency of a protein called bullous pemphigoid antigen. It is more common in elderly people and can be life threatening.<br><br>Q3. With previous information, please write a paragraph to explain in detail the cause of this skin disease.<br><br>A3. Bullous pemphigoid is a rare autoimmune disorder that causes large, fluid filled blisters on the skin and mucous membranes. The blisters are formed as a result of an abnormal immune response that causes the immune system to attack the skin and mucous membranes. The cause of bullous pemphigoid is not fully understood, but it is thought to be associated with a deficiency of a protein called bullous pemphigoid antigen. This deficiency causes the immune system to attack the skin and mucous	Q1. Could you describe the skin disease in this image for me?<br><br>A1. Bullous disease, Bullous pemphigoid<br><br>Q2. You should write a paragraph to list more features you observed in the image.<br><br>A2. Generalized tense bullae and erythema.<br><br>Q3. With previous information, please write a paragraph to explain in detail the cause of this skin disease.<br><br>A3. Bullous pemphigoid (BP) is the most common autoimmune subepidermal blistering disease; it predominantly affects the elderly. It is usually a chronic disease, with spontaneous exacerbations and remissions. BP is associated with tissue-bound and circulating autoantibodies directed against BP antigen 180 (BP180, BPAG2 or type XVII collagen) and BP antigen 230 (BP230 or BPAG1e), components of junctional adhesion complexes called hemidesmosomes that promote dermal–epidermal cohesion. Diagnosis relies on immunopathologic examinations, particularly direct and indirect immunofluorescence microscopy as well as anti-BP180/BP230 ELISAs.<br><br>Q4. How should I treat it? What medicine should I use?<br><br>A4. Systemic corticosteroids represent the most endorsed treatment. While the optimal duration of

membranes, leading to the formation of blisters. Bullous pemphigoid is more common in elderly people and can be life threatening if not treated properly.<br><br>Q4. How should I treat it? What medicine should I use?<br><br>A4. The treatment of bullous pemphigoid depends on the severity of the disease and the patient's overall health. The main goal of treatment is to control the immune response that is causing the blisters. Corticosteroids are the most commonly used medication to treat bullous pemphigoid. They help to reduce inflammation and suppress the immune system. Other medications that may be used include immunosuppressive drugs and enzymes that help to break down the protein that is causing the immune response. In severe cases, a plasma transfusion may be necessary. It is important to consult with a dermatologist for proper diagnosis and treatment.	therapy has not been established, BP patients usually need to be treated for approximately 12–18 months. Dupilumab Injection also a safe and effective medication.
Evaluation of Physician	
Evaluation Item	Score (1-5, the higher the better)
SkinGPT-4's diagnosis is correct or relevant.	5
SkinGPT-4's description is informative.	5
SkinGPT-4's suggestions are useful.	5
SkinGPT-4 can help doctors with diagnosis.	5
SkinGPT-4 can help patients to understand their disease better.	5
If SkinGPT-4 can be deployed locally, it protects patients' privacy.	5
Willingness to use SkinGPT-4	5

Case bullous disease 3

Image ID: bullous disease3	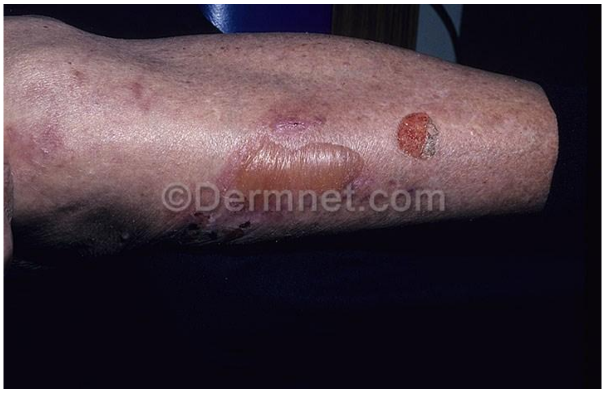
SkinGPT-4	Dermatology Physician
Q1. Could you describe the skin disease in this image for me?<br><br>A1. bullous disease<br><br>Q2. You should write a paragraph to list more features you observed in the image.<br><br>A2. In the image, the patient has a large, fluid - filled blister on their lower leg. The blister is filled with clear fluid and has a distinct border. The surrounding skin appears healthy and there are no other lesions or signs of inflammation.<br><br>Q3. With previous information, please write a paragraph to explain in detail the cause of this skin disease.<br><br>A3. The most likely cause of this skin disease is bullous pemphigoid, a rare autoimmune disorder that causes the body's immune system to attack the epidermis and cause blisters to form on the skin. It is characterized by the formation of large, fluid - filled blisters that are often accompanied by mild itching and burning sensations. It typically affects middle - aged adults and is more common in people with compromised immune systems, such as those with autoimmune disorders or those taking immunosuppressive medications. The exact cause of bullous pemphigoid is not fully understood, but it is believed to be related to the presence of autoantibodies that target the basement membrane zone of the epidermis.<br><br>Q4. How should I treat it? What medicine should I	Q1. Could you describe the skin disease in this image for me?<br><br>A1. Bullous disease, Bullous pemphigoid<br><br>Q2. You should write a paragraph to list more features you observed in the image.<br><br>A2. Tense bullae and erosion on leg.<br><br>Q3. With previous information, please write a paragraph to explain in detail the cause of this skin disease.<br><br>A3. Bullous pemphigoid (BP) is the most common autoimmune subepidermal blistering disease; it predominantly affects the elderly. It is usually a chronic disease, with spontaneous exacerbations and remissions. BP is associated with tissue-bound and circulating autoantibodies directed against BP antigen 180 (BP180, BPAG2 or type XVII collagen) and BP antigen 230 (BP230 or BPAG1e), components of junctional adhesion complexes called hemidesmosomes that promote dermal–epidermal cohesion. Diagnosis relies on immunopathologic examinations, particularly direct and indirect immunofluorescence microscopy as well as anti-BP180/BP230 ELISAs.<br><br>Q4. How should I treat it? What medicine should I use?<br><br>A4. Systemic corticosteroids represent the most endorsed treatment. While the optimal duration of therapy has not been established, BP patients

use?<br><br>A4. Treatment for bullous pemphigoid typically involves the use of corticosteroids, either topical or oral, to reduce inflammation and suppress the immune system. In severe cases, immunosuppressive medications such as methotrexate or mycophenolate mofetil may be used. Additionally, moisture - retaining dressings and topical anesthetics may be used to manage symptoms such as itching and pain. In cases where the patient has an underlying autoimmune disorder, treatment of the underlying condition may be necessary. In all cases, the treatment plan should be developed in consultation with a dermatologist or other healthcare provider.	usually need to be treated for approximately 12–18 months. Dupilumab Injection also a safe and effective medication.

Evaluation of Physician	
Evaluation Item	Score (1-5, the higher the better)
SkinGPT-4's diagnosis is correct or relevant.	5
SkinGPT-4's description is informative.	5
SkinGPT-4's suggestions are useful.	5
SkinGPT-4 can help doctors with diagnosis.	5
SkinGPT-4 can help patients to understand their disease better.	5
If SkinGPT-4 can be deployed locally, it protects patients' privacy.	5
Willingness to use SkinGPT-4	5

Case bullous disease 4

Image ID: bullous disease4	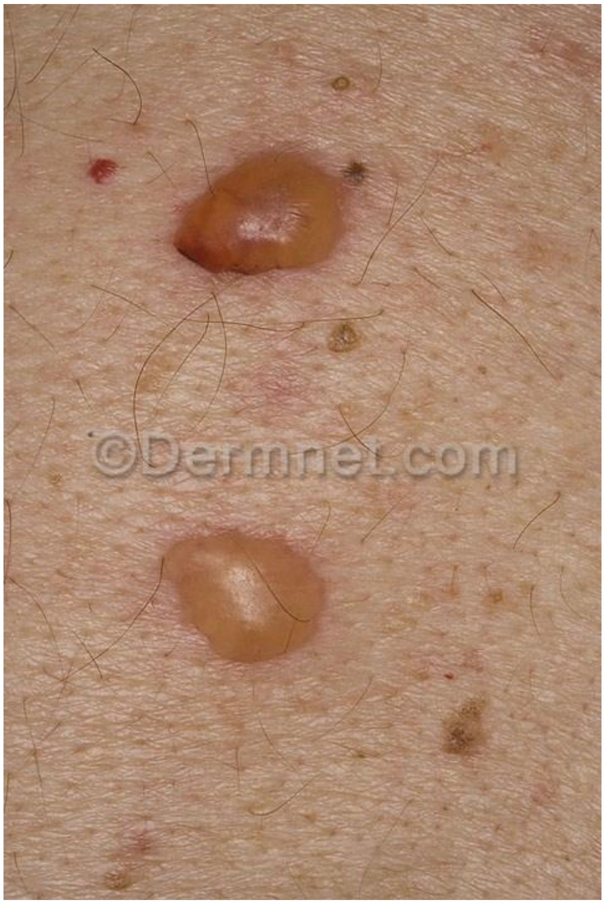
SkinGPT-4	Dermatology Physician
Q1. Could you describe the skin disease in this image for me?<br><br>A1. Bullous disease<br><br>Q2. You should write a paragraph to list more features you observed in the image.<br><br>A2. The image shows a lesion with a bullous pattern, characterized by the presence of large, fluid-filled blisters on the surface of the skin. The blisters are surrounded by red, inflamed tissue and there is an accumulation of white blood cells, known as neutrophils, in the surrounding dermis. The epidermis, the outermost layer of skin, is separated from the dermis by the blisters. This type of lesion is often associated with autoimmune disorders, such as pemphigus and bullous pemphigoid, as well as with certain types of	Q1. Could you describe the skin disease in this image for me?<br><br>A1. Bullous disease, Bullous pemphigoid<br><br>Q2. You should write a paragraph to list more features you observed in the image.<br><br>A2. Tense bullae<br><br>Q3. With previous information, please write a paragraph to explain in detail the cause of this skin disease.<br><br>A3.<br>Bullous pemphigoid (BP) is the most common autoimmune subepidermal blistering disease; it predominantly affects the elderly. It is usually a chronic disease, with spontaneous exacerbations

cancer and medications. The exact diagnosis can only be made by a dermatologist after a biopsy of the lesion.<br><br>Q3. With previous information, please write a paragraph to explain in detail the cause of this skin disease.<br><br>A3. This skin disease is caused by a variety of factors, including genetics, environmental factors, and autoimmune disorders. It can also be caused by viral or bacterial infections, and certain medications. In some cases, the exact cause may not be known. Autoimmune disorders, such as pemphigus and bullous pemphigoid, are characterized by the production of antibodies that attack the skin and cause blistering. In other cases, the disease may be caused by a viral or bacterial infection, or a reaction to certain medications. The exact cause of this lesion cannot be determined without a biopsy and examination by a dermatologist.<br><br>Q4. How should I treat it? What medicine should I use?<br><br>A4. The treatment for this skin disease will depend on the specific diagnosis and the severity of the condition. In general, treatment may include topical medications, such as corticosteroids and antibiotics, to reduce inflammation and prevent infection. Oral medications, such as immunosuppressants, may also be prescribed to suppress the immune system and prevent further damage to the skin. In severe cases, hospitalization and intravenous medications may be necessary. The best treatment plan will be determined by a dermatologist or other healthcare professional after a thorough examination and diagnosis.<br>Please note that the above information is not a substitute for medical advice from a qualified healthcare professional. If you suspect that you may have a skin disease, you should consult with a dermatologist or other healthcare professional for an accurate diagnosis and appropriate treatment.	and remissions. BP is associated with tissue-bound and circulating autoantibodies directed against BP antigen 180 (BP180, BPAG2 or type XVII collagen) and BP antigen 230 (BP230 or BPAG1e), components of junctional adhesion complexes called hemidesmosomes that promote dermal–epidermal cohesion. Diagnosis relies on immunopathologic examinations, particularly direct and indirect immunofluorescence microscopy as well as anti-BP180/BP230 ELISAs.<br><br>Q4. How should I treat it? What medicine should I use?<br><br>A4. Systemic corticosteroids represent the most endorsed treatment. While the optimal duration of therapy has not been established, BP patients usually need to be treated for approximately 12–18 months. Dupilumab Injection also a safe and effective medication.
Evaluation of Physician	
Evaluation Item	Score (1-5, the higher the better)

SkinGPT-4's diagnosis is correct or relevant.	5
SkinGPT-4's description is informative.	5
SkinGPT-4's suggestions are useful.	5
SkinGPT-4 can help doctors with diagnosis.	5
SkinGPT-4 can help patients to understand their disease better.	5
If SkinGPT-4 can be deployed locally, it protects patients' privacy.	5
Willingness to use SkinGPT-4	5

Case bullous disease 5

Image ID: bullous disease5	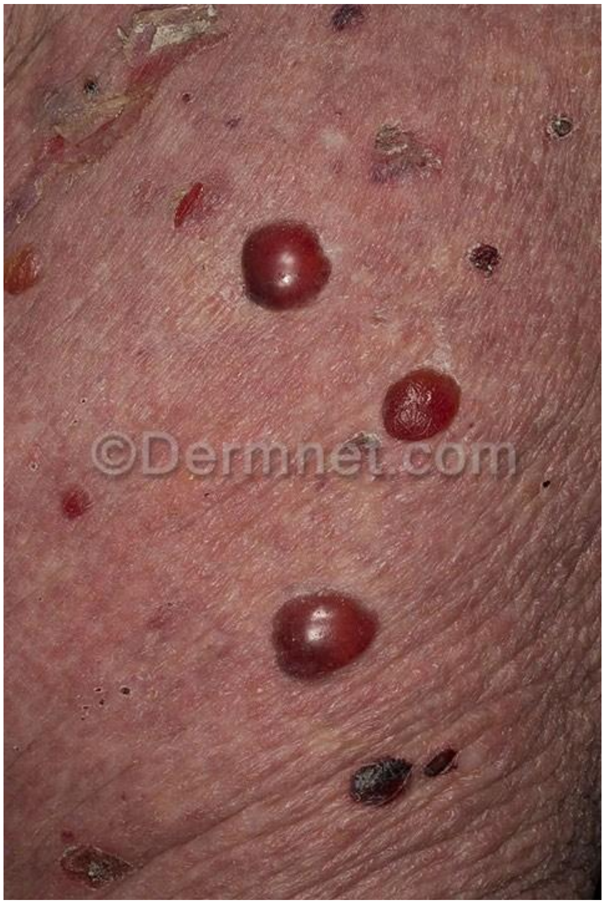
SkinGPT-4	Dermatology Physician
Q1. Could you describe the skin disease in this image for me?<br><br>A1. This could be a skin disease image of bullous pemphigoid.<br><br>Q2. You should write a paragraph to list more features you observed in the image.<br><br>A2. The image shows several unique features. The most prominent feature is the presence of bullous pemphigoid, a rare autoimmune disorder that causes large fluid filled blisters on the skin and mucous membranes. The blisters are usually most prominent on the skin, and can be found on the arms, legs, and trunk. In this image, the blisters are present on the genital area. Another feature observed in this image is the presence of bullous pemphigoid antibodies, which are involved	Q1. Could you describe the skin disease in this image for me?<br><br>A1. Bullous disease, Bullous pemphigoid<br><br>Q2. You should write a paragraph to list more features you observed in the image.<br><br>A2. Tension blister with blood in the contents. Crusted erosions.<br><br>Q3. With previous information, please write a paragraph to explain in detail the cause of this skin disease.<br><br>A3. Bullous pemphigoid (BP) is the most common autoimmune subepidermal blistering disease; it predominantly affects the elderly. It is usually a chronic disease, with spontaneous exacerbations

in the pathogenesis of the disorder. These antibodies are detected in the fluid of the blisters and can be used to diagnose the condition. A third feature observed in this image is the presence of uraemia, which is characterized by the presence of blood in the urine. Uraemia is a complication of chronic kidney disease, which is a common concurrent disease in patients with bullous pemphigoid. Finally, the image also shows the presence of ulceration, which is a common complication of bullous pemphigoid. Ulceration can occur on the skin, mucous membranes, and cornea, and can be caused by bacterial or fungal infections.<br><br>Q3. With previous information, please write a paragraph to explain in detail the cause of this skin disease.<br><br>A3. Bullous pemphigoid is a rare autoimmune disorder that causes large fluid filled blisters on the skin and mucous membranes. The blisters are usually most prominent on the skin, and can be found on the arms, legs, and trunk. The cause of bullous pemphigoid is not well understood, but it is thought to be triggered by an abnormal immune response that leads to the production of antibodies that attack the skin and mucous membranes. These antibodies cause damage to the basement membrane zone, which separates the epidermis from the dermis, leading to the formation of blisters. The blisters are usually flat and are filled with a clear fluid. Bullous pemphigoid can also affect other parts of the body, including the eyes, mouth, and genitals, and can cause serious complications such as infection, scarring, and even death. Treatment for bullous pemphigoid includes corticosteroids, immunosuppressants, and other medications to manage symptoms and prevent complications. A healthy lifestyle, including avoiding exposure to sun and stress, can also help in managing the disease.<br><br>Q4. How should I treat it? What medicine should I use?<br><br>A4. The treatment for bullous pemphigoid typically involves a combination of medications to manage symptoms and prevent complications. The main goals of treatment are to reduce the formation of new blisters and to promote healing of existing	and remissions. BP is associated with tissue-bound and circulating autoantibodies directed against BP antigen 180 (BP180, BPAG2 or type XVII collagen) and BP antigen 230 (BP230 or BPAG1e), components of junctional adhesion complexes called hemidesmosomes that promote dermal–epidermal cohesion. Diagnosis relies on immunopathologic examinations, particularly direct and indirect immunofluorescence microscopy as well as anti-BP180/BP230 ELISAs.<br><br>Q4. How should I treat it? What medicine should I use?<br><br>A4. Systemic corticosteroids represent the most endorsed treatment. While the optimal duration of therapy has not been established, BP patients usually need to be treated for approximately 12–18 months. Dupilumab Injection also a safe and effective medication.

blisters.<br>Corticosteroids are the mainstay of treatment for bullous pemphigoid. They work by reducing inflammation and suppressing the immune system, which helps to reduce the formation of new blisters. Steroids can be applied directly to the skin or taken orally.<br>Immunosuppressants such as azathioprine or mycophenolate mofetil can also be used to suppress the immune system and reduce inflammation.<br>Antibiotics may be prescribed if there is an underlying bacterial infection, which can sometimes occur in bullous pemphigoid.<br>Pain management is also an important aspect of treatment, and may involve over-the-counter pain medications, such as acetaminophen or ibuprofen.	
Evaluation of Physician	
Evaluation Item	Score (1-5, the higher the better)
SkinGPT-4's diagnosis is correct or relevant.	5
SkinGPT-4's description is informative.	5
SkinGPT-4's suggestions are useful.	5
SkinGPT-4 can help doctors with diagnosis.	5
SkinGPT-4 can help patients to understand their disease better.	5
If SkinGPT-4 can be deployed locally, it protects patients' privacy.	5
Willingness to use SkinGPT-4	5

Case bullous disease 6

<table>
<tr><td>Image ID: bullous disease6</td><td>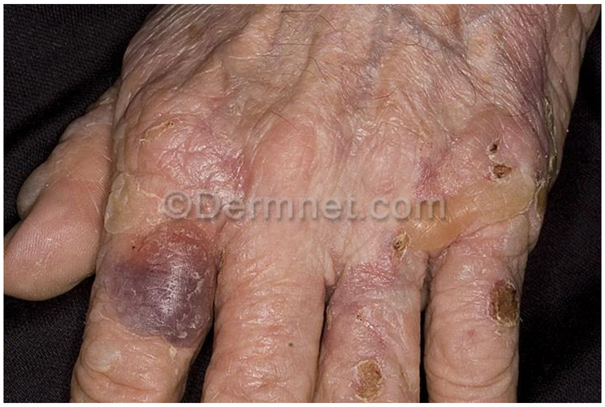
</td></tr>
<tr><th>SkinGPT-4</th><th>Dermatology Physician</th></tr>
<tr><td>Q1. Could you describe the skin disease in this image for me?

A1. This could be a skin disease image of bullous pemphigoid.

Q2. You should write a paragraph to list more features you observed in the image.

A2. The image provided is of bullous pemphigoid, a subepidermal blistering disorder that commonly affects older adults. The lesions are typically located on the trunk and lower extremities and can be painful. In this image, the eruption is seen on the hand and is characterized by tense, clear, fluid filled blisters on an erythematous base. The blisters are separated from the epidermis by a thick floor and are often accompanied by mucous membrane involvement.

Q3. With previous information, please write a paragraph to explain in detail the cause of this skin disease.

A3. Bullous pemphigoid is a rare, autoimmune, subepidermal blistering disorder that typically affects older adults. The pathogenesis of bullous pemphigoid is not fully understood, but it is thought to be mediated by autoantibodies directed against desmoglein 3, a transmembrane protein found in the basal layer of the epidermis. These autoantibodies cause subepidermal blistering by disrupting the interaction between desmoglein 3 and keratin 10, resulting in separation of the epidermis from the dermis. The blisters are</td><td>Q1. Could you describe the skin disease in this image for me?

A1. Bullous disease, Bullous pemphigoid

Q2. You should write a paragraph to list more features you observed in the image.

A2. Tension blister with blood in the contents. Crusted erosions.

Q3. With previous information, please write a paragraph to explain in detail the cause of this skin disease.

A3. Bullous pemphigoid (BP) is the most common autoimmune subepidermal blistering disease; it predominantly affects the elderly. It is usually a chronic disease, with spontaneous exacerbations and remissions. BP is associated with tissue-bound and circulating autoantibodies directed against BP antigen 180 (BP180, BPAG2 or type XVII collagen) and BP antigen 230 (BP230 or BPAG1e), components of junctional adhesion complexes called hemidesmosomes that promote dermal–epidermal cohesion. Diagnosis relies on immunopathologic examinations, particularly direct and indirect immunofluorescence microscopy as well as anti-BP180/BP230 ELISAs.

Q4. How should I treat it? What medicine should I use?

A4. Systemic corticosteroids represent the most</td></tr>
</table>

typically located on the trunk and lower extremities and can be painful. Mucous membrane involvement is also common in bullous pemphigoid. Treatment options include corticosteroids, immunomodulatory drugs, and plasma exchange.<br><br>Q4. How should I treat it? What medicine should I use?<br><br>A4. The treatment of bullous pemphigoid depends on the severity and extent of the disease. Corticosteroids are the mainstay of treatment and are used to reduce inflammation and suppress the immune system. Other treatments include immunomodulatory drugs such as rituximab and mycophenolate mofetil, and plasma exchange. In addition, management of any underlying medical conditions such as diabetes and hypertension is important. Topical treatments such as lidocaine and diazoxide may be used for pain and itching. In severe cases, supportive care may be required, including fluid and electrolyte replacement, blood transfusions, and antibiotics for infected wounds.<br><br>Please note that the information provided is for general educational purposes only and is not a substitute for professional medical advice, diagnosis, or treatment. Always seek the advice of a qualified healthcare provider for any questions you may have regarding a medical condition.	endorsed treatment. While the optimal duration of therapy has not been established, BP patients usually need to be treated for approximately 12–18 months. Dupilumab Injection also a safe and effective medication.

Evaluation of Physician	
Evaluation Item	Score (1-5, the higher the better)
SkinGPT-4's diagnosis is correct or relevant.	5
SkinGPT-4's description is informative.	4
SkinGPT-4's suggestions are useful.	5
SkinGPT-4 can help doctors with diagnosis.	5
SkinGPT-4 can help patients to understand their disease better.	5
If SkinGPT-4 can be deployed locally, it protects patients' privacy.	5

Willingness to use SkinGPT-4	4

Case bullous disease 7

Image ID: bullous disease7	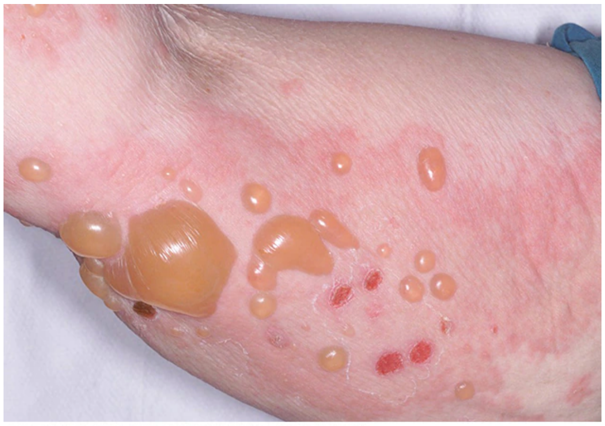<br>© MAYO FOUNDATION FOR MEDICAL EDUCATION AND RESEARCH. ALL RIGHTS RESERVED.
SkinGPT-4	Dermatology Physician
Q1. Could you describe the skin disease in this image for me?<br><br>A1. bullous disease<br><br>Q2. You should write a paragraph to list more features you observed in the image.<br><br>A2. The patient has bullous disease, which is characterized by the presence of large, fluid - filled blisters on the skin or mucous membranes. This is caused by a defect in the immune system that prevents the skin from properly protecting itself from harmful substances. In addition to the characteristic blisters, patients with bullous disease often have dry, rough, or scaly skin and may experience itching or pain. The condition can be inherited or acquired, and treatment includes the use of topical medications, light therapy, or systemic medications depending on the severity of symptoms.<br><br>Q3. With previous information, please write a paragraph to explain in detail the cause of this skin disease.<br><br>A3. The cause of bullous disease is not fully understood, but it is thought to be related to a defect in the immune system that prevents the skin from properly protecting itself from harmful substances. This can be inherited or acquired through exposure to certain substances or conditions. Inherited forms of bullous disease are caused by mutations in genes that regulate the immune system, while acquired forms may be	Q1. Could you describe the skin disease in this image for me?<br><br>A1. Bullous disease, Bullous pemphigoid<br><br>Q2. You should write a paragraph to list more features you observed in the image.<br><br>A2. Generalized tense bullae, crusted erosions and erythema.<br><br>Q3. With previous information, please write a paragraph to explain in detail the cause of this skin disease.<br><br>A3. Bullous pemphigoid (BP) is the most common autoimmune subepidermal blistering disease; it predominantly affects the elderly. It is usually a chronic disease, with spontaneous exacerbations and remissions. BP is associated with tissue-bound and circulating autoantibodies directed against BP antigen 180 (BP180, BPAG2 or type XVII collagen) and BP antigen 230 (BP230 or BPAG1e), components of junctional adhesion complexes called hemidesmosomes that promote dermal–epidermal cohesion. Diagnosis relies on immunopathologic examinations, particularly direct and indirect immunofluorescence microscopy as well as anti-BP180/BP230 ELISAs.<br><br>Q4. How should I treat it? What medicine should I use?<br><br>A4. Systemic corticosteroids represent the most

caused by exposure to chemicals, infections, or other irritants. In general, bullous disease occurs when the body produces too much of a protein called Type 7 collagen, which causes the formation of large, fluid - filled blisters on the skin or mucous membranes. The condition can also be triggered by certain medications or medical conditions, such as cancer or HIV infection.<br><br>Q4. How should I treat it? What medicine should I use?<br><br>A4. Treatment for bullous disease depends on the severity of symptoms and the underlying cause of the condition. In general, treatment may include the use of topical medications, light therapy, or systemic medications.<br>Topical medications: These medications are applied directly to the skin and are used to reduce itching, redness, and the formation of blisters. Examples of topical medications used to treat bullous disease include corticosteroids, which reduce inflammation, and moisturizers, which help to keep the skin hydrated.<br>Light therapy: This type of therapy uses ultraviolet light to suppress the immune system and reduce the production of Type 7 collagen, which causes the formation of blisters. This treatment is usually reserved for patients with severe cases of bullous disease that do not respond to other treatments.<br>Systemic medications: These medications are taken by mouth or injection and are used to treat more severe cases of bullous disease. Examples of systemic medications used to treat bullous disease include immunosuppressive drugs, which reduce the activity of the immune system, and biologic agents, which target specific components of the immune system.<br>It’s important to consult with a dermatologist or other healthcare provider for a proper diagnosis and treatment plan.	endorsed treatment. While the optimal duration of therapy has not been established, BP patients usually need to be treated for approximately 12–18 months. Dupilumab Injection also a safe and effective medication.
Evaluation of Physician	
Evaluation Item	Score (1-5, the higher the better)
SkinGPT-4’s diagnosis is correct or relevant.	5
SkinGPT-4’s description is informative.	4

SkinGPT-4's suggestions are useful.	5
SkinGPT-4 can help doctors with diagnosis.	5
SkinGPT-4 can help patients to understand their disease better.	5
If SkinGPT-4 can be deployed locally, it protects patients' privacy.	5
Willingness to use SkinGPT-4	5

Case bullous disease 8

Image ID: bullous disease8	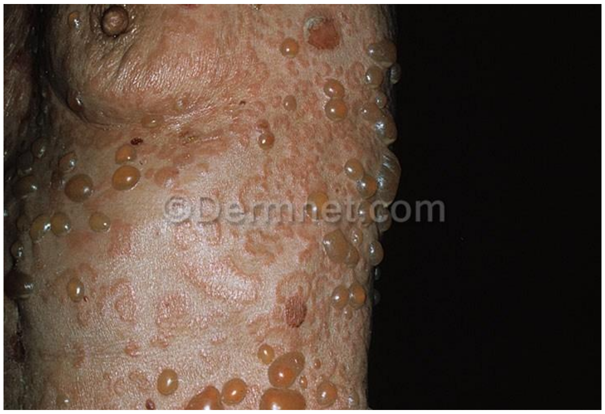
**SkinGPT-4**	**Dermatology Physician**
Q1. Could you describe the skin disease in this image for me?<br><br>A1. This could be a skin disease image of bullous pemphigoid.<br><br>Q2. You should write a paragraph to list more features you observed in the image.<br><br>A2. In this image, we can see a skin disease called bullous pemphigoid. It is a blistering disease that affects the skin and mucous membranes. The blisters are usually large and thin-walled, and they typically occur on the skin that is exposed to the sun, such as the face, hands, and arms. In this image, we can see a person with bullous pemphigoid affected skin. It is a chronic autoimmune disorder that causes large, fluid-filled blisters on the skin. The blisters are most commonly found on the skin that is exposed to the sun, such as the face, hands, and arms. The blisters usually occur in clusters and can be very itchy and painful. The disease can also affect the mucous membranes, such as the mouth and nose, causing ulcers and blisters. Bullous pemphigoid is more common in older people and in people with weakened immune systems. It can be treated with corticosteroids and other immunosuppressive drugs.<br><br>Q3. With previous information, please write a paragraph to explain in detail the cause of this skin disease.	Q1. Could you describe the skin disease in this image for me?<br><br>A1. Bullous disease, Bullous pemphigoid<br><br>Q2. You should write a paragraph to list more features you observed in the image.<br><br>A2. Generalized tense bullae and crusted erosions.<br><br>Q3. With previous information, please write a paragraph to explain in detail the cause of this skin disease.<br><br>A3. Bullous pemphigoid (BP) is the most common autoimmune subepidermal blistering disease; it predominantly affects the elderly. It is usually a chronic disease, with spontaneous exacerbations and remissions. BP is associated with tissue-bound and circulating autoantibodies directed against BP antigen 180 (BP180, BPAG2 or type XVII collagen) and BP antigen 230 (BP230 or BPAG1e), components of junctional adhesion complexes called hemidesmosomes that promote dermal–epidermal cohesion. Diagnosis relies on immunopathologic examinations, particularly direct and indirect immunofluorescence microscopy as well as anti-BP180/BP230 ELISAs.<br><br>Q4. How should I treat it? What medicine should I use?

A3. Bullous pemphigoid is a chronic autoimmune disorder that is characterized by the formation of large, fluid-filled blisters on the skin. The blisters are most commonly found on the skin that is exposed to the sun, such as the face, hands, and arms. The cause of bullous pemphigoid is not well understood, but it is thought to be mediated by autoantibodies that target components of the basement membrane zone, which is the layer of skin that separates the epidermis from the dermis. These autoantibodies cause the immune system to attack the basement membrane zone, leading to blister formation and damage to the skin and other tissues. Bullous pemphigoid can also affect the mucous membranes, such as the mouth and nose, causing ulcers and blisters. It is more common in older people and in people with weakened immune systems. There is no cure for bullous pemphigoid, but it can be treated with corticosteroids and other immunosuppressive drugs to manage symptoms and prevent complications.<br><br>Q4. How should I treat it? What medicine should I use?<br><br>A4. Bullous pemphigoid is a chronic autoimmune disorder that is characterized by the formation of large, fluid-filled blisters on the skin. The blisters are most commonly found on the skin that is exposed to the sun, such as the face, hands, and arms. The cause of bullous pemphigoid is not well understood, but it is thought to be mediated by autoantibodies that target components of the basement membrane zone, which is the layer of skin that separates the epidermis from the dermis. These autoantibodies cause the immune system to attack the basement membrane zone, leading to blister formation and damage to the skin and other tissues. Bullous pemphigoid can also affect the mucous membranes, such as the mouth and nose, causing ulcers and blisters. It is more common in older people and in people with weakened immune systems. There is no cure for bullous pemphigoid, but it can be treated with corticosteroids and other immunosuppressive drugs to manage symptoms and prevent complications.<br>How should I treat it? What medicine should I use?<br>Bullous pemphigoid is usually treated with corticosteroids and other immunosuppressive	A4. Systemic corticosteroids represent the most endorsed treatment. While the optimal duration of therapy has not been established, BP patients usually need to be treated for approximately 12–18 months. Dupilumab Injection also a safe and effective medication.

drugs to manage symptoms and prevent complications. The goal of treatment is to reduce blistering, reduce inflammation, and prevent scarring.<br>Corticosteroids: These medications help to reduce inflammation and suppress the immune system. They are usually prescribed in the form of creams, ointments, or oral medications.<br>Immunosuppressive drugs: These medications help to suppress the immune system and reduce blistering. Examples include azathioprine, mycophenolate mofetil, and rituximab.<br>Pain relief: Pain can be managed with over-the-counter pain relievers such as acetaminophen or ibuprofen.<br>Wound care: Any wounds or ulcers should be cleaned and dressed to prevent infection.<br>Protection from sun: Sun exposure can worsen the condition, so sun protection measures should be taken.<br>It is important to note that treatment may vary depending on the severity of the disease, and a dermatologist should be consulted for the proper treatment plan.	
Evaluation of Physician	
Evaluation Item	Score (1-5, the higher the better)
SkinGPT-4's diagnosis is correct or relevant.	5
SkinGPT-4's description is informative.	4
SkinGPT-4's suggestions are useful.	5
SkinGPT-4 can help doctors with diagnosis.	5
SkinGPT-4 can help patients to understand their disease better.	5
If SkinGPT-4 can be deployed locally, it protects patients' privacy.	5
Willingness to use SkinGPT-4	5

Case bullous disease 9

Image ID: bullous disease9	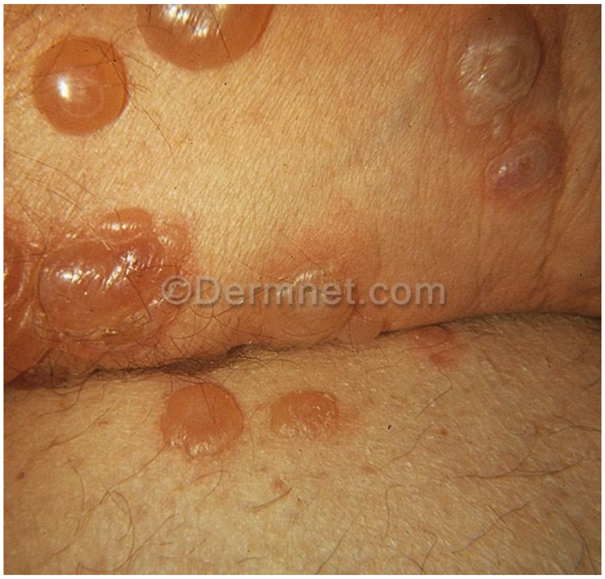
**SkinGPT-4**	**Dermatology Physician**
Q1. Could you describe the skin disease in this image for me?<br><br>A1. This could be a skin disease image of bullous pemphigoid.<br><br>Q2. You should write a paragraph to list more features you observed in the image.<br><br>A2. This image is a skin disease image of bullous pemphigoid. The disease is characterized by large fluid filled blisters that typically occur on the skin and mucous membranes. The blisters are usually most noticeable on the skin of the hands, feet, and genital area. In this image, the blisters are present on the genital area which is a common site for bullous pemphigoid. The blisters are flat and round and have a clear fluid. The surrounding skin is usually normal in color and texture. The disease can be mild, moderate or severe. In severe cases, the blisters can be large and numerous, and they can cover large areas of the skin. The disease can also be life threatening if the blisters involve the mucous membranes of the mouth, throat, or genital area.<br><br>Q3. With previous information, please write a paragraph to explain in detail the cause of this skin disease.	Q1. Could you describe the skin disease in this image for me?<br><br>A1. Bullous disease, Bullous pemphigoid<br><br>Q2. You should write a paragraph to list more features you observed in the image.<br><br>A2. Generalized tense bullae and crusted erosions.<br><br>Q3. With previous information, please write a paragraph to explain in detail the cause of this skin disease.<br><br>A3. Bullous pemphigoid (BP) is the most common autoimmune subepidermal blistering disease; it predominantly affects the elderly. It is usually a chronic disease, with spontaneous exacerbations and remissions. BP is associated with tissue-bound and circulating autoantibodies directed against BP antigen 180 (BP180, BPAG2 or type XVII collagen) and BP antigen 230 (BP230 or BPAG1e), components of junctional adhesion complexes called hemidesmosomes that promote dermal–epidermal cohesion. Diagnosis relies on immunopathologic examinations, particularly direct and indirect immunofluorescence microscopy as well as anti-BP180/BP230 ELISAs.

A3. Bullous pemphigoid is a autoimmune disorder that causes large, fluid filled blisters on the skin and mucous membranes. The cause of bullous pemphigoid is not well understood, but it is thought to be caused by an abnormal immune response that causes the immune system to attack healthy cells in the skin. This leads to the formation of blisters, which are filled with a clear fluid. Bullous pemphigoid can be triggered by certain medications, infections, and physical trauma, but in most cases, the cause is unknown. It is more common in older adults and in people with a weakened immune system. It can also be associated with other autoimmune diseases such as thyroid disorders, and celiac disease. In this case, it could be a mild form of the disease which is present on the genital area.<br><br>Q4. How should I treat it? What medicine should I use?<br><br>A4. The treatment of bullous pemphigoid depends on the severity of the disease. In mild cases, the blisters can be treated with topical medications such as hydrocortisone cream to reduce inflammation and pain. In more severe cases, oral medications may be needed to suppress the immune system and reduce inflammation. These medications include corticosteroids, such as prednisone, and immunosuppressive drugs, such as azathioprine or mycophenolate mofetil.<br>In addition, it is important to avoid exposure to sunlight and moisture, as both can worsen the symptoms of bullous pemphigoid. Wearing protective clothing and using a sunscreen with a high SPF can help to protect the skin from the sun.<br>It's important to note that treatment should be under the guidance of a dermatologist or other skin specialist, as they will be able to determine the best course of treatment based on the severity of the disease and the patient's overall health.	Q4. How should I treat it? What medicine should I use?<br><br>A4. Systemic corticosteroids represent the most endorsed treatment. While the optimal duration of therapy has not been established, BP patients usually need to be treated for approximately 12–18 months. Dupilumab Injection also a safe and effective medication.
Evaluation of Physician	
Evaluation Item	Score (1-5, the higher the better)
SkinGPT-4's diagnosis is correct or relevant.	5

SkinGPT-4's description is informative.	4
SkinGPT-4's suggestions are useful.	4
SkinGPT-4 can help doctors with diagnosis.	5
SkinGPT-4 can help patients to understand their disease better.	5
If SkinGPT-4 can be deployed locally, it protects patients' privacy.	5
Willingness to use SkinGPT-4	5

Case bullous disease 10

<table>
<tr><td>Image ID: bullous disease10</td><td>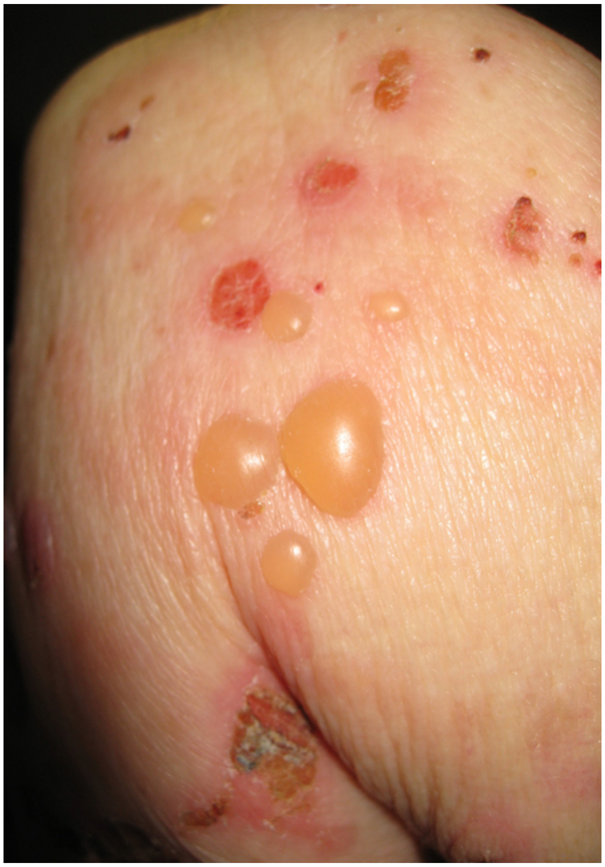</td></tr>
<tr><td>SkinGPT-4</td><td>Dermatology Physician</td></tr>
<tr><td>Q1. Could you describe the skin disease in this image for me?

A1. bullous disease

Q2. You should write a paragraph to list more features you observed in the image.

A2. lesions may be present and can be itchy and painful

Q3. With previous information, please write a paragraph to explain in detail the cause of this skin disease.

A3. Bullous disease is caused by a defect in the lysosome and the deposition of lipids and other substances in the skin and mucous membranes, leading to blistering and itching.</td><td>Q1. Could you describe the skin disease in this image for me?

A1. Bullous disease, Bullous pemphigoid

Q2. You should write a paragraph to list more features you observed in the image.

A2. Generalized tense bullae and crusted erosions.

Q3. With previous information, please write a paragraph to explain in detail the cause of this skin disease.

A3. Bullous pemphigoid (BP) is the most common autoimmune subepidermal blistering disease; it predominantly affects the elderly. It is usually a chronic disease, with spontaneous exacerbations and remissions. BP is associated with tissue-</td></tr>
</table>

Q4. How should I treat it? What medicine should I use?<br><br>A4. The treatment for bullous disease depends on the severity of symptoms and the underlying cause. A healthcare professional should be consulted for proper diagnosis and treatment. Medications such as topical steroids, antibiotics, and antihistamines may be prescribed to manage symptoms and prevent infection. In severe cases, systemic medications or surgery may be necessary.	bound and circulating autoantibodies directed against BP antigen 180 (BP180, BPAG2 or type XVII collagen) and BP antigen 230 (BP230 or BPAG1e), components of junctional adhesion complexes called hemidesmosomes that promote dermal–epidermal cohesion. Diagnosis relies on immunopathologic examinations, particularly direct and indirect immunofluorescence microscopy as well as anti-BP180/BP230 ELISAs.<br><br>Q4. How should I treat it? What medicine should I use?<br><br>A4. Systemic corticosteroids represent the most endorsed treatment. While the optimal duration of therapy has not been established, BP patients usually need to be treated for approximately 12–18 months. Dupilumab Injection also a safe and effective medication.
Evaluation of Physician	
Evaluation Item	Score (1-5, the higher the better)
SkinGPT-4's diagnosis is correct or relevant.	5
SkinGPT-4's description is informative.	3
SkinGPT-4's suggestions are useful.	2
SkinGPT-4 can help doctors with diagnosis.	4
SkinGPT-4 can help patients to understand their disease better.	4
If SkinGPT-4 can be deployed locally, it protects patients' privacy.	5
Willingness to use SkinGPT-4	5

Case Cellulitis 1

Image ID: Cellulitis1	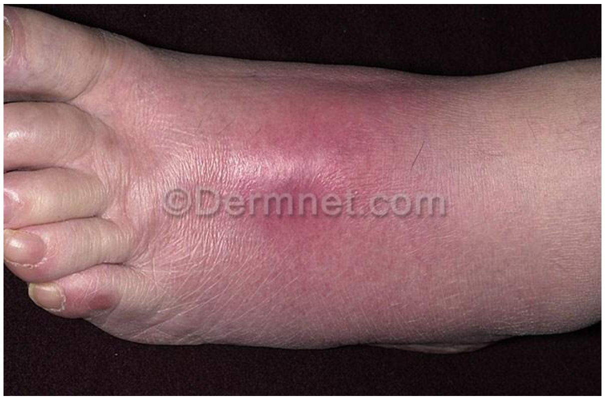
**SkinGPT-4**	**Dermatology Physician**
Q1. Could you describe the skin disease in this image for me?<br><br>A1. This could be a skin disease image of Cellulitis.<br><br>Q2. You should write a paragraph to list more features you observed in the image.<br><br>A2. The image shows a cellulitis, which is a bacterial infection of the skin that can occur due to breaks in the skin, such as from minor trauma, surgery, or insect bites. The infection causes inflammation and redness, and the skin may be painful or tender to the touch. The affected area may also have a thick, white superficial exudate that is referred to as whey cheese. The skin may also feel warm to the touch, and there may be regional lymphadenopathy. The wound is covered by a brownish gray slough, which is easily removed with minimal trauma. There is no abscess present. The lesion is now 3 cm in size and is located on the lateral aspect of the left calf, 2 cm below the midcalf level. The lesion is still quite erythematous and indurated, with minimal yellow-white fluid on top. No blisters are present. Rolling the lesion shows no redness. Ulceration is not present. The skin type is classified as type III. The photo was taken 11 days after the onset of symptoms, and the patient is a 43 year old woman who was diagnosed with cellulitis. No spread is seen in this photo. The lesion is well demarcated and there is no fuzzy margin.<br><br>Q3. With previous information, please write a	Q1. Could you describe the skin disease in this image for me?<br><br>A1. Skin infections; Cellulitis; Erysipelas.<br><br>Q2. You should write a paragraph to list more features you observed in the image.<br><br>A2. An Erythema on acrotarsium which borders is usually ill-defined.<br><br>Q3. With previous information, please write a paragraph to explain in detail the cause of this skin disease.<br><br>A3. Cellulitis is an infection of the deep dermis and subcutaneous tissue that manifests as areas of erythema, swelling, warmth and tenderness. Erysipelas is a superficial variant of cellulitis caused primarily by group A streptococci that affects the dermis with prominent lymphatic involvement; in contrast, classic cellulitis is centered in the deep dermis and subcutaneous tissues.<br><br>Q4. How should I treat it? What medicine should I use?<br><br>A4. Oral antibiotics to clear the infection and prevent it from spreading to other parts of the body are very important. For uncomplicated cases, a 10-day course of an oral antibiotic that covers these organisms (e.g. dicloxacillin, cephalexin or clindamycin) is appropriate.

paragraph to explain in detail the cause of this skin disease.<br><br>A3. The image shows a cellulitis, which is a bacterial infection of the skin that can occur due to breaks in the skin, such as from minor trauma, surgery, or insect bites. The infection causes inflammation and redness, and the skin may be painful or tender to the touch. The affected area may also have a thick, white superficial exudate that is referred to as whey cheese. The skin may also feel warm to the touch, and there may be regional lymphadenopathy. The wound is covered by a brownish gray slough, which is easily removed with minimal trauma. There is no abscess present. The lesion is now 3 cm in size and is located on the lateral aspect of the left calf, 2 cm below the midcalf level. The lesion is still quite erythematous and indurated, with minimal yellow-white fluid on top. No blisters are present. Rolling the lesion shows no redness. Ulceration is not present. The skin type is classified as type III. The photo was taken 11 days after the onset of symptoms, and the patient is a 43 year old woman who was diagnosed with cellulitis. No spread is seen in this photo. The lesion is well demarcated and there is no fuzzy margin. No<br>With previous information, please write a paragraph to explain in detail the cause of this skin disease.<br>The cause of cellulitis is an infection of the skin that occurs when bacteria penetrate the skin through breaks or damaged skin. This can happen due to minor trauma, surgery, or insect bites. The bacteria that typically cause cellulitis are Streptococcus species and Staphylococcus aureus. These bacteria infect the skin and underlying tissue, leading to inflammation, redness, pain, and swelling. The skin may also feel warm to the touch, and there may be regional lymphadenopathy. The body's immune system tries to fight the infection, which leads to the release of white blood cells and fluids into the affected area. This can cause the skin to become discolored and thickened, and may also result in the formation of pus. If left untreated, cellulitis can spread to other parts of the body, causing more serious complications such as sepsis and gangrene.<br>It is important to identify the underlying cause of the skin break and treat it promptly to prevent the occurrence of cellulitis. This may involve cleaning	

the wound, applying antibiotic ointment, and covering it with a clean, dry dressing. It is also important to maintain good hygiene and keep the affected area clean and dry to prevent further irritation.<br><br>Q4. How should I treat it? What medicine should I use?<br><br>A4. The treatment of cellulitis typically involves antibiotics to clear the infection, as well as symptomatic treatment to reduce inflammation, pain, and fever. The choice of antibiotic will depend on the severity and location of the infection, as well as the causative organism. In most cases, oral antibiotics such as penicillins or cephalosporins are prescribed. Topical treatments such as hydrocortisone cream or calamine lotion may also be recommended to reduce inflammation and itching.<br>It is important to follow the treatment plan prescribed by your healthcare provider, even if you feel better before the prescription is finished. This will help to ensure that the infection is fully treated and to prevent the development of antibiotic-resistant bacteria.<br>It is also important to take steps to prevent the recurrence of cellulitis, such as practicing good hygiene, avoiding exposure to cold temperatures, and treating any skin breaks or injuries promptly.	
Evaluation of Physician	
Evaluation Item	Score (1-5, the higher the better)
SkinGPT-4’s diagnosis is correct or relevant.	5
SkinGPT-4’s description is informative.	5
SkinGPT-4’s suggestions are useful.	4
SkinGPT-4 can help doctors with diagnosis.	4
SkinGPT-4 can help patients to understand their disease better.	5
If SkinGPT-4 can be deployed locally, it protects patients’ privacy.	5

Willingness to use SkinGPT-4	4

Case Cellulitis 2

Image ID: Cellulitis2	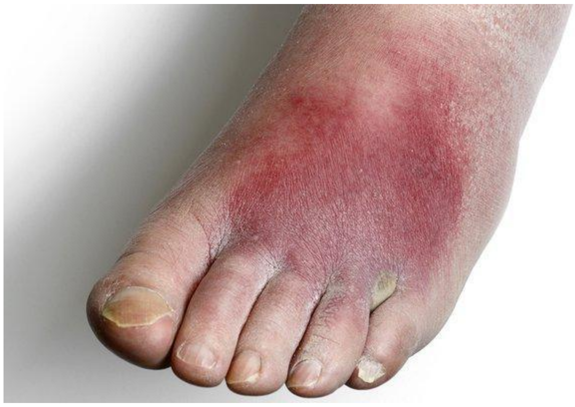
**SkinGPT-4**	**Dermatology Physician**
Q1. Could you describe the skin disease in this image for me?<br><br>A1. This could be a skin disease image of Erythema Infectiosum.<br><br>Q2. You should write a paragraph to list more features you observed in the image.<br><br>A2. Erythema Infectiosum is a chronic subacute skin condition characterized by red nodules or plaques that may ulcerate and leave scars. It is often found in immunocompromised individuals and can be caused by various bacteria, including Streptococcus, Staphylococcus, and Corynebacterium. The lesions can be itchy and sometimes have a white or yellow center. They can also be found on the face, neck, and limbs.<br><br>Q3. With previous information, please write a paragraph to explain in detail the cause of this skin disease.<br><br>A3. Erythema Infectiosum is caused by bacterial infection, usually in individuals with compromised immune systems, such as those with AIDS. The bacteria enter the body through a break in the skin and multiply, causing local inflammation, redness, and pain. The immune system attempts to fight the infection, leading to the formation of pus-filled bumps or nodules. Over time, the bumps may clear, leaving scars. The condition can also cause systemic symptoms such as fever and fatigue. The lesions are most commonly found on the skin,	Q1. Could you describe the skin disease in this image for me?<br><br>A1. Skin infections; Cellulitis; Erysipelas.<br><br>Q2. You should write a paragraph to list more features you observed in the image.<br><br>A2. An Erythema on acrotarsium which borders is usually ill-defined.<br><br>Q3. With previous information, please write a paragraph to explain in detail the cause of this skin disease.<br><br>A3. Cellulitis is an infection of the deep dermis and subcutaneous tissue that manifests as areas of erythema, swelling, warmth and tenderness. Erysipelas is a superficial variant of cellulitis caused primarily by group A streptococci that affects the dermis with prominent lymphatic involvement; in contrast, classic cellulitis is centered in the deep dermis and subcutaneous tissues.<br><br>Q4. How should I treat it? What medicine should I use?<br><br>A4. Oral antibiotics to clear the infection and prevent it from spreading to other parts of the body are very important.

but they can also occur on mucous membranes, nails, and hair follicles.<br><br>Q4. How should I treat it? What medicine should I use?<br><br>A4. Erythema Infectiosum is treated with antibiotics to clear the bacterial infection. The choice of antibiotic depends on the causative organism and the severity of the infection. Topical antibiotics, such as creams or ointments, may be used for mild cases. Oral antibiotics, such as penicillin or tetracycline, are prescribed for more severe or widespread infections. In some cases, a combination of topical and oral antibiotics may be necessary. It is important to follow the recommended treatment plan and complete the full course of medication to ensure that the infection is fully cleared. Proper hygiene and wound care are also essential to prevent the spread of infection and promote healing.	
Evaluation of Physician	
Evaluation Item	Score (1-5, the higher the better)
SkinGPT-4's diagnosis is correct or relevant.	1
SkinGPT-4's description is informative.	1
SkinGPT-4's suggestions are useful.	1
SkinGPT-4 can help doctors with diagnosis.	1
SkinGPT-4 can help patients to understand their disease better.	1
If SkinGPT-4 can be deployed locally, it protects patients' privacy.	5
Willingness to use SkinGPT-4	1

Case Cellulitis 3

Image ID: Cellulitis3	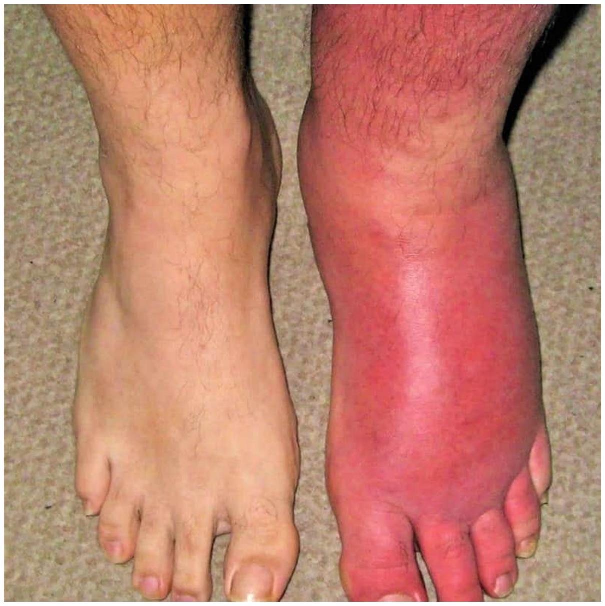
**SkinGPT-4**	**Dermatology Physician**
Q1. Could you describe the skin disease in this image for me?<br><br>A1. This could be a skin disease image of shoe allergy.<br><br>Q2. You should write a paragraph to list more features you observed in the image.<br><br>A2. The image is of a shoe allergy skin disease with features of eczema, erythema, edema, and fissures.<br><br>Q3. With previous information, please write a paragraph to explain in detail the cause of this skin disease.<br><br>A3. The cause of this skin disease is unknown. It could be idiopathic or caused by an allergy to a substance in shoes, such as a dye, adhesive, or leather. The symptoms include redness (erythema), swelling (edema), itching (pruritus), and fissures which are a sign of eczema. The fissures can be seen in the picture as white lines on the skin. The erythema and edema are due to an allergic response to the substance in the shoes, which leads to inflammation of the skin.	Q1. Could you describe the skin disease in this image for me?<br><br>A1. Skin infections; Cellulitis; Erysipelas.<br><br>Q2. You should write a paragraph to list more features you observed in the image.<br><br>A2. An Erythema on foot and lower legs which borders is usually ill-defined.<br><br>Q3. With previous information, please write a paragraph to explain in detail the cause of this skin disease.<br><br>A3. Cellulitis is an infection of the deep dermis and subcutaneous tissue that manifests as areas of erythema, swelling, warmth and tenderness. Erysipelas is a superficial variant of cellulitis caused primarily by group A streptococci that affects the dermis with prominent lymphatic involvement; in contrast, classic cellulitis is centered in the deep dermis and subcutaneous tissues.<br><br>Q4. How should I treat it? What medicine should I use?

The itching can be due to the same reason or due to an infectious agent taking advantage of the damaged skin.<br><br>Q4. How should I treat it? What medicine should I use?<br><br>A4.<br>The treatment of this skin disease depends on the severity of symptoms and the cause of the allergy. The first step in treatment is to identify and avoid the substance that is causing the allergy. This may involve removing the shoes, patch testing, or avoiding certain types of shoes. Topical corticosteroids can be used to reduce inflammation and itching. If the symptoms are severe, oral antihistamines or steroids may be prescribed. Antibiotics may be prescribed if there is any sign of infection. The best approach would be to consult a dermatologist for a proper diagnosis and treatment plan.	A4. Oral antibiotics to clear the infection and prevent it from spreading to other parts of the body are very important. Oral antibiotics to clear the infection and prevent it from spreading to other parts of the body are very important. For uncomplicated cases, a 10-day course of an oral antibiotic that covers these organisms (e.g. dicloxacillin, cephalexin or clindamycin) is appropriate.
Evaluation of Physician	
Evaluation Item	Score (1-5, the higher the better)
SkinGPT-4's diagnosis is correct or relevant.	1
SkinGPT-4's description is informative.	1
SkinGPT-4's suggestions are useful.	1
SkinGPT-4 can help doctors with diagnosis.	1
SkinGPT-4 can help patients to understand their disease better.	1
If SkinGPT-4 can be deployed locally, it protects patients' privacy.	5
Willingness to use SkinGPT-4	1

Case Cellulitis 4

Image ID: Cellulitis4	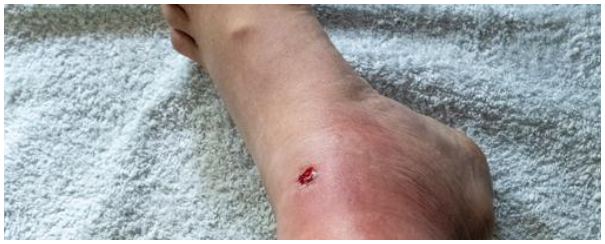
**SkinGPT-4**	**Dermatology Physician**
Q1. Could you describe the skin disease in this image for me?<br><br>A1. This could be a skin disease image of gonorrhea.<br><br>Q2. You should write a paragraph to list more features you observed in the image.<br><br>A2. This could be a skin disease image of gonorrhea. The image shows a person with a lesion on their skin which is a bit swollen and has a yellow color. The lesion has a central papule that is a bit elevated and has a pale colored center. The surrounding skin has erythema and slight desquamation. There is no pustule or vesicle. The lesion is on the thigh.<br><br>Q3. With previous information, please write a paragraph to explain in detail the cause of this skin disease.<br><br>A3. This image shows a case of gonorrhea, a sexually transmitted infection caused by Neisseria gonorrhea bacteria. The infection spreads through contact with infected sexual fluids, genital secretions, or skin lesions. It can also be transmitted from mother to baby during childbirth. Symptoms of gonorrhea include painful or burning urination, discharge from the penis or vagina, and painful or swollen testicles. Complications of gonorrhea include pelvic inflammatory disease, epididymitis, and infertility. The infection can be diagnosed through a laboratory test or by visual examination of discharge or tissue. Treatment involves antibiotics, usually ceftriaxone and azithromycin, which can cure the infection if diagnosed and treated in time. Prevention	Q1. Could you describe the skin disease in this image for me?<br><br>A1. Skin infections; Cellulitis; Erysipelas.<br><br>Q2. You should write a paragraph to list more features you observed in the image.<br><br>A2. An Erythema on foot which borders is usually ill-defined.<br><br>Q3. With previous information, please write a paragraph to explain in detail the cause of this skin disease.<br><br>A3. Cellulitis is an infection of the deep dermis and subcutaneous tissue that manifests as areas of erythema, swelling, warmth and tenderness. Erysipelas is a superficial variant of cellulitis caused primarily by group A streptococci that affects the dermis with prominent lymphatic involvement; in contrast, classic cellulitis is centered in the deep dermis and subcutaneous tissues.<br><br>Q4. How should I treat it? What medicine should I use?<br><br>A4. Oral antibiotics to clear the infection and prevent it from spreading to other parts of the body are very important. Oral antibiotics to clear the infection and prevent it from spreading to other parts of the body are very important. For uncomplicated cases, a 10-day course of an oral antibiotic that covers these organisms (e.g. dicloxacillin, cephalexin or clindamycin) is appropriate.

includes practicing safe sex, getting tested regularly, and seeking treatment if diagnosed with the infection. Complications can be prevented by completing the full course of antibiotics and avoiding sexual contact until the infection is completely cured.<br><br>Q4. How should I treat it? What medicine should I use?<br><br>A4. Gonorrhea is treated with antibiotics, usually a combination of ceftriaxone and azithromycin. The treatment regimen depends on the severity and location of the infection. If the infection is located in the throat, then an oral antibiotic may be prescribed. The antibiotics are usually given as a single dose or a short course, and they can cure the infection if diagnosed and treated in time. It is important to complete the full course of antibiotics as prescribed by a healthcare professional to prevent the development of antibiotic resistance and to ensure that the infection is fully cured.<br>If you are experiencing symptoms of gonorrhea, you should seek medical attention immediately. Your healthcare professional will perform a laboratory test or visual examination to diagnose the infection. They will also prescribe the appropriate antibiotics to treat the infection. It's essential to complete the full course of antibiotics as prescribed to avoid complications and to prevent the infection from recurring.	

Evaluation of Physician	
Evaluation Item	Score (1-5, the higher the better)
SkinGPT-4's diagnosis is correct or relevant.	1
SkinGPT-4's description is informative.	1
SkinGPT-4's suggestions are useful.	1
SkinGPT-4 can help doctors with diagnosis.	1
SkinGPT-4 can help patients to understand their disease better.	1
If SkinGPT-4 can be deployed locally, it protects patients' privacy.	5

Willingness to use SkinGPT-4	1

Case Cellulitis 5

<table>
<tr><td>Image ID: Cellulitis5</td><td>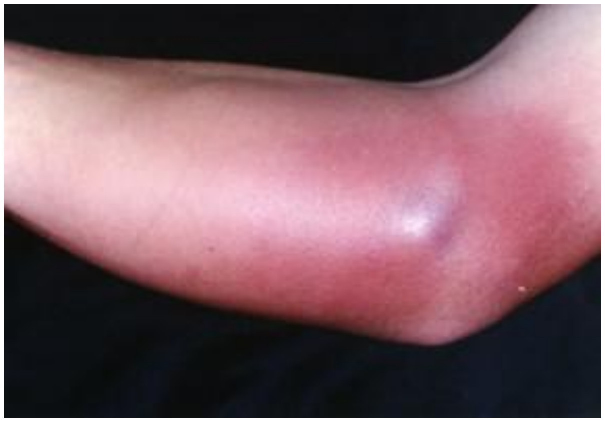</td></tr>
<tr><th>SkinGPT-4</th><th>Dermatology Physician</th></tr>
<tr><td>Q1. Could you describe the skin disease in this image for me?

A1. This could be a skin disease disease image of Cellulitis.

Q2. You should write a paragraph to list more features you observed in the image.

A2. This image shows a cellulitis, a bacterial infection that causes inflammation and swelling of the skin and subcutaneous tissue. The infection typically occurs in the lower legs, but can also occur in other parts of the body. The skin becomes bright red, warm, and swollen, and may have pus or oozing. It is accompanied by fever, chills, and general ill feeling. The condition is treated with oral antibiotics and wound care.

Q3. With previous information, please write a paragraph to explain in detail the cause of this skin disease.

A3. Cellulitis is caused by bacterial infection that penetrates the skin and spreads to the underlying tissue. The most common bacteria that cause cellulitis are streptococci and staphylococci. These bacteria enter the body through breaks in the skin, such as cuts, burns, or insect bites. Once inside the body, the bacteria release toxins that cause inflammation and swelling. The infection can also spread to other parts of the body through the lymphatic system. Risk factors for cellulitis include</td><td>Q1. Could you describe the skin disease in this image for me?

A1. Skin infections; Cellulitis; Erysipelas.

Q2. You should write a paragraph to list more features you observed in the image.

A2. An Erythema which borders is usually ill-defined and non-palpable.

Q3. With previous information, please write a paragraph to explain in detail the cause of this skin disease.

A3. Cellulitis is an infection of the deep dermis and subcutaneous tissue that manifests as areas of erythema, swelling, warmth and tenderness. Erysipelas is a superficial variant of cellulitis caused primarily by group A streptococci that affects the dermis with prominent lymphatic involvement; in contrast, classic cellulitis is centered in the deep dermis and subcutaneous tissues.

Q4. How should I treat it? What medicine should I use?

A4. Oral antibiotics to clear the infection and prevent it from spreading to other parts of the body are very important. Oral antibiotics to clear the infection and prevent it from spreading to other</td></tr>
</table>

obesity, diabetes, and chronic leg swelling. In this image, the person is most likely to have developed cellulitis as a result of a break in the skin, such as a cut or insect bite, allowing bacteria to enter and cause infection. The image shows a bright red, swollen, and warm skin, which is a characteristic feature of cellulitis. The condition is treated with antibiotics to clear the infection, and wound care to prevent further damage to the skin.<br><br>Q4. How should I treat it? What medicine should I use?<br><br>A4. Cellulitis is typically treated with oral antibiotics to clear the infection and prevent it from spreading to other parts of the body. The choice of antibiotic will depend on the cause of the infection and the person's medical history. In most cases, a broad-spectrum antibiotic such as cephalosporin or penicillin is used. Topical antibiotics and creams may also be prescribed to treat the skin lesion. It is important to follow the full course of antibiotics as prescribed by a healthcare professional, even if the symptoms improve before the full course is finished. Additionally, wound care is important to prevent further damage to the skin and promote healing. Pain relief medication may also be prescribed if necessary. It is important to seek medical attention for cellulitis to receive proper diagnosis and treatment.	parts of the body are very important. For uncomplicated cases, a 10-day course of an oral antibiotic that covers these organisms (e.g. dicloxacillin, cephalexin or clindamycin) is appropriate.
Evaluation of Physician	
Evaluation Item	Score (1-5, the higher the better)
SkinGPT-4's diagnosis is correct or relevant.	5
SkinGPT-4's description is informative.	5
SkinGPT-4's suggestions are useful.	5
SkinGPT-4 can help doctors with diagnosis.	5
SkinGPT-4 can help patients to understand their disease better.	5
If SkinGPT-4 can be deployed locally, it protects patients' privacy.	5

Willingness to use SkinGPT-4	5

Case Impetigo 1

<table>
<tr><td>Image ID: Impetigo1</td><td>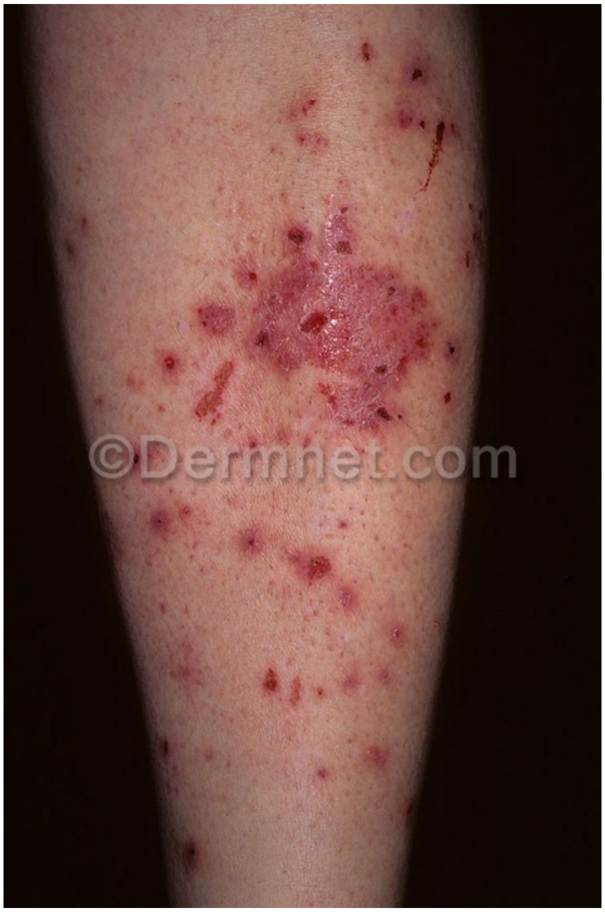
</td></tr>
<tr><td>SkinGPT-4</td><td>Dermatology Physician</td></tr>
<tr><td>Q1. Could you describe the skin disease in this image for me?

A1. This could be a skin disease image of impetigo.

Q2. You should write a paragraph to list more features you observed in the image.

A2. This image shows the skin disease impetigo. The disease is caused by bacterial infection, typically staphylococci or streptococci. It typically presents as a localized eruption of pustules, which rupture to form crusts. The lesions are often seen on the face, axillae, and groin. The disease is highly contagious and can spread through skin to skin contact or through contact with contaminated objects. The disease is typically treated with</td><td>Q1. Could you describe the skin disease in this image for me?

A1. Eczema + Skin infections; Hypersensitivity angiitis

Q2. You should write a paragraph to list more features you observed in the image.

A2. Epidermal erythema and scratching scattered over the lower leg.

Q3. With previous information, please write a paragraph to explain in detail the cause of this skin disease.

A3. Erythema and scabs can be the result of scratching because the itching.</td></tr>
</table>

topical antibiotics and/or oral antibiotics. Good personal hygiene, keeping the affected area clean and dry, and avoiding crowded places can help prevent the spread of impetigo.<br><br>Q3. With previous information, please write a paragraph to explain in detail the cause of this skin disease.<br><br>A3. Impetigo is a bacterial skin infection that is caused by two types of bacteria: streptococci and staphylococci. It typically affects the skin on the face, hands, and feet, and can spread through skin-to-skin contact or contact with contaminated objects. The bacteria enter the skin through breaks in the skin, such as cuts, scratches, or eczema. Once inside the skin, the bacteria release toxins that damage the tissue and cause inflammation. This leads to the formation of pustules, which are small, white-topped bumps filled with pus. When the pustules rupture, they leave behind red, crusty lesions. The lesions can be painful, especially when they occur on the hands or feet. Impetigo is highly contagious and can spread easily through contact with an infected person or object. Good personal hygiene, keeping the affected area clean and dry, and avoiding crowded places can help prevent the spread of impetigo. Treatment usually involves topical antibiotics and/or oral antibiotics to clear the infection and prevent complications.<br><br>Q4. How should I treat it? What medicine should I use?<br><br>A4. The treatment of impetigo depends on the severity and extent of the infection. Mild cases can be treated with topical antibiotics, such as bacitracin, polymyxin B, or erythromycin. These medications can be applied directly to the affected area two to three times a day. More severe cases or those that do not improve with topical treatment may require oral antibiotics, such as penicillin or doxycycline. It is important to follow the instructions for using the medication and complete the full course of treatment, even if the symptoms have improved. Good personal hygiene, keeping the affected area clean and dry, and avoiding crowded places can help prevent the spread of impetigo. It is important to see a doctor if the symptoms do not improve or if there are signs of	Q4. How should I treat it? What medicine should I use?<br><br>A4. Topical and oral antibiotics are required if skin infection is suspected. If there is a possibility of eczema, oral glucocorticoids will be required.

systemic illness, such as fever or swollen lymph nodes.	
Evaluation of Physician	
Evaluation Item	Score (1-5, the higher the better)
SkinGPT-4's diagnosis is correct or relevant.	2
SkinGPT-4's description is informative.	5
SkinGPT-4's suggestions are useful.	5
SkinGPT-4 can help doctors with diagnosis.	5
SkinGPT-4 can help patients to understand their disease better.	5
If SkinGPT-4 can be deployed locally, it protects patients' privacy.	5
Willingness to use SkinGPT-4	5

Case Impetigo 2

Image ID: Impetigo2	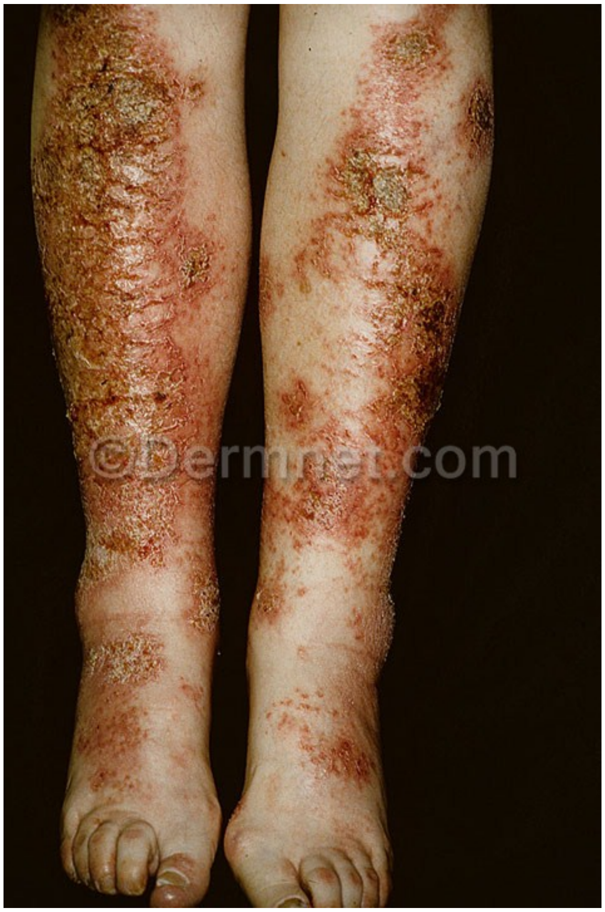
SkinGPT-4	Dermatology Physician
Q1. Could you describe the skin disease in this image for me?<br><br>A1. This could be a skin disease image of impetigo.<br><br>Q2. You should write a paragraph to list more features you observed in the image.<br><br>A2. This image depicts a child with impetigo, which is a bacterial skin infection that typically presents as a fluid filled blister on an erythematous base. The lesion can be either purulent or non purulent. The infection commonly occurs in children and can be spread through direct contact with a carrier or a contaminated object. It is important to treat impetigo promptly to prevent further spread and complications.	Q1. Could you describe the skin disease in this image for me?<br><br>A1. Eczema?<br><br>Q2. You should write a paragraph to list more features you observed in the image.<br><br>A2. Large erythema and scarred and crusted on lower leg<br><br>Q3. With previous information, please write a paragraph to explain in detail the cause of this skin disease.<br><br>A3. Erythema and scabs can be the result of scratching because the itching.

Q3. With previous information, please write a paragraph to explain in detail the cause of this skin disease.<br><br>A3. Impetigo is a bacterial skin infection caused by either Staphylococcus aureus or Group A Streptococcus. It typically affects the face, particularly around the nose and mouth, but can also occur on other parts of the body. The infection is contagious and can be spread through direct contact with an infected person or a contaminated object, such as a towel or razor. The bacteria enter the skin through small breaks, such as cuts or abrasions, and reproduce rapidly, causing the characteristic lesions of impetigo. The infection can be treated with antibiotics, but it is important to seek medical attention promptly to prevent complications and spread of the infection.<br><br>Q4. How should I treat it? What medicine should I use?<br><br>A4. Impetigo is typically treated with antibiotics. The most common bacteria responsible for impetigo are Staphylococcus aureus and Group A Streptococcus. The antibiotic of choice for impetigo is usually penicillin or amoxicillin, which are effective against both Staphylococcus aureus and Group A Streptococcus. If the patient is allergic to penicillin or amoxicillin, alternative antibiotics such as erythromycin, azithromycin, or cefuroxime can be used. Topical antibiotics, such as neomycin and bacitracin, can also be used in combination with oral antibiotics. It is important to follow the recommended treatment regimen and complete the full course of antibiotics to ensure that the infection is fully cleared.	Q4. How should I treat it? What medicine should I use?<br><br>A4. Topical and oral antibiotics are required if skin infection is suspected. If there is a possibility of eczema, oral glucocorticoids will be required.
Evaluation of Physician	
Evaluation Item	Score (1-5, the higher the better)
SkinGPT-4’s diagnosis is correct or relevant.	2
SkinGPT-4’s description is informative.	5
SkinGPT-4’s suggestions are useful.	5

SkinGPT-4 can help doctors with diagnosis.	5
SkinGPT-4 can help patients to understand their disease better.	5
If SkinGPT-4 can be deployed locally, it protects patients’ privacy.	5
Willingness to use SkinGPT-4	5

Case Impetigo 3

Image ID: Impetigo3	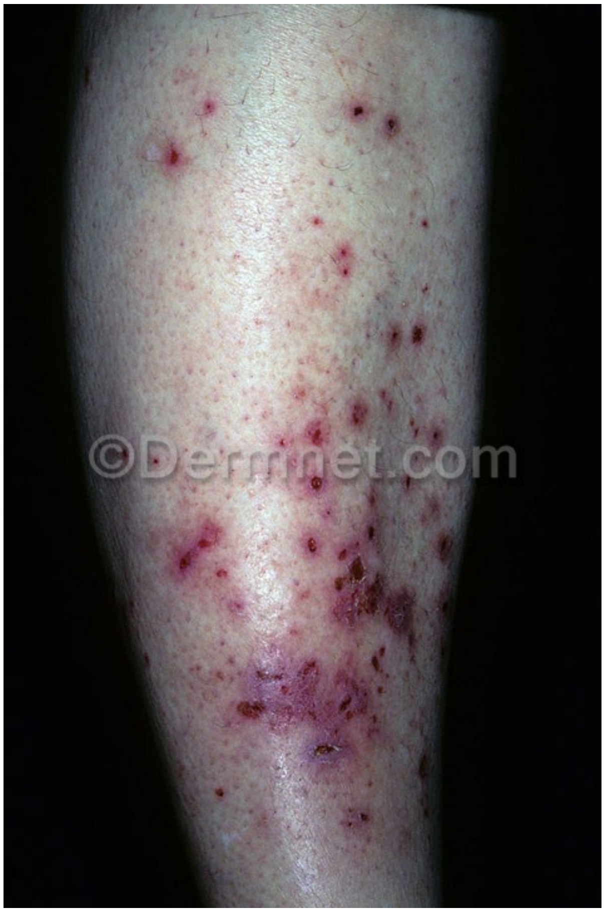
**SkinGPT-4**	**Dermatology Physician**
Q1. Could you describe the skin disease in this image for me?<br><br>A1. This could be a skin disease image of trauma.<br><br>Q2. You should write a paragraph to list more features you observed in the image.<br><br>A2. This image appears to be a skin disease image of trauma. The image shows several features such as redness, swelling, and pain. The skin appears to be inflamed with redness and the area looks swollen. The pain could be a sign of injury.<br><br>Q3. With previous information, please write a paragraph to explain in detail the cause of this skin disease.	Q1. Could you describe the skin disease in this image for me?<br><br>A1. Eczama, Skin infection; Hypersensitivity angiitis.<br><br>Q2. You should write a paragraph to list more features you observed in the image.<br><br>A2. Scattered erythema and scratching and crusting on the lower legs<br><br>Q3. With previous information, please write a paragraph to explain in detail the cause of this skin disease.<br><br>A3. Erythema and scabs can be the result of scratching because the itching.

A3. The cause of this skin disease is injury. The skin appears inflamed and swollen, which could be a result of an injury or trauma to the area. The redness and pain are also signs of injury, indicating that the skin is trying to heal itself. It is important to take care of the skin and provide appropriate treatment to prevent infection and promote healing. The image could also show the presence of bleeding, which is another sign of injury. The skin may also appear discolored or have an open wound, which can be seen in the image. Overall, this skin disease is caused by injury and it is important to seek medical attention to prevent complications and promote healing.<br><br>Q4. How should I treat it? What medicine should I use?<br><br>A4. The treatment for this skin disease caused by injury will depend on the severity of the injury and the overall health of the individual. It is important to seek medical attention to ensure proper treatment and prevent complications. The healthcare provider may recommend topical treatments such as antibiotics or antimicrobial creams to prevent infection and promote healing. Pain medication may also be prescribed to manage pain. In some cases, oral antibiotics may be prescribed if there is a risk of infection or if the wound is severe. The healthcare provider may also recommend wound care, such as cleaning the wound and covering it with a bandage, to promote healing and prevent infection. It is important to follow the healthcare provider's instructions and complete the full course of treatment to ensure full recovery and prevent complications.	Q4. How should I treat it? What medicine should I use?<br><br>A4. Topical and oral antibiotics are required if skin infection is suspected. If there is a possibility of eczema, oral glucocorticoids will be required.
Evaluation of Physician	
Evaluation Item	Score (1-5, the higher the better)
SkinGPT-4's diagnosis is correct or relevant.	2
SkinGPT-4's description is informative.	5
SkinGPT-4's suggestions are useful.	5

SkinGPT-4 can help doctors with diagnosis.	5
SkinGPT-4 can help patients to understand their disease better.	5
If SkinGPT-4 can be deployed locally, it protects patients' privacy.	5
Willingness to use SkinGPT-4	3

Case Sycosis Barbae 1

Image ID: Sycosis Barbae1	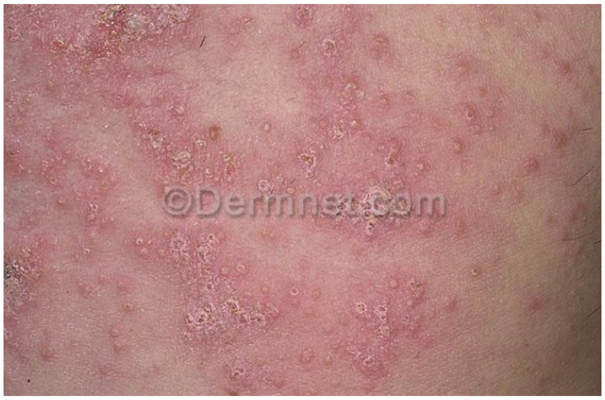
SkinGPT-4	Dermatology Physician
Q1. Could you describe the skin disease in this image for me?<br><br>A1. This could be a skin disease image of Sycosis Barbae.<br><br>Q2. You should write a paragraph to list more features you observed in the image.<br><br>A2. This image shows a skin disease sycosis barbae. The disease affects men and causes smooth, waxy feeling bumps on the face, especially on the cheeks and mustache area. The bumps are usually white or pale and can be mistaken for acne. Sycosis barbae is caused by an overgrowth of a normal skin bacteria, Staphylococcus epidermidis. It is more common in men and can be treated with antibiotics.<br><br>Q3. With previous information, please write a paragraph to explain in detail the cause of this skin disease.<br><br>A3. Sycosis barbae is caused by an overgrowth of the bacteria Staphylococcus epidermidis, which is normally present on the skin. The bacteria can infect the hair follicles in the face and cause inflammation and formation of bumps. The bumps are usually white or pale and can be mistaken for acne. The exact cause of the overgrowth of the bacteria is not known, but it can be associated with hormonal changes, stress, and poor hygiene. It is more common in men and can be treated with antibiotics.	Q1. Could you describe the skin disease in this image for me?<br><br>A1. Eczema(?); Skin infection(?).<br><br>Q2. You should write a paragraph to list more features you observed in the image.<br><br>A2. Extensive erythema, papules and crusting<br><br>Q3. With previous information, please write a paragraph to explain in detail the cause of this skin disease.<br><br>A3. The causes of eczema are complex, with both intrinsic and extrinsic factors.<br>The interaction between these factors is often complex. External factors such as living environment and climate External factors such as living environment and climate conditions can influence the development of eczema. External stimuli such as sunlight, ultraviolet rays, cold, heat, humidity and solar radiation can affect the development of eczema.<br>Some everyday products such as balms, soaps, artificial fibres, etc. can also affect the development of eczema.<br>Eczema can also be triggered by certain foods, such as chronic digestive disorders, functional disorders of the gastrointestinal tract.<br>Psychological changes such as emotional changes, infectious lesions, metabolic disorders and endocrine dysfunction can all contribute to eczema.

Q4. How should I treat it? What medicine should I use?<br><br>A4. Sycosis barbae is treated with topical antibiotics, such as erythromycin or clindamycin, which are applied directly to the affected areas of the face. The antibiotic helps to kill the bacteria that are causing the infection. The use of topical antibiotics may improve symptoms within a few weeks, but treatment may last for several months to ensure that the infection is fully cleared. Oral antibiotics may also be prescribed if the infection is severe or does not respond to topical treatment. It is important to follow the instructions of your healthcare provider regarding the use of medication for this skin disease.	Q4. How should I treat it? What medicine should I use?<br><br>A4. Topical and oral antibiotics are required if skin infection is suspected. If there is a possibility of eczema, oral glucocorticoids will be required.
Evaluation of Physician	
Evaluation Item	Score (1-5, the higher the better)
SkinGPT-4's diagnosis is correct or relevant.	1
SkinGPT-4's description is informative.	5
SkinGPT-4's suggestions are useful.	5
SkinGPT-4 can help doctors with diagnosis.	5
SkinGPT-4 can help patients to understand their disease better.	5
If SkinGPT-4 can be deployed locally, it protects patients' privacy.	5
Willingness to use SkinGPT-4	3

Case Bacteria Infection

<table>
<tr><td>Image ID: Bacteria Infection</td><td>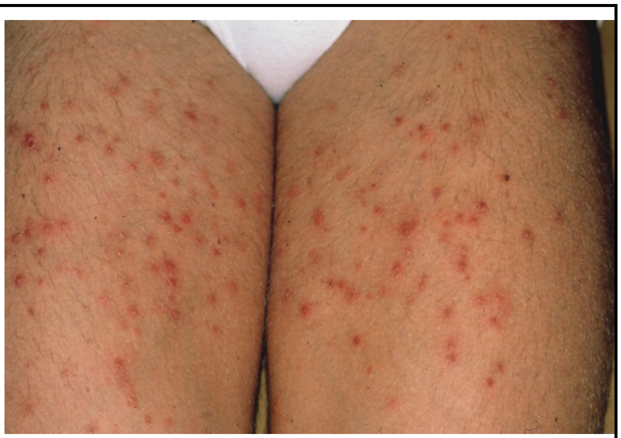</td></tr>
<tr><th>SkinGPT-4</th><th>Dermatology Physician</th></tr>
<tr><td>Q1. Could you describe the skin disease in this image for me?

A1. This could be a skin disease image of Pityriasis Lichenoides.

Q2. You should write a paragraph to list more features you observed in the image.

A2. This could be a skin disease image of Pityriasis Lichenoides. The disease is characterized by annular or polycyclic eruptions with clearly defined borders and sharply outlined white patches. The lesions are usually asymptomatic but can be pruritic. They are commonly found on the trunk and limbs, particularly in children. The disease is more common in males than females and is not considered life threatening. It is important to note that this image is not related to the question asked.

Q3. With previous information, please write a paragraph to explain in detail the cause of this skin disease.

A3. Pityriasis Lichenoides is a benign, acquired skin disease that is caused by a proliferation of T-cells in the epidermis. It is believed to be triggered by a viral infection, although the exact trigger is not known. The disease is characterized by the formation of annular or polycyclic eruptions with clearly defined borders and sharply outlined white</td><td>Q1. Could you describe the skin disease in this image for me?

A1. Prurigo

Q2. You should write a paragraph to list more features you observed in the image.

A2.

Q3. With previous information, please write a paragraph to explain in detail the cause of this skin disease.

A3. The cause of the disease is unclear. Most scientists believe it is related to a metamorphic reaction. Some patients have allergic conditions, such as urticaria and asthma, and the skin scratch test is positive. It is also thought to be caused by insect bites or allergies to drugs and food.

Q4. How should I treat it? What medicine should I use?

A4. Anti-histamine drugs can be a combination or alternation of two. Topical treatment is mainly antipruritic and anti-inflammatory.</td></tr>
</table>

patches. The lesions are asymptomatic but can be pruritic. The disease is more common in males than females and is not considered life threatening. It is important to note that this image is not related to the question asked.<br><br>Q4. How should I treat it? What medicine should I use?<br><br>A4. Pityriasis Lichenoides is a benign skin condition and does not require treatment. The disease typically resolves on its own within several months to a year without leaving any scars or changes in pigmentation. If symptoms such as itching are present, antihistamines or hydrocortisone cream may be prescribed by a dermatologist. However, as the disease is self limiting, treatment is usually not required. It is important to note that this image is not related to the question asked.	
Evaluation of Physician	
Evaluation Item	Score (1-5, the higher the better)
SkinGPT-4's diagnosis is correct or relevant.	3
SkinGPT-4's description is informative.	5
SkinGPT-4's suggestions are useful.	5
SkinGPT-4 can help doctors with diagnosis.	5
SkinGPT-4 can help patients to understand their disease better.	5
If SkinGPT-4 can be deployed locally, it protects patients' privacy.	5
Willingness to use SkinGPT-4	3

Case vitiligo 1

Image ID: vitiligo1	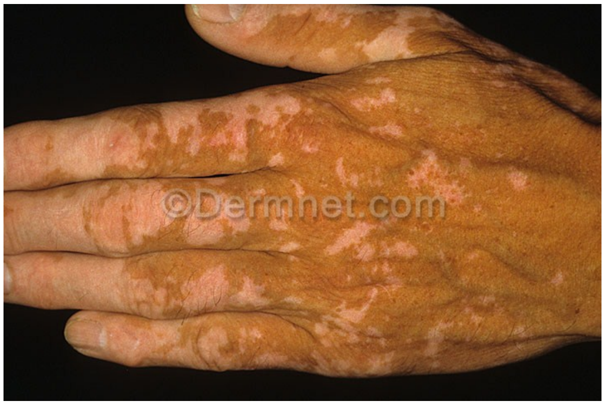
**SkinGPT-4**	**Dermatology Physician**
Q1. Could you describe the skin disease in this image for me?<br><br>A1. This could be a skin disease image of vitiligo.<br><br>Q2. You should write a paragraph to list more features you observed in the image.<br><br>A2. This image is of vitiligo, a skin condition characterized by the loss of pigmentation causing white patches on the skin. The patches are usually irregularly shaped and involve different parts of the body, including the face, neck, and skin folds. The cause of vitiligo is unknown, but it is thought to be an autoimmune disorder that affects melanin production. Melanin is the pigment responsible for skin color, and vitiligo occurs when the immune system attacks the melanin cells, causing them to die and resulting in the loss of pigmentation. This condition affects all races and both genders, and can be associated with other autoimmune diseases such as thyroid disorders, type 1 diabetes, and pernicious anemia. Treatment options for vitiligo include skin depigmentation, pigmentary restoration, and phototherapy.<br><br>Q3. With previous information, please write a paragraph to explain in detail the cause of this skin disease.<br><br>A3. This image represents vitiligo, which is a long-term skin condition characterized by patches of skin losing their pigment. The patches are usually white and flat, and may be different shapes and	Q1. Could you describe the skin disease in this image for me?<br><br>A1. Vitiligo.<br><br>Q2. You should write a paragraph to list more features you observed in the image.<br><br>A2. There are milk- or chalk-white macules or patches surrounded by normal skin on the hand.<br><br>Q3. With previous information, please write a paragraph to explain in detail the cause of this skin disease.<br><br>A3. Vitiligo is an acquired disorder characterized by circumscribed depigmented macules and patches that result from a progressive loss of functional melanocytes.<br><br>Q4. How should I treat it? What medicine should I use?<br><br>A4. The aims of vitiligo treatment are repigmentation and stabilization of the depigmentation process. Although there is still no therapeutic panacea for vitiligo, the available options lead to satisfactory results in a substantial number of patients. The choice of therapy depends on the extent, location, and activity of disease as well as the patient’s age, skin type, and motivation to undergo treatment. In general, a period of at least 2–3 months is required to determine whether a particular treatment is effective. The areas of the body that typically have

sizes. The cause of vitiligo is not known, but it is thought to be an autoimmune disease that occurs when the immune system attacks the cells that produce melanin. Melanin is the pigment that gives skin, hair, and eyes their color. When the cells that produce melanin are destroyed, the skin patches lose their pigment and turn white. Vitiligo can occur in anyone, but it is more noticeable in people with darker skin. It is not contagious and does not affect life expectancy, but it can affect a person's self-esteem and emotional well-being. There is no cure for vitiligo, but there are treatments available to help improve the appearance of the skin, such as skin depigmentation, pigmentary restoration, and phototherapy.<br><br>Q4. How should I treat it? What medicine should I use?<br><br>A4. Treatment for vitiligo depends on the severity and location of the patches, as well as the individual's preferences. There are several options available, including:<br>* Skin depigmentation: This involves using a cream or ointment to remove the remaining pigment from the affected skin, so that it matches the color of the surrounding skin.<br>* Pigmentary restoration: This involves using a cream or ointment to repigment the affected skin.<br>* Phototherapy: This involves exposing the affected skin to ultraviolet light in a controlled environment.<br>It's important to consult a dermatologist for the best treatment option as they will be able to evaluate the extent and location of the patches, and recommend the most appropriate treatment.	the best response to medical therapy are the face, neck, mid extremities and trunk, while the distal extremities and lips are the most resistant to treatment. Topical corticosteroids, tacrolimus 0.1% ointment or pimecrolimus 1% cream twice daily are useful for localized areas of vitiligo. The JAK inhibitor has are the latest agent for vitiligo. The narrowband UVB (NB-UVB) and the 308 nm excimer laser and lamp are considered to be effective in the treatment of vitiligo.
Evaluation of Physician	
Evaluation Item	Score (1-5, the higher the better)
SkinGPT-4's diagnosis is correct or relevant.	5
SkinGPT-4's description is informative.	5
SkinGPT-4's suggestions are useful.	5

SkinGPT-4 can help doctors with diagnosis.	5
SkinGPT-4 can help patients to understand their disease better.	5
If SkinGPT-4 can be deployed locally, it protects patients' privacy.	5
Willingness to use SkinGPT-4	5

Case vitiligo 2

Image ID: vitiligo2	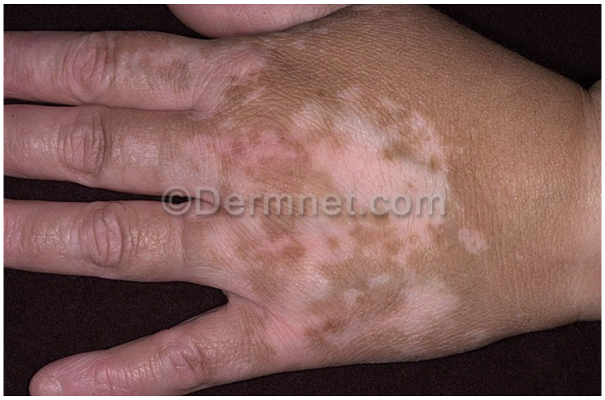
SkinGPT-4	Dermatology Physician
Q1. Could you describe the skin disease in this image for me?<br><br>A1. This could be a skin disease image of vitiligo.<br><br>Q2. You should write a paragraph to list more features you observed in the image.<br><br>A2. This image depicts a child with vitiligo, which is a skin condition characterized by the loss of pigmentation resulting in white patches on the skin. The patches are usually irregularly shaped and can affect any part of the body, including the face, neck, and skin folds. In this image, the child has vitiligo involving the patches on the lower extremities, and the trunk is involved as well. The disease is likely to affect other family members, as it has a genetic basis with a probability of 5 % in first degree relatives. The progression of the disease varies, but usually follows a piebald distribution, meaning it affects the skin in a mottled pattern, with a random distribution of depigmented and pigmented macules. This disease is difficult to treat, and attempts to repigment the skin with corticosteroids and other medical treatments have been unsuccessful.<br><br>Q3. With previous information, please write a paragraph to explain in detail the cause of this skin disease.<br><br>A3. This image depicts a child with vitiligo, which is a skin condition characterized by the loss of pigmentation resulting in white patches on the skin. The loss of pigmentation is due to the destruction	Q1. Could you describe the skin disease in this image for me?<br><br>A1. Vitiligo.<br><br>Q2. You should write a paragraph to list more features you observed in the image.<br><br>A2. There are milk- or chalk-white macules or patches surrounded by normal skin on the hand.<br><br>Q3. With previous information, please write a paragraph to explain in detail the cause of this skin disease.<br><br>A3. Vitiligo is an acquired disorder characterized by circumscribed depigmented macules and patches that result from a progressive loss of functional melanocytes.<br><br>Q4. How should I treat it? What medicine should I use?<br><br>A4. The aims of vitiligo treatment are repigmentation and stabilization of the depigmentation process. Although there is still no therapeutic panacea for vitiligo, the available options lead to satisfactory results in a substantial number of patients. The choice of therapy depends on the extent, location, and activity of disease as well as the patient’s age, skin type, and motivation to undergo treatment. In general, a period of at least 2–3 months is required to determine whether a particular treatment is effective. The areas of the body that typically have the best response to

of melanocytes, which are the cells responsible for producing melanin, the pigment that gives skin its color. The exact cause of vitiligo is not known, but it is believed to be an autoimmune disease in which the body's immune system attacks the melanocytes. The destruction of melanocytes leads to a loss of pigmentation, resulting in white patches on the skin. Vitiligo affects people of all skin types and can affect any part of the body, including the face, neck, and skin folds. The disease is more noticeable in people with darker skin because the contrast between the white patches and the surrounding skin is more pronounced. Vitiligo is not contagious and does not spread to other parts of the body. There is no cure for vitiligo, but treatments are available to help repigment the skin and improve the appearance of white patches. These treatments include corticosteroids, phototherapy, and skin grafting.

Q4. How should I treat it? What medicine should I use?

A4. The treatment of vitiligo depends on the severity and extent of the disease, as well as the patient's personal preferences. The goal of treatment is to repigment the skin and improve the appearance of white patches. Corticosteroids are the most commonly used treatment for vitiligo. They work by reducing the activity of the immune system, which in turn decreases the destruction of melanocytes. Corticosteroids are applied topically to the affected areas of the skin, and the dosage and frequency of application will depend on the severity of the disease.
Another option for treatment is phototherapy, which involves exposing the skin to ultraviolet (UV) light on a regular basis. This type of therapy is used to stimulate the production of melanin in the remaining melanocytes, which can help to repigment the skin.
Skin grafting is a surgical procedure in which healthy skin is taken from one part of the body and transplanted to an area with vitiligo. This procedure can be effective in treating small patches of vitiligo, but it is not commonly used due to the risk of rejection and scarring.
In summary, the treatment options for vitiligo are corticosteroids, phototherapy, and skin grafting.

medical therapy are the face, neck, mid extremities and trunk, while the distal extremities and lips are the most resistant to treatment. Topical corticosteroids, tacrolimus 0.1% ointment or pimecrolimus 1% cream twice daily are useful for localized areas of vitiligo. The JAK inhibitor has are the latest agent for vitiligo. The narrowband UVB (NB-UVB) and the 308 nm excimer laser and lamp are considered to be effective in the treatment of vitiligo.

Evaluation of Physician

Evaluation Item	Score (1-5, the higher the better)
SkinGPT-4's diagnosis is correct or relevant.	5
SkinGPT-4's description is informative.	5
SkinGPT-4's suggestions are useful.	5
SkinGPT-4 can help doctors with diagnosis.	5
SkinGPT-4 can help patients to understand their disease better.	5
If SkinGPT-4 can be deployed locally, it protects patients' privacy.	5
Willingness to use SkinGPT-4	5

Case vitiligo 3

Image ID: vitiligo3	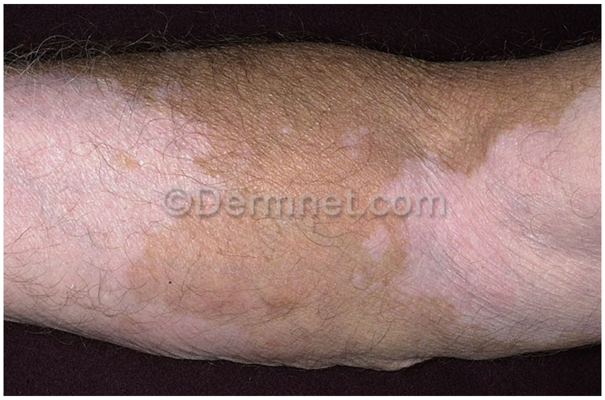
**SkinGPT-4**	**Dermatology Physician**
Q1. Could you describe the skin disease in this image for me?<br><br>A1. This could be a skin disease image of vitiligo.<br><br>Q2. You should write a paragraph to list more features you observed in the image.<br><br>A2. This image is a skin disease image of vitiligo. The disease is characterized by white patches on the skin, which are caused by a loss of pigment cells. The patches are usually symmetrical and may affect any part of the body, but are most commonly found on the face, neck, and extremities. In this image, the white patches are seen on the face and neck, which is a common area for vitiligo to affect. The patches appear as irregularly shaped and have sharp margins. The surrounding skin appears normal, suggesting that the patches are distinct from the surrounding skin. This image is a good example of vitiligo, a common autoimmune disorder that causes destruction of melanocytes, resulting in white patches on the skin.<br><br>Q3. With previous information, please write a paragraph to explain in detail the cause of this skin disease.<br><br>A3. Vitiligo is a skin disease characterized by white patches on the skin caused by a loss of pigment cells, called melanocytes. The exact cause of vitiligo is not known, but it is believed to be an autoimmune disorder, in which the immune system attacks the melanocytes. This leads to a	Q1. Could you describe the skin disease in this image for me?<br><br>A1. Vitiligo<br><br>Q2. You should write a paragraph to list more features you observed in the image.<br><br>A2. There are milk- or chalk-white macules or patches surrounded by normal skin on the arm.<br><br>Q3. With previous information, please write a paragraph to explain in detail the cause of this skin disease.<br><br>A3. Vitiligo is an acquired disorder characterized by circumscribed depigmented macules and patches that result from a progressive loss of functional melanocytes.<br><br>Q4. How should I treat it? What medicine should I use?<br><br>A4. The aims of vitiligo treatment are repigmentation and stabilization of the depigmentation process. Although there is still no therapeutic panacea for vitiligo, the available options lead to satisfactory results in a substantial number of patients. The choice of therapy depends on the extent, location, and activity of disease as well as the patient’s age, skin type, and motivation to undergo treatment. In general, a period of at least 2–3 months is required to determine whether a particular treatment is

deficiency of melanin, which is the pigment responsible for skin color. The process of vitiligo is thought to be a result of both genetic and environmental factors, and it can affect people of any race. The disorder affects both men and women, and it can appear at any age, but it is most common in people between the ages of 10 and 30. The white patches on the skin are usually symmetrical and may affect any part of the body, but are most commonly found on the face, neck, and extremities. There is no cure for vitiligo, but various treatments are available to help restore the skin color, such as corticosteroids, vitamin D analogs, and depigmentation. In this image, the white patches are seen on the face and neck, which is a common area for vitiligo to affect.<br><br>Q4. How should I treat it? What medicine should I use?<br><br>A4. Treatment of vitiligo depends on the severity and size of the white patches, as well as the patient's personal preferences. There are several options for treatment, including:<br>1. Corticosteroids: This is the most common treatment for vitiligo. Corticosteroids are applied topically to the skin or taken orally to decrease inflammation and promote repigmentation.<br>2. Vitamin D analogs: This is another common treatment for vitiligo. Vitamin D analogs are applied topically to the skin and work by stimulating the production of melanin.<br>3. Depigmentation: This treatment involves the removal of melanin from the unaffected skin to match the color of the affected skin. This can be done using creams, lotions, or surgical procedures.<br>4. Phototherapy: This treatment involves the use of ultraviolet (UV) light to stimulate the production of melanin.<br>It is important to consult a dermatologist for proper diagnosis and treatment. They will be able to evaluate the extent of the disease and recommend the best course of treatment based on the patient's individual needs.	effective. The areas of the body that typically have the best response to medical therapy are the face, neck, mid extremities and trunk, while the distal extremities and lips are the most resistant to treatment. Topical corticosteroids, tacrolimus 0.1% ointment or pimecrolimus 1% cream twice daily are useful for localized areas of vitiligo. The JAK inhibitor has are the latest agent for vitiligo. The narrowband UVB (NB-UVB) and the 308 nm excimer laser and lamp are considered to be effective in the treatment of vitiligo.
Evaluation of Physician	
Evaluation Item	Score (1-5, the higher the better)

SkinGPT-4's diagnosis is correct or relevant.	5
SkinGPT-4's description is informative.	5
SkinGPT-4's suggestions are useful.	5
SkinGPT-4 can help doctors with diagnosis.	5
SkinGPT-4 can help patients to understand their disease better.	5
If SkinGPT-4 can be deployed locally, it protects patients' privacy.	5
Willingness to use SkinGPT-4	5

Case vitiligo 4

<table>
<tr><td>Image ID: vitiligo4</td><td>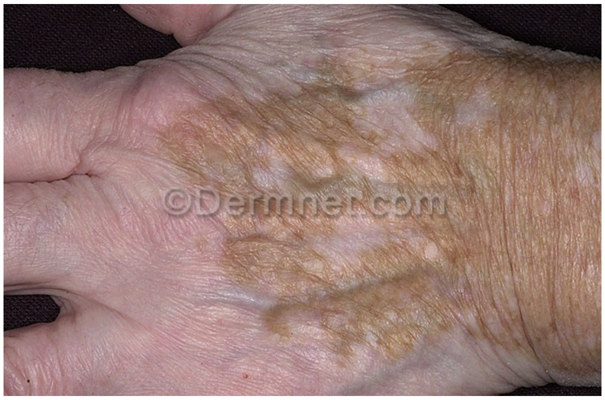
</td></tr>
<tr><th>SkinGPT-4</th><th>Dermatology Physician</th></tr>
<tr><td>Q1. Could you describe the skin disease in this image for me?

A1. This could be a skin disease image of vitiligo.

Q2. You should write a paragraph to list more features you observed in the image.

A2. The image is a skin disease image of vitiligo, which is a long term skin condition characterized by the loss of melanin pigment in patches on the skin, causing depigmentation. The patches are usually flat, white and irregularly shaped. In this image, the patches are seen on the shins and the stomach. The affected skin is hypopigmented, which is the opposite of hyperpigmentation. The pigmentation loss can affect both the skin and hair, and in this image, the hair on the head appears normal while the eyelashes are missing pigment. The pigment loss can occur due to autoimmune disease, trauma, or sunburn. This condition is progressive and treatment options include steroid creams, vitamin D analogs, and depigmentation.

Q3. With previous information, please write a paragraph to explain in detail the cause of this skin disease.

A3. The cause of this skin disease is autoimmune in nature. The immune system mistakenly attacks the melanocytes (cells responsible for producing melanin pigment) in the skin, leading to their destruction and subsequent loss of pigmentation. This destruction of melanocytes results in the characteristic white patches or leucoderma. The</td><td>Q1. Could you describe the skin disease in this image for me?

A1. Vitiligo.

Q2. You should write a paragraph to list more features you observed in the image.

A2. There are milk- or chalk-white macules or patches surrounded by normal skin on the hand.

Q3. With previous information, please write a paragraph to explain in detail the cause of this skin disease.

A3. Vitiligo is an acquired disorder characterized by circumscribed depigmented macules and patches that result from a progressive loss of functional melanocytes.

Q4. How should I treat it? What medicine should I use?

A4. The aims of vitiligo treatment are repigmentation and stabilization of the depigmentation process. Although there is still no therapeutic panacea for vitiligo, the available options lead to satisfactory results in a substantial number of patients. The choice of therapy depends on the extent, location, and activity of disease as well as the patient's age, skin type, and motivation to undergo treatment. In general, a period of at least 2–3 months is required to determine whether a particular treatment is effective. The areas of the body that typically have</td></tr>
</table>

exact trigger for this autoimmune response is not fully understood, but genetic predisposition, stress, trauma, and viral infections have been identified as potential triggers. The patches can appear on any part of the body, but are most commonly seen on the skin that is exposed to the sun, such as the face, hands, and feet. The condition is chronic and does not have a cure, but treatment options such as steroid creams, vitamin D analogs, and depigmentation can help in managing the symptoms and preventing progression of the disease.<br><br>Q4. How should I treat it? What medicine should I use?<br><br>A4. Treatment for vitiligo depends on the severity and extent of the disease. The goal of treatment is to stabilize pigmentation and prevent further depigmentation. The options for treatment include:<br>* Steroid creams: Corticosteroids can be applied topically to the affected areas to slow down the loss of pigment and repigment the skin.<br>* Vitamin D analogs: These are oral medications that can be used to repigment the skin. They work by increasing the levels of vitamin D in the body, which is essential for melanin production.<br>* Depigmentation: This involves removing the remaining pigment from the unaffected skin to match the affected skin. This can be done surgically or with bleaching creams.<br>* Combination therapy: A combination of the above treatments may be used to achieve the best results.<br>It is important to consult a dermatologist for proper evaluation and appropriate treatment. Self-medication should be avoided.	the best response to medical therapy are the face, neck, mid extremities and trunk, while the distal extremities and lips are the most resistant to treatment. Topical corticosteroids, tacrolimus 0.1% ointment or pimecrolimus 1% cream twice daily are useful for localized areas of vitiligo. The JAK inhibitor has are the latest agent for vitiligo. The narrowband UVB (NB-UVB) and the 308 nm excimer laser and lamp are considered to be effective in the treatment of vitiligo.

Evaluation of Physician	
Evaluation Item	Score (1-5, the higher the better)
SkinGPT-4's diagnosis is correct or relevant.	5
SkinGPT-4's description is informative.	5
SkinGPT-4's suggestions are useful.	5

SkinGPT-4 can help doctors with diagnosis.	5
SkinGPT-4 can help patients to understand their disease better.	5
If SkinGPT-4 can be deployed locally, it protects patients' privacy.	5
Willingness to use SkinGPT-4	5

Case vitiligo 5

Image ID: vitiligo5	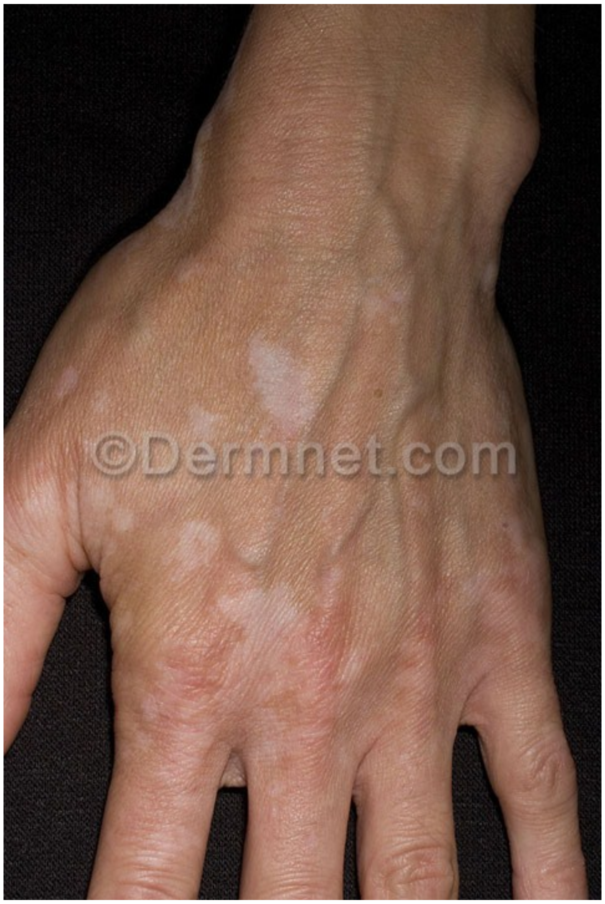
**SkinGPT-4**	**Dermatology Physician**
Q1. Could you describe the skin disease in this image for me?<br><br>A1. This could be a skin disease image of vitiligo.<br><br>Q2. You should write a paragraph to list more features you observed in the image.<br><br>A2. The following is a list of features observed in the image of vitiligo: The image shows a skin condition where the pigment producing cells called melanocytes are destroyed, leading to depigmented patches on the skin. The patches are white or lighter in color than the surrounding skin and can be varying sizes and shapes. The edges of the patches are often irregular and may appear rough or scaly. The affected skin may also be more sensitive to the sun and require protection with clothing or sunscreens. The cause	Q1. Could you describe the skin disease in this image for me?<br><br>A1. Vitiligo<br><br>Q2. You should write a paragraph to list more features you observed in the image.<br><br>A2. There are milk- or chalk-white macules or patches surrounded by normal skin on the hand.<br><br>Q3. With previous information, please write a paragraph to explain in detail the cause of this skin disease.<br><br>A3. Vitiligo is an acquired disorder characterized by circumscribed depigmented macules and patches that result from a progressive loss of functional melanocytes.

of vitiligo is not known, but it is thought to be an autoimmune disorder where the body attacks its own pigment cells.<br><br>Q3. With previous information, please write a paragraph to explain in detail the cause of this skin disease.<br><br>A3. Vitiligo is a skin disease characterized by the loss of pigment (melanin) from areas of the skin, resulting in irregular white patches or spots. The cause of vitiligo is not fully understood, but it is believed to be an autoimmune disorder, in which the immune system attacks the pigment-producing cells (melanocytes) in the skin. This results in a loss of melanin, which is responsible for giving skin its color. It can affect any race, but it is more noticeable in people with darker skin. There is no known cure for vitiligo, but there are various treatments available to help improve the appearance of the skin, such as creams, depigmentation, and skin grafting.<br><br>Q4. How should I treat it? What medicine should I use?<br><br>A4. Treatment for vitiligo depends on the severity and extent of the condition. The goal of treatment is to restore the skin's natural pigmentation and improve the appearance of the affected areas. Some treatments for vitiligo include:<br>* Corticosteroid creams or ointments to slow the loss of pigment<br>* Depigmentation, which involves removing the remaining pigment from the affected area to match the surrounding skin<br>* Skin grafting, which involves taking a small piece of healthy skin from another part of the body and attaching it to the affected area<br>* Excimer laser treatment, which can help repigment the skin in some cases<br>* Wearing sunscreen and avoiding sun exposure to prevent further pigment loss<br>It's important to note that there is no single treatment that works for everyone and the best treatment option depends on the individual case. It's best to consult with a dermatologist to determine the best course of treatment.	Q4. How should I treat it? What medicine should I use?<br><br>A4. The aims of vitiligo treatment are repigmentation and stabilization of the depigmentation process. Although there is still no therapeutic panacea for vitiligo, the available options lead to satisfactory results in a substantial number of patients. The choice of therapy depends on the extent, location, and activity of disease as well as the patient's age, skin type, and motivation to undergo treatment. In general, a period of at least 2–3 months is required to determine whether a particular treatment is effective. The areas of the body that typically have the best response to medical therapy are the face, neck, mid extremities and trunk, while the distal extremities and lips are the most resistant to treatment. Topical corticosteroids, tacrolimus 0.1% ointment or pimecrolimus 1% cream twice daily are useful for localized areas of vitiligo. The JAK inhibitor has are the latest agent for vitiligo. The narrowband UVB (NB-UVB) and the 308 nm excimer laser and lamp are considered to be effective in the treatment of vitiligo.
Evaluation of Physician	

Evaluation Item	Score (1-5, the higher the better)
SkinGPT-4's diagnosis is correct or relevant.	5
SkinGPT-4's description is informative.	5
SkinGPT-4's suggestions are useful.	5
SkinGPT-4 can help doctors with diagnosis.	5
SkinGPT-4 can help patients to understand their disease better.	5
If SkinGPT-4 can be deployed locally, it protects patients' privacy.	5
Willingness to use SkinGPT-4	5

Case vitiligo 6

Image ID: vitiligo6	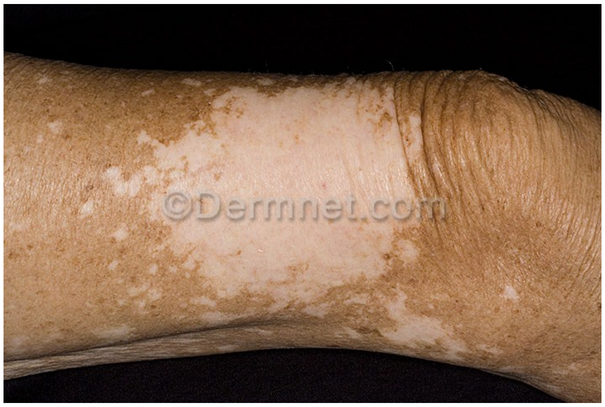
**SkinGPT-4**	**Dermatology Physician**
Q1. Could you describe the skin disease in this image for me?<br><br>A1. This could be a skin disease image of vitiligo.<br><br>Q2. You should write a paragraph to list more features you observed in the image.<br><br>A2. This could be a skin disease image of vitiligo. The image shows white patches on the skin, which are devoid of melanin pigment. The patches are irregularly shaped and have sharp margins. The affected areas are depigmented and appear lighter than the surrounding skin. The disease affects both skin and hair, leading to a loss of pigmentation in the affected areas. It can affect any part of the body, but common sites include the face, neck, and limbs. The cause of vitiligo is unknown, but it is believed to be an autoimmune disorder that affects the production of melanin pigment in the skin.<br><br>Q3. With previous information, please write a paragraph to explain in detail the cause of this skin disease.<br><br>A3. Vitiligo is an autoimmune skin disease that causes patches of skin to lose their pigment. The exact cause of vitiligo is not known, but it is believed to be related to an immune system disorder that destroys the cells responsible for producing melanin, the pigment that gives skin its color. These cells, called melanocytes, are located in the basal layer of the epidermis. In vitiligo, the immune system attacks and destroys these cells, leading to depigmentation of the skin. The disease affects	Q1. Could you describe the skin disease in this image for me?<br><br>A1. Vitiligo<br><br>Q2. You should write a paragraph to list more features you observed in the image.<br><br>A2. There are milk- or chalk-white macules or patches surrounded by normal skin on the hand/leg.<br><br>Q3. With previous information, please write a paragraph to explain in detail the cause of this skin disease.<br><br>A3. Vitiligo is an acquired disorder characterized by circumscribed depigmented macules and patches that result from a progressive loss of functional melanocytes.<br><br>Q4. How should I treat it? What medicine should I use?<br><br>A4. The aims of vitiligo treatment are repigmentation and stabilization of the depigmentation process. Although there is still no therapeutic panacea for vitiligo, the available options lead to satisfactory results in a substantial number of patients. The choice of therapy depends on the extent, location, and activity of disease as well as the patient's age, skin type, and motivation to undergo treatment. In general, a period of at least 2–3 months is required to determine whether a particular treatment is

both skin and hair, and can occur on any part of the body, although it is most commonly found on the face, neck, and limbs. Vitiligo is not contagious and does not cause any other symptoms, but it can have a significant impact on a person's self-esteem and quality of life. It is important to note that vitiligo is not a serious medical condition, but it can be difficult to treat. Treatment options include topical creams, UV light therapy, and skin grafting.<br><br>Q4. How should I treat it? What medicine should I use?<br><br>A4. The treatment of vitiligo depends on the severity and extent of the disease. The goal of treatment is to restore the pigment to the affected areas and improve the appearance of the skin. For mild cases of vitiligo, the doctor may recommend topical creams, such as corticosteroids or tacrolimus, to be applied to the affected areas of skin. These creams work by reducing inflammation and slowing down the loss of pigment. For more extensive vitiligo, the doctor may recommend a combination of treatments, including topical creams, phototherapy, and skin grafting. Phototherapy, also known as ultraviolet (UV) therapy, involves exposing the affected areas of skin to a specific wavelength of UV light to stimulate the production of melanin. Skin grafting involves taking healthy skin from one part of the body and attaching it to the affected area to repigment the skin.<br>In some cases, vitiligo can be difficult to treat, and the results may not be noticeable. It is important to talk to a dermatologist or other healthcare provider to determine the best course of treatment for your specific case of vitiligo.	effective. The areas of the body that typically have the best response to medical therapy are the face, neck, mid extremities and trunk, while the distal extremities and lips are the most resistant to treatment. Topical corticosteroids, tacrolimus 0.1% ointment or pimecrolimus 1% cream twice daily are useful for localized areas of vitiligo. The JAK inhibitor has are the latest agent for vitiligo. The narrowband UVB (NB-UVB) and the 308 nm excimer laser and lamp are considered to be effective in the treatment of vitiligo.
Evaluation of Physician	
Evaluation Item	Score (1-5, the higher the better)
SkinGPT-4's diagnosis is correct or relevant.	5
SkinGPT-4's description is informative.	5
SkinGPT-4's suggestions are useful.	5

SkinGPT-4 can help doctors with diagnosis.	5
SkinGPT-4 can help patients to understand their disease better.	5
If SkinGPT-4 can be deployed locally, it protects patients’ privacy.	5
Willingness to use SkinGPT-4	5

Case vitiligo 7

Image ID: vitiligo7	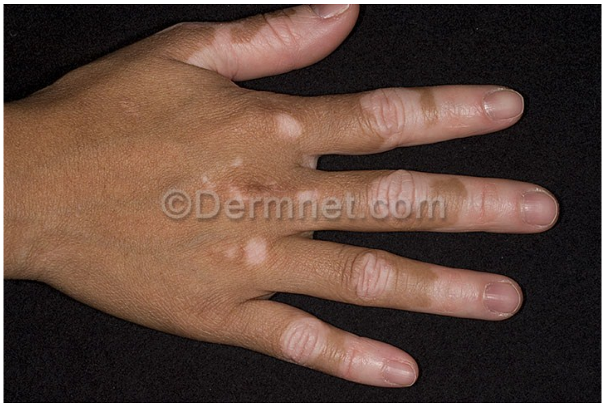
SkinGPT-4	Dermatology Physician
Q1. Could you describe the skin disease in this image for me?<br><br>A1. This could be a skin disease image of pompholyx.<br><br>Q2. You should write a paragraph to list more features you observed in the image.<br><br>A2. This image is a skin disease image of pompholyx. It is also known as "vacuoulcerosis" or "botryoma". It is a benign tumor of the sweat glands that typically occurs on the palms and soles. The tumor is made up of dilated sweat ducts that become inflamed and fill with keratin, causing a waxy appearance. The skin may appear normal or have a reddish hue. Itching, pain, and edema may occur. The disease most commonly affects middle aged adults and is more common in those with a history of wet work or moist environments.<br><br>Q3. With previous information, please write a paragraph to explain in detail the cause of this skin disease.<br><br>A3. Pompholyx is caused by an inflammatory response to an infectious agent such as Staphylococcus aureus, which colonizes the sweat ducts and triggers the immune response. The skin lesions typically occur on the palms and soles and are characterized by erythema, edema, and exudate. The wax-like material that accumulates in the dilated sweat ducts is composed of keratin and other proteins. The release of these proteins into the sweat ducts results in inflammation, which can	Q1. Could you describe the skin disease in this image for me?<br><br>A1. Vitiligo<br><br>Q2. You should write a paragraph to list more features you observed in the image.<br><br>A2. There are milk- or chalk-white macules or patches surrounded by normal skin on the hand.<br><br>Q3. With previous information, please write a paragraph to explain in detail the cause of this skin disease.<br><br>A3. Vitiligo is an acquired disorder characterized by circumscribed depigmented macules and patches that result from a progressive loss of functional melanocytes.<br><br>Q4. How should I treat it? What medicine should I use?<br><br>A4. The aims of vitiligo treatment are repigmentation and stabilization of the depigmentation process. Although there is still no therapeutic panacea for vitiligo, the available options lead to satisfactory results in a substantial number of patients. The choice of therapy depends on the extent, location, and activity of disease as well as the patient's age, skin type, and motivation to undergo treatment. In general, a period of at least 2–3 months is required to determine whether a particular treatment is effective. The areas of the

cause itching, pain, and discharge. The condition is more common in individuals with a history of moist work or exposure to moist environments, such as farmers, bakers, and kitchen workers. Risk factors for pompholyx include obesity, diabetes, and hormonal imbalances. Proper hygiene and use of topical antibiotics can help prevent the development of pompholyx.<br><br>Q4. How should I treat it? What medicine should I use?<br><br>A4. The treatment of pompholyx typically involves a combination of topical and oral medications. Topical therapy typically includes the use of antibiotic creams or ointments to eradicate the infecting organism and reduce inflammation. Oral antibiotics may also be prescribed if the infection is severe or there are systemic symptoms.<br><br>Steroid creams or ointments may be used to reduce inflammation and suppress the immune response. Warm compresses or soaks in dilute bleach solution can help soften and remove the hardened plugs in the ducts.<br>Avoiding moist work and exposure to moist environments can help prevent recurrence of the condition.<br>Proper hygiene and keeping the affected areas dry can also help prevent the spread of infection and reduce symptoms.<br>It is important to consult a dermatologist for proper diagnosis and treatment plan.	body that typically have the best response to medical therapy are the face, neck, mid extremities and trunk, while the distal extremities and lips are the most resistant to treatment. Topical corticosteroids, tacrolimus 0.1% ointment or pimecrolimus 1% cream twice daily are useful for localized areas of vitiligo. The JAK inhibitor has are the latest agent for vitiligo. The narrowband UVB (NB-UVB) and the 308 nm excimer laser and lamp are considered to be effective in the treatment of vitiligo.
Evaluation of Physician	
Evaluation Item	Score (1-5, the higher the better)
SkinGPT-4's diagnosis is correct or relevant.	1
SkinGPT-4's description is informative.	5
SkinGPT-4's suggestions are useful.	5
SkinGPT-4 can help doctors with diagnosis.	5
SkinGPT-4 can help patients to understand their disease better.	5

If SkinGPT-4 can be deployed locally, it protects patients’ privacy.	5
Willingness to use SkinGPT-4	1

Case vitiligo 8

Image ID: vitiligo8	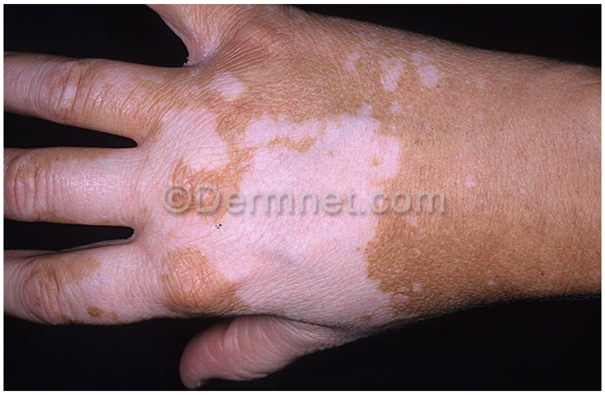
SkinGPT-4	Dermatology Physician
Q1. Could you describe the skin disease in this image for me?<br><br>A1. This could be a skin disease image of vitiligo.<br><br>Q2. You should write a paragraph to list more features you observed in the image.<br><br>A2. This image is of a vitiligo patch on the arm. The patch is white with border pallor and depigmented center. The patch is smooth, flat, and irregular in shape with a white border. The border is sharp and well defined with no scaling or crusting. The depigmented center is gray white with no skin texture change. There is no surrounding erythema or inflammation. The image is a skin disease image of vitiligo.<br><br>Q3. With previous information, please write a paragraph to explain in detail the cause of this skin disease.<br><br>A3. Vitiligo is a skin disease that causes depigmentation of the skin. It occurs when there is a loss of melanin, the pigment that gives skin its color, in certain areas of the skin. The exact cause of vitiligo is not known, but it is believed to be an autoimmune disorder in which the immune system attacks the melanin-producing cells in the skin. This leads to a loss of pigment and results in the development of white patches on the skin. The patches are usually symmetrical and can appear on any part of the body, including the face, neck, and limbs. Vitiligo does not affect skin function, but it can cause emotional distress and stigmatization, especially in people with darker	Q1. Could you describe the skin disease in this image for me?<br><br>A1. Vitiligo<br><br>Q2. You should write a paragraph to list more features you observed in the image.<br><br>A2. There are milk- or chalk-white macules or patches surrounded by normal skin on the hand.<br><br>Q3. With previous information, please write a paragraph to explain in detail the cause of this skin disease.<br><br>A3. Vitiligo is an acquired disorder characterized by circumscribed depigmented macules and patches that result from a progressive loss of functional melanocytes.<br><br>Q4. How should I treat it? What medicine should I use?<br><br>A4. The aims of vitiligo treatment are repigmentation and stabilization of the depigmentation process. Although there is still no therapeutic panacea for vitiligo, the available options lead to satisfactory results in a substantial number of patients. The choice of therapy depends on the extent, location, and activity of disease as well as the patient’s age, skin type, and motivation to undergo treatment. In general, a period of at least 2–3 months is required to determine whether a particular treatment is effective. The areas of the body that typically have the best response to medical therapy are the face, neck, mid extremities

skin types. There is no cure for vitiligo, but treatments such as steroids, UV therapy, and depigmentation can help to repigment the skin or blend in the white patches.<br><br>Q4. How should I treat it? What medicine should I use?<br><br>A4. Treatment of vitiligo depends on the severity and extent of the disease. The goal of treatment is to repigment the skin and reduce the size and number of white patches. The following treatments can be used:<br>1. Steroids: Applied topically or systemically, steroids can help to repigment the skin in some patients.<br>2. UV therapy: UV light exposure can help to repigment the skin in some patients.<br>3. Depigmentation: This involves the use of medications to lighten the remaining pigment in the skin to match the color of the white patches.<br>4. Surgery: Surgical techniques, such as skin grafting, can be used to repigment the skin in some patients.<br>It is important to consult a dermatologist for the appropriate treatment plan.	and trunk, while the distal extremities and lips are the most resistant to treatment. Topical corticosteroids, tacrolimus 0.1% ointment or pimecrolimus 1% cream twice daily are useful for localized areas of vitiligo. The JAK inhibitor has are the latest agent for vitiligo. The narrowband UVB (NB-UVB) and the 308 nm excimer laser and lamp are considered to be effective in the treatment of vitiligo.
Evaluation of Physician	
Evaluation Item	Score (1-5, the higher the better)
SkinGPT-4's diagnosis is correct or relevant.	5
SkinGPT-4's description is informative.	5
SkinGPT-4's suggestions are useful.	5
SkinGPT-4 can help doctors with diagnosis.	5
SkinGPT-4 can help patients to understand their disease better.	5
If SkinGPT-4 can be deployed locally, it protects patients' privacy.	5
Willingness to use SkinGPT-4	5

Case vitiligo 9

Image ID: vitiligo9	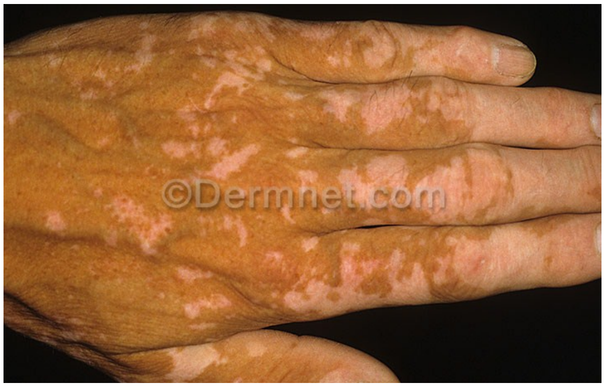
SkinGPT-4	Dermatology Physician
Q1. Could you describe the skin disease in this image for me?<br><br>A1. This could be a skin disease image of vitiligo.<br><br>Q2. You should write a paragraph to list more features you observed in the image.<br><br>A2. This image is of vitiligo, a condition characterized by loss of pigmentation resulting in white patches on the skin. The patches are usually circular or oval and vary in size. The patches may be stable for years or progressive and spread to other parts of the body. This condition affects both skin and mucosa and can be associated with other autoimmune diseases. The pigment-producing cells, melanocytes, are destroyed by the immune system, leading to depigmentation.<br><br>Q3. With previous information, please write a paragraph to explain in detail the cause of this skin disease.<br><br>A3. This image is of vitiligo, a skin condition characterized by patches of white skin caused by the loss of pigment-producing cells called melanocytes. The exact cause of vitiligo is not known, but it is believed to be an autoimmune disorder in which the immune system attacks the melanocytes, causing them to die and resulting in depigmentation. It is more common in people with a family history of the disorder and in people with other autoimmune diseases. The disease can affect any race, but it is more noticeable in people with darker skin. The white patches are usually	Q1. Could you describe the skin disease in this image for me?<br><br>A1. Vitiligo<br><br>Q2. You should write a paragraph to list more features you observed in the image.<br><br>A2. There are milk- or chalk-white macules or patches surrounded by normal skin on the hand.<br><br>Q3. With previous information, please write a paragraph to explain in detail the cause of this skin disease.<br><br>A3. Vitiligo is an acquired disorder characterized by circumscribed depigmented macules and patches that result from a progressive loss of functional melanocytes.<br><br>Q4. How should I treat it? What medicine should I use?<br><br>A4. The aims of vitiligo treatment are repigmentation and stabilization of the depigmentation process. Although there is still no therapeutic panacea for vitiligo, the available options lead to satisfactory results in a substantial number of patients. The choice of therapy depends on the extent, location, and activity of disease as well as the patient’s age, skin type, and motivation to undergo treatment. In general, a period of at least 2–3 months is required to determine whether a particular treatment is effective. The areas of the body that typically have the best response to

circular or oval and may be stable for years or progressive and spread to other parts of the body. There is no cure for vitiligo, but treatment options include skin grafting, depigmentation, and laser therapy.<br><br>Q4. How should I treat it? What medicine should I use?<br><br>A4. Treatment options for vitiligo include skin grafting, depigmentation, and laser therapy. Skin grafting involves taking a patch of normal-colored skin from another part of the body and transplanting it onto the affected area. Depigmentation involves using a cream to lighten the remaining pigment in the affected area. Laser therapy uses ultraviolet B light to stimulate the production of melanocytes and repigment the skin. In some cases, a combination of these treatments may be used. Medications such as corticosteroids and tacrolimus have also been used to treat vitiligo, but they may have side effects and are not commonly used. It is important to consult a dermatologist for the best treatment option for individual cases of vitiligo.	medical therapy are the face, neck, mid extremities and trunk, while the distal extremities and lips are the most resistant to treatment. Topical corticosteroids, tacrolimus 0.1% ointment or pimecrolimus 1% cream twice daily are useful for localized areas of vitiligo. The JAK inhibitor has are the latest agent for vitiligo. The narrowband UVB (NB-UVB) and the 308 nm excimer laser and lamp are considered to be effective in the treatment of vitiligo.

Evaluation of Physician	
Evaluation Item	Score (1-5, the higher the better)
SkinGPT-4’s diagnosis is correct or relevant.	5
SkinGPT-4’s description is informative.	5
SkinGPT-4’s suggestions are useful.	5
SkinGPT-4 can help doctors with diagnosis.	5
SkinGPT-4 can help patients to understand their disease better.	5
If SkinGPT-4 can be deployed locally, it protects patients’ privacy.	5
Willingness to use SkinGPT-4	5

Case vitiligo 10

<table>
<tr><td>Image ID: vitiligo10</td><td>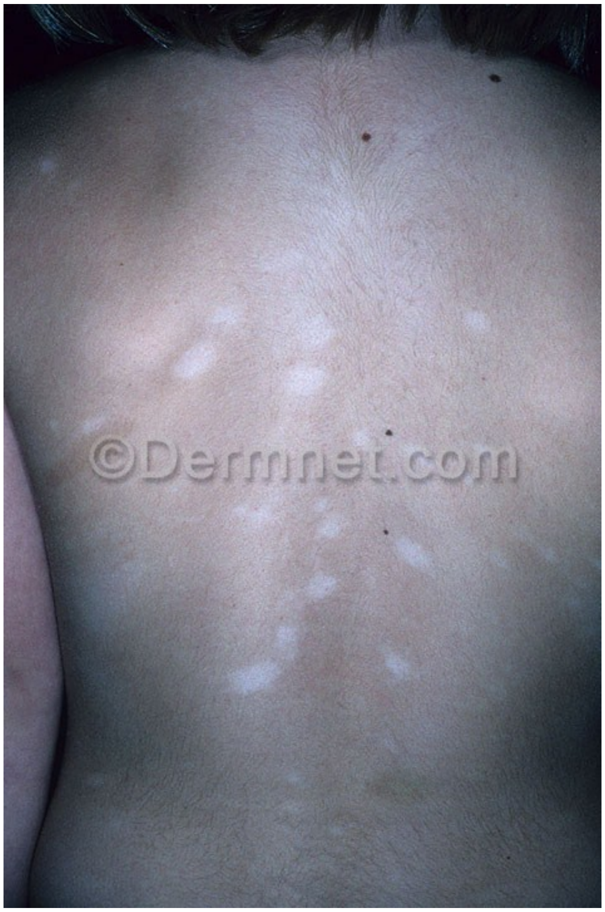
</td></tr>
<tr><th>SkinGPT-4</th><th>Dermatology Physician</th></tr>
<tr><td>Q1. Could you describe the skin disease in this image for me?

A1. This could be a skin disease image of tinea versicolor.

Q2. You should write a paragraph to list more features you observed in the image.

A2. The image shows a child with tinea versicolor, a common superficial fungal infection of the skin. The affected skin has whitish and scaly patches that are erythematous and slightly raised. The lesions are mostly found on the trunk and limbs, although they can appear on any part of the body. The child has a fair complexion and the patches are more pronounced on areas that have had sun exposure. The disease is caused by the overgrowth of a type of yeast, known as Pityrosporum ovale,</td><td>Q1. Could you describe the skin disease in this image for me?

A1. Vitiligo

Q2. You should write a paragraph to list more features you observed in the image.

A2. There are milk- or chalk-white macules or patches surrounded by normal skin on the back.

Q3. With previous information, please write a paragraph to explain in detail the cause of this skin disease.

A3. Vitiligo is an acquired disorder characterized by circumscribed depigmented macules and patches that result from a progressive loss of functional melanocytes.</td></tr>
</table>

which normally inhabits the skin and hair follicles. It is most common in tropical and subtropical regions, where humidity and temperature are high. The lesions are usually asymptomatic but can cause itching and discomfort. The disease can be treated with antifungal creams, lotions or shampoos, depending on the severity and extent of the infection.<br><br>Q3. With previous information, please write a paragraph to explain in detail the cause of this skin disease.<br><br>A3. Tinea versicolor is a skin disease caused by the overgrowth of a type of yeast, known as Pityrosporum ovale, which normally inhabits the skin and hair follicles. The disease is most common in tropical and subtropical regions, where humidity and temperature are high. The fungus produces metabolites that cause inflammation and skin damage, leading to the characteristic white, scaly, and erythematous patches that are observed in tinea versicolor. The erythema (redness) is due to increased blood flow to the affected skin, which is a part of the body's immune response to the fungal infection. The patches are usually asymptomatic but can cause itching and discomfort. The disease can be treated with antifungal creams, lotions or shampoos, depending on the severity and extent of the infection. The use of these medications can help to eradicate the fungus and restore the skin to its normal appearance. It is important to follow the instructions of the healthcare provider to ensure that the treatment is effective and safe. Prevention measures include keeping the skin clean and dry, avoiding excessive use of topical steroids, and reducing the use of antibiotics.<br><br>Q4. How should I treat it? What medicine should I use?<br><br>A4. The treatment of tinea versicolor depends on the severity and extent of the infection. Mild cases can be treated with over-the-counter antifungal creams or lotions containing ingredients such as clotrimazole, miconazole, or selenium sulfide. These creams or lotions are applied to the affected areas of skin once or twice a day for 2-4 weeks. More severe cases may require prescription antifungal medications, such as ketoconazole or itraconazole, which are taken orally or applied topically. These medications may	Q4. How should I treat it? What medicine should I use?<br><br>A4. The aims of vitiligo treatment are repigmentation and stabilization of the depigmentation process. Although there is still no therapeutic panacea for vitiligo, the available options lead to satisfactory results in a substantial number of patients. The choice of therapy depends on the extent, location, and activity of disease as well as the patient's age, skin type, and motivation to undergo treatment. In general, a period of at least 2–3 months is required to determine whether a particular treatment is effective. The areas of the body that typically have the best response to medical therapy are the face, neck, mid extremities and trunk, while the distal extremities and lips are the most resistant to treatment. Topical corticosteroids, tacrolimus 0.1% ointment or pimecrolimus 1% cream twice daily are useful for localized areas of vitiligo. The JAK inhibitor has are the latest agent for vitiligo. The narrowband UVB (NB-UVB) and the 308 nm excimer laser and lamp are considered to be effective in the treatment of vitiligo.

have more side effects than the over-the-counter options, so it is important to follow the instructions of a healthcare provider.<br>It is also important to follow these steps to prevent the spread of the infection and promote healing:<br>* Keep the affected skin clean and dry<br>* Avoid excessive use of topical steroids<br>* Reduce the use of antibiotics<br>* Wear loose-fitting clothing and avoid tight-fitting garments that can rub against the skin<br>* Avoid sharing personal items such as towels, razors, or hair brushes<br>* Avoid exposure to hot and humid environments	
Evaluation of Physician	
Evaluation Item	Score (1-5, the higher the better)
SkinGPT-4's diagnosis is correct or relevant.	1
SkinGPT-4's description is informative.	5
SkinGPT-4's suggestions are useful.	5
SkinGPT-4 can help doctors with diagnosis.	5
SkinGPT-4 can help patients to understand their disease better.	5
If SkinGPT-4 can be deployed locally, it protects patients' privacy.	5
Willingness to use SkinGPT-4	5

Case Lupus 1

Image ID: Lupus1	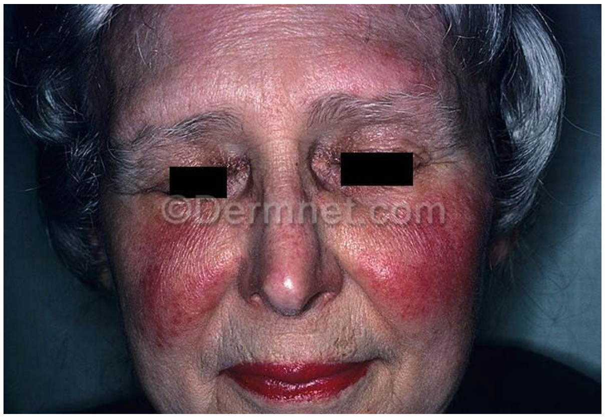
**SkinGPT-4**	**Dermatology Physician**
Q1. Could you describe the skin disease in this image for me?<br><br>A1. This could be a skin disease image of lupus acute.<br><br>Q2. You should write a paragraph to list more features you observed in the image.<br><br>A2. This image appears to depict a chronic inflammatory disease of the skin characterized by the presence of a malar rash, which is a reddish color, and a butterfly distribution. The rash is present on the face and has a distinct pattern resembling a butterfly. There is also a fine scale and erythema. The diagnosis of lupus erythematosus is considered as this disease affects multiple organ systems and has a chronic course.<br><br>Q3. With previous information, please write a paragraph to explain in detail the cause of this skin disease.<br><br>A3. The cause of this skin disease is unknown, but it is believed to have a genetic basis. It is characterized by chronic inflammation of the skin and other organ systems, such as the kidneys, heart, and lungs. The disease is caused by the presence of autoantibodies, which attack healthy cells in the body. The exact trigger that causes the autoimmune response is not known, but environmental factors, such as sun exposure, may play a role in the development of the disease. The	Q1. Could you describe the skin disease in this image for me?<br><br>A1. Inflammatory skin disease(?), Rosacea, Solar dermatitis, Cutaneous Lupus.<br><br>Q2. You should write a paragraph to list more features you observed in the image.<br><br>A2. The facial erythema, as a “butterfly rash” .<br><br>Q3. With previous information, please write a paragraph to explain in detail the cause of this skin disease.<br><br>A3. Lupus erythematosus is a multisystem disorder that prominently affects the skin. Cutaneous lesions are a source of disability and, on many occasions, an indicator of internal disease. The pathogenesis of cutaneous LE is complex, and it involves an interaction between genetic and environmental factors. The latter include ultraviolet radiation (UVR), medications, and possibly viruses. This interplay triggers a complex inflammatory cascade of cytokine, chemokine and inflammatory cell responses that include cells residing within as well as recruited to the skin. Diagnosis was by histopathological examination and direct immunofluorescence (DIF).<br><br>Q4. How should I treat it? What medicine should I use?

chronic inflammation in the skin leads to damage to tissues and can cause the characteristic rash seen in the image. The rash is often more prominent on sun-exposed areas, such as the face, and can be accompanied by other symptoms, such as fatigue and joint pain. The disease can be treated with medications that suppress the immune system, such as corticosteroids and antimalarial drugs, to reduce inflammation and prevent further damage to the skin.<br><br>Q4. How should I treat it? What medicine should I use?<br><br>A4. The treatment of lupus erythematosus depends on the severity of the disease and the affected organ systems. Medications that suppress the immune system, such as corticosteroids and antimalarial drugs, are often used to reduce inflammation and prevent further damage to the skin. These medications can be taken orally or applied topically, depending on the severity of the disease. In addition to medication, avoiding sun exposure and using sunscreen can help prevent worsening of the rash. Other treatments, such as phototherapy or immunosuppressive medications, may be used for severe cases of the disease that do not respond to other treatments. It is important to consult a dermatologist or a rheumatologist for proper diagnosis and treatment of this condition.	A4. Sun protection is very important. For local therapy, we can use topical and intralesional corticosteroids, calcineurin inhibitors and retinoids. For systemic therapy, we can use antimalarial drugs, Retinoids, Thalidomide and Sulfasalazine when antimalarial-resistant.
Evaluation of Physician	
Evaluation Item	Score (1-5, the higher the better)
SkinGPT-4's diagnosis is correct or relevant.	5
SkinGPT-4's description is informative.	5
SkinGPT-4's suggestions are useful.	5
SkinGPT-4 can help doctors with diagnosis.	5
SkinGPT-4 can help patients to understand their disease better.	5

If SkinGPT-4 can be deployed locally, it protects patients' privacy.	5
Willingness to use SkinGPT-4	5

Case Lupus 2

Image ID: Lupus2	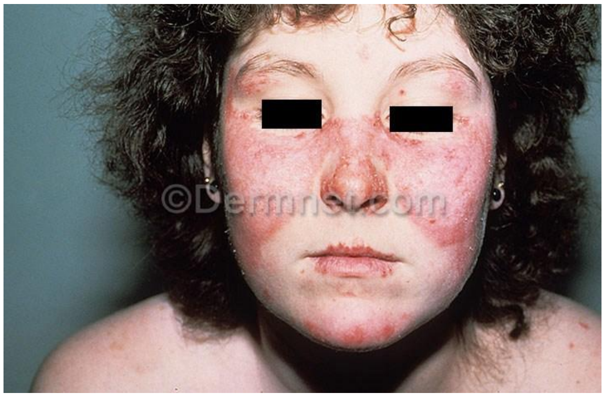
SkinGPT-4	Dermatology Physician
Q1. Could you describe the skin disease in this image for me?<br><br>A1. This could be a skin disease image of lupus acute.<br><br>Q2. You should write a paragraph to list more features you observed in the image.<br><br>A2. This image appears to depict a chronic inflammatory skin condition, likely lupus erythematosus. The rash is discoid in shape and involves the face, particularly the cheeks and nose. There is edema and erythema with a thick scale. The disease is active with many new lesions and some old lesions in the process of resolving. The lesions are sensitive to light and there is a heliotrophy. The disease is mild and there is no malar rash or lupus nephritis.<br><br>Q3. With previous information, please write a paragraph to explain in detail the cause of this skin disease.<br><br>A3. This skin disease is caused by a chronic autoimmune disorder known as systemic lupus erythematosus (SLE). The disease involves the production of autoantibodies against nuclear antigens and leads to inflammation and damage to various tissues and organs, including the skin. The rash in this image is likely a result of cutaneous involvement in SLE, which can present as discoid rash, involuntary scarring, and photosensitivity. The chronicity of the rash and the presence of scale suggest a more chronic and	Q1. Could you describe the skin disease in this image for me?<br><br>A1. Lupus, Cutaneous Lupus<br><br>Q2. You should write a paragraph to list more features you observed in the image.<br><br>A2. The facial erythema, with greyish-white adhesive scales, as a “butterfly rash”.<br><br>Q3. With previous information, please write a paragraph to explain in detail the cause of this skin disease.<br><br>A3. Lupus erythematosus is a multisystem disorder that prominently affects the skin. Cutaneous lesions are a source of disability and, on many occasions, an indicator of internal disease. The pathogenesis of cutaneous LE is complex, and it involves an interaction between genetic and environmental factors. The latter include ultraviolet radiation (UVR), medications, and possibly viruses. This interplay triggers a complex inflammatory cascade of cytokine, chemokine and inflammatory cell responses that include cells residing within as well as recruited to the skin.<br>Diagnosis was by histopathological examination and direct immunofluorescence (DIF).<br><br>Q4. How should I treat it? What medicine should I use?<br><br>A4. Sun protection is very important. For local

established process. The lack of malar rash and nephritis in this image suggests that the disease is milder and affecting fewer organs.<br><br>Q4. How should I treat it? What medicine should I use?<br><br>A4. The treatment of systemic lupus erythematosus (SLE) is typically managed by a rheumatologist, and may involve a combination of medications to control symptoms and prevent flare-ups. The mainstay of treatment is usually anti-inflammatory medications, such as non-steroidal anti-inflammatory drugs (NSAIDs) and corticosteroids, to reduce inflammation and pain. Additionally, immunosuppressive medications, such as azathioprine, mycophenolate mofetil, or cyclophosphamide, may be used to suppress the immune system and reduce the production of autoantibodies. Biologic agents, such as belimumab, may also be used to target specific molecules involved in the autoimmune process. It is important to note that each case of SLE is unique and treatment plans should be tailored to the individual patient based on the severity and specific symptoms of their disease.<br>Please let me know if you have any further questions.	therapy, we can use topical and intralesional corticosteroids, calcineurin inhibitors and retinoids. For systemic therapy, we can use antimalarial drugs, Retinoids, Thalidomide and Sulfasalazine when antimalarial-resistant.
Evaluation of Physician	
Evaluation Item	Score (1-5, the higher the better)
SkinGPT-4's diagnosis is correct or relevant.	5
SkinGPT-4's description is informative.	5
SkinGPT-4's suggestions are useful.	5
SkinGPT-4 can help doctors with diagnosis.	5
SkinGPT-4 can help patients to understand their disease better.	5
If SkinGPT-4 can be deployed locally, it protects patients' privacy.	5
Willingness to use SkinGPT-4	5

Case Lupus 3

Image ID: Lupus3	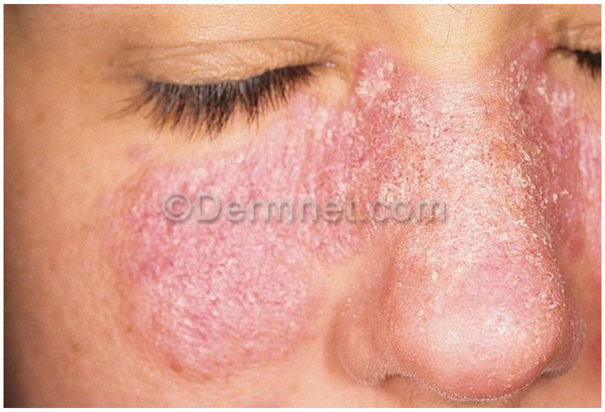
**SkinGPT-4**	**Dermatology Physician**
Q1. Could you describe the skin disease in this image for me?<br><br>A1. This could be a skin disease image of lupus chronic cutaneous.<br><br>Q2. You should write a paragraph to list more features you observed in the image.<br><br>A2. In this image, you can see a chronic cutaneous lupus erythematosus lesion. It presents as a reddish-purple, chronically inflamed plaque with irregular borders, scaly and fissured surface, and central clearing. The lesion is located on the face, which is a common site for lupus erythematosus. This image is a skin disease image of lupus chronic cutaneous.<br><br>Q3. With previous information, please write a paragraph to explain in detail the cause of this skin disease.<br><br>A3. Lupus chronic cutaneous is a type of cutaneous lupus erythematosus that presents as persistent, chronically inflamed skin lesions. The lesions can be flat or raised, and can occur on any part of the body, but are most commonly seen on the face, ears, and scalp. The exact cause of lupus chronic cutaneous is not known, but it is believed to be related to an immune-mediated disorder. In this disorder, the immune system attacks healthy cells and tissues, leading to inflammation and damage. Chronic cutaneous lupus erythematosus is thought to be caused by the same autoantibodies that cause systemic	Q1. Could you describe the skin disease in this image for me?<br><br>A1. Lupus, Cutaneous Lupus<br><br>Q2. You should write a paragraph to list more features you observed in the image.<br><br>A2. The facial erythema, with greyish-white adhesive scales, as a “butterfly rash”.<br><br>Q3. With previous information, please write a paragraph to explain in detail the cause of this skin disease.<br><br>A3. Lupus erythematosus is a multisystem disorder that prominently affects the skin. Cutaneous lesions are a source of disability and, on many occasions, an indicator of internal disease. The pathogenesis of cutaneous LE is complex, and it involves an interaction between genetic and environmental factors. The latter include ultraviolet radiation (UVR), medications, and possibly viruses. This interplay triggers a complex inflammatory cascade of cytokine, chemokine and inflammatory cell responses that include cells residing within as well as recruited to the skin.<br>Diagnosis was by histopathological examination and direct immunofluorescence (DIF).<br><br>Q4. How should I treat it? What medicine should I use?<br><br>A4. Sun protection is very important. For local

lupus erythematosus, but the exact trigger that leads to the development of skin lesions is not clear. The lesions of chronic cutaneous lupus erythematosus are usually resistant to treatment and require long-term management with topical steroids, antimalarials, and immunosuppressive medications.<br><br>Q4. How should I treat it? What medicine should I use?<br><br>A4. The treatment of lupus chronic cutaneous is aimed at reducing inflammation, preventing further damage, and managing symptoms. The following medications may be used to treat this condition:<br><br>1. Topical steroids: These are the first-line treatment for mild to moderate disease and are applied directly to the skin lesions.<br>2. Antimalarials: These medications, such as hydroxychloroquine, can help to reduce inflammation and prevent flare-ups.<br>3. Immunosuppressive medications: These medications, such as azathioprine or mycophenolate mofetil, are used to suppress the immune system and reduce inflammation in severe cases.<br>4. Phototherapy: This is a type of treatment that involves exposing the skin to ultraviolet light. It is used to improve the appearance of skin lesions.<br>In addition to medication, it is important to avoid sun exposure, use sunscreen, and follow a healthy lifestyle to manage this condition. Regular follow-ups with a dermatologist are necessary to monitor the disease and adjust treatment if needed.	therapy, we can use topical and intralesional corticosteroids, calcineurin inhibitors and retinoids. For systemic therapy, we can use antimalarial drugs, Retinoids, Thalidomide and Sulfasalazine when antimalarial-resistant.
Evaluation of Physician	
Evaluation Item	Score (1-5, the higher the better)
SkinGPT-4's diagnosis is correct or relevant.	5
SkinGPT-4's description is informative.	5
SkinGPT-4's suggestions are useful.	4
SkinGPT-4 can help doctors with diagnosis.	5

SkinGPT-4 can help patients to understand their disease better.	4
If SkinGPT-4 can be deployed locally, it protects patients' privacy.	5
Willingness to use SkinGPT-4	5

Case Lupus 4

Image ID: Lupus4	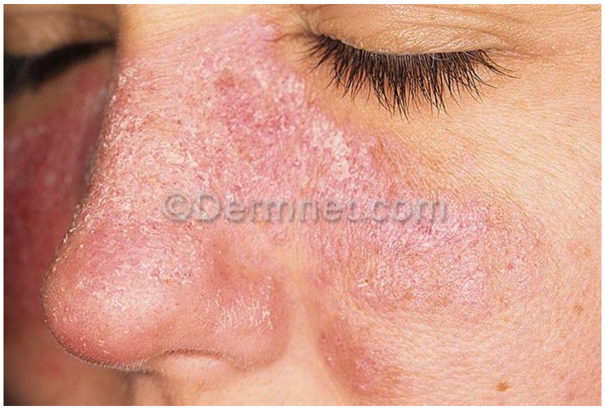
**SkinGPT-4**	**Dermatology Physician**
Q1. Could you describe the skin disease in this image for me?<br><br>A1. This could be a skin disease image of lupus chronic cutaneous.<br><br>Q2. You should write a paragraph to list more features you observed in the image.<br><br>A2. This could be a skin disease image of lupus chronic cutaneous. The lesion is located on the forehead and temples, with a red-blue color and sharp margins. There is scarring and atrophy with a thick, white, scaly plaque on the forehead. The nose and ears are affected, with a depressed, erythematous scar on the tip of the nose and a white, atrophic plaque on the lobule of the ear. The cheeks are also affected with a red, flaky scar, and the chin has a white, thick, scaling plaque. There is also a large, dark red, irregularly shaped scar on the neck. The disease is accompanied by hair loss and a photosensitive eruption on the scalp. The diagnosis is chronic cutaneous lupus.<br><br>Q3. With previous information, please write a paragraph to explain in detail the cause of this skin disease.<br><br>A3. This condition is caused by chronic cutaneous lupus, an autoimmune disorder that affects the skin and other organs. The disease is characterized by persistent inflammation and scarring of the skin, caused by the production of autoantibodies that attack healthy cells and	Q1. Could you describe the skin disease in this image for me?<br><br>A1. Lupus, Cutaneous Lupus<br><br>Q2. You should write a paragraph to list more features you observed in the image.<br><br>A2. The facial erythema, with greyish-white adhesive scales, as a “butterfly rash”.<br><br>Q3. With previous information, please write a paragraph to explain in detail the cause of this skin disease.<br><br>A3. Lupus erythematosus is a multisystem disorder that prominently affects the skin. Cutaneous lesions are a source of disability and, on many occasions, an indicator of internal disease. The pathogenesis of cutaneous LE is complex, and it involves an interaction between genetic and environmental factors. The latter include ultraviolet radiation (UVR), medications, and possibly viruses. This interplay triggers a complex inflammatory cascade of cytokine, chemokine and inflammatory cell responses that include cells residing within as well as recruited to the skin.<br>Diagnosis was by histopathological examination and direct immunofluorescence (DIF).<br><br>Q4. How should I treat it? What medicine should I use?<br><br>A4. Sun protection is very important. For local

<table>
<tr><td>tissues. The exact cause of lupus is not known, but it is believed to be related to genetic, environmental, and hormonal factors. In this particular case, the lesions are located on the forehead and temples, nose, ears, cheeks, chin, and neck, and are accompanied by hair loss and a photosensitive eruption on the scalp. The lesions are red-blue, thick, and scaly, with white, atrophic plaques, and depressed, erythematous scars. The disease is accompanied by a chronic inflammatory response in the skin, resulting in thickening of the skin, scarring, and hair loss. The diagnosis is confirmed by the presence of characteristic lesions and the presence of autoantibodies in the blood. Treatment involves corticosteroids, hydroxychloroquine, and sun protection to prevent further damage to the skin.

Q4. How should I treat it? What medicine should I use?

A4. Treatment for chronic cutaneous lupus involves a combination of medications to control inflammation, prevent scarring, and protect the skin from sun damage. The following medications are commonly used:
1. Topical corticosteroids: Applied directly to the skin to reduce inflammation and prevent scarring.
2. Hydroxychloroquine: An anti-inflammatory medication that can help reduce inflammation and prevent scarring.
3. Antimalarials: Medications such as quinine and chloroquine can help reduce inflammation and prevent skin damage from sunlight.
4. Photoprotection: Wearing protective clothing, hats, and sunscreens to avoid sun exposure, which can worsen the symptoms.
In some cases, systemic medications such as immunosuppressants, and biologic agents may be prescribed to treat severe cases of chronic cutaneous lupus that do not respond to topical medications. It is important to work closely with a dermatologist to develop an individualized treatment plan that takes into account the specific symptoms and severity of the disease.</td><td>therapy, we can use topical and intralesional corticosteroids, calcineurin inhibitors and retinoids. For systemic therapy, we can use antimalarial drugs, Retinoids, Thalidomide and Sulfasalazine when antimalarial-resistant.</td></tr>
<tr><td colspan="2">Evaluation of Physician</td></tr>
<tr><td>Evaluation Item</td><td>Score (1-5, the higher the better)</td></tr>
</table>

SkinGPT-4's diagnosis is correct or relevant.	5
SkinGPT-4's description is informative.	5
SkinGPT-4's suggestions are useful.	5
SkinGPT-4 can help doctors with diagnosis.	5
SkinGPT-4 can help patients to understand their disease better.	5
If SkinGPT-4 can be deployed locally, it protects patients' privacy.	5
Willingness to use SkinGPT-4	5

Case Lupus 5

Image ID: Lupus5	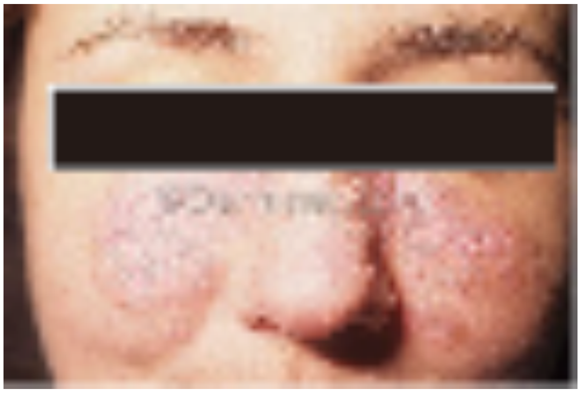
**SkinGPT-4**	**Dermatology Physician**
Q1. Could you describe the skin disease in this image for me?<br><br>A1. This could be a skin disease image of lupus chronic cutaneous.<br><br>Q2. You should write a paragraph to list more features you observed in the image.<br><br>A2. This image appears to depict a chronic cutaneous lupus erythematosus lesion, which is a type of lupus erythematosus that affects the skin. The lesion is flat and erythematous, with a sharp border and a central clearing. The surrounding skin is hyperpigmented, and there is telangiectasia and atrophy in the center of the lesion. The lesion is located on the cheek, which is a common site for lupus erythematosus cutaneous lesions.<br><br>Q3. With previous information, please write a paragraph to explain in detail the cause of this skin disease.<br><br>A3. This skin disease is caused by chronic cutaneous lupus erythematosus, which is an autoimmune disorder that affects the skin. In lupus erythematosus, the immune system attacks healthy cells and tissues in the body, including the skin. Chronic cutaneous lupus erythematosus is characterized by the development of flat, erythematous plaques on the skin that are usually located on sun-exposed areas such as the face, neck, and arms. The plaques have a sharp border and a central clearing, and can be accompanied	Q1. Could you describe the skin disease in this image for me?<br><br>A1. Lupus, Cutaneous Lupus<br><br>Q2. You should write a paragraph to list more features you observed in the image.<br><br>A2. The facial erythema, with greyish-white adhesive scales, as a “butterfly rash”.<br><br>Q3. With previous information, please write a paragraph to explain in detail the cause of this skin disease.<br><br>A3. Lupus erythematosus is a multisystem disorder that prominently affects the skin. Cutaneous lesions are a source of disability and, on many occasions, an indicator of internal disease. The pathogenesis of cutaneous LE is complex, and it involves an interaction between genetic and environmental factors. The latter include ultraviolet radiation (UVR), medications, and possibly viruses. This interplay triggers a complex inflammatory cascade of cytokine, chemokine and inflammatory cell responses that include cells residing within as well as recruited to the skin.<br>Diagnosis was by histopathological examination and direct immunofluorescence (DIF).<br><br>Q4. How should I treat it? What medicine should I use?<br><br>A4. Sun protection is very important. For local

by telangiectasia, atrophy, and scarring. The exact cause of chronic cutaneous lupus erythematosus is not known, but it is thought to be related to genetic and environmental factors. Treatment typically involves topical steroids, sun protection, and hydroxychloroquine, a drug that helps suppress the immune system.<br><br>Q4. How should I treat it? What medicine should I use?<br><br>A4. The treatment of chronic cutaneous lupus erythematosus typically involves a combination of topical and systemic therapies.<br>Topical therapy:<br>* Topical corticosteroids to reduce inflammation and suppress the immune system.<br>* Moisturizers to help hydrate the skin and reduce dryness.<br>Systemic therapy:<br>* Nonsteroidal anti-inflammatory drugs (NSAIDs) to reduce inflammation and suppress the immune system.<br>* Corticosteroids to reduce inflammation and suppress the immune system.<br>* Immunosuppressive drugs, such as hydroxychloroquine, to suppress the immune system.<br>* Biologic agents, such as rituximab or belimumab, which target specific parts of the immune system.<br><br>It's important to note that treatment for chronic cutaneous lupus erythematosus should be individualized and tailored to the specific needs of the patient. The best treatment approach for you can be determined by your doctor.	therapy, we can use topical and intralesional corticosteroids, calcineurin inhibitors and retinoids. For systemic therapy, we can use antimalarial drugs, Retinoids, Thalidomide and Sulfasalazine when antimalarial-resistant.
Evaluation of Physician	
Evaluation Item	Score (1-5, the higher the better)
SkinGPT-4's diagnosis is correct or relevant.	5
SkinGPT-4's description is informative.	5
SkinGPT-4's suggestions are useful.	5
SkinGPT-4 can help doctors with diagnosis.	5

SkinGPT-4 can help patients to understand their disease better.	5
If SkinGPT-4 can be deployed locally, it protects patients' privacy.	5
Willingness to use SkinGPT-4	5

Case Lupus 6

Image ID: Lupus6	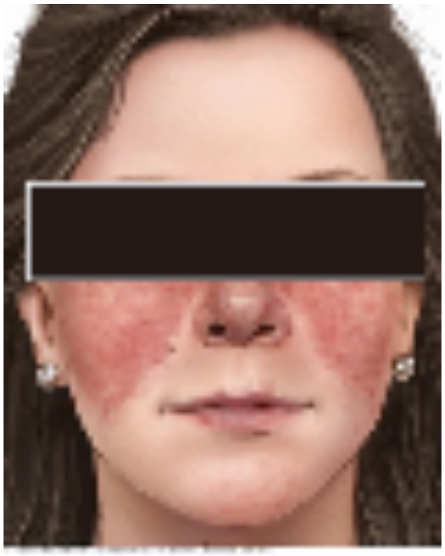
SkinGPT-4	Dermatology Physician
Q1. Could you describe the skin disease in this image for me?<br><br>A1. This could be a skin disease image of lupus acute.<br><br>Q2. You should write a paragraph to list more features you observed in the image.<br><br>A2. This image depicts a chronic cutaneous lupus erythematosus lesion. The lesion is erythematous and has a sharp margin. The center is atrophic and grayish white in color. The diameter of the lesion is approximately 1.5 cm. This image is a skin disease image of lupus chronic cutaneous.<br><br>Q3. With previous information, please write a paragraph to explain in detail the cause of this skin disease.<br><br>A3. The cause of this skin disease is not clear, but it is thought to be related to exposure to ultraviolet light, genetic predisposition, and immune dysfunction. Chronic cutaneous lupus erythematosus is a type of lupus that affects the skin, causing a persistent, erythematous rash on exposed areas of the skin, such as the face, ears, and neck. The rash is often photosensitive, meaning it becomes more inflamed and erythematous after exposure to sunlight. The	Q1. Could you describe the skin disease in this image for me?<br><br>A1. Lupus, Cutaneous Lupus<br><br>Q2. You should write a paragraph to list more features you observed in the image.<br><br>A2. The facial erythema, as a “butterfly rash”.<br><br>Q3. With previous information, please write a paragraph to explain in detail the cause of this skin disease.<br><br>A3. Lupus erythematosus is a multisystem disorder that prominently affects the skin. Cutaneous lesions are a source of disability and, on many occasions, an indicator of internal disease. The pathogenesis of cutaneous LE is complex, and it involves an interaction between genetic and environmental factors. The latter include ultraviolet radiation (UVR), medications, and possibly viruses. This interplay triggers a complex inflammatory cascade of cytokine, chemokine and inflammatory cell responses that include cells residing within as well as recruited to the skin.<br>Diagnosis was by histopathological examination and direct immunofluorescence (DIF).

center of the rash may be atrophic and grayish white in color. The rash can also be accompanied by scaly, red plaques, and scarring. The condition is treated with sun protection, corticosteroids, and antimalarials.<br><br>Q4. How should I treat it? What medicine should I use?<br><br>A4. The treatment of chronic cutaneous lupus erythematosus involves protecting the skin from ultraviolet light, reducing inflammation and promoting wound healing. The following medications can be used to treat chronic cutaneous lupus erythematosus:<br>* Topical corticosteroids: to reduce inflammation and promote healing.<br>* Photoprotective agents: to protect the skin from ultraviolet light.<br>* Anti-inflammatory agents: to reduce inflammation and pain.<br>* Antimalarials: to suppress the production of skin cells and reduce inflammation.<br>* Immunosuppressive agents: to suppress the immune system and reduce inflammation.<br>It is important to consult with a dermatologist for proper diagnosis and treatment of this skin disease.	Q4. How should I treat it? What medicine should I use?<br><br>A4. Sun protection is very important. For local therapy, we can use topical and intralesional corticosteroids, calcineurin inhibitors and retinoids. For systemic therapy, we can use antimalarial drugs, Retinoids, Thalidomide and Sulfasalazine when antimalarial-resistant.
Evaluation of Physician	
Evaluation Item	Score (1-5, the higher the better)
SkinGPT-4’s diagnosis is correct or relevant.	5
SkinGPT-4’s description is informative.	5
SkinGPT-4’s suggestions are useful.	4
SkinGPT-4 can help doctors with diagnosis.	5
SkinGPT-4 can help patients to understand their disease better.	5
If SkinGPT-4 can be deployed locally, it protects patients’ privacy.	5
Willingness to use SkinGPT-4	5

Case Lupus 7

Image ID: Lupus7	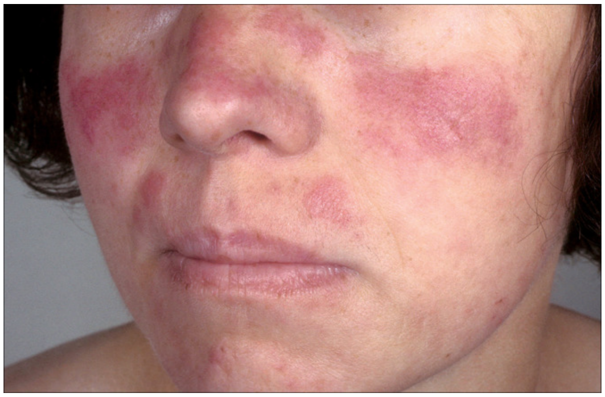
SkinGPT-4	Dermatology Physician
Q1. Could you describe the skin disease in this image for me?<br><br>A1. This could be a skin disease image of rosacea.<br><br>Q2. You should write a paragraph to list more features you observed in the image.<br><br>A2. In this image, we can see a face of a woman with a blush look on her cheeks. Her cheeks appear to be inflamed and red, and there is a bright eruption on her nose and cheeks. There is a thickening of the skin on her nose, and her skin appears to be scaly, with a yellowish tinge. Her eye lids appear to be swollen, and there is a blackhead on her nose. The image is an accurate representation of rosacea, a common skin condition that causes redness and inflammation of the face, particularly the cheeks, nose, and eyes.<br><br>Q3. With previous information, please write a paragraph to explain in detail the cause of this skin disease.<br><br>A3. Rosacea is a common skin condition that causes redness and inflammation of the face, particularly the cheeks, nose, and eyes. The exact cause of rosacea is unknown, but it is believed to be related to dilated blood vessels and inflammation of the skin's oil glands. It can be triggered by factors such as alcohol consumption, spicy foods, hot drinks, and sun exposure. The condition affects people of all skin types, but is more common in people with fair skin, and it tends	Q1. Could you describe the skin disease in this image for me?<br><br>A1. Rosacea, Solar dermatitis, Lupus, Cutaneous Lupus<br><br>Q2. You should write a paragraph to list more features you observed in the image.<br><br>A2. The facial erythema, as a "butterfly rash".<br><br>Q3. With previous information, please write a paragraph to explain in detail the cause of this skin disease.<br><br>A3. Lupus erythematosus is a multisystem disorder that prominently affects the skin. Cutaneous lesions are a source of disability and, on many occasions, an indicator of internal disease. The pathogenesis of cutaneous LE is complex, and it involves an interaction between genetic and environmental factors. The latter include ultraviolet radiation (UVR), medications, and possibly viruses. This interplay triggers a complex inflammatory cascade of cytokine, chemokine and inflammatory cell responses that include cells residing within as well as recruited to the skin.<br>Diagnosis was by histopathological examination and direct immunofluorescence (DIF).<br><br>Q4. How should I treat it? What medicine should I use?<br><br>A4. Sun protection is very important. For local

to run in families. Rosacea can be treated with topical creams, oral antibiotics, and laser therapy. The symptoms can be managed by avoiding triggers, wearing sunscreen, and using a gentle skincare routine.<br><br>Q4. How should I treat it? What medicine should I use?<br><br>A4. The treatment of rosacea depends on the severity and duration of symptoms. Mild cases can be treated with over-the-counter creams or gels containing ingredients such as metronidazole or azelaic acid. These medications help reduce inflammation and redness. More severe cases may require prescription oral antibiotics such as doxycycline or minocycline, or antibiotic creams or gels applied directly to the skin. In some cases, isotretinoin, a retinoid medication, may be prescribed for treatment-resistant rosacea. Laser therapy or photodynamic therapy may also be recommended to help reduce inflammation and improve symptoms. It's important to consult a dermatologist for proper diagnosis and treatment plan.<br>The medicines that can be used to treat rosacea are:<br>* Topical metronidazole or azelaic acid<br>* Oral antibiotics such as doxycycline or minocycline<br>* Antibiotic creams or gels<br>* Isotretinoin (Accutane)<br>* Laser therapy or photodynamic therapy.	therapy, we can use topical and intralesional corticosteroids, calcineurin inhibitors and retinoids. For systemic therapy, we can use antimalarial drugs, Retinoids, Thalidomide and Sulfasalazine when antimalarial-resistant.

Evaluation of Physician	
Evaluation Item	Score (1-5, the higher the better)
SkinGPT-4's diagnosis is correct or relevant.	2
SkinGPT-4's description is informative.	5
SkinGPT-4's suggestions are useful.	5
SkinGPT-4 can help doctors with diagnosis.	5
SkinGPT-4 can help patients to understand their disease better.	5

If SkinGPT-4 can be deployed locally, it protects patients' privacy.	5
Willingness to use SkinGPT-4	1

Case Lupus 8

Image ID: Lupus8	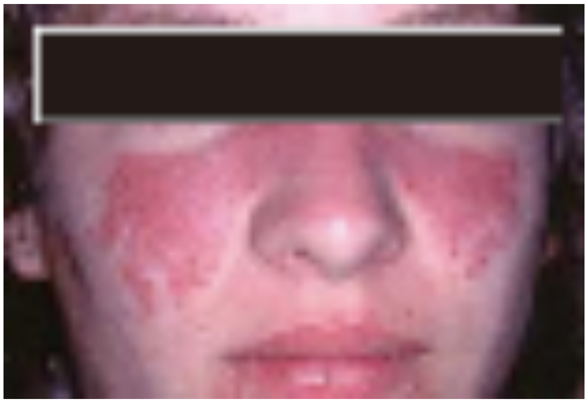
SkinGPT-4	Dermatology Physician
Q1. Could you describe the skin disease in this image for me?<br><br>A1. This could be a skin disease image of lupus.<br><br>Q2. You should write a paragraph to list more features you observed in the image.<br><br>A2. The image is of a skin disease called lupus erythematosus. This disease is characterized by chronic cutaneous inflammation and can affect any part of the body, especially exposed areas. The lesions are erythematous and may have a malar distribution. The disease can also involve mucous membranes, nails, and hair.<br><br>Q3. With previous information, please write a paragraph to explain in detail the cause of this skin disease.<br><br>A3. Lupus erythematosus is an autoimmune disease in which the immune system attacks healthy cells and tissues, causing inflammation and damage. The exact cause of lupus erythematosus is not known, but it is thought to be a genetically determined disorder that can be triggered by environmental factors. The disease is characterized by the production of autoantibodies, which attack nucleic acids, proteins, and cell surface antigens. This leads to the activation of immune cells, particularly T cells and B cells, which cause inflammation and damage to tissues. Lupus erythematosus can affect various parts of the body, including the skin, joints, kidneys, heart,	Q1. Could you describe the skin disease in this image for me?<br><br>A1. Rosacea, Solar dermatitis, Lupus, Cutaneous Lupus<br><br>Q2. You should write a paragraph to list more features you observed in the image.<br><br>A2. The facial erythema , as a “butterfly rash”.<br><br>Q3. With previous information, please write a paragraph to explain in detail the cause of this skin disease.<br><br>A3. Lupus erythematosus is a multisystem disorder that prominently affects the skin. Cutaneous lesions are a source of disability and, on many occasions, an indicator of internal disease. The pathogenesis of cutaneous LE is complex, and it involves an interaction between genetic and environmental factors. The latter include ultraviolet radiation (UVR), medications, and possibly viruses. This interplay triggers a complex inflammatory cascade of cytokine, chemokine and inflammatory cell responses that include cells residing within as well as recruited to the skin.<br>Diagnosis was by histopathological examination and direct immunofluorescence (DIF).<br><br>Q4. How should I treat it? What medicine should I use?<br><br>A4. Sun protection is very important. For local

and lungs, leading to a wide range of symptoms such as skin rashes, joint pain, fatigue, and fever.<br><br>Q4. How should I treat it? What medicine should I use?<br><br>A4. Treatment of lupus erythematosus depends on the severity and extent of the disease. The goal of treatment is to manage symptoms, reduce inflammation, and prevent further damage to tissues and organs. Medications commonly used to treat lupus erythematosus include nonsteroidal antiinflammatory drugs (NSAIDs) for pain and inflammation, corticosteroids to reduce inflammation, and immunosuppressive drugs to suppress the immune system. In severe cases, treatment may involve chemotherapy or plasma exchange. It is important to consult with a dermatologist or rheumatologist for proper diagnosis and treatment.	therapy, we can use topical and intralesional corticosteroids, calcineurin inhibitors and retinoids. For systemic therapy, we can use antimalarial drugs, Retinoids, Thalidomide and Sulfasalazine when antimalarial-resistant.
Evaluation of Physician	
Evaluation Item	Score (1-5, the higher the better)
SkinGPT-4's diagnosis is correct or relevant.	5
SkinGPT-4's description is informative.	5
SkinGPT-4's suggestions are useful.	5
SkinGPT-4 can help doctors with diagnosis.	5
SkinGPT-4 can help patients to understand their disease better.	5
If SkinGPT-4 can be deployed locally, it protects patients' privacy.	5
Willingness to use SkinGPT-4	5

Case Lupus 9

Image ID: Lupus9	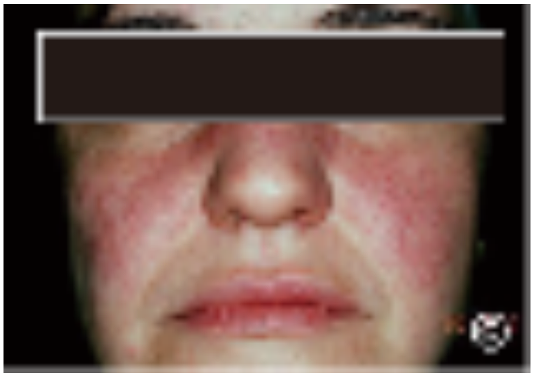
SkinGPT-4	Dermatology Physician
Q1. Could you describe the skin disease in this image for me?<br><br>A1. This could be a skin disease image of lupus acute.<br><br>Q2. You should write a paragraph to list more features you observed in the image.<br><br>A2. The image is of a face with acute lupus erythematosus. The rash is diffuse and erythematous with malar flushing and some scarring. The nose and ears are also affected with erythema. The eyes are affected with conjunctivitis. The lips are swollen and atrophic. There is a white rim around the erythema, which is called the lupus erythematosus rash or butterfly rash.<br><br>Q3. With previous information, please write a paragraph to explain in detail the cause of this skin disease.<br><br>A3. The cause of this skin disease is unknown, but it is thought to be related to a complex interplay of genetic, environmental, and immunological factors. It is characterized by chronic inflammation of the skin, which can result in a variety of skin lesions, including erythema, edema, and scarring. The rash can be triggered by exposure to UV light, physical trauma, infections, or stress, and can worsen with time. The immune system plays a critical role in the development of lupus erythematosus, as it produces autoantibodies that attack healthy cells and tissues. The condition is often diagnosed in	Q1. Could you describe the skin disease in this image for me?<br><br>A1. Rosacea, Solar dermatitis, Lupus, Cutaneous Lupus<br><br>Q2. You should write a paragraph to list more features you observed in the image.<br><br>A2. The facial erythema and papules, as a “butterfly rash”.<br><br>Q3. With previous information, please write a paragraph to explain in detail the cause of this skin disease.<br><br>A3. Lupus erythematosus is a multisystem disorder that prominently affects the skin. Cutaneous lesions are a source of disability and, on many occasions, an indicator of internal disease. The pathogenesis of cutaneous LE is complex, and it involves an interaction between genetic and environmental factors. The latter include ultraviolet radiation (UVR), medications, and possibly viruses. This interplay triggers a complex inflammatory cascade of cytokine, chemokine and inflammatory cell responses that include cells residing within as well as recruited to the skin.<br>Diagnosis was by histopathological examination and direct immunofluorescence (DIF).<br><br>Q4. How should I treat it? What medicine should I use?<br><br>A4. Sun protection is very important. For local

individuals with a family history of the disease and is more common in women, particularly those of African or Asian descent. Treatment options include topical steroids, sun avoidance, and systemic medications to suppress the immune system, such as corticosteroids, antimalarials, and biologics.<br><br>Q4. How should I treat it? What medicine should I use?<br><br>A4. The treatment of lupus erythematosus depends on the severity of the disease and the specific symptoms that are present. The primary treatment options include topical and systemic medications.<br>Topical medications:<br>* Corticosteroids: Reduce inflammation and are applied directly to the skin.<br>* Tacrolimus and pimecrolimus: Immunosuppressants that are applied topically to reduce inflammation and suppress the immune system.<br>Systemic medications:<br>* Corticosteroids: Prescribed to reduce inflammation and suppress the immune system.<br>* Antimalarials: Drugs that are used to treat lupus erythematosus, such as hydroxychloroquine.<br>* Immunomodulatory agents: Medications that modify the immune system's response, such as azathioprine and mycophenolate mofetil.<br>* Biologic agents: A new class of medications that targets specific parts of the immune system, such as belimumab.<br>It is important to note that treatment options and recommendations may vary based on the severity and specific symptoms of the disease, and should be determined by a dermatologist or other healthcare professional.	therapy, we can use topical and intralesional corticosteroids, calcineurin inhibitors and retinoids. For systemic therapy, we can use antimalarial drugs, Retinoids, Thalidomide and Sulfasalazine when antimalarial-resistant.
Evaluation of Physician	
Evaluation Item	Score (1-5, the higher the better)
SkinGPT-4's diagnosis is correct or relevant.	5
SkinGPT-4's description is informative.	5
SkinGPT-4's suggestions are useful.	5

SkinGPT-4 can help doctors with diagnosis.	5
SkinGPT-4 can help patients to understand their disease better.	5
If SkinGPT-4 can be deployed locally, it protects patients' privacy.	5
Willingness to use SkinGPT-4	5

Case Lupus 10

Image ID: Lupus10	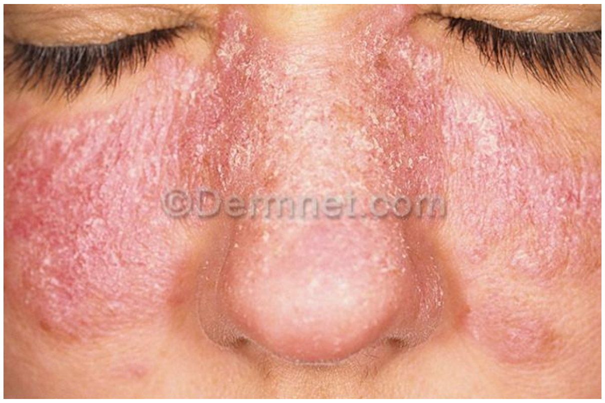
SkinGPT-4	Dermatology Physician
Q1. Could you describe the skin disease in this image for me?<br><br>A1. This could be a skin disease image of lupus chronic cutaneous.<br><br>Q2. You should write a paragraph to list more features you observed in the image.<br><br>A2. This could be a skin disease image of lupus chronic cutaneous. The rash is erythematous and has a scaly appearance. There may be photosensitivity, as the skin is sensitive to light. The lesions can be annular or irregular and can be found on sun exposed areas, such as the face, neck, and hands.<br><br>Q3. With previous information, please write a paragraph to explain in detail the cause of this skin disease.<br><br>A3. Lupus chronic cutaneous is a type of lupus erythematosus that affects the skin. It is caused by autoimmune disorders in which the immune system attacks healthy cells and tissues in the body, including the skin. The exact cause of lupus is not known, but genetics, environmental factors, and hormones may play a role. Symptoms include skin rashes, inflammation, and scarring, which can be triggered by sun exposure. The rashes can be treated with corticosteroids, sunscreens, and other immunosuppressive drugs. It is important to note that this condition is chronic and requires ongoing management and monitoring.	Q1. Could you describe the skin disease in this image for me?<br><br>A1. Lupus, Cutaneous Lupus<br><br>Q2. You should write a paragraph to list more features you observed in the image.<br><br>A2. The facial erythema, with greyish-white adhesive scales, as a “butterfly rash”.<br><br>Q3. With previous information, please write a paragraph to explain in detail the cause of this skin disease.<br><br>A3. Lupus erythematosus is a multisystem disorder that prominently affects the skin. Cutaneous lesions are a source of disability and, on many occasions, an indicator of internal disease. The pathogenesis of cutaneous LE is complex, and it involves an interaction between genetic and environmental factors. The latter include ultraviolet radiation (UVR), medications, and possibly viruses. This interplay triggers a complex inflammatory cascade of cytokine, chemokine and inflammatory cell responses that include cells residing within as well as recruited to the skin.<br>Diagnosis was by histopathological examination and direct immunofluorescence (DIF).<br><br>Q4. How should I treat it? What medicine should I use?<br><br>A4. Sun protection is very important. For local

Q4. How should I treat it? What medicine should I use?<br><br>A4. The treatment of lupus chronic cutaneous depends on the severity and location of the rash. Mild cases can be treated with topical corticosteroids, antimalarials, or immunosuppressive medications, while more severe cases may require oral corticosteroids or immunosuppressive drugs. The use of sunscreens and avoiding sun exposure is essential in managing this condition. In addition, medications such as hydroxychloroquine, mycophenolate mofetil, and belimumab are used to control the immune system's response and reduce inflammation. It is important to consult a dermatologist or a rheumatologist for proper diagnosis and treatment.	therapy, we can use topical and intralesional corticosteroids, calcineurin inhibitors and retinoids. For systemic therapy, we can use antimalarial drugs, Retinoids, Thalidomide and Sulfasalazine when antimalarial-resistant.

Evaluation of Physician

Evaluation Item	Score (1-5, the higher the better)
SkinGPT-4's diagnosis is correct or relevant.	5
SkinGPT-4's description is informative.	5
SkinGPT-4's suggestions are useful.	5
SkinGPT-4 can help doctors with diagnosis.	5
SkinGPT-4 can help patients to understand their disease better.	5
If SkinGPT-4 can be deployed locally, it protects patients' privacy.	5
Willingness to use SkinGPT-4	5

Case Seborrheic Keratoses 1

Image ID: Seborrheic Keratoses1	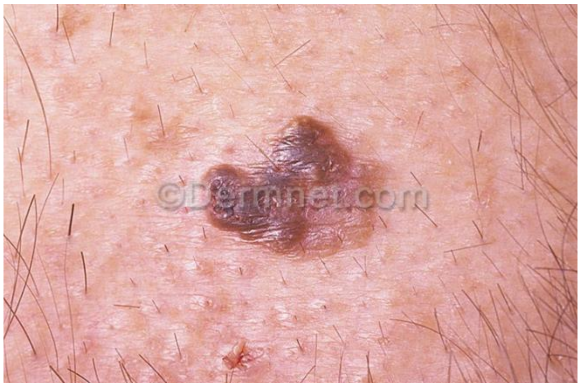
**SkinGPT-4**	**Dermatology Physician**
Q1. Could you describe the skin disease in this image for me?<br><br>A1. This could be a skin disease image of seborrheic keratoses smooth.<br><br>Q2. You should write a paragraph to list more features you observed in the image.<br><br>A2. This could be a skin disease image of seborrheic keratoses smooth. They are common benign skin growths that can appear waxy or scaly, and have a brown or black color. They are often found on the face, chest, and back, and can be removed easily with sharp instruments or cryotherapy.<br><br>Q3. With previous information, please write a paragraph to explain in detail the cause of this skin disease.<br><br>A3. Seborrheic keratoses are thought to be caused by a combination of genetics and environmental factors. They are commonly found in people over the age of 30 and are more common in people with a fair complexion. The exact cause of seborrheic keratoses is not known, but it is believed to be related to excessive cell proliferation and differentiation. They are usually benign and do not require treatment, but can be removed for cosmetic purposes.<br><br>Q4. How should I treat it? What medicine should I use?	Q1. Could you describe the skin disease in this image for me?<br><br>A1. Seborrheic keratoses; Melanoma<br><br>Q2. You should write a paragraph to list more features you observed in the image.<br><br>A2. Smooth surface, brown, irregularly rounded, flat papules.<br><br>Q3. With previous information, please write a paragraph to explain in detail the cause of this skin disease.<br><br>A3. (1) SK is a common benign lesions that typically begin to appear during the fourth decade of life. (2) Solitary or multiple, tan to black, macular, papular or verrucous lesions. (3) May occur anywhere except mucous membranes, palms and soles. (4) Clinical appearance of SK is in large variation and may simulate melanocytic neoplasms. (5) If the SK has undergone an abrupt increase in size or number (Leser-Trélat sign), it is associated with an internal malignancy.<br><br>Q4. How should I treat it? What medicine should I use?<br><br>A4. The first step is a dermoscopy for the exclusion of melanoma. Treatment of asymptomatic SKs is largely performed for cosmetic reasons. The most common method of destruction is cryotherapy. Other methods include laser vaporization or electrodesiccation.

A4. Seborrheic keratoses are usually harmless and do not require treatment. However, if the growth bothers the person, it can be removed with simple office procedures such as cryotherapy, curettage, or shave excision. The most common treatment for seborrheic keratoses is cryotherapy which involves freezing the growth with liquid nitrogen. The procedure is relatively painless and can be done in a dermatologist's office. The area may be slightly red and swollen after the procedure, but it usually resolves within a few days. It is important to note that after the treatment, the keratosis will return, and it will require more treatments in the future.	

Evaluation of Physician	
Evaluation Item	Score (1-5, the higher the better)
SkinGPT-4's diagnosis is correct or relevant.	5
SkinGPT-4's description is informative.	5
SkinGPT-4's suggestions are useful.	5
SkinGPT-4 can help doctors with diagnosis.	5
SkinGPT-4 can help patients to understand their disease better.	5
If SkinGPT-4 can be deployed locally, it protects patients' privacy.	5
Willingness to use SkinGPT-4	5

Case Seborrheic Keratoses 2

Image ID: Seborrheic Keratoses2	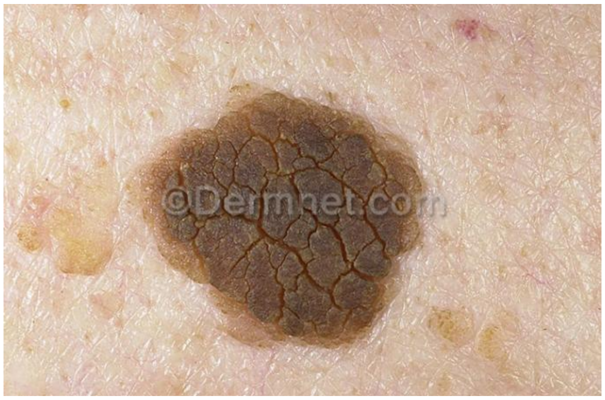
SkinGPT-4	Dermatology Physician
Q1. Could you describe the skin disease in this image for me?<br><br>A1. This could be a skin disease image of seborrheic keratoses ruff.<br><br>Q2. You should write a paragraph to list more features you observed in the image.<br><br>A2. The image shows a seborrheic keratosis, a common benign skin growth. It presents as a smooth, waxy, dark brown to black patches with sharp margins on the face, neck, and trunk. It can also be found on mucous membranes, such as the conjunctiva. The size can vary from a few millimeters to several centimeters. It is not painful but can be cosmetically bothersome. The underlying skin is not affected, and it does not have a clinically significant risk of malignant transformation. It is more common in people with fair skin and light hair, and it tends to run in families.<br><br>Q3. With previous information, please write a paragraph to explain in detail the cause of this skin disease.<br><br>A3. Seborrheic keratoses are caused by the accumulation of dead skin cells on the skin surface, which results in the formation of a waxy, raised lesion. The exact cause of this accumulation of dead skin cells is not fully understood, but it is thought to be related to hormonal changes, skin aging, and sun exposure. Seborrheic keratoses are more common in people	Q1. Could you describe the skin disease in this image for me?<br><br>A1. Seborrheic keratoses.<br><br>Q2. You should write a paragraph to list more features you observed in the image.<br><br>A2. A brown irregularly rounded flat papules with a rough surface.<br><br>Q3. With previous information, please write a paragraph to explain in detail the cause of this skin disease.<br><br>A3. (1) SK is a common benign lesion that typically begin to appear during the fourth decade of life. (2) Solitary or multiple, tan to black, macular, papular or verrucous lesions. (3) May occur anywhere except mucous membranes, palms and soles. (4) Clinical appearance of SK is in large variation and may simulate melanocytic neoplasms. (5) If the SK has undergone an abrupt increase in size or number (Leser-Trélat sign), it is associated with an internal malignancy.<br><br>Q4. How should I treat it? What medicine should I use?<br><br>A4. The first step is a dermoscopy for the exclusion of melanoma. Treatment of asymptomatic SKs is largely performed for cosmetic reasons. The most common method of destruction is cryotherapy. Other methods include laser vaporization or electrodesiccation.

with fair skin and light hair, and it tends to run in families, suggesting a genetic component. These lesions are benign and do not turn into skin cancer, but they can be mistaken for skin cancer due to their appearance, so it is important to have them evaluated by a dermatologist when found.<br><br>Q4. How should I treat it? What medicine should I use?<br><br>A4. Seborrheic keratoses are benign and do not require treatment, but some people may choose to treat them for cosmetic reasons or because they are worried about their potential to become cancerous. Treatment options for seborrheic keratoses include:<br>* Cryotherapy: Using liquid nitrogen to freeze the lesion and remove it from the skin<br>* Curettage: Scraping the lesion with a curette to remove it from the skin<br>* Surgical excision: Removing the lesion with surgical instruments, such as a scalpel or laser<br>* Chemical peels: Using a chemical solution to remove the lesion<br>* Dermabrasion: Removing the top layer of skin with a mechanical device<br><br>It is important to note that these treatments may cause scarring, and it is best to talk to a dermatologist about the best treatment option for your individual case.	
Evaluation of Physician	
Evaluation Item	Score (1-5, the higher the better)
SkinGPT-4’s diagnosis is correct or relevant.	5
SkinGPT-4’s description is informative.	5
SkinGPT-4’s suggestions are useful.	5
SkinGPT-4 can help doctors with diagnosis.	5
SkinGPT-4 can help patients to understand their disease better.	5
If SkinGPT-4 can be deployed locally, it protects patients’ privacy.	5

Willingness to use SkinGPT-4	5

Case Seborrheic Keratoses 3

Image ID: Seborrheic Keratoses3	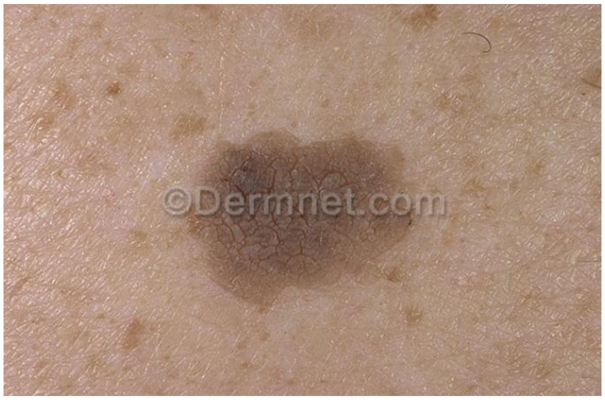
SkinGPT-4	Dermatology Physician
Q1. Could you describe the skin disease in this image for me?<br><br>A1. This could be a skin disease image of seborrheic keratoses ruff.<br><br>Q2. You should write a paragraph to list more features you observed in the image.<br><br>A2. The image shows a seborrheic keratosis, which is a common benign skin lesion that affects middle aged and older adults. It presents as a brown to black macule or plaque with a greasy and adherent scale. The scale is not sharply demarcated from the surrounding normal skin and can vary in size from a few millimeters to several centimeters. The surface of the lesion is often rough and can be covered with whitish scales. The borders of the lesion are well defined and the surrounding skin is normal. This image is a skin disease image of seborrheic keratosis.<br><br>Q3. With previous information, please write a paragraph to explain in detail the cause of this skin disease.<br><br>A3. Seborrheic keratosis is caused by the accumulation of dead skin cells on the skin surface. These cells form a scaly, waxy layer that gives the skin a yellow-brown color. The exact cause of this accumulation is not known, but it is believed to be related to hormonal changes, sun exposure, and aging. Seborrheic keratosis is more common in people with fair skin and it tends to run in families. The condition is benign and not	Q1. Could you describe the skin disease in this image for me?<br><br>A1. Seborrheic keratoses.<br><br>Q2. You should write a paragraph to list more features you observed in the image.<br><br>A2. An irregularly rounded, flat papule with a rough surface.<br><br>Q3. With previous information, please write a paragraph to explain in detail the cause of this skin disease.<br><br>A3. (1) SK is a common benign lesions that typically begin to appear during the fourth decade of life. (2) Solitary or multiple, tan to black, macular, papular or verrucous lesions. (3) May occur anywhere except mucous membranes, palms and soles. (4) Clinical appearance of SK is in large variation and may simulate melanocytic neoplasms. (5) If the SK has undergone an abrupt increase in size or number (Leser-Trélat sign), it is associated with an internal malignancy.<br><br>Q4. How should I treat it? What medicine should I use?<br><br>A4. The first step is a dermoscopy for the exclusion of melanoma. Treatment of asymptomatic SKs is largely performed for cosmetic reasons. The most common method of destruction is cryotherapy. Other methods include laser vaporization or electrodesiccation.

contagious, but it can be confused with other skin lesions such as warts or skin cancer, so it is important to have it evaluated by a dermatologist. The lesions can be treated with cryotherapy, curettage, or electrodessication, or they can be left untreated as they are usually benign and do not cause any harm.<br><br>Q4. How should I treat it? What medicine should I use?<br><br>A4. Seborrheic keratosis is a benign skin condition and generally does not require treatment. However, if the lesion is causing discomfort, such as itching or burning, or if it is interfering with daily activities, treatment may be considered. The most common treatment options for seborrheic keratosis are cryotherapy, curettage, or electrodessication. Cryotherapy involves the use of liquid nitrogen to freeze the lesion, curettage is the removal of the lesion with a sharp instrument, and electrodessication is the use of an electrical current to remove the lesion. These treatments can be done in a dermatologist's office and are generally well tolerated, but they may cause some discomfort, redness, or swelling.<br><br>There is no medicine that can prevent seborrheic keratosis, but you can use some moisturizers, creams or ointments to help reduce the symptoms such as itching or irritation caused by the lesion. These over the counter products usually contain hydrocortisone, which is a steroid that can reduce inflammation and itching.	
Evaluation of Physician	
Evaluation Item	Score (1-5, the higher the better)
SkinGPT-4's diagnosis is correct or relevant.	5
SkinGPT-4's description is informative.	5
SkinGPT-4's suggestions are useful.	4
SkinGPT-4 can help doctors with diagnosis.	5

SkinGPT-4 can help patients to understand their disease better.	4
If SkinGPT-4 can be deployed locally, it protects patients’ privacy.	5
Willingness to use SkinGPT-4	5

Case Seborrheic Keratoses 4

Image ID: Seborrheic Keratoses4	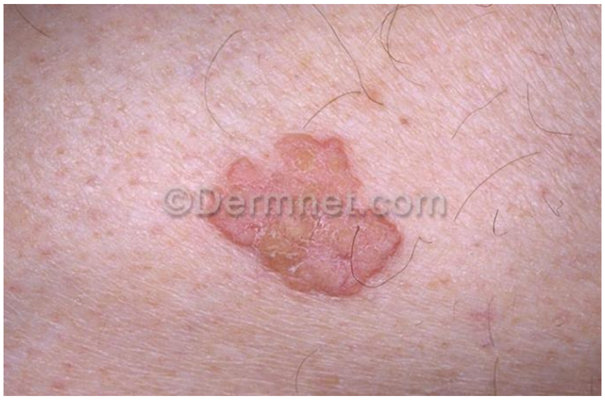
SkinGPT-4	Dermatology Physician
Q1. Could you describe the skin disease in this image for me?<br><br>A1. This could be a skin disease image of seborrheic keratoses smooth.<br><br>Q2. You should write a paragraph to list more features you observed in the image.<br><br>A2. The skin lesion presented in this image is a seborrheic keratosis. It is a common benign skin growth that affects people of all ages. It typically presents as a rough, brownish-grey, waxy patch on the skin, often on the face, neck, and hands. It can vary in size from a few millimeters to several centimeters and has a sharply defined border. The surface of the seborrheic keratosis is usually smooth and slightly elevated. The color of the lesion can range from brown to black, and it may have a waxy or greasy appearance. It is important to note that seborrheic keratosis is a benign condition and does not usually cause any symptoms, but it can be cosmetically bothersome.<br><br>Q3. With previous information, please write a paragraph to explain in detail the cause of this skin disease.<br><br>A3. Seborrheic keratosis is caused by the accumulation of dead skin cells that form a scale on the skin's surface. This accumulation of cells is due to the activity of the skin's oil glands, called sebaceous glands, which produce sebum to moisturize the skin. As people age, the skin's ability to shed dead skin cells decreases, causing	Q1. Could you describe the skin disease in this image for me?<br><br>A1. Skin tumor, Seborrheic keratoses<br><br>Q2. You should write a paragraph to list more features you observed in the image.<br><br>A2. Irregularly rounded, flat papules that are close in colour to the surrounding skin. The surface is rough.<br><br>Q3. With previous information, please write a paragraph to explain in detail the cause of this skin disease.<br><br>A3. (1) SK is a common benign lesions that typically begin to appear during the fourth decade of life. (2) Solitary or multiple, tan to black, macular, papular or verrucous lesions. (3) May occur anywhere except mucous membranes, palms and soles. (4) Clinical appearance of SK is in large variation and may simulate melanocytic neoplasms. (5) If the SK has undergone an abrupt increase in size or number (Leser-Trélat sign), it is associated with an internal malignancy.<br><br>Q4. How should I treat it? What medicine should I use?<br><br>A4. The first step is a dermoscopy for the exclusion of melanoma. Treatment of asymptomatic SKs is largely performed for cosmetic reasons. The most common method of destruction is cryotherapy. Other methods include

a buildup of cells that can form seborrheic keratoses. The exact cause of this decrease in skin cell shedding is not fully understood, but it may be related to hormonal changes, sun exposure, or skin inflammation. Seborrheic keratoses are not contagious and are not related to skin cancer. They are a common benign skin condition that can appear at any time during adulthood and are more common in people with fair skin. They can be treated with various methods, including cryotherapy, curettage, and electrocautery.<br><br>Q4. How should I treat it? What medicine should I use?<br><br>A4. Seborrheic keratosis is a benign skin condition and does not usually require treatment unless it is causing discomfort or cosmetic concerns. However, if you would like to remove the seborrheic keratosis, there are several treatment options available. Cryotherapy, curettage, and electrocautery are common methods to remove seborrheic keratoses. Cryotherapy involves freezing the lesion with liquid nitrogen, curettage involves scraping the lesion with a curette, and electrocautery involves using an electrical current to remove the lesion.<br><br>It's important to note that these treatments are usually not covered by insurance, and the cost can vary depending on the location and the size of the lesion. Before starting any treatment, it's best to consult a dermatologist to determine the best option for you.<br><br>Also, over the counter medicines such as salicylic acid or hydrocortisone cream can be used to help remove the seborrheic keratosis. But, it's important to use these creams for a short period of time and only on small lesions.<br>It's important to note that these treatments can cause skin irritation, and it's important to follow the instructions provided by your dermatologist or the product's manufacturer.	laser vaporization or electrodesiccation.
Evaluation of Physician	
Evaluation Item	Score (1-5, the higher the better)

SkinGPT-4's diagnosis is correct or relevant.	5
SkinGPT-4's description is informative.	5
SkinGPT-4's suggestions are useful.	4
SkinGPT-4 can help doctors with diagnosis.	5
SkinGPT-4 can help patients to understand their disease better.	4
If SkinGPT-4 can be deployed locally, it protects patients' privacy.	5
Willingness to use SkinGPT-4	5

Case Seborrheic Keratoses 5

Image ID: Seborrheic Keratoses5	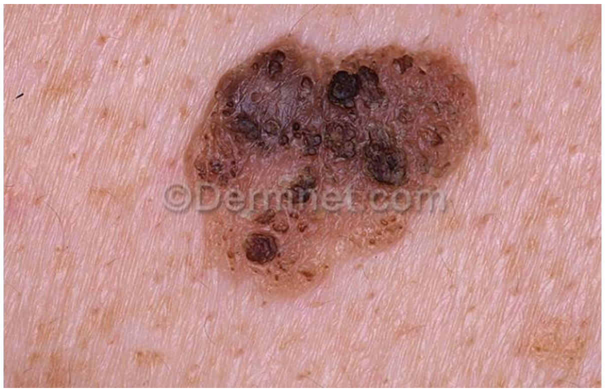
SkinGPT-4	Dermatology Physician
Q1. Could you describe the skin disease in this image for me?<br><br>A1. This could be a skin disease image of seborrheic keratoses ruff.<br><br>Q2. You should write a paragraph to list more features you observed in the image.<br><br>A2. The image is of a seborrheic keratosis, a common benign skin growth that can present as a solitary lesion or in clusters. The lesion has a pearly or shiny surface and is often found on the face, neck, and trunk. It has a sharply defined, irregular border and can vary in color from tan to brown to black. The center of the lesion may have a white or yellowish surface. The size can range from a few millimeters to several centimeters. This type of keratosis is caused by the overproduction of keratin, which leads to the formation of a horny layer. It is not cancerous and does not usually require treatment, but can be removed for cosmetic purposes.<br><br>Q3. With previous information, please write a paragraph to explain in detail the cause of this skin disease.<br><br>A3. Seborrheic keratosis is a benign skin growth that is caused by the overproduction of keratin, a protein that makes up the skin's outer layer. This overproduction results in the formation of a horny layer, which creates the characteristic pearly or shiny surface of the lesion. The exact cause of this overproduction is not known, but it is believed	Q1. Could you describe the skin disease in this image for me?<br><br>A1. Seborrheic keratoses<br><br>Q2. You should write a paragraph to list more features you observed in the image.<br><br>A2. Brown rounded flat papules with rough surface and black crusts(?)<br>.<br>Q3. With previous information, please write a paragraph to explain in detail the cause of this skin disease.<br><br>A3. (1) SK is a common benign lesions that typically begin to appear during the fourth decade of life. (2) Solitary or multiple, tan to black, macular, papular or verrucous lesions. (3) May occur anywhere except mucous membranes, palms and soles. (4) Clinical appearance of SK is in large variation and may simulate melanocytic neoplasms. (5) If the SK has undergone an abrupt increase in size or number (Leser-Trélat sign), it is associated with an internal malignancy.<br><br>Q4. How should I treat it? What medicine should I use?<br><br>A4. The first step is a dermoscopy for the exclusion of melanoma. Treatment of asymptomatic SKs is largely performed for cosmetic reasons. The most common method of destruction is cryotherapy. Other methods include laser vaporization or electrodesiccation.

<table>
<tr><td>to be related to aging, exposure to sunlight, and hormonal changes. Seborrheic keratosis is more common in people with fair skin and is not associated with an increased risk of skin cancer. It can occur as a solitary lesion or in clusters and is more common in people with a history of sun exposure. It is important to note that although seborrheic keratosis is benign, it can be mistaken for a skin cancer, so it is important to have any new or changing skin lesions evaluated by a dermatologist.

Q4. How should I treat it? What medicine should I use?

A4. Seborrheic keratosis is a benign skin growth and does not require treatment unless it is causing discomfort or cosmetic concerns. However, if the lesion is causing discomfort or bleeding, it can be removed by a dermatologist using techniques such as cryotherapy, curettage, or shave excision. These methods remove the lesion by removing the horny layer and the top layer of skin. These methods are usually done in the dermatologist’s office and do not require anesthesia.
If the lesion is causing cosmetic concerns, it can be removed for cosmetic purposes. However, it is important to note that removing a seborrheic keratosis may result in the formation of a new lesion.
There is no medication that can remove seborrheic keratosis, but some topical medications such as salicylic acid, lactic acid, or glycolic acid can help to improve the appearance of the lesion by removing the outer layer of the skin. These medications can be applied to the skin up to two times a day for a few weeks. It is important to follow the instructions of the dermatologist or the pharmacist.
Please keep in mind that self-treatment with over-the-counter medications can be dangerous and cause more harm than good. It is important to consult.</td><td></td></tr>
<tr><td colspan="2">Evaluation of Physician</td></tr>
<tr><td>Evaluation Item</td><td>Score (1-5, the higher the better)</td></tr>
<tr><td>SkinGPT-4’s diagnosis is correct or relevant.</td><td>5</td></tr>
</table>

SkinGPT-4's description is informative.	5
SkinGPT-4's suggestions are useful.	4
SkinGPT-4 can help doctors with diagnosis.	5
SkinGPT-4 can help patients to understand their disease better.	5
If SkinGPT-4 can be deployed locally, it protects patients' privacy.	5
Willingness to use SkinGPT-4	5

Case Seborrheic Keratoses 6

Image ID: Seborrheic Keratoses6	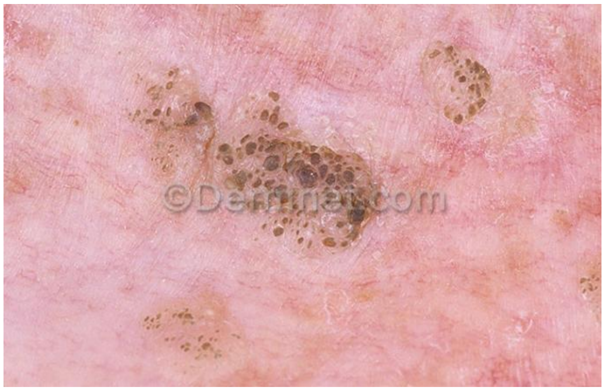
SkinGPT-4	Dermatology Physician
Q1. Could you describe the skin disease in this image for me?<br><br>A1. This could be a skin disease image of seborrheic keratoses smooth.<br><br>Q2. You should write a paragraph to list more features you observed in the image.<br><br>A2. In this image, there is a seborrheic keratosis lesion with a smooth surface. It has a tan color and is flat in appearance. The lesion has a sharp margin and is slightly elevated. The lesion is oval in shape with a diameter of approximately 5 mm. The surrounding skin is normal in appearance. There is no scaling, redness, or inflammation present. This lesion could be a seborrheic keratosis, which are benign skin growths that develop from keratin-producing cells. They are common and can appear waxy or scaly, brown or black, and may have a rough or smooth surface. They can range in size from a few millimeters to several centimeters and can appear on any part of the body, but are most commonly found on the face, neck, and trunk. Seborrheic keratoses are benign and do not require treatment unless they are painful, itchy, or cause some other bothersome symptoms.<br><br>Q3. With previous information, please write a paragraph to explain in detail the cause of this skin disease.<br><br>A3. Seborrheic keratosis is caused by the accumulation of keratin, a protein that makes up	Q1. Could you describe the skin disease in this image for me?<br><br>A1. Skin tumor; seborrheic keratoses<br><br>Q2. You should write a paragraph to list more features you observed in the image.<br><br>A2. Flat papules with black spots and a rough surface.<br><br>Q3. With previous information, please write a paragraph to explain in detail the cause of this skin disease.<br><br>A3. (1) SK is a common benign lesions that typically begin to appear during the fourth decade of life. (2) Solitary or multiple, tan to black, macular, papular or verrucous lesions. (3) May occur anywhere except mucous membranes, palms and soles. (4) Clinical appearance of SK is in large variation and may simulate melanocytic neoplasms. (5) If the SK has undergone an abrupt increase in size or number (Leser-Trélat sign), it is associated with an internal malignancy.<br><br>Q4. How should I treat it? What medicine should I use?<br><br>A4. The first step is a dermoscopy for the exclusion of melanoma. Treatment of asymptomatic SKs is largely performed for cosmetic reasons. The most common method of destruction is cryotherapy. Other methods include laser vaporization or electrodesiccation.

the outer layer of skin, on the skin's surface. This accumulation can occur due to aging, sun exposure, or other factors that disrupt the normal shedding of skin cells. As keratin builds up, it forms a rough, scaly surface on the skin.<br>Seborrheic keratosis is a benign condition and is not associated with any underlying disease or serious health problems. It is common in people over the age of 40 and is more prevalent in people with fair skin. It can appear waxy or scaly, brown or black, and may have a rough or smooth surface. They can range in size from a few millimeters to several centimeters and can appear on any part of the body, but are most commonly found on the face, neck, and trunk. Seborrheic keratoses are benign and do not require treatment unless they are painful, itchy, or cause some other bothersome symptoms.<br><br>Q4. How should I treat it? What medicine should I use?<br><br>A4. Seborrheic keratosis is a benign condition and does not require treatment unless it is causing bothersome symptoms such as itching or pain. However, if the lesion is causing any symptoms, it can be treated with topical medications, cryotherapy or curettage.<br>Topical medications: The most commonly used topical medication for seborrheic keratosis is 10% salicylic acid. It helps to peel off the outer layers of the skin and remove the lesion. It is applied to the skin 1-2 times a day for several weeks or until the lesion disappears.<br>Cryotherapy: This method involves freezing the lesion with liquid nitrogen. It is a quick and effective method to remove the lesion.<br>Curettage: This method involves scraping the lesion with a curette. It is performed under local anesthesia and is less painful than cryotherapy.<br>It is always best to consult with a dermatologist for a proper diagnosis and treatment plan. They may also prescribe medication such as fluorouracil or imiquimod which can be used in conjunction with the above methods.	
Evaluation of Physician	
Evaluation Item	Score (1-5, the higher the better)

SkinGPT-4's diagnosis is correct or relevant.	5
SkinGPT-4's description is informative.	5
SkinGPT-4's suggestions are useful.	4
SkinGPT-4 can help doctors with diagnosis.	5
SkinGPT-4 can help patients to understand their disease better.	5
If SkinGPT-4 can be deployed locally, it protects patients' privacy.	5
Willingness to use SkinGPT-4	5

Case Seborrheic Keratoses 7

<table>
<tr><td>Image ID: Seborrheic Keratoses7</td><td>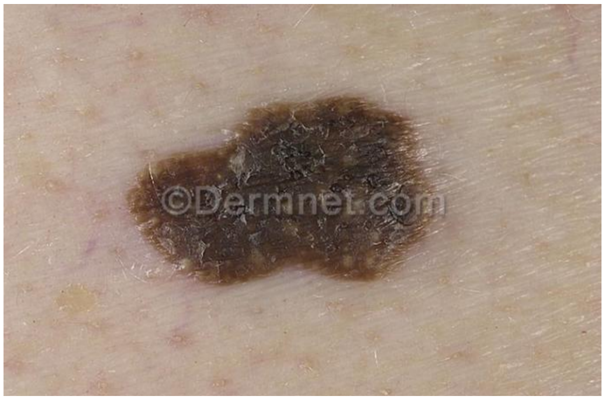
</td></tr>
<tr><th>SkinGPT-4</th><th>Dermatology Physician</th></tr>
<tr><td>Q1. Could you describe the skin disease in this image for me?

A1. This could be a skin disease image of seborrheic keratoses smooth.

Q2. You should write a paragraph to list more features you observed in the image.

A2. The image shows a seborrheic keratosis, which is a common benign skin growth. The lesion has a brown, warty surface with a central keratotic plug. It is flat or slightly elevated and has a sharp margin. The background skin is not inflamed or scaling. It measures about 5 mm in diameter.

Q3. With previous information, please write a paragraph to explain in detail the cause of this skin disease.

A3. Seborrheic keratosis is a common benign skin growth that is caused by the accumulation of dead skin cells on the skin surface. It is not known exactly what triggers the accumulation of these cells, but it is believed to be related to hormonal and immune system changes that occur with aging. Seborrheic keratoses are more common in people with fair skin and are rare in Africa and Asia. They can appear on any part of the body, but they are most commonly found on the face, neck, and trunk. Seborrheic keratoses are usually asymptomatic and do not require treatment, but they can be removed for cosmetic purposes. However, it is important to differentiate seborrheic keratosis from skin cancer because they can look</td><td>Q1. Could you describe the skin disease in this image for me?

A1. Seborrheic keratoses

Q2. You should write a paragraph to list more features you observed in the image.

A2. Oval-shaped, black, flat papules with a rough surface.

Q3. With previous information, please write a paragraph to explain in detail the cause of this skin disease.

A3. (1) SK is a common benign lesions that typically begin to appear during the fourth decade of life. (2) Solitary or multiple, tan to black, macular, papular or verrucous lesions. (3) May occur anywhere except mucous membranes, palms and soles. (4) Clinical appearance of SK is in large variation and may simulate melanocytic neoplasms. (5) If the SK has undergone an abrupt increase in size or number (Leser-Trélat sign), it is associated with an internal malignancy.

Q4. How should I treat it? What medicine should I use?

A4. The first step is a dermoscopy for the exclusion of melanoma. Treatment of asymptomatic SKs is largely performed for cosmetic reasons. The most common method of destruction is cryotherapy. Other methods include laser vaporization or electrodesiccation.</td></tr>
</table>

similar. A biopsy is sometimes necessary to confirm the diagnosis.<br><br>Q4. How should I treat it? What medicine should I use?<br><br>A4. Seborrheic keratoses are benign and do not require treatment, but if they become bothersome, they can be removed for cosmetic purposes. The most common method of removal is by cryotherapy, which involves freezing the lesion with liquid nitrogen. This method is relatively painless and effective, but it may require multiple treatments to completely remove the lesion. Other methods of removal include curettage, electrocautery, and surgical excision. However, these methods are more invasive and may have a longer recovery time.	

Evaluation of Physician	
Evaluation Item	Score (1-5, the higher the better)
SkinGPT-4's diagnosis is correct or relevant.	5
SkinGPT-4's description is informative.	5
SkinGPT-4's suggestions are useful.	5
SkinGPT-4 can help doctors with diagnosis.	5
SkinGPT-4 can help patients to understand their disease better.	5
If SkinGPT-4 can be deployed locally, it protects patients' privacy.	5
Willingness to use SkinGPT-4	5

Case Seborrheic Keratoses 8

Image ID: Seborrheic Keratoses8	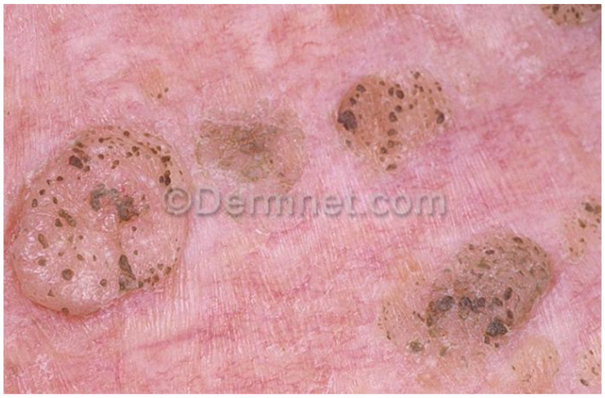
SkinGPT-4	Dermatology Physician
Q1. Could you describe the skin disease in this image for me?<br><br>A1. This could be a skin disease image of seborrheic keratoses smooth.<br><br>Q2. You should write a paragraph to list more features you observed in the image.<br><br>A2. In this image, there is a seborrheic keratosis lesion with a smooth surface. The lesion is light brown in color and has a distinct, irregular border. The surrounding skin appears normal. There are no visible lesions or skin eruptions nearby. The image is a close-up view of a single lesion. The size of the lesion is approximately 5 mm in diameter. No skin tears, blisters, or scabs are visible. No pustules, erythema, or edema are present. No lymphadenopathy or skin changes consistent with irritation or inflammation are visible.<br><br>Q3. With previous information, please write a paragraph to explain in detail the cause of this skin disease.<br><br>A3. This image shows a seborrheic keratosis, which is a common benign skin growth. It is caused by excessive growth of skin cells in the epidermis, typically due to sun exposure, aging, or genetics. Seborrheic keratoses are characterized by their smooth, waxy surface and distinct, irregular borders. They can appear in a variety of colors, including light brown, black, and white, and can range in size from small to large. They are not	Q1. Could you describe the skin disease in this image for me?<br><br>A1. seborrheic keratoses.<br><br>Q2. You should write a paragraph to list more features you observed in the image.<br><br>A2. Papules of the same colour as the skin, which may be rough with black spots on the surface<br><br>Q3. With previous information, please write a paragraph to explain in detail the cause of this skin disease.<br><br>A3. (1) SK is a common benign lesions that typically begin to appear during the fourth decade of life. (2) Solitary or multiple, tan to black, macular, papular or verrucous lesions. (3) May occur anywhere except mucous membranes, palms and soles. (4) Clinical appearance of SK is in large variation and may simulate melanocytic neoplasms. (5) If the SK has undergone an abrupt increase in size or number (Leser-Trélat sign), it is associated with an internal malignancy.<br><br>Q4. How should I treat it? What medicine should I use?<br><br>A4. The first step is a dermoscopy for the exclusion of melanoma. Treatment of asymptomatic SKs is largely performed for cosmetic reasons. The most common method of destruction is cryotherapy. Other methods include laser vaporization or electrodesiccation.

contagious and usually do not require treatment, but can be removed by a dermatologist if desired. The smooth surface of this seborrheic keratosis indicates that it is a type known as a warty seborrheic keratosis. These tend to be more common in men and are often found on the face, neck, and trunk.<br><br>Q4. How should I treat it? What medicine should I use?<br><br>A4. Seborrheic keratoses are generally harmless and do not require treatment. However, if the growth bothers you, you can have it removed by a dermatologist. The dermatologist can remove it by cutting it off with a scalpel or by using a laser to remove it. There is no medicine or cream that can remove a seborrheic keratosis.<br>Please note that this information is not intended to be a substitute for professional medical advice, diagnosis, or treatment. Always seek the advice of a qualified healthcare provider with any questions you may have regarding a medical condition.	
Evaluation of Physician	
Evaluation Item	Score (1-5, the higher the better)
SkinGPT-4's diagnosis is correct or relevant.	5
SkinGPT-4's description is informative.	4
SkinGPT-4's suggestions are useful.	4
SkinGPT-4 can help doctors with diagnosis.	5
SkinGPT-4 can help patients to understand their disease better.	5
If SkinGPT-4 can be deployed locally, it protects patients' privacy.	5
Willingness to use SkinGPT-4	5

Case Seborrheic Keratoses 9

Image ID: Seborrheic Keratoses9	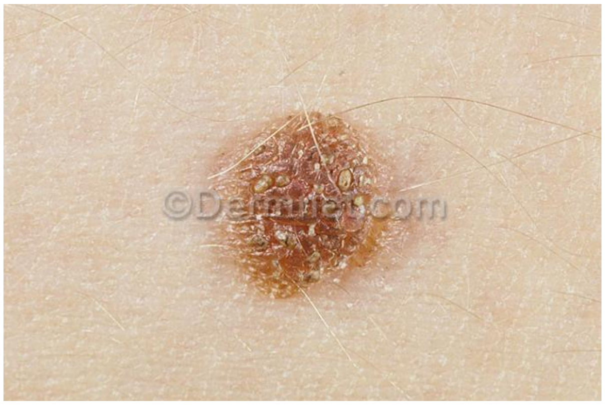
SkinGPT-4	Dermatology Physician
Q1. Could you describe the skin disease in this image for me?<br><br>A1. This could be a skin disease image of seborrheic keratoses smooth.<br><br>Q2. You should write a paragraph to list more features you observed in the image.<br><br>A2. The image shows a seborrheic keratosis, which is a common benign skin growth. It presents as a smooth, brownish-black, flat or slightly raised patch on the skin. It often occurs on the face, neck, and trunk and can be found in both men and women. Seborrheic keratoses are benign and do not typically cause any discomfort or pain, but some people may find them cosmetically bothersome. They are commonly seen in individuals over the age of 40 and their exact cause is unknown, but heredity and sun exposure are believed to play a role in their development.<br><br>Q3. With previous information, please write a paragraph to explain in detail the cause of this skin disease.<br><br>A3. Seborrheic keratoses are benign skin growths that are caused by a combination of genetics and environmental factors, particularly sun exposure. They are caused by the accumulation of dead skin cells on the surface of the skin, which form a scaly, crusty layer. This build-up of dead skin cells is thought to be triggered by hormonal changes, particularly androgen hormones, and by sun exposure, which causes the skin to produce more	Q1. Could you describe the skin disease in this image for me?<br><br>A1. seborrheic keratoses (?); Actinic Keratosis (?); Melanoma(?)<br><br>Q2. You should write a paragraph to list more features you observed in the image.<br><br>A2. Brown papules with a scaly surface.<br><br>Q3. With previous information, please write a paragraph to explain in detail the cause of this skin disease.<br><br>A3. (1) SK is a common benign lesions that typically begin to appear during the fourth decade of life. (2) Solitary or multiple, tan to black, macular, papular or verrucous lesions. (3) May occur anywhere except mucous membranes, palms and soles. (4) Clinical appearance of SK is in large variation and may simulate melanocytic neoplasms. (5) If the SK has undergone an abrupt increase in size or number (Leser-Trélat sign), it is associated with an internal malignancy.<br><br>Q4. How should I treat it? What medicine should I use?<br><br>A4. The first step is a dermoscopy for the exclusion of melanoma. Treatment of asymptomatic SKs is largely performed for cosmetic reasons. The most common method of destruction is cryotherapy. Other methods include laser vaporization or electrodesiccation.

pigment. The exact reason why some people develop seborrheic keratoses and others do not is not well understood, but it is thought to be related to genetics and sun exposure. Seborrheic keratoses are more common in people who have a family history of the condition and in those who have had significant sun exposure over their lifetime.<br><br>Q4. How should I treat it? What medicine should I use?<br><br>A4. Seborrheic keratoses are benign and do not typically require treatment, as they do not cause any harm to the body. However, if the growth bothers you, there are several treatment options available. Cryotherapy, which involves freezing the growth with liquid nitrogen, is a common and effective treatment option. This method removes the top layer of the growth, causing it to fall off, revealing the new skin underneath. Other treatment options include curettage, which involves scraping the growth with a sharp instrument, and electrocautery, which involves using an electrical current to remove the growth. These methods are also effective, but may cause more discomfort and have a higher risk of scarring. In some cases, the growth may return after treatment. It's important to consult with a dermatologist to determine the best treatment option for you.	
Evaluation of Physician	
Evaluation Item	Score (1-5, the higher the better)
SkinGPT-4's diagnosis is correct or relevant.	5
SkinGPT-4's description is informative.	5
SkinGPT-4's suggestions are useful.	5
SkinGPT-4 can help doctors with diagnosis.	5
SkinGPT-4 can help patients to understand their disease better.	5
If SkinGPT-4 can be deployed locally, it protects patients' privacy.	5

Willingness to use SkinGPT-4	5

Case Seborrheic Keratoses 10

Image ID: Seborrheic Keratoses10	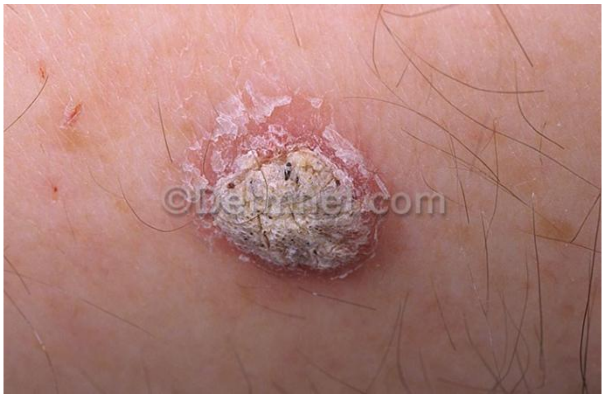
SkinGPT-4	Dermatology Physician
Q1. Could you describe the skin disease in this image for me?<br><br>A1. This could be a skin disease image of seborrheic keratosis irritated.<br><br>Q2. You should write a paragraph to list more features you observed in the image.<br><br>A2. This image depicts a seborrheic keratosis, which is a common benign skin growth. It presents as a brown to black papule with a rough, scaly surface. It often appears on the face, neck, and trunk in fair skinned individuals and can be induced by sun exposure. It can be mistaken for warts, actinic keratosis or skin cancer. Treatment options include cryotherapy, curettage, electrodesiccation, laser surgery and surgical excision.<br><br>Q3. With previous information, please write a paragraph to explain in detail the cause of this skin disease.<br><br>A3. Seborrheic keratosis is a common benign skin growth that is caused by the accumulation of dead skin cells on the skin surface. It is believed to be caused by the interaction of genetic and environmental factors. The exact cause is not known, but it is thought to be related to the aging process and the accumulation of dead skin cells on the skin surface. The sun exposure is also known to play a role in its development, as it tends to occur more frequently in fair skinned individuals. The risk is also increased by a family	Q1. Could you describe the skin disease in this image for me?<br><br>A1. seborrheic keratosis<br><br>Q2. You should write a paragraph to list more features you observed in the image.<br><br>A2. A Keratotic papules.<br><br>Q3. With previous information, please write a paragraph to explain in detail the cause of this skin disease.<br><br>A3. (1) SK is a common benign lesions that typically begin to appear during the fourth decade of life. (2) Solitary or multiple, tan to black, macular, papular or verrucous lesions. (3) May occur anywhere except mucous membranes, palms and soles. (4) Clinical appearance of SK is in large variation and may simulate melanocytic neoplasms. (5) If the SK has undergone an abrupt increase in size or number (Leser-Trélat sign), it is associated with an internal malignancy.<br><br>Q4. How should I treat it? What medicine should I use?<br><br>A4. The first step is a dermoscopy for the exclusion of melanoma. Treatment of asymptomatic SKs is largely performed for cosmetic reasons. The most common method of destruction is cryotherapy. Other methods include laser vaporization or electrodesiccation.

history of seborrheic keratoses and skin cancer.<br>There is no way to prevent the development of seborrheic keratoses, but avoiding sun exposure and using sunscreen may help to prevent new growths from developing.<br><br>Q4. How should I treat it? What medicine should I use?<br><br>A4. Seborrheic keratosis is a benign skin growth and generally does not require treatment. However, if the growth is bothersome, such as if it is painful, bleeding, or itching, or if it is interfering with daily activities, it can be treated. Treatment options for seborrheic keratosis include cryotherapy, curettage, electrodesiccation, laser surgery and surgical excision.<br>Cryotherapy is the use of cold liquid nitrogen to freeze the growth, which causes it to fall off.<br>Curettage is the use of a currette, a instrument to scrape the growth from the skin.<br>Electrodesiccation is the use of a small electric current to remove the growth by burning it.<br>Laser surgery uses a beam of light to remove the growth.<br>Surgical excision is the removal of the growth by surgery.<br>It is important to note that these treatments can cause scarring and pigmentation, and may not be suitable for everyone. It is always best to consult a dermatologist for the best treatment option for you.	
Evaluation of Physician	
Evaluation Item	Score (1-5, the higher the better)
SkinGPT-4's diagnosis is correct or relevant.	4
SkinGPT-4's description is informative.	5
SkinGPT-4's suggestions are useful.	5
SkinGPT-4 can help doctors with diagnosis.	5
SkinGPT-4 can help patients to understand their disease better.	5

If SkinGPT-4 can be deployed locally, it protects patients’ privacy.	5
Willingness to use SkinGPT-4	5

Case Melanoma 1

Image ID: Melanoma1	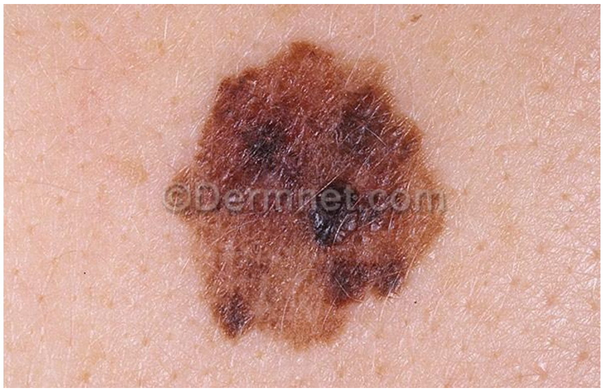
**SkinGPT-4**	**Dermatology Physician**
Q1. Could you describe the skin disease in this image for me?<br><br>A1. Melanoma Large Cell<br><br>Q2. You should write a paragraph to list more features you observed in the image.<br><br>A2. Melanoma is a type of skin cancer that arises from pigmented cells known as melanocytes. It can occur anywhere on the skin, but is most commonly found on the face, neck, and trunk of the body. Melanoma can occur in people of all skin types, but is more common in people with fair skin, light hair, and light eyes.<br><br>Q3. With previous information, please write a paragraph to explain in detail the cause of this skin disease.<br><br>A3. The main risk factors for melanoma include a family history of melanoma, a personal history of sunburns, and excessive exposure to UV radiation from the sun or tanning beds. Other factors that may increase the risk of melanoma include a weakened immune system, certain genetic mutations, and certain types of benign skin growths.<br><br>Q4. How should I treat it? What medicine should I use?<br><br>A4. The treatment for melanoma depends on the stage of the cancer. If the melanoma is caught early and has not spread beyond the original site,	Q1. Could you describe the skin disease in this image for me?<br><br>A1. Melanoma<br><br>Q2. You should write a paragraph to list more features you observed in the image.<br><br>A2. Brown patches of varying colour with black papules.<br><br>Q3. With previous information, please write a paragraph to explain in detail the cause of this skin disease.<br><br>A3. Melanoma represents a malignant tumor that arises from melanocytes, and, due to its metastatic potential, leads to >75% of skin cancer deaths. The incidence rates of melanoma have increased over the past four decades. In *situ* and early invasive cutaneous melanoma can be subtle in appearance but dermoscopy has led to an improvement in diagnostic accuracy. Early-stage melanomas are often curable by surgical excision. For metastatic melanoma, immunotherapies (e.g. ipilimumab) and<br>targeted therapies (e.g. vemurafenib) can be tried.<br><br>Q4. How should I treat it? What medicine should I use?<br><br>A4. First, a dermoscopy is required. Second, an extended excision of the skin lesion is often necessary. Further treatment is also based on the pathological findings after surgery and other

it may be treated with surgery to remove the cancer. If the melanoma has spread to the lymph nodes, it may be treated with surgery and/or adjuvant therapy, such as chemotherapy or immunotherapy. In more advanced stages of melanoma, a combination of these treatments may be used. In some cases, a clinical trial may be an option for treatment. It is important to discuss the best treatment options with a dermatologist or oncologist.	ancillary findings.
Evaluation of Physician	
Evaluation Item	Score (1-5, the higher the better)
SkinGPT-4's diagnosis is correct or relevant.	5
SkinGPT-4's description is informative.	5
SkinGPT-4's suggestions are useful.	5
SkinGPT-4 can help doctors with diagnosis.	5
SkinGPT-4 can help patients to understand their disease better.	5
If SkinGPT-4 can be deployed locally, it protects patients' privacy.	5
Willingness to use SkinGPT-4	5

Case Melanoma 2

Image ID: Melanoma2	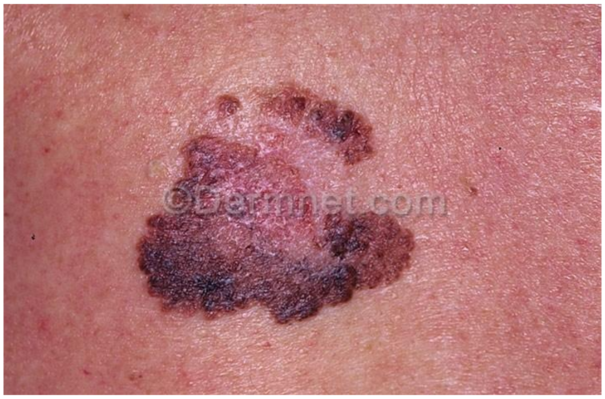
**SkinGPT-4**	**Dermatology Physician**
Q1. Could you describe the skin disease in this image for me?<br><br>A1. melanoma<br><br>Q2. You should write a paragraph to list more features you observed in the image.<br><br>A2. This is a picture of melanoma on the skin. It is a type of skin cancer that is caused by the growth of pigmented cells called melanocytes. Melanoma can occur on any part of the body, but it is most common on the skin that is exposed to the sun. The lesion in this photo is a typical example of melanoma, with a brownish black color and irregular borders. Melanoma can be either benign or malignant. Benign melanoma is not cancerous and does not spread to other parts of the body. Malignant melanoma is cancerous and can spread to other parts of the body, including the lymph nodes, lungs, and other organs. It is important to have any suspicious skin lesions evaluated by a dermatologist or other healthcare provider.<br><br>Q3. With previous information, please write a paragraph to explain in detail the cause of this skin disease.<br><br>A3. Melanoma is caused by the abnormal growth of melanocytes, which are the cells responsible for producing melanin pigment. Melanin is responsible for the color of the skin, hair, and eyes. Melanocytes can be found in the basal layer of the epidermis, the outermost layer of the skin.	Q1. Could you describe the skin disease in this image for me?<br><br>A1. melanoma.<br><br>Q2. You should write a paragraph to list more features you observed in the image.<br><br>A2. An irregular shape, brown patches of varying colour with black-thicken edge.<br><br>Q3. With previous information, please write a paragraph to explain in detail the cause of this skin disease.<br><br>A3. Melanoma represents a malignant tumor that arises from melanocytes, and, due to its metastatic potential, leads to >75% of skin cancer deaths. The incidence rates of melanoma have increased over the past four decades. In *situ* and early invasive cutaneous melanoma can be subtle in appearance but dermoscopy has led to an improvement in diagnostic accuracy. Early-stage melanomas are often curable by surgical excision. For metastatic melanoma, immunotherapies (e.g. ipilimumab) and targeted therapies (e.g. vemurafenib) can be tried.<br><br>Q4. How should I treat it? What medicine should I use?<br><br>A4. First, a dermoscopy is required. Second, an extended excision of the skin lesion is often necessary. Further treatment is also based on the pathological findings after surgery and other

The primary cause of melanoma is exposure to ultraviolet (UV) light from the sun or other sources. UV light causes damage to the DNA in skin cells, leading to the development of mutations that can cause cells to become cancerous. Other risk factors for melanoma include a family history of the disease, fair skin, red hair, and a large number of moles. People with a weakened immune system, such as those with HIV/AIDS, are also at increased risk of developing melanoma.<br><br>It is important to practice sun safety measures to reduce the risk of developing melanoma. This includes seeking shade, wearing protective clothing, and using a broad-spectrum sunscreen with an SPF of at least 30. Regular skin exams by a dermatologist or other healthcare provider can also help to detect melanoma early, when it is most treatable.<br><br>Q4. How should I treat it? What medicine should I use?<br><br>A4. Melanoma is typically treated with surgery to remove the cancerous mole or lesion. The surgery may be performed by a dermatologist or a general surgeon, depending on the location and size of the lesion. In some cases, the lesion may be removed in a process called excisional biopsy, where the entire lesion is removed along with some healthy tissue around it. In other cases, the lesion may be removed in a process called shave biopsy, where only the top layer of the lesion is removed.<br>In addition to surgery, other treatment options for melanoma may include immunotherapy, targeted therapy, and radiation therapy. These treatments are used to help prevent the cancer from spreading to other parts of the body and to improve the chances of a successful outcome.<br>The specific treatment plan for melanoma will depend on the stage of the cancer, the size and location of the lesion, and the patient's overall health.<br>It is important to follow the treatment plan recommended by your healthcare provider to ensure the best possible outcome. This may include regular follow-up appointments to monitor the progress of the treatment and to address any side effects. It is also important to practice sun safety measures to reduce the risk of developing melanoma or other types of skin cancer.	ancillary findings.

Evaluation of Physician	
Evaluation Item	Score (1-5, the higher the better)
SkinGPT-4's diagnosis is correct or relevant.	5
SkinGPT-4's description is informative.	3
SkinGPT-4's suggestions are useful.	2
SkinGPT-4 can help doctors with diagnosis.	4
SkinGPT-4 can help patients to understand their disease better.	5
If SkinGPT-4 can be deployed locally, it protects patients' privacy.	5
Willingness to use SkinGPT-4	3

Case Melanoma 3

Image ID: Melanoma3	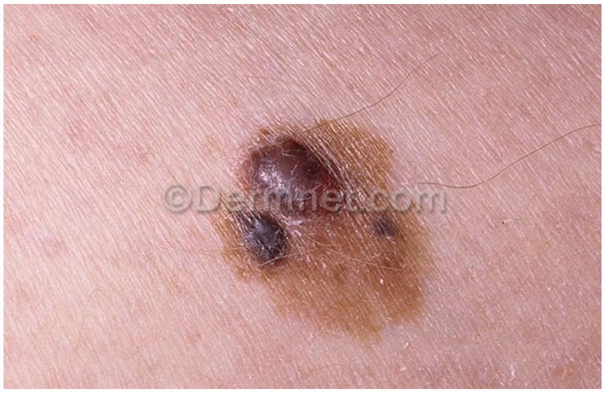
**SkinGPT-4**	**Dermatology Physician**
Q1. Could you describe the skin disease in this image for me?<br><br>A1. melanoma skin cancer<br><br>Q2. You should write a paragraph to list more features you observed in the image.<br><br>A2. The image shows a mole on the skin, which is a benign tumor consisting of cells that produce melanin and is found in the skin. Moles can be raised or flat and can be different colors, including brown, black, and tan. They can appear anywhere on the body, including the face, neck, and arms. Moles can be removed for various reasons, including cosmetic purposes or because they are potentially cancerous.<br><br>Q3. With previous information, please write a paragraph to explain in detail the cause of this skin disease.<br><br>A3. The cause of skin cancer, including melanoma, is largely due to exposure to ultraviolet (UV) radiation from the sun or tanning beds. This can cause mutations in the DNA of skin cells, leading to the development of cancer. Other risk factors for melanoma include having a family history of the disease, having a large number of moles, and having a weakened immune system. Melanoma can also occur in areas of the skin that have been previously injured or sunburned. It is important to protect the skin from UV radiation by using sunscreen, wearing protective clothing, and avoiding tanning beds. Regular skin exams can	Q1. Could you describe the skin disease in this image for me?<br><br>A1. Melanoma; Melanocytic nevus<br><br>Q2. You should write a paragraph to list more features you observed in the image.<br><br>A2. Brown patches of varying colour with black papules.<br><br>Q3. With previous information, please write a paragraph to explain in detail the cause of this skin disease.<br><br>A3. Melanoma represents a malignant tumor that arises from melanocytes, and, due to its metastatic potential, leads to >75% of skin cancer deaths. The incidence rates of melanoma have increased over the past four decades. In *situ* and early invasive cutaneous melanoma can be subtle in appearance but dermoscopy has led to an improvement in diagnostic accuracy. Early-stage melanomas are often curable by surgical excision. For metastatic melanoma, immunotherapies (e.g. ipilimumab) and targeted therapies (e.g. vemurafenib) can be tried.<br><br>Q4. How should I treat it? What medicine should I use?<br><br>A4. First, a dermoscopy is required. Second, an extended excision of the skin lesion is often necessary. Further treatment is also based on the pathological findings after surgery and other

also help to detect melanoma early, when it is most treatable.<br><br>Q4. How should I treat it? What medicine should I use?<br><br>A4. Treatment for melanoma skin cancer will depend on the stage and severity of the disease. Surgery is the most common treatment for melanoma, and may involve removing the tumor and some surrounding tissue. In some cases, lymph nodes may also be removed. For more advanced stages of melanoma, additional treatments such as immunotherapy, targeted therapy, or radiation therapy may be used. A dermatologist or oncologist can determine the best course of treatment based on the specific diagnosis and individual circumstances. Medications such as topical creams or oral medications may also be used to treat any symptoms or side effects associated with the disease. It is important to follow the treatment plan recommended by your healthcare provider to ensure the best possible outcome.	ancillary findings.
Evaluation of Physician	
Evaluation Item	Score (1-5, the higher the better)
SkinGPT-4's diagnosis is correct or relevant.	5
SkinGPT-4's description is informative.	5
SkinGPT-4's suggestions are useful.	4
SkinGPT-4 can help doctors with diagnosis.	5
SkinGPT-4 can help patients to understand their disease better.	5
If SkinGPT-4 can be deployed locally, it protects patients' privacy.	5
Willingness to use SkinGPT-4	5

Case Melanoma 4

Image ID: Melanoma4	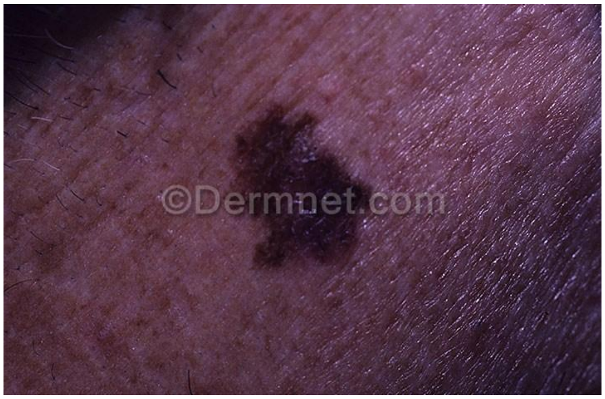
**SkinGPT-4**	**Dermatology Physician**
Q1. Could you describe the skin disease in this image for me?<br><br>A1. melanoma skin cancer<br><br>Q2. You should write a paragraph to list more features you observed in the image.<br><br>A2. The image shows a brown mole on the skin, which is a benign growth that can be removed for cosmetic purposes or if it becomes cancerous.<br><br>Q3. With previous information, please write a paragraph to explain in detail the cause of this skin disease.<br><br>A3. The cause of this skin disease is likely due to excessive exposure to the sun's harmful ultraviolet rays, which can cause mutations in the DNA of skin cells and lead to the formation of moles, some of which may develop into malignant melanoma. Other factors that can contribute to the development of melanoma include family history, fair skin, multiple moles, and a history of sunburns.<br><br>Q4. How should I treat it? What medicine should I use?<br><br>A4. The treatment for this skin disease will depend on the size, location, and severity of the mole. If the mole is small and not causing any symptoms, it may not require treatment. However, if the mole is large, changing in appearance, or causing symptoms such as itching, bleeding, or pain, a	Q1. Could you describe the skin disease in this image for me?<br><br>A1. Melanoma; Melanocytic nevus<br><br>Q2. You should write a paragraph to list more features you observed in the image.<br><br>A2. An irregular shape, brown-black patches.<br><br>Q3. With previous information, please write a paragraph to explain in detail the cause of this skin disease.<br><br>A3. Melanoma represents a malignant tumor that arises from melanocytes, and, due to its metastatic potential, leads to >75% of skin cancer deaths. The incidence rates of melanoma have increased over the past four decades. In *situ* and early invasive cutaneous melanoma can be subtle in appearance but dermoscopy has led to an improvement in diagnostic accuracy. Early-stage melanomas are often curable by surgical excision. For metastatic melanoma, immunotherapies (e.g. ipilimumab) and<br>targeted therapies (e.g. vemurafenib) can be tried.<br><br>Q4. How should I treat it? What medicine should I use?<br><br>A4. First, a dermoscopy is required. Second, an extended excision of the skin lesion is often necessary. Further treatment is also based on the pathological findings after surgery and other ancillary findings.

dermatologist should be consulted.<br>In some cases, the dermatologist may recommend removing the mole surgically, using local anesthesia. In other cases, a topical medication or freezing the mole may be recommended. In some cases, a biopsy may be performed to determine if the mole is cancerous.<br>It is important to remember that prevention is the best way to avoid this skin disease. This includes wearing protective clothing, using sunscreen with a high SPF, and avoiding tanning beds. Regular skin exams can also help detect any changes in the skin early on.	

Evaluation of Physician	
Evaluation Item	Score (1-5, the higher the better)
SkinGPT-4's diagnosis is correct or relevant.	5
SkinGPT-4's description is informative.	4
SkinGPT-4's suggestions are useful.	4
SkinGPT-4 can help doctors with diagnosis.	5
SkinGPT-4 can help patients to understand their disease better.	5
If SkinGPT-4 can be deployed locally, it protects patients' privacy.	5
Willingness to use SkinGPT-4	5

Case Melanoma 5

Image ID: Melanoma5	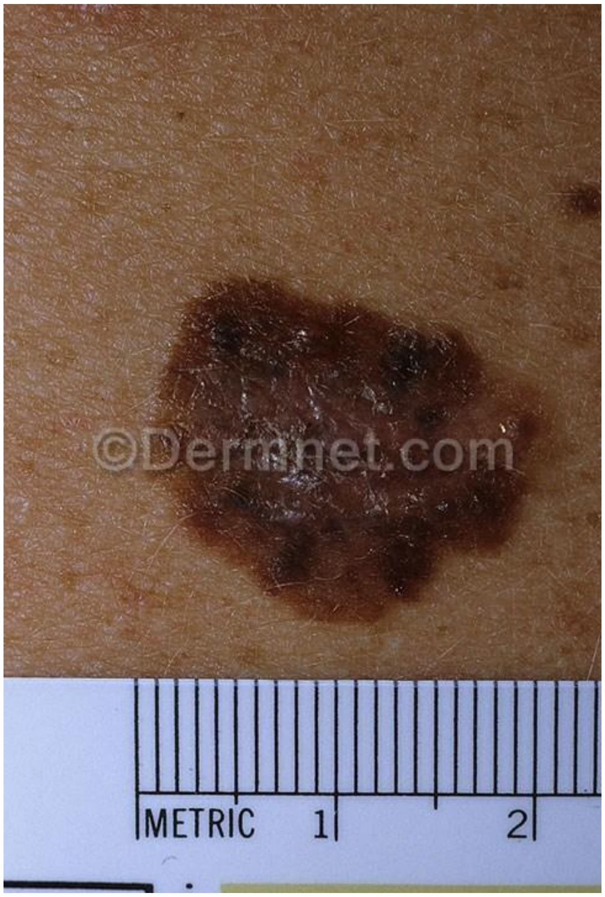
SkinGPT-4	Dermatology Physician
Q1. Could you describe the skin disease in this image for me?<br><br>A1. melanoma skin cancer<br><br>Q2. You should write a paragraph to list more features you observed in the image.<br><br>A2. The image shows a brownish black mole on the skin, which is a benign nevus.<br><br>Q3. With previous information, please write a paragraph to explain in detail the cause of this skin disease.<br><br>A3. The cause of this skin disease is likely due to exposure to ultraviolet radiation from the sun or tanning beds, which can cause mutations in the DNA of skin cells and lead to the formation of	Q1. Could you describe the skin disease in this image for me?<br><br>A1. Melanoma; Melanocytic nevus<br><br>Q2. You should write a paragraph to list more features you observed in the image.<br><br>A2. A brown patches of varying colour with irregular black-thicken crumb near its edge.<br><br>Q3. With previous information, please write a paragraph to explain in detail the cause of this skin disease.<br><br>A3. Melanoma represents a malignant tumor that arises from melanocytes, and, due to its metastatic potential, leads to >75% of skin cancer deaths. The incidence rates of melanoma have

moles or nevi. Over time, some of these moles may develop into malignant melanoma, a type of skin cancer. It is important to monitor any changes in the appearance of moles and to seek medical attention if any changes are observed.<br><br>Q4. How should I treat it? What medicine should I use?<br><br>A4. The treatment for this skin disease will depend on the type and severity of the condition. For benign nevi, no treatment may be necessary, as they are generally harmless. However, if the nevus is causing symptoms or is located in a sensitive area, it may be removed through surgery or other methods. For malignant melanoma, treatment may involve surgery to remove the tumor, as well as other therapies such as chemotherapy, radiation therapy, or immunotherapy. It is important to consult a dermatologist or other medical professional for a proper diagnosis and appropriate treatment plan.	increased over the past four decades. In *situ* and early invasive cutaneous melanoma can be subtle in appearance but dermoscopy has led to an improvement in diagnostic accuracy. Early-stage melanomas are often curable by surgical excision. For metastatic melanoma, immunotherapies (e.g. ipilimumab) and<br>targeted therapies (e.g. vemurafenib) can be tried.<br><br>Q4. How should I treat it? What medicine should I use?<br><br>A4. First, a dermoscopy is required. Second, an extended excision of the skin lesion is often necessary. Further treatment is also based on the pathological findings after surgery and other ancillary findings.
Evaluation of Physician	
Evaluation Item	Score (1-5, the higher the better)
SkinGPT-4's diagnosis is correct or relevant.	5
SkinGPT-4's description is informative.	2
SkinGPT-4's suggestions are useful.	5
SkinGPT-4 can help doctors with diagnosis.	5
SkinGPT-4 can help patients to understand their disease better.	5
If SkinGPT-4 can be deployed locally, it protects patients' privacy.	5
Willingness to use SkinGPT-4	5

Case Melanoma 6

Image ID: Melanoma6	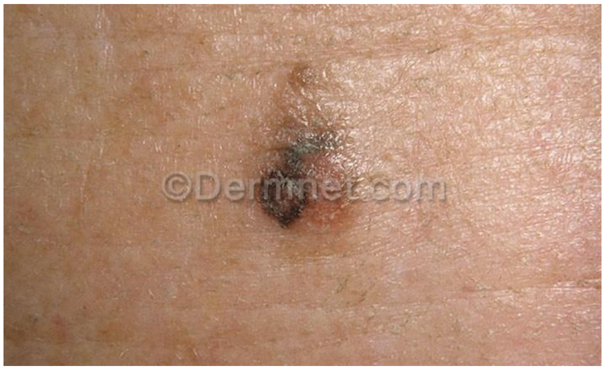
SkinGPT-4	Dermatology Physician
Q1. Could you describe the skin disease in this image for me?<br><br>A1. melanoma skin cancer<br><br>Q2. You should write a paragraph to list more features you observed in the image.<br><br>A2. The lesion in the image is a melanoma, a malignant tumor of melanocytes. It presents as a pigmented or nonpigmented macule or papule, with irregular borders and variable color, and can be preceded by changes in a preexisting mole or by the development of new pigmented lesions. Melanoma can arise from preexisting benign nevi or from de novo melanocytes. It can also occur in mucosal and extramucosal sites, such as the conjunctiva, cornea, and nail bed.<br><br>Q3. With previous information, please write a paragraph to explain in detail the cause of this skin disease.<br><br>A3. Melanoma is caused by mutations in the DNA of melanocytes, which result in the development of malignant cells. The most common cause of melanoma is exposure to ultraviolet (UV) radiation from the sun or tanning beds, which can cause mutations in the DNA of skin cells. Other risk factors for melanoma include a family history of the disease, a large number of moles, and a history of sunburns or tanning. Melanoma can also occur in individuals with a weakened immune system, such as those with HIV or those taking immunosuppressive drugs after an organ	Q1. Could you describe the skin disease in this image for me?<br><br>A1. Melanoma; melanocytic nevus<br><br>Q2. You should write a paragraph to list more features you observed in the image.<br><br>A2. Brown patches of varying colour with black papules on edge.<br><br>Q3. With previous information, please write a paragraph to explain in detail the cause of this skin disease.<br><br>A3. Melanoma represents a malignant tumor that arises from melanocytes, and, due to its metastatic potential, leads to >75% of skin cancer deaths. The incidence rates of melanoma have increased over the past four decades. In *situ* and early invasive cutaneous melanoma can be subtle in appearance but dermoscopy has led to an improvement in diagnostic accuracy. Early-stage melanomas are often curable by surgical excision. For metastatic melanoma, immunotherapies (e.g. ipilimumab) and targeted therapies (e.g. vemurafenib) can be tried.<br><br>Q4. How should I treat it? What medicine should I use?<br><br>A4. First, a dermoscopy is required. Second, an extended excision of the skin lesion is often necessary. Further treatment is also based on the pathological findings after surgery and other ancillary findings.

transplant. In rare cases, melanoma can occur spontaneously, without any known risk factors.<br><br>Q4. How should I treat it? What medicine should I use?<br><br>A4. Treatment for melanoma depends on the stage of the disease, which is determined by the size of the tumor, the presence of metastases, and the thickness of the tumor. In early stages, surgery is the primary treatment, and may involve excision of the tumor or a wide excision with surrounding tissue. In more advanced stages, additional treatments such as chemotherapy, immunotherapy, or targeted therapy may be used. In some cases, a combination of these treatments may be used.<br>It is important to note that the specific treatment plan for melanoma will depend on the individual case and should be discussed with a dermatologist or oncologist. Medications that may be used include:<br>* BRAF inhibitors, such as vemurafenib and dabrafenib, which target a specific protein in melanoma cells and can slow the growth and spread of the cancer<br>* MEK inhibitors, such as trametinib and cobimetinib, which target another protein in melanoma cells and can be used in combination with BRAF inhibitors<br>* Immunotherapy drugs, such as ipilimumab and nivolumab, which work by stimulating the immune system to attack cancer cells.	
Evaluation of Physician	
Evaluation Item	Score (1-5, the higher the better)
SkinGPT-4's diagnosis is correct or relevant.	5
SkinGPT-4's description is informative.	5
SkinGPT-4's suggestions are useful.	5
SkinGPT-4 can help doctors with diagnosis.	5
SkinGPT-4 can help patients to understand their disease better.	5

If SkinGPT-4 can be deployed locally, it protects patients' privacy.	5
Willingness to use SkinGPT-4	5

Case Melanoma 7

Image ID: Melanoma7	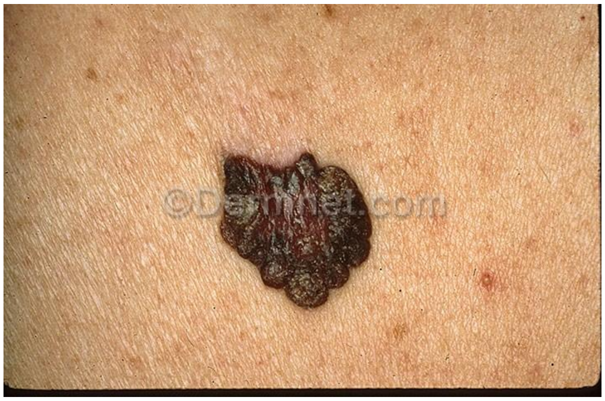
SkinGPT-4	Dermatology Physician
Q1. Could you describe the skin disease in this image for me?<br><br>A1. melanoma skin cancer<br><br>Q2. You should write a paragraph to list more features you observed in the image.<br><br>A2. The lesion in this image is a melanoma, a type of skin cancer that arises from melanocytes, the cells that produce melanin, which gives skin its pigmentation. Melanomas can occur anywhere on the skin but are most common on the skin that is exposed to the sun, such as the face, neck, and arms. They often present as a new or changing mole or a dark spot on the skin. Melanomas can be benign or malignant and can spread to other parts of the body if not treated.<br><br>Q3. With previous information, please write a paragraph to explain in detail the cause of this skin disease.<br><br>A3. Melanoma is caused by a combination of genetic and environmental factors. The primary risk factor for melanoma is exposure to ultraviolet (UV) radiation from the sun or other sources. This can cause mutations in the DNA of melanocytes, leading to the development of melanoma. Other risk factors include a family history of melanoma, a history of sunburns, fair skin, many moles, and a weakened immune system.<br><br>Q4. How should I treat it? What medicine should I	Q1. Could you describe the skin disease in this image for me?<br><br>A1. Melanoma; melanocytic nevus<br><br>Q2. You should write a paragraph to list more features you observed in the image.<br><br>A2. Unevenly colored dark brown patches. Irregular surface thickness .<br><br>Q3. With previous information, please write a paragraph to explain in detail the cause of this skin disease.<br><br>A3. Melanoma represents a malignant tumor that arises from melanocytes, and, due to its metastatic potential, leads to >75% of skin cancer deaths. The incidence rates of melanoma have increased over the past four decades. In *situ* and early invasive cutaneous melanoma can be subtle in appearance but dermoscopy has led to an improvement in diagnostic accuracy. Early-stage melanomas are often curable by surgical excision. For metastatic melanoma, immunotherapies (e.g. ipilimumab) and targeted therapies (e.g. vemurafenib) can be tried.<br><br>Q4. How should I treat it? What medicine should I use?<br><br>A4. First, a dermoscopy is required. Second, an extended excision of the skin lesion is often necessary. Further treatment is also based on the pathological findings after surgery and other

use?<br><br>A4. Treatment for melanoma depends on the stage of the disease. Early stage melanoma can be treated with surgery, while more advanced stages may require a combination of surgery, radiation therapy, and/or chemotherapy. In some cases, immunotherapy drugs may also be used to treat melanoma.	ancillary findings.
Evaluation of Physician	
Evaluation Item	Score (1-5, the higher the better)
SkinGPT-4's diagnosis is correct or relevant.	5
SkinGPT-4's description is informative.	5
SkinGPT-4's suggestions are useful.	5
SkinGPT-4 can help doctors with diagnosis.	5
SkinGPT-4 can help patients to understand their disease better.	5
If SkinGPT-4 can be deployed locally, it protects patients' privacy.	5
Willingness to use SkinGPT-4	5

Case Melanoma 8

Image ID: Melanoma8	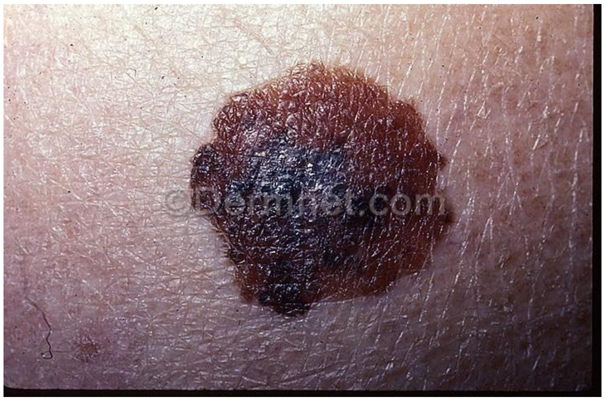
SkinGPT-4	Dermatology Physician
Q1. Could you describe the skin disease in this image for me?<br><br>A1. Melanoma<br><br>Q2. You should write a paragraph to list more features you observed in the image.<br><br>A2. Melanoma is a type of skin cancer that can develop on any part of the skin. It is caused by the abnormal growth of melanocytes, which are cells that produce melanin, the pigment that gives skin its color. Melanoma can be flat or raised and can be brown, black, or multicolored. It can also be found on the nails or in the eye. Melanoma can be treated by surgery, radiation therapy, or immunotherapy. It is important to check for melanoma regularly, especially if you have a family history of the disease. Wearing sunscreen, avoiding tanning beds, and wearing protective clothing can also help reduce your risk of developing melanoma. Melanoma is a serious disease and can be life threatening if not treated in time.<br>Q3. With previous information, please write a paragraph to explain in detail the cause of this skin disease.<br><br>A3. Melanoma is caused by the abnormal growth of melanocytes, which are cells that produce melanin, the pigment that gives skin its color. The cause of melanoma is not yet fully understood, but it is thought to be caused by a combination of genetic and environmental factors. People with a family history of melanoma, fair skin, blue or green	Q1. Could you describe the skin disease in this image for me?<br><br>A1. Melanoma; melanocytic nevus<br><br>Q2. You should write a paragraph to list more features you observed in the image.<br><br>A2. Unevenly colored dark brown patches. Irregular surface thickness .<br><br>Q3. With previous information, please write a paragraph to explain in detail the cause of this skin disease.<br><br>A3. Melanoma represents a malignant tumor that arises from melanocytes, and, due to its metastatic potential, leads to >75% of skin cancer deaths. The incidence rates of melanoma have increased over the past four decades. In *situ* and early invasive cutaneous melanoma can be subtle in appearance but dermoscopy has led to an improvement in diagnostic accuracy. Early-stage melanomas are often curable by surgical excision. For metastatic melanoma, immunotherapies (e.g. ipilimumab) and<br>targeted therapies (e.g. vemurafenib) can be tried.<br><br>Q4. How should I treat it? What medicine should I use?<br><br>A4. First, a dermoscopy is required. Second, an extended excision of the skin lesion is often necessary. Further treatment is also based on the pathological findings after surgery and other

eyes, and a tendency to burn rather than tan are at higher risk of developing melanoma. Exposure to ultraviolet (UV) light from the sun or tanning beds, as well as certain chemicals, can also increase the risk of melanoma. It is important to protect yourself from the sun and avoid tanning beds to reduce your risk of developing melanoma.<br><br>Q4. How should I treat it? What medicine should I use?<br><br>A4. Melanoma can be treated by surgery, radiation therapy, or immunotherapy. The treatment options depend on the stage of the cancer, which is determined by how far the cancer has spread. Surgery is typically the first line of treatment for melanoma and involves removing the tumor and some surrounding tissue. In some cases, the lymph nodes may also be removed. Radiation therapy uses high-energy X-rays or other types of radiation to kill cancer cells. Immunotherapy, also known as biologic therapy, works by stimulating the body's immune system to fight the cancer. These are some of the treatment options for melanoma. It is important to discuss the best treatment plan with a dermatologist or other healthcare provider.	ancillary findings.
Evaluation of Physician	
Evaluation Item	Score (1-5, the higher the better)
SkinGPT-4's diagnosis is correct or relevant.	5
SkinGPT-4's description is informative.	5
SkinGPT-4's suggestions are useful.	5
SkinGPT-4 can help doctors with diagnosis.	5
SkinGPT-4 can help patients to understand their disease better.	5
If SkinGPT-4 can be deployed locally, it protects patients' privacy.	5
Willingness to use SkinGPT-4	5

Case Melanoma 9

Image ID: Melanoma9	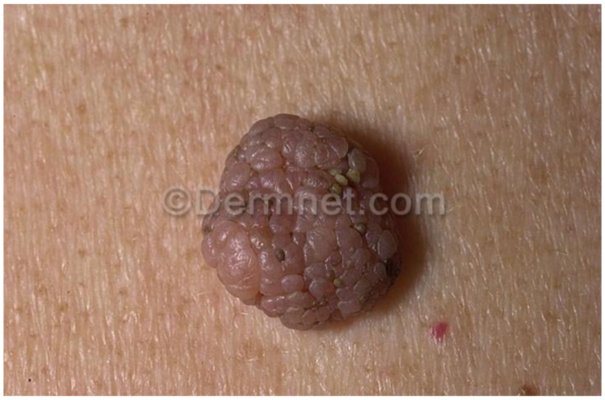
**SkinGPT-4**	**Dermatology Physician**
Q1. Could you describe the skin disease in this image for me?<br><br>A1. melanoma skin cancer<br><br>Q2. You should write a paragraph to list more features you observed in the image.<br><br>A2. The image shows a small brown mole on the skin, which is a benign skin growth that is usually harmless. It has a smooth surface and is slightly raised above the surrounding skin. It has a round shape and is about the size of a pencil eraser. It has a dark brown color and a slightly irregular border. It appears to be growing on the skin and may be slightly raised or flat. It is not painful and does not itch or bleed.<br><br>Q3. With previous information, please write a paragraph to explain in detail the cause of this skin disease.<br><br>A3. The cause of this skin disease is likely to be a benign mole, which is a small growth on the skin that is composed of pigment-producing cells. These moles can develop as a result of sun exposure, genetics, or other factors. They are usually harmless and do not cause any symptoms. However, in some cases, moles can become cancerous and develop into melanoma, which is a more serious form of skin cancer. It is important to monitor any changes in the appearance of moles	Q1. Could you describe the skin disease in this image for me?<br><br>A1. Melanocytic nevus<br><br>Q2. You should write a paragraph to list more features you observed in the image.<br><br>A2. Pale brown papillomatous papule/nodule.<br><br>Q3. With previous information, please write a paragraph to explain in detail the cause of this skin disease.<br><br>A3. Melanoma represents a malignant tumor that arises from melanocytes, and, due to its metastatic potential, leads to >75% of skin cancer deaths. The incidence rates of melanoma have increased over the past four decades. In *situ* and early invasive cutaneous melanoma can be subtle in appearance but dermoscopy has led to an improvement in diagnostic accuracy. Early-stage melanomas are often curable by surgical excision. For metastatic melanoma, immunotherapies (e.g. ipilimumab) and targeted therapies (e.g. vemurafenib) can be tried.<br><br>Q4. How should I treat it? What medicine should I use?<br><br>A4. First, a dermoscopy is required. Second, an extended excision of the skin lesion is often

and to seek medical attention if any unusual symptoms develop, such as bleeding, itching, or pain.<br><br>Q4. How should I treat it? What medicine should I use?<br><br>A4. It is important to note that the image provided is not sufficient to determine the appropriate treatment for this skin disease. A diagnosis can only be made by a dermatologist or other medical professional who has examined the skin in person.<br>If the mole is found to be benign, no treatment may be necessary. However, if it is cancerous, treatment may involve surgery to remove the mole and surrounding tissue, as well as possible radiation or chemotherapy. The specific treatment plan will depend on the severity and stage of the cancer.<br>It is important to consult a medical professional for an accurate diagnosis and appropriate treatment. Do not attempt to treat any skin growths or moles on your own, as this can be dangerous and may lead to further complications.	necessary. Further treatment is also based on the pathological findings after surgery and other ancillary findings.
Evaluation of Physician	
Evaluation Item	Score (1-5, the higher the better)
SkinGPT-4's diagnosis is correct or relevant.	3
SkinGPT-4's description is informative.	5
SkinGPT-4's suggestions are useful.	1
SkinGPT-4 can help doctors with diagnosis.	1
SkinGPT-4 can help patients to understand their disease better.	1
If SkinGPT-4 can be deployed locally, it protects patients' privacy.	5
Willingness to use SkinGPT-4	1

Case Melanoma 10

Image ID: Melanoma10	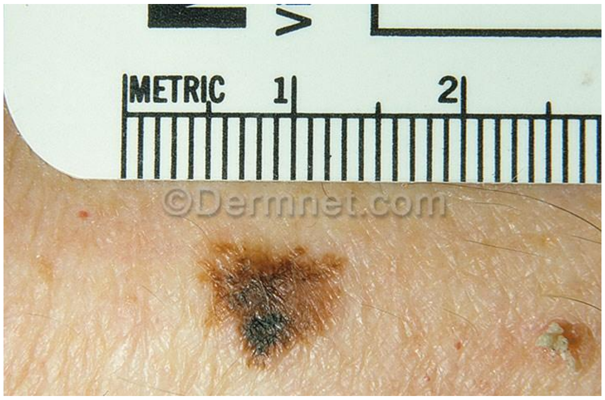
**SkinGPT-4**	**Dermatology Physician**
Q1. Could you describe the skin disease in this image for me?<br><br>A1. melanoma skin cancer<br><br>Q2. You should write a paragraph to list more features you observed in the image.<br><br>A2. The image shows a lesion on the skin, which is a common type of skin cancer known as melanoma. It has a brownish black color and is irregular in shape with a scaly surface. It has a well-defined border and is raised above the surrounding skin. It is located on the back of the right hand and appears to be growing in size.<br><br>Q3. With previous information, please write a paragraph to explain in detail the cause of this skin disease.<br><br>A3. Melanoma is caused by mutations in the genes that control the development and growth of melanocytes. Melanocytes are cells that produce the pigment melanin, which is responsible for the color of skin, hair, and eyes. The mutations lead to the growth of abnormal melanocytes, which can form malignant tumors. The cause of these mutations is largely unknown, but they are thought to be caused by a combination of genetic and environmental factors. Exposure to ultraviolet radiation from sunlight is a known risk factor for melanoma, and people with fair skin, light hair, and light eyes are more likely to develop the disease. Other risk factors include a family history of melanoma, multiple moles, and a weakened	Q1. Could you describe the skin disease in this image for me?<br><br>A1. Melanoma; melanocytic nevus<br><br>Q2. You should write a paragraph to list more features you observed in the image.<br><br>A2. Triangular brown spots. Black in the center. Irregularly bordered, unevenly colored dark brown macules<br><br>Q3. With previous information, please write a paragraph to explain in detail the cause of this skin disease.<br><br>A3. Melanoma represents a malignant tumor that arises from melanocytes, and, due to its metastatic potential, leads to >75% of skin cancer deaths. The incidence rates of melanoma have increased over the past four decades. In *situ* and early invasive cutaneous melanoma can be subtle in appearance but dermoscopy has led to an improvement in diagnostic accuracy. Early-stage melanomas are often curable by surgical excision. For metastatic melanoma, immunotherapies (e.g. ipilimumab) and targeted therapies (e.g. vemurafenib) can be tried.<br><br>Q4. How should I treat it? What medicine should I use?<br><br>A4. First, a dermoscopy is required. Second, an extended excision of the skin lesion is often necessary. Further treatment is also based on the

immune system.<br><br>Q4. How should I treat it? What medicine should I use?<br><br>A4. Melanoma is typically treated with surgery to remove the tumor. If the melanoma is in an early stage, it can be surgically removed with a wide margin of healthy tissue around the tumor to reduce the risk of recurrence. If the melanoma has spread to other parts of the body, it may be treated with immunotherapy, targeted therapy, or a combination of these approaches. In some cases, a clinical trial may be recommended to test new treatments or combinations of treatments. Treatment for melanoma may also involve supporting care, such as pain management, wound care, and nutritional support, to help patients cope with the physical and emotional aspects of treatment. The treatment plan will depend on the stage and characteristics of the melanoma, as well as the patient's overall health and preferences.	pathological findings after surgery and other ancillary findings.

Evaluation of Physician	
Evaluation Item	Score (1-5, the higher the better)
SkinGPT-4's diagnosis is correct or relevant.	5
SkinGPT-4's description is informative.	5
SkinGPT-4's suggestions are useful.	5
SkinGPT-4 can help doctors with diagnosis.	5
SkinGPT-4 can help patients to understand their disease better.	5
If SkinGPT-4 can be deployed locally, it protects patients' privacy.	5
Willingness to use SkinGPT-4	5

Case Fungal infection-Tinea 1

Image ID: Tinea1	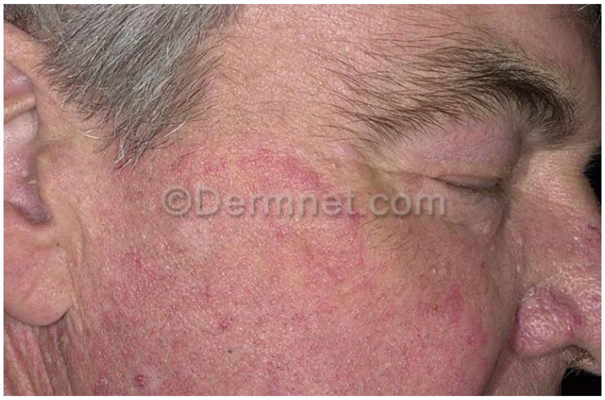
SkinGPT-4	Dermatology Physician
Q1. Could you describe the skin disease in this image for me?<br><br>A1. This could be a skin disease image of tinea face.<br><br>Q2. You should write a paragraph to list more features you observed in the image.<br><br>A2. This image appears to depict a person's face with a rash. The rash is scaly and appears to have clear boundaries. There is erythema (redness) and papules (small bumps) present on the rash. The rash is on both sides of the face and appears to extend to the eyebrows and eyelids. There is also a fissure on the lower lip. The lesions appear to be active and there is some crusting present. This could be a possible diagnosis of seborrheic dermatitis, tinea faciei or psoriasis. A biopsy would be needed to confirm the diagnosis.<br><br>Q3. With previous information, please write a paragraph to explain in detail the cause of this skin disease.<br><br>A3. This image appears to depict a rash on the face of an individual. The rash is scaly and has clear boundaries, erythema (redness) and papules (small bumps) are present on the rash. The rash is on both sides of the face and extends to the eyebrows and eyelids. This suggests that the rash may be caused by a fungal infection such as tinea faciei or seborrheic dermatitis. Both of these conditions are caused by a fungus, and can present with similar symptoms. Tinea faciei is	Q1. Could you describe the skin disease in this image for me?<br><br>A1. Tinea Faciei; Erythema(of undetermined origin)<br><br>Q2. You should write a paragraph to list more features you observed in the image.<br><br>A2. Circumscribed facial erythema with red-edged papules, but fading in the central area.<br><br>Q3. With previous information, please write a paragraph to explain in detail the cause of this skin disease.<br><br>A3. Dermatophytoses are fungal infections caused by three genera of fungi that have the unique ability to invade and multiply within keratinized tissue (hair, skin and nails).<br><br>Q4. How should I treat it? What medicine should I use?<br><br>A4. (1) fungal examination to confirm the diagnosis;<br>(2) topical antifungal drug (used for at least one month).

caused by the fungus Trichophyton, and commonly affects the face, especially around the nose, mouth, and eyelids. Seborrheic dermatitis is caused by Malassezia fungus and can cause scaly and flaky skin. The presence of a fissure on the lower lip suggests that the condition may also be psoriasis, which is an autoimmune condition that causes scaly, red skin. To confirm the diagnosis, a biopsy should be performed. It's important to note that without proper diagnosis and treatment, these conditions can lead to significant discomfort and discomfort for the patient, as well as possible complications.<br><br>Q4. How should I treat it? What medicine should I use?<br><br>A4. The treatment for this skin disease will depend on the underlying cause. If the condition is caused by a fungal infection such as tinea faciei or seborrheic dermatitis, antifungal medication will be prescribed. If the condition is caused by psoriasis, topical corticosteroids or vitamin D analogs may be prescribed.<br>For tinea faciei, antifungal creams or ointments containing ciclopirox, naftifine, or selenium sulfide are typically used. These medications work by killing the fungus that causes the infection. The cream or ointment is applied directly to the affected areas of the skin twice daily for two to four weeks.<br>For seborrheic dermatitis, medications such as ketoconazole, selenium sulfide, or zinc pyrithione can be prescribed. These medications work by reducing the amount of fungus on the skin, which in turn reduces inflammation and scaling. The cream or shampoo is applied to the affected areas of the skin once or twice daily for several weeks.<br>For psoriasis, topical corticosteroids or vitamin D analogs are often prescribed. These medications help to reduce inflammation, redness and scaling.	
Evaluation of Physician	
Evaluation Item	Score (1-5, the higher the better)
SkinGPT-4's diagnosis is correct or relevant.	5
SkinGPT-4's description is informative.	5

SkinGPT-4's suggestions are useful.	4
SkinGPT-4 can help doctors with diagnosis.	5
SkinGPT-4 can help patients to understand their disease better.	5
If SkinGPT-4 can be deployed locally, it protects patients' privacy.	5
Willingness to use SkinGPT-4	5

Case Fungal infection-Tinea 2

Image ID: Tinea2	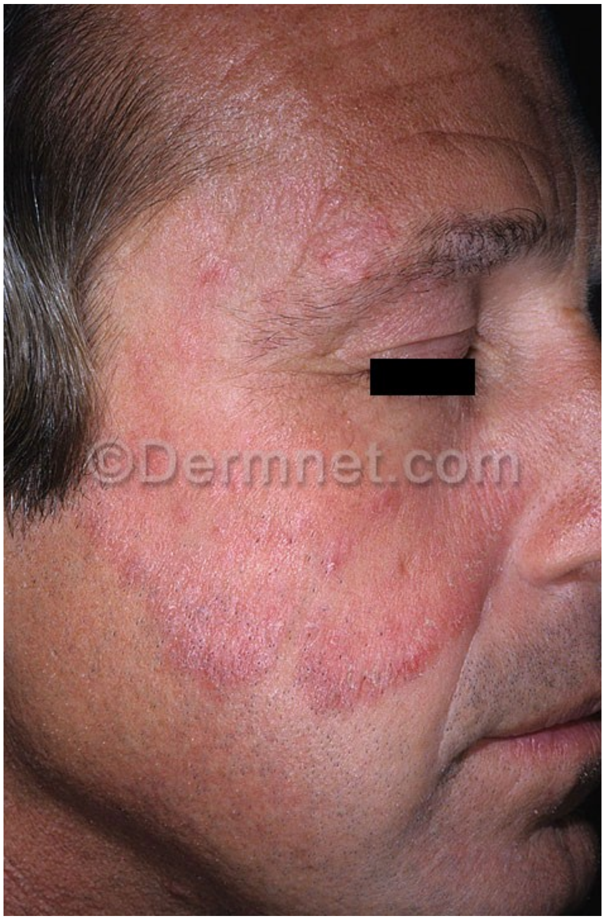
**SkinGPT-4**	**Dermatology Physician**
Q1. Could you describe the skin disease in this image for me?<br><br>A1. This could be a skin disease image of tinea face.<br><br>Q2. You should write a paragraph to list more features you observed in the image.<br><br>A2. In the image, there are several features observed. Firstly, there is a rash present on the face, which is erythematous and scaly. Secondly, there are visible follicles, which appear as small, black dots on the rash. Thirdly, the edges of the rash are well defined and have a clear boundary. Fourthly, there is a central clearing present on the nose, which is known as the “clean area” sign. Lastly, the rash appears to be confined to the face and does not extend to other areas of the body.	Q1. Could you describe the skin disease in this image for me?<br><br>A1. Tinea Facieil; Erythema(of undetermined origin)<br><br>Q2. You should write a paragraph to list more features you observed in the image.<br><br>A2. Circumscribed facial erythema with red-edged papules, but fading in the central area.<br><br>Q3. With previous information, please write a paragraph to explain in detail the cause of this skin disease.<br><br>A3. Dermatophytoses are fungal infections caused by three genera of fungi that have the unique ability to invade and multiply within keratinized tissue

Q3. With previous information, please write a paragraph to explain in detail the cause of this skin disease.<br><br>A3. This skin disease is caused by a fungal infection known as Tinea Face. The fungus causes an allergic response in the skin, leading to inflammation and the characteristic rash seen in the image. The follicles visible in the image are probably dilated due to the inflammation caused by the fungus. The fungus can be spread through direct contact with an infected person or animal, or through contact with an object that has been contaminated with the fungus. The "clean area" sign on the nose is likely due to the fact that the fungus infects the hair follicles, but not the surrounding skin, leading to a clearing in the center of the rash. The face is a common site for this type of fungal infection because the skin is moist and warm, providing an ideal environment for fungal growth. The infection can be treated with topical or or oral antifungal medications, depending on the severity of the disease. It is important to seek medical attention if you suspect you have a fungal infection to receive the appropriate treatment and to prevent the spread of the infection to others.<br><br>Q4. How should I treat it? What medicine should I use?<br><br>A4. Treatment for tinea face (also known as facial tinea) typically involves topical antifungal medications, such as creams or ointments containing azoles (e.g. clotrimazole, ketoconazole) or allylamines (e.g. naftifine). Oral antifungal medications, such as fluconazole, itraconazole, or terbinafine, may also be prescribed for more severe cases or for individuals who have a weakened immune system. It is important to follow the instructions for use and complete the full course of treatment, even if the symptoms improve before the full course is finished. It is also important to avoid sharing personal items, such as towels, razors, or pillows, to avoid spreading the infection to others. Additionally, keeping the face clean and dry, and avoiding use of harsh soaps or fragrances, can help prevent the spread of the infection and improve the effectiveness of treatment.	(hair, skin and nails).<br><br>Q4. How should I treat it? What medicine should I use?<br><br>A4. (1) fungal examination to confirm the diagnosis;<br>(2) topical antifungal drug (used for at least one month).

Evaluation of Physician	
Evaluation Item	Score (1-5, the higher the better)
SkinGPT-4's diagnosis is correct or relevant.	5
SkinGPT-4's description is informative.	5
SkinGPT-4's suggestions are useful.	4
SkinGPT-4 can help doctors with diagnosis.	5
SkinGPT-4 can help patients to understand their disease better.	4
If SkinGPT-4 can be deployed locally, it protects patients' privacy.	5
Willingness to use SkinGPT-4	5

Case Fungal infection-Tinea 3

<table>
<tr><td>Image ID: Tinea3</td><td>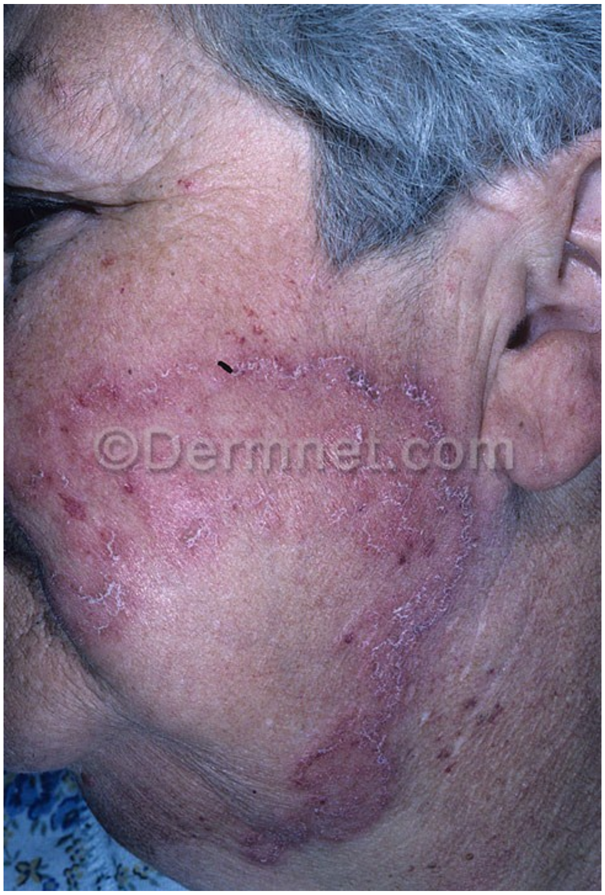
</td></tr>
<tr><th>SkinGPT-4</th><th>Dermatology Physician</th></tr>
<tr><td>Q1. Could you describe the skin disease in this image for me?

A1. This could be a skin disease image of tinea face.

Q2. You should write a paragraph to list more features you observed in the image.

A2. This image appears to depict a face with tinea face. The rash is characterized by erythema, papules, and vesicles on the cheeks, nose, and eyelids. The face appears scaly and there is hypopigmentation. The lesions extend to the forehead and temples and there is some crusting. The patient has a negative Tinel sign and a positive Kogojakow test. The eruption is more pronounced on the left side of the face.</td><td>Q1. Could you describe the skin disease in this image for me?

A1. Tinea Faciei; Erythema(of undetermined origin)

Q2. You should write a paragraph to list more features you observed in the image.

A2. Dermatophytoses are fungal infections caused by three genera of fungi that have the unique ability to invade and multiply within keratinized tissue (hair, skin and nails).

Q3. With previous information, please write a paragraph to explain in detail the cause of this skin disease.

A3. Dermatophytoses are fungal infections caused by three genera of fungi that have the unique ability</td></tr>
</table>

Q3. With previous information, please write a paragraph to explain in detail the cause of this skin disease.<br><br>A3. The cause of this skin disease is a fungal infection caused by the dermatophyte fungus. Dermatophytes are a type of fungus that infect the skin and cause superficial fungal infections, such as tinea faciei. These fungi are highly specialized and can only infect keratin-rich skin, hair, and nails. The infection occurs through direct contact with an infected person or animal or by contact with an object that has been contaminated with the fungus, such as a towel or comb. Once the fungus infects the skin, it feeds on the keratin and produces metabolites that cause inflammation and irritation, leading to the characteristic symptoms of tinea face. The fungus can also spread to other parts of the body, leading to tinea corporis (ringworm) or tinea pedis (athlete's foot). The symptoms of tinea face can be treated with antifungal medications, such as topical corticosteroids, antifungal creams, or oral antifungals. It is important to identify and treat the underlying fungal infection to prevent recurrence and spread of the disease.<br><br>Q4. How should I treat it? What medicine should I use?<br><br>A4. The treatment of tinea face depends on the severity and extent of the infection. Mild cases can be treated with topical antifungal creams or ointments, while more severe cases may require oral antifungal medications. The most common topical antifungal medications used to treat tinea face are clotrimazole, miconazole, and ketoconazole. These medications work by disrupting the fungal cell membrane and inhibiting the growth of the fungus. They are applied to the affected area twice daily for two weeks.<br>Oral antifungal medications, such as fluconazole or itraconazole, are prescribed for severe or extensive infections that do not respond to topical treatment. These medications work by inhibiting the enzymes involved in the fungal cell cycle and preventing the growth of the fungus. They are taken orally for several weeks.<br>It is important to follow the instructions provided by your healthcare provider and complete the full course of medication to ensure that the fungal infection is fully treated and to prevent the spread	to invade and multiply within keratinized tissue (hair, skin and nails).<br><br>Q4. How should I treat it? What medicine should I use?<br><br>A4. (1) fungal examination to confirm the diagnosis;<br>(2) topical antifungal drug (used for at least one month).

of the infection to other parts of the body. Proper hygiene and avoiding contact with infected individuals or animals can also help prevent the development of tinea face and other fungal infections.	
Evaluation of Physician	
Evaluation Item	Score (1-5, the higher the better)
SkinGPT-4's diagnosis is correct or relevant.	5
SkinGPT-4's description is informative.	4
SkinGPT-4's suggestions are useful.	5
SkinGPT-4 can help doctors with diagnosis.	5
SkinGPT-4 can help patients to understand their disease better.	5
If SkinGPT-4 can be deployed locally, it protects patients' privacy.	5
Willingness to use SkinGPT-4	5

Case Fungal infection-Tinea 4

Image ID: Tinea4	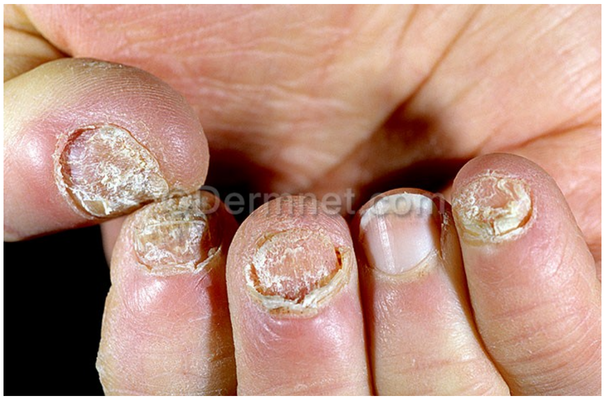
SkinGPT-4	Dermatology Physician
Q1. Could you describe the skin disease in this image for me?<br><br>A1. This could be a skin disease image of tinea.<br><br>Q2. You should write a paragraph to list more features you observed in the image.<br><br>A2. This could be a skin disease image of tinea. The lesions are caused by an overgrowth of the skin due to infection with the fungus Trichophyton. The infection can be treated with antifungal creams, sprays or medicated powders.<br><br>Q3. With previous information, please write a paragraph to explain in detail the cause of this skin disease.<br><br>A3. Tinea, also known as ringworm, is a fungal infection. It is caused by a group of fungi called dermatophytes, which are commonly found on the skin. These fungi are often found on humans and animals and can thrive in various environments. Exposure to these fungi can lead to infection. Tinea is highly contagious and can be easily spread through direct contact with an infected person or animal..<br><br>Q4. How should I treat it? What medicine should I use?<br><br>A4. Treatment of tinea depends on the severity of the infection. Mild tinea infections can be treated with over-the-counter antifungal creams. However, severe cases may require prescription	Q1. Could you describe the skin disease in this image for me?<br><br>A1. Onycholysis; Nail Dystrophy<br><br>Q2. You should write a paragraph to list more features you observed in the image.<br><br>A2. The nail plate becomes cloudy, yellow. The nail plate becomes thin and the distal segment of the nail is missing.<br><br>Q3. With previous information, please write a paragraph to explain in detail the cause of this skin disease.<br><br>A3. The etiology is unknown and may be related to nail damage caused by multiple factors.<br>It may be related to nail damage caused by various factors. For example, lichen planus, psoriasis, baldness, and fungal infections can cause this disease.<br><br>Q4. How should I treat it? What medicine should I use?<br><br>A4. A detailed medical history, medication history and examination for nail fungus is essential. Careful examination of the body is needed to exclude other skin conditions. Fungal infection, if present, may be treated with oral antifungals.

medications from a healthcare provider. It is important to keep the affected area clean and dry, and to avoid close contact with other people to prevent the infection from spreading.	
Evaluation of Physician	
Evaluation Item	Score (1-5, the higher the better)
SkinGPT-4's diagnosis is correct or relevant.	5
SkinGPT-4's description is informative.	5
SkinGPT-4's suggestions are useful.	5
SkinGPT-4 can help doctors with diagnosis.	5
SkinGPT-4 can help patients to understand their disease better.	5
If SkinGPT-4 can be deployed locally, it protects patients' privacy.	5
Willingness to use SkinGPT-4	5

Case Fungal infection-Tinea 5

Image ID: Tinea5	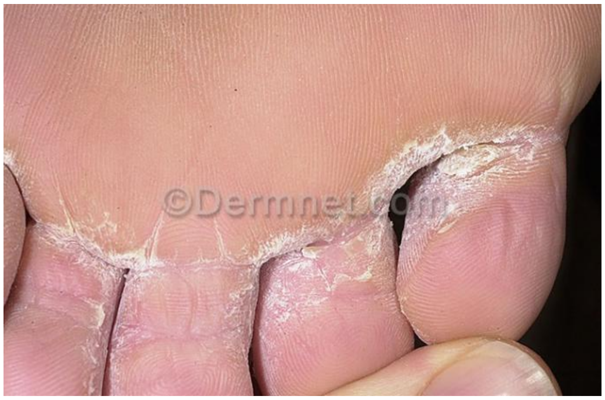
**SkinGPT-4**	**Dermatology Physician**
Q1. Could you describe the skin disease in this image for me?<br><br>A1. This could be a skin disease image of tinea foot webs.<br><br>Q2. You should write a paragraph to list more features you observed in the image.<br><br>A2. In this image, the foot has tinea foot webs, which are fine, white, velvety threads that follow the lines of pressure on the sole and sides of the foot. The tinea infection has caused the skin to become thickened and hyperkeratotic, with a whitish-gray color. The tinea has also caused the nails to become discolored and thickened, with a distorted shape. The skin around the nails appears normal, but there is no pus or odor present. This image could be a skin disease image of tinea foot.<br><br>Q3. With previous information, please write a paragraph to explain in detail the cause of this skin disease.<br><br>A3. Tinea foot is caused by a dermatophyte fungal infection, which affects the skin on the feet. The fungus attacks the keratin protein in the skin, causing it to become thickened and hyperkeratotic. The fungus thrives in a humid and moist environment, and the skin on the feet is a common site for infection because it is often in contact with moisture. The fungus can be transmitted through direct contact with an infected person or animal, or by sharing personal items	Q1. Could you describe the skin disease in this image for me?<br><br>A1. Tinea Pedis<br><br>Q2. You should write a paragraph to list more features you observed in the image.<br><br>A2. Between the toes, small flakes of debridement are visible. They may be arcuate or ring-shaped and attached to the edge of the lesion.<br><br>Q3. With previous information, please write a paragraph to explain in detail the cause of this skin disease.<br><br>A3. Dermatophytoses are fungal infections caused by three genera of fungi that have the unique ability to invade and multiply within keratinized tissue (hair, skin and nails).<br><br>Q4. How should I treat it? What medicine should I use?<br><br>A4. (1) fungal examination to confirm the diagnosis;<br>(2) topical antifungal drug (used for at least one month).

<table>
<tr><td>such as towels or socks. The fungus can also be found in public places such as gyms, swimming pools, and locker rooms. Once the fungus infects the skin, it produces tiny threads of white, velvety material called tinea fibrillae, also known as ring worm. These threads can be seen on the skin and can be mistaken for a rash. The infection can spread to other parts of the body, including the nails, causing them to become discolored, thickened and distorted. The infection can be treated with antifungal creams, powders or tablets, and the nails may need to be removed if the infection is severe. Prevention includes keeping the feet dry, wearing breathable shoes, and avoiding sharing personal items.

Q4. How should I treat it? What medicine should I use?

A4. Tinea foot is treated with antifungal medications, such as topical creams, ointments, or oral medications. The most common topical antifungal agents used are clotrimazole, miconazole, and econazole. These medications work by inhibiting the growth of the fungus, and preventing it from producing the enzymes that break down the skin’s natural oils.
Oral antifungal medications, such as fluconazole, itraconazole, and terbinafine, are also used to treat tinea foot. These medications are prescribed for more severe or extensive infections, or for those that do not respond to topical therapy.
It is important to follow the instructions provided by your healthcare provider, and to complete the full course of medication, even if the symptoms have improved.
It’s also important to keep the feet clean and dry, and to avoid sharing personal items such as towels or socks. Wearing breathable shoes and sandals can help to keep the feet dry and prevent the fungus from spreading.
Please consult a healthcare professional for a proper diagnosis, and for guidance on the most appropriate treatment for your specific case.</td><td></td></tr>
<tr><td colspan="2">Evaluation of Physician</td></tr>
<tr><td>Evaluation Item</td><td>Score (1-5, the higher the better)</td></tr>
<tr><td>SkinGPT-4’s diagnosis is correct or relevant.</td><td>5</td></tr>
</table>

SkinGPT-4's description is informative.	5
SkinGPT-4's suggestions are useful.	5
SkinGPT-4 can help doctors with diagnosis.	5
SkinGPT-4 can help patients to understand their disease better.	5
If SkinGPT-4 can be deployed locally, it protects patients' privacy.	5
Willingness to use SkinGPT-4	5

Case Fungal infection-Nail Fungal infection 1

<table>
<tr><td>Image ID: Nail Fungal infection1</td><td>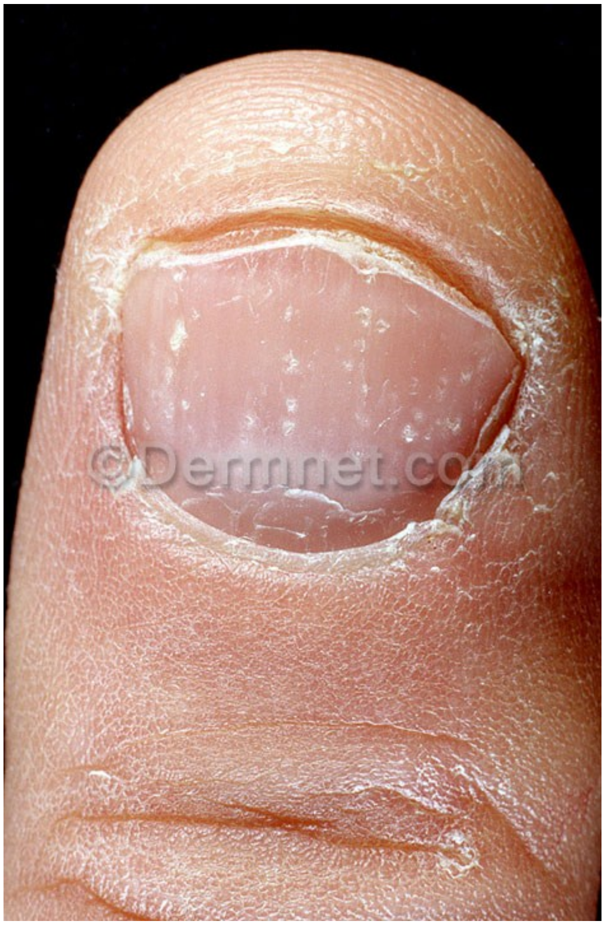
</td></tr>
<tr><th>SkinGPT-4</th><th>Dermatology Physician</th></tr>
<tr><td>Q1. Could you describe the skin disease in this image for me?

A1. This could be a skin disease image of psoriasis.

Q2. You should write a paragraph to list more features you observed in the image.

A2. The image of psoriasis on the nails is a skin disease image that shows changes in the appearance of the nails. The nails appear abnormal with changes in shape, size, color, and texture. The nails may become pitted, discolored, and thickened, with an irregular surface. In some cases, the nails may also separate from the nail bed, a condition known as onycholysis. This</td><td>Q1. Could you describe the skin disease in this image for me?

A1. Thimble-shaped nail

Q2. You should write a paragraph to list more features you observed in the image.

A2. Needle-sized punctate depressions on the surface of the deck.

Q3. With previous information, please write a paragraph to explain in detail the cause of this skin disease.

A3. The nail dimples are the result of defects in the nail formation of the nail matrix. They can occasionally occur in healthy people. They can also</td></tr>
</table>

image demonstrates the characteristic features of psoriasis affecting the nails.<br><br>Q3. With previous information, please write a paragraph to explain in detail the cause of this skin disease.<br><br>A3. Psoriasis is an autoimmune skin condition that causes inflammation and rapid growth of skin cells. It can affect many parts of the body, including the nails. The exact cause of psoriasis is not known, but it is thought to be related to an overactive immune system and genetic factors. The immune system mistakenly attacks healthy cells in the skin, causing inflammation and the rapid growth of skin cells. This results in the formation of thick, scaly plaques on the skin's surface. When psoriasis affects the nails, it causes changes in their appearance, such as pitting, discoloration, and thickening. The exact reason why psoriasis affects the nails is not well understood, but it may be related to an underlying systemic inflammation or genetic factors. This image shows the changes in the appearance of the nails caused by psoriasis.<br><br>Q4. How should I treat it? What medicine should I use?<br><br>A4. The treatment of psoriasis nail changes typically involves topical therapy, phototherapy, and systemic medications. Topical therapy involves applying medicated creams or ointments directly to the affected nails. These medications may include corticosteroids, vitamin D analogs, and coal tar. Phototherapy, which involves exposure to ultraviolet light, may also be used to help slow the growth of skin cells and reduce inflammation. Systemic medications, such as biologics, immunosuppressants, and retinoids, may be prescribed for severe cases of psoriasis affecting the nails. It's important to consult a dermatologist for an accurate diagnosis and appropriate treatment plan.	occur in eczema, ringworm and lichen planus on bald hands.<br><br>Q4. How should I treat it? What medicine should I use?<br><br>A4. A detailed medical history, medication history and examination for nail fungus is essential. Careful examination of the body is needed to exclude other skin conditions. Fungal infection, if present, may be treated with oral antifungals.
Evaluation of Physician	
Evaluation Item	Score (1-5, the higher the better)
SkinGPT-4's diagnosis is correct or relevant.	2

SkinGPT-4's description is informative.	5
SkinGPT-4's suggestions are useful.	4
SkinGPT-4 can help doctors with diagnosis.	5
SkinGPT-4 can help patients to understand their disease better.	5
If SkinGPT-4 can be deployed locally, it protects patients' privacy.	5
Willingness to use SkinGPT-4	5

Case Fungal infection-Nail Fungal infection 2

Image ID: Nail Fungal infection2	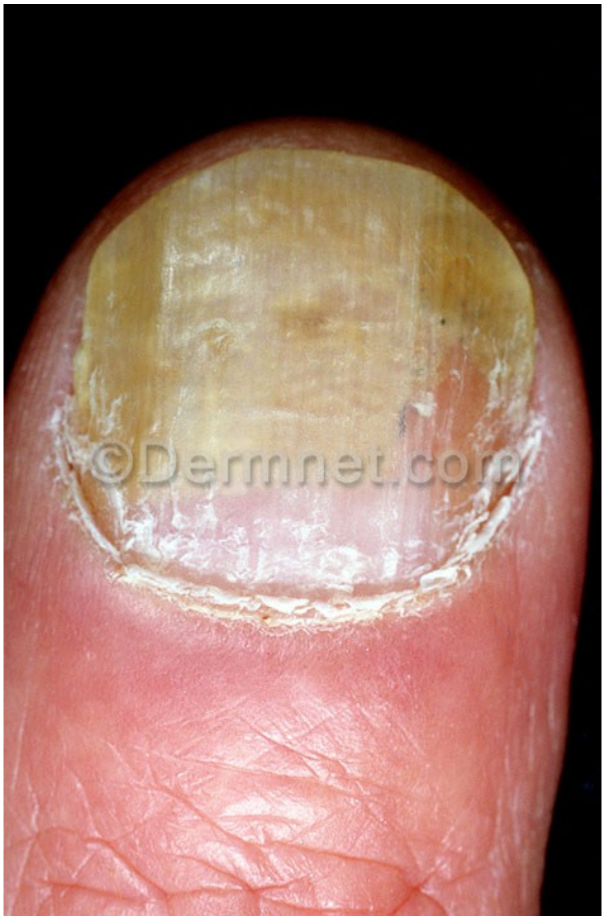
SkinGPT-4	Dermatology Physician
Q1. Could you describe the skin disease in this image for me?<br><br>A1. This could be a skin disease image of distal subungual onychomycosis.<br><br>Q2. You should write a paragraph to list more features you observed in the image.<br><br>A2. The image is of a nail with distal subungual onychomycosis. The nail plate is discolored and there is an abnormal curvature of the nail plate. The nail plate appears thickened and there is a deformity of the nail plate. The nail bed appears pink and there is no onycholysis or nail bed involvement. This is a chronic infection caused by dermatophytes and can result in nail deformity	Q1. Could you describe the skin disease in this image for me?<br><br>A1. Onycholysis or Tinea unguium/onychomycosis<br><br>Q2. You should write a paragraph to list more features you observed in the image.<br><br>A2. Distal nail yellowing and possible separation. Nail surface fragmentation at root.<br><br>Q3. With previous information, please write a paragraph to explain in detail the cause of this skin disease.<br><br>A3. A nail separation is the separation of the nail plate from the nail bed.<br>and certain medications (e.g. norethindrone or tetracycline in the treatment of

and discoloration. The laser treatment given to the nail did not improve the condition.<br><br>Q3. With previous information, please write a paragraph to explain in detail the cause of this skin disease.<br><br>A3. Distal subungual onychomycosis is caused by dermatophytes, a type of fungus that infects the skin. The infection starts as a superficial skin infection and spreads to the nail plate, leading to its deformity and discoloration. The fungus attacks the keratin protein in the nail plate and nail bed, causing the nail to become thick and discolored. As the infection progresses, the nail plate becomes harder and more brittle, leading to the characteristic curvature and deformity seen in this image. The symptoms of distal subungual onychomycosis can be improved with topical and oral antifungal treatments, but the nail may take several months to a year to grow out completely, and complete cure may not always be achieved. The chronic nature of the disease and the poor response to treatment make it a challenging condition to manage.<br><br>Q4. How should I treat it? What medicine should I use?<br><br>A4. Treatment of distal subungual onychomycosis typically involves a combination of topical and oral medications. Topical treatments may include the use of antifungal nail polish, creams or gels that are applied directly to the affected nail. Oral antifungal medications, such as terbinafine, itraconazole, or fluconazole, may also be prescribed to help clear the infection.<br>It's important to note that treatment success varies and may take several months to a year to see improvement. In some cases, the nail may need to be removed surgically if the infection is severe and not responding to treatment.<br>It's important to follow up with your dermatologist for proper diagnosis and treatment plan, as the choice of medication and duration of treatment may vary depending on the severity of the infection and individual response to treatment.	Photogenic nail separation). Topical application of chemical agents, such as solvents, insecticides and insecticides containing phenol or fulvic acid, can cause nail separation.<br>and nail cosmetics (nail hardeners) containing phenols or formalin, can also<br>nail separation.<br><br>Q4. How should I treat it? What medicine should I use?<br><br>A4. A detailed medical history, medication history and examination for nail fungus is essential. Fungal infection, if present, may be treated with oral antifungals.
Evaluation of Physician	

Evaluation Item	Score (1-5, the higher the better)
SkinGPT-4's diagnosis is correct or relevant.	3
SkinGPT-4's description is informative.	5
SkinGPT-4's suggestions are useful.	4
SkinGPT-4 can help doctors with diagnosis.	4
SkinGPT-4 can help patients to understand their disease better.	5
If SkinGPT-4 can be deployed locally, it protects patients' privacy.	5
Willingness to use SkinGPT-4	4

Case Fungal infection-Fungal infection 3

<table>
<tr><td>Image ID: Nail Fungal infection3</td><td>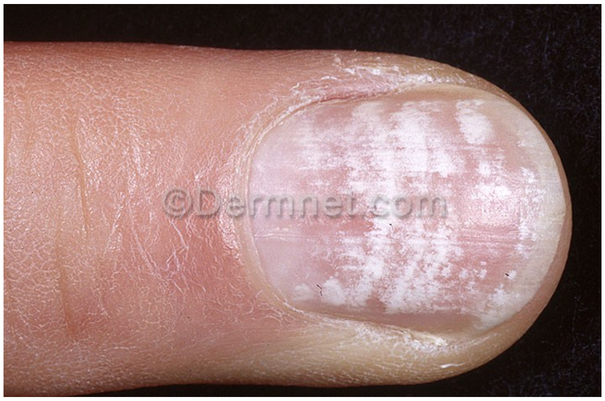
</td></tr>
<tr><th>SkinGPT-4</th><th>Dermatology Physician</th></tr>
<tr><td>Q1. Could you describe the skin disease in this image for me?

A1. This could be a skin disease image of psoriasis.

Q2. You should write a paragraph to list more features you observed in the image.

A2. The image is of psoriasis nails. The features observed in the image include pitting, distal onycholysis, white spots, and discoloration. The nails appear to be deformed and have a rough texture. The color of the nail is uneven, with white spots visible on the surface. The distal portion of the nail appears to be separated from the rest of the nail, causing onycholysis. Overall, the image depicts the changes observed in the nails of a patient with psoriasis.

Q3. With previous information, please write a paragraph to explain in detail the cause of this skin disease.

A3. The cause of psoriasis is not well understood, but it is believed to be an autoimmune disorder. In psoriasis, the immune system attacks healthy cells in the skin, causing them to reproduce rapidly and form thick, scaly plaques on the skin's surface. These plaques can occur anywhere on the body, but are most commonly found on the skin of the elbows, knees, and scalp. The exact trigger for the immune response in psoriasis is not known, but it can be caused by external factors such as infections, stress, and environmental factors.</td><td>Q1. Could you describe the skin disease in this image for me?

A1. Tinea unguium/onychomycosis

Q2. You should write a paragraph to list more features you observed in the image.

A2. White cloudy changes in the whole nail.

Q3. With previous information, please write a paragraph to explain in detail the cause of this skin disease.

A3. Tinea unguium/onychomycosis is a nail infection caused by fungi such as dermatophytes, yeasts and non-dermatophytes.

Q4. How should I treat it? What medicine should I use?

A4. Topical antifungalsare the first-line treatment for many patients. But in this case, systemic antifungal therapy, although associated with a higher incidence of side effects is required.</td></tr>
</table>

Psoriasis is a genetic disorder and can be inherited from a parent, although most people with the genetic predisposition will not develop the disease. The mechanism of psoriasis is complex and involves various genetic and environmental factors, making it a multifactorial disease.<br><br>Q4. How should I treat it? What medicine should I use?<br><br>A4. The treatment of psoriasis depends on the severity and location of the disease, as well as individual preferences and medical history. There are several types of medications used to treat psoriasis, including topical treatments, phototherapy, and systemic medications.<br><br>Topical treatments: These include creams, ointments, and lotions applied directly to the skin. They can help to reduce inflammation and slow the growth of skin cells. Examples include corticosteroids, vitamin D analogs, and salicylic acid.<br>Phototherapy: This involves exposing the skin to ultraviolet light. It can be done in a dermatologist's office or at home with a specialized phototherapy machine. Phototherapy can slow the growth of skin cells and reduce inflammation.<br>Systemic medications: These are taken by mouth or injection and work throughout the body to slow the growth of skin cells. Examples include biologics, methotrexate, and cyclosporine.<br>It is important to consult a dermatologist to determine the best treatment for your specific case of psoriasis. The dermatologist may also recommend a combination of treatments to achieve optimal results.	
Evaluation of Physician	
Evaluation Item	Score (1-5, the higher the better)
SkinGPT-4's diagnosis is correct or relevant.	1
SkinGPT-4's description is informative.	5
SkinGPT-4's suggestions are useful.	5

SkinGPT-4 can help doctors with diagnosis.	1
SkinGPT-4 can help patients to understand their disease better.	5
If SkinGPT-4 can be deployed locally, it protects patients' privacy.	5
Willingness to use SkinGPT-4	1

Case Fungal infection- Nail Fungal infection 4

<table>
<tr><td>Image ID: Nail Fungal infection4</td><td>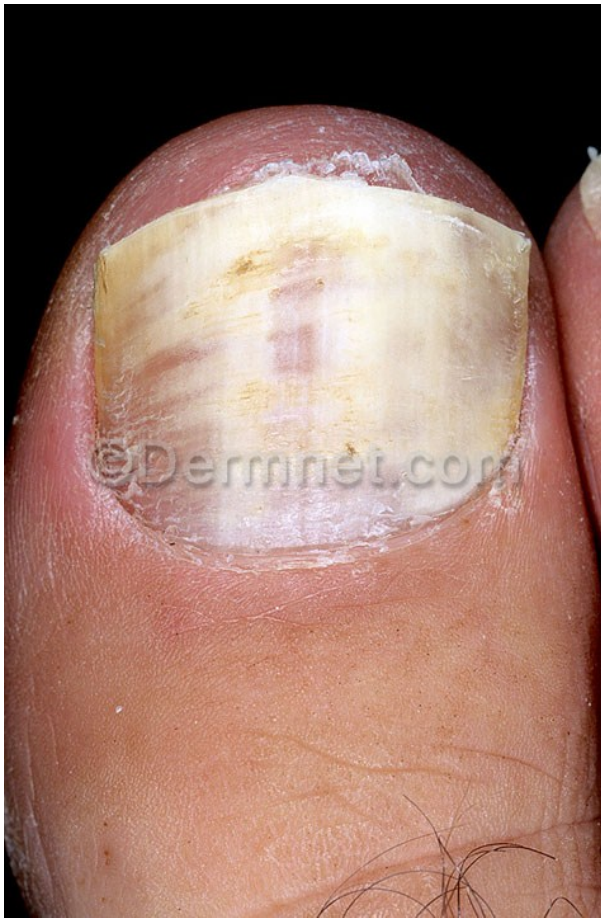
</td></tr>
<tr><th>SkinGPT-4</th><th>Dermatology Physician</th></tr>
<tr><td>Q1. Could you describe the skin disease in this image for me?

A1. This could be a skin disease image of distal subungual onychomycosis.

Q2. You should write a paragraph to list more features you observed in the image.

A2. The image shows a distal subungual onychomycosis, a type of nail fungus that affects the distal (last 25%) of the nail. The nail appears white and crumbly with an uneven surface. The nail plate is thickened and yellowish. The onychomycosis involves the distal half of the nail, which appears white and crumbly with an uneven surface. The nail plate is thickened and yellowish. There is no apparent pitting or onycholysis.</td><td>Q1. Could you describe the skin disease in this image for me?

A1. Tinea unguium/onychomycosis

Q2. You should write a paragraph to list more features you observed in the image.

A2. Onycholysis, yellowing, crumbling and thickening of the fingernails (A), thumb nails

Q3. With previous information, please write a paragraph to explain in detail the cause of this skin disease.

A3. Distal onychomycosis is caused by a fungal infection that affects the nail plate and the surrounding skin.</td></tr>
</table>

Q3. With previous information, please write a paragraph to explain in detail the cause of this skin disease.<br><br>A3. Distal subungual onychomycosis is caused by a fungal infection that affects the nail plate and the surrounding skin. The exact cause of the infection is not known, but it is believed to be caused by a combination of environmental and genetic factors. The fungus attacks the nail plate and the skin surrounding the nail, causing thickening, yellowing, and crumbling of the nail. The fungus can enter the nail through trauma or a break in the skin, and once inside, it multiplies and spreads, causing the symptoms associated with onychomycosis. The infection can be difficult to treat and may require a combination of oral and topical medications, as well as removal of the infected nail. Prevention includes keeping the nails clean and dry, avoiding nail biting or excessive filing, and wearing shoes that allow adequate ventilation.<br><br>Q4. How should I treat it? What medicine should I use?<br><br>A4. Treatment for distal subungual onychomycosis typically involves a combination of oral and topical medications, as well as removal of the infected nail.<br>Oral medications: Oral antifungal medications such as itraconazole, fluconazole, or terbinafine can be prescribed to treat the underlying fungal infection. These medications work by preventing the growth of the fungus and can be taken for several weeks to several months, depending on the severity of the infection.<br>Topical medications: Topical medications, such as ciclopirox, can be applied directly to the nail and surrounding skin to treat the infection. These medications work by slowing down the growth of the fungus and may be used in combination with oral medications.<br>Nail removal: In some cases, the removal of the infected nail may be necessary to completely treat the infection. This can be done by a podiatrist or dermatologist and may be performed in the office under local anesthesia.<br>It is important to note that treatment may take several months and requires patience and compliance with the prescribed treatment plan.	Q4. How should I treat it? What medicine should I use?<br><br>A4. In this case, systemic antifungal therapy is required.

Prevention includes keeping the nails clean and dry, avoiding nail biting or excessive filing, and wearing shoes that allow adequate ventilation.	
Evaluation of Physician	
Evaluation Item	Score (1-5, the higher the better)
SkinGPT-4's diagnosis is correct or relevant.	5
SkinGPT-4's description is informative.	5
SkinGPT-4's suggestions are useful.	4
SkinGPT-4 can help doctors with diagnosis.	4
SkinGPT-4 can help patients to understand their disease better.	5
If SkinGPT-4 can be deployed locally, it protects patients' privacy.	5
Willingness to use SkinGPT-4	4

Case Fungal infection-Nail Fungal infection 5

<table>
<tr><td>Image ID: Nail Fungal infection5</td><td>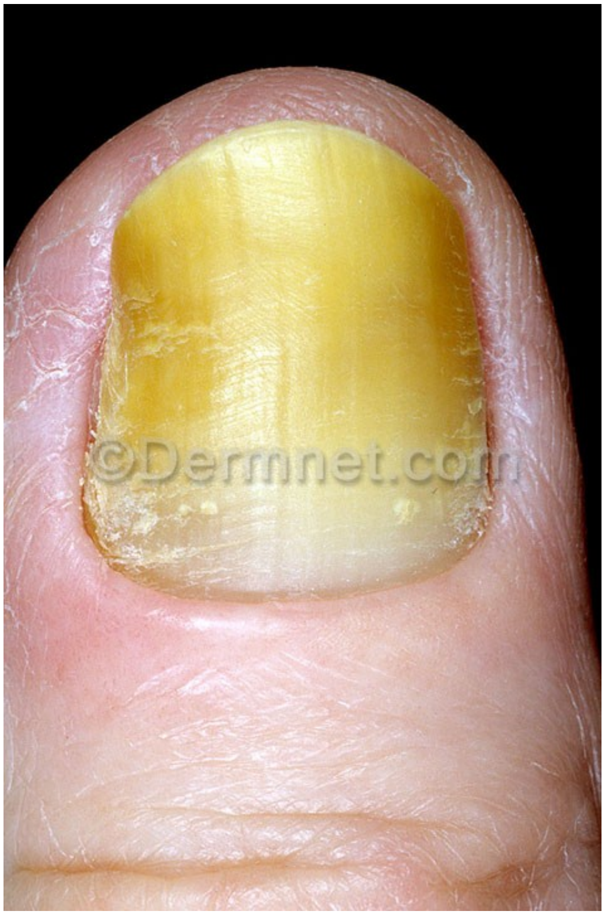
</td></tr>
<tr><th>SkinGPT-4</th><th>Dermatology Physician</th></tr>
<tr><td>Q1. Could you describe the skin disease in this image for me?

A1. This could be a skin disease image of yellow nails.

Q2. You should write a paragraph to list more features you observed in the image.

A2. The image of yellow nails shows a distortion in the normal pigmentation of the nails. The nails appear yellowish in color, and the discoloration affects the entire nail plate, including the lunula (the half moon shaped whitish area at the base of the nail). The yellow color is not a normal pigmentation of the nails, and it may be caused by various underlying conditions such as chronic lymphedema, lichen sclerosus, or yellow nail syndrome. The distal nail plates appear normal in</td><td>Q1. Could you describe the skin disease in this image for me?

A1. Xanthonychia (origin unknown)

Q2. You should write a paragraph to list more features you observed in the image.

A2. Distal end yellowing and thickening.

Q3. With previous information, please write a paragraph to explain in detail the cause of this skin disease.

A3. The cause is unknown and presents as a symmetrical slowing of nail growth and yellowing. Yellow nails are also common in psoriasis, nail fungus, congenital It is also common in psoriasis, nail fungus, congenital thick nails and in the elderly.</td></tr>
</table>

shape, width, and curvature, indicating that the yellow coloration affects only the proximal nail plate. This image could help in the diagnosis of yellow nail syndrome, a rare genetic disorder characterized by yellow, thick, and fragile nails.<br><br>Q3. With previous information, please write a paragraph to explain in detail the cause of this skin disease.<br><br>A3. The cause of yellow nails in this image is likely to be yellow nail syndrome, a rare genetic disorder characterized by yellow, thick, and fragile nails. The disorder is inherited in an autosomal dominant pattern and is caused by mutations in the SPINK5 gene, which encodes for a protein involved in the production of beta-carotene, a precursor to vitamin A. This results in decreased levels of vitamin A and impaired transport of calcium to the nail matrix, leading to the characteristic yellow coloration of the nails. Yellow nail syndrome is also associated with other features such as deformed nails, onycholysis (separation of the nail from the nail bed), and white nail bed (leukonychia). The condition is benign and not associated with any systemic symptoms, but it may be aesthetically bothersome for some individuals. Treatment for yellow nail syndrome is mostly symptomatic, involving the use of topical moisturizers and sunscreen to prevent dryness and sun damage to the nails. In severe cases, nail avulsion (removal of the nail plate) may be considered to relieve symptoms.<br><br>Q4. How should I treat it? What medicine should I use?<br><br>A4. The treatment for yellow nail syndrome is mostly symptomatic and focuses on managing the symptoms of the condition. Treatment options include:<br>1. Moisturizers: Applying moisturizers to the nails and surrounding skin can help to prevent dryness and improve the appearance of the yellow nails.<br>2. Sunscreen: Since individuals with yellow nail syndrome are at an increased risk of developing skin cancer due to the genetic mutation, it is important to use sunscreen to protect the skin and nails from ultraviolet damage.<br>3. Nutritional supplements: Some healthcare providers may recommend taking vitamin A supplements or other nutritional supplements to	Yellow nails can also be caused by yellowing of the nail bed in xanthogranuloma.<br><br>Q4. How should I treat it? What medicine should I use?<br><br>A4. Health check and nail fungus test.

improve nail health and prevent nail abnormalities. It is important to note that there is no specific medication to treat yellow nail syndrome, and the use of topical corticosteroids or other medications may not be effective or may cause adverse effects. It is recommended to consult a dermatologist or other healthcare provider for proper diagnosis and treatment of yellow nail syndrome.	
Evaluation of Physician	
Evaluation Item	Score (1-5, the higher the better)
SkinGPT-4's diagnosis is correct or relevant.	5
SkinGPT-4's description is informative.	5
SkinGPT-4's suggestions are useful.	5
SkinGPT-4 can help doctors with diagnosis.	5
SkinGPT-4 can help patients to understand their disease better.	5
If SkinGPT-4 can be deployed locally, it protects patients' privacy.	5
Willingness to use SkinGPT-4	5

Case Psoriasis 1

Image ID: Psoriasis1	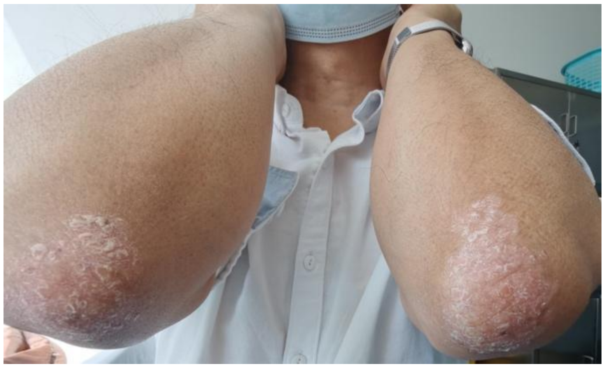
**SkinGPT-4**	**Dermatology Physician**
Q1. Could you describe the skin disease in this image for me?<br><br>A1. This could be a skin disease image of psoriasis.<br><br>Q2. You should write a paragraph to list more features you observed in the image.<br><br>A2. The lesions on the face and hands appear to be a symptom of psoriasis and lichen planus, which are chronic autoimmune skin conditions characterized by the accumulation of dead cells on the skin surface, resulting in the formation of scaly, itchy patches and plaques. The lesions on the face and hands are likely caused by the overproduction of skin cells, leading to the formation of thick, raised patches. The lesions appear to be inflamed and have a rough texture, which is common in psoriasis and lichen planus.<br><br>Q3. With previous information, please write a paragraph to explain in detail the cause of this skin disease.<br><br>A3. The skin disease in this image is likely caused by an overactive immune system, which results in the accumulation of dead skin cells on the skin surface. This overproduction of skin cells is thought to be caused by a combination of genetic and environmental factors. The lesions on the face and hands are likely caused by the excessive production of skin cells, which leads to the formation of thick, raised patches. The autoimmune response that causes psoriasis and lichen planus is still not well understood, but it is	Q1. Could you describe the skin disease in this image for me?<br><br>A1. Psoriasis or dermatitis.<br><br>Q2. You should write a paragraph to list more features you observed in the image.<br><br>A2. The sharp demarcation and silvery scale.<br><br>Q3. With previous information, please write a paragraph to explain in detail the cause of this skin disease.<br><br>A3. Psoriasis is an immune-mediated polygenic skin disorder. Various environmental triggering factors(e.g. trauma, infections or medications, may elicit disease in predisposed individuals). The characteristic lesion is a sharply demarcated erythematous plaque with micaceous scale, and the plaques may be localized or widespread in distribution. Psoriasis is a systemic disease process in which up to 20–30% of the patients have or will develop psoriatic arthritis. In addition, in patients with moderate to severe psoriasis, there is an increased relative risk for metabolic syndrome and atherosclerotic cardiovascular disease. The current treatments, although often effective, do not provide a satisfactory long-term solution.<br>Typical histologic findings include acanthosis with elongated rete ridges, hypogranulosis, hyper- and parakeratosis, dilated blood vessels and a perivascular infiltrate of lymphocytes with neutrophils singly or within aggregates in the epidermis. Phototherapy, methotrexate,

thought to involve the T cells and immune system components that regulate the growth and shedding of skin cells. These disorders are often associated with other chronic inflammatory conditions, such as psoriatic arthritis and metabolic syndrome, which may share common underlying mechanisms with psoriasis and lichen planus.<br><br>Q4. How should I treat it? What medicine should I use?<br><br>A4. Treatment for psoriasis and lichen planus typically involves a combination of topical and systemic therapies.<br>Topical treatments include:<br>* Corticosteroids to reduce inflammation and suppress the immune system<br>* Vitamin D analogs, such as calcipotriol and calcitriol, which help to slow the growth of skin cells<br>* Moisturizers to help prevent dryness and itching<br>Systemic therapies include:<br>* Biologic drugs, such as adalimumab, ustekinumab, and secukinumab, which target specific molecules in the immune system to reduce inflammation<br>* Medications that suppress the immune system, such as methotrexate and cyclosporine<br>* Biologic agents that target specific molecules in the immune system, such as tacrolimus and pimecrolimus.<br>The choice of treatment depends on the severity of the disease, the affected area, and the individual's overall health. It's important to consult with a dermatologist or healthcare professional for the most appropriate treatment options.	cyclosporine and "biologic" therapies that target key immune effector cells and cytokines lead to significant clinical improvement.<br><br>Q4. How should I treat it? What medicine should I use?<br><br>A4. The type of treatment depends on the severity of the patient's condition as well as their willingness to undergo treatment and their financial situation. In this case, *topical Vitamin D3 analogues*, *Corticosteroids* and *Topical retinoids* (*tazarotene*) are the first line medications. Biologic drugs, such as *Adalimumab*, *Ustekinumab*, and *Secukinumab*, which target specific molecules in the immune system are more effective to reduce inflammation.
Evaluation of Physician	
Evaluation Item	Score (1-5, the higher the better)
SkinGPT-4's diagnosis is correct or relevant.	5
SkinGPT-4's description is informative.	5
SkinGPT-4's suggestions are useful.	5

SkinGPT-4 can help doctors with diagnosis.	5
SkinGPT-4 can help patients to understand their disease better.	5
If SkinGPT-4 can be deployed locally, it protects patients’ privacy.	5
Willingness to use SkinGPT-4	5

Case Psoriasis 2

Image ID: Psoriasis2	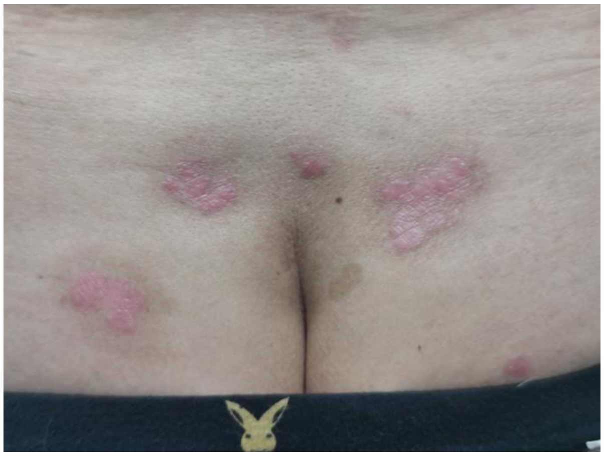
SkinGPT-4	Dermatology Physician
Q1. Could you describe the skin disease in this image for me?<br><br>A1. Psoriasis.<br><br>Q2. You should write a paragraph to list more features you observed in the image.<br><br>A2. The patient has a chronic inflammatory skin condition characterized by red, scaly, raised plaques on the skin. The skin lesions are commonly found on the elbows, knees, and scalp, but can occur anywhere on the body. The lesions are usually itchy and can be painful. Psoriasis is a chronic condition that can be treated with topical medications, light therapy, and systemic medications. The condition is not contagious and does not pose a risk to the patient's overall health.<br><br>Q3. With previous information, please write a paragraph to explain in detail the cause of this skin disease.<br><br>A3. The exact cause of psoriasis is not known, but it is thought to be an autoimmune disorder. In psoriasis, the immune system attacks healthy cells in the skin and causes them to produce too much keratin, which results in the formation of the thick, scaly plaques that are characteristic of the condition. It is also believed that psoriasis may be triggered by certain factors, such as stress, infections, and certain medications. Genetics may also play a role in the development of psoriasis,	Q1. Could you describe the skin disease in this image for me?<br><br>A1. Psoriasis.<br><br>Q2. You should write a paragraph to list more features you observed in the image.<br><br>A2. Plaques, with the sharp demarcation and scale.<br><br>Q3. With previous information, please write a paragraph to explain in detail the cause of this skin disease.<br><br>A3. Psoriasis is an immune-mediated polygenic skin disorder. Various environmental triggering factors(e.g. trauma, infections or medications, may elicit disease in predisposed individuals). The characteristic lesion is a sharply demarcated erythematous plaque with micaceous scale, and the plaques may be localized or widespread in distribution. Psoriasis is a systemic disease process in which up to 20–30% of the patients have or will develop psoriatic arthritis. In addition, in patients with moderate to severe psoriasis, there is an increased relative risk for metabolic syndrome and atherosclerotic cardiovascular disease. The current treatments, although often effective, do not provide a satisfactory long-term solution.<br>Typical histologic findings include acanthosis with elongated rete ridges, hypogranulosis, hyper- and

<table>
<tr><td>as the condition tends to run in families.

Q4. How should I treat it? What medicine should I use?

A4. Psoriasis can be treated with a variety of different approaches, depending on the severity and location of the symptoms. Treatment options for psoriasis include:
1. Topical treatments: These include creams, ointments, and lotions that are applied directly to the skin. Over-the-counter creams containing coal tar, salicylic acid, or capsaicin can help to reduce itching and scaling. Prescription topical treatments, such as corticosteroids, vitamin D analogs, and calcineurin inhibitors, are also available and can be more effective than over-the-counter products.
2. Phototherapy: This involves exposing the skin to ultraviolet light on a regular basis. Phototherapy can be done at home with a special lamp or in a dermatologist’s office.
3. Systemic medications: These are medications that are taken by mouth or injection and work throughout the body to suppress the immune system and reduce inflammation. Systemic medications may be used for more severe cases of psoriasis, or when topical treatments have not been effective.
4. Biologics: These are a newer class of medications that target specific parts of the immune system and are given by injection or infusion. Biologics have been shown to be effective in treating moderate.</td><td>parakeratosis, dilated blood vessels and a perivascular infiltrate of lymphocytes with neutrophils singly or within aggregates in the epidermis. Phototherapy, methotrexate, cyclosporine and “biologic” therapies that target key immune effector cells and cytokines lead to significant clinical improvement.

Q4. How should I treat it? What medicine should I use?

A4. The type of treatment depends on the severity of the patient's condition as well as their willingness to undergo treatment and their financial situation. In this case, *topical Vitamin D3 analogues*, *Corticosteroids* and *Topical retinoids* (*tazarotene*) are the first line medications. Biologic drugs, such as adalimumab, ustekinumab, and secukinumab, which target specific molecules in the immune system are more effective to reduce inflammation.</td></tr>
<tr><td colspan="2">Evaluation of Physician</td></tr>
<tr><td>Evaluation Item</td><td>Score (1-5, the higher the better)</td></tr>
<tr><td>SkinGPT-4’s diagnosis is correct or relevant.</td><td>5</td></tr>
<tr><td>SkinGPT-4’s description is informative.</td><td>5</td></tr>
<tr><td>SkinGPT-4’s suggestions are useful.</td><td>5</td></tr>
<tr><td>SkinGPT-4 can help doctors with diagnosis.</td><td>5</td></tr>
<tr><td>SkinGPT-4 can help patients to understand their disease better.</td><td>5</td></tr>
</table>

If SkinGPT-4 can be deployed locally, it protects patients' privacy.	5
Willingness to use SkinGPT-4	5

Case Psoriasis 3

<table>
<tr><td>Image ID: Psoriasis3</td><td>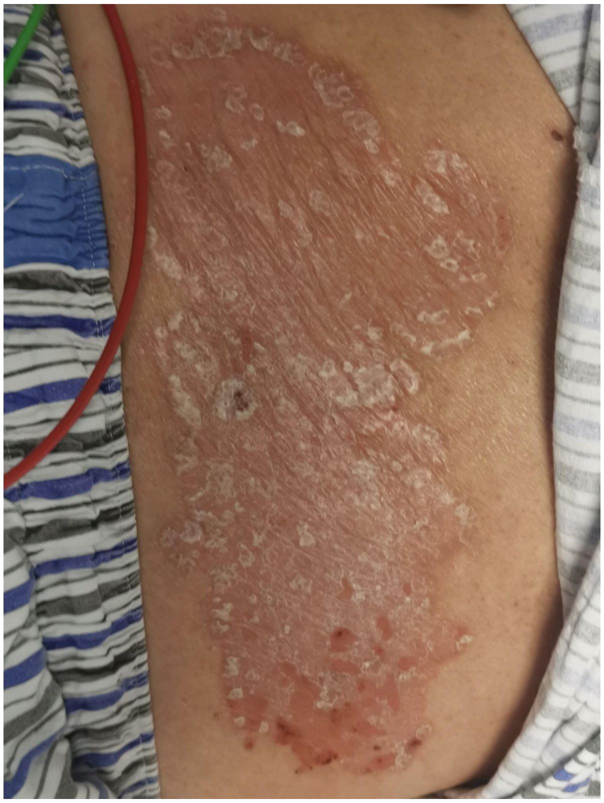</td></tr>
<tr><th>SkinGPT-4</th><th>Dermatology Physician</th></tr>
<tr><td>Q1. Could you describe the skin disease in this image for me?

A1. Psoriasis.

Q2. You should write a paragraph to list more features you observed in the image.

A2. The patient has a inflammatory skin with scaly, raised plaques on the skin.

Q3. With previous information, please write a paragraph to explain in detail the cause of this skin disease.

A3. Psoriasis is caused by an overactive immune system. The immune system is responsible for protecting the body against infections and other harmful substances, but in people with psoriasis, the immune system mistakenly attacks healthy skin cells and causes them to grow too quickly. This leads to the formation of red, scaly patches</td><td>Q1. Could you describe the skin disease in this image for me?

A1. Psoriasis.

Q2. You should write a paragraph to list more features you observed in the image.

A2. Patching, with the sharp demarcation and silvery scale.

Q3. With previous information, please write a paragraph to explain in detail the cause of this skin disease.

A3. Psoriasis is an immune-mediated polygenic skin disorder. Various environmental triggering factors(e.g. trauma, infections or medications, may elicit disease in predisposed individuals). The characteristic lesion is a sharply demarcated erythematous plaque with micaceous scale, and the plaques may be localized or widespread in</td></tr>
</table>

on the skin. The exact cause of the overactive immune system in psoriasis is not known, but it is thought to be related to genetics, environmental factors, and other medical conditions such as obesity, high blood pressure, and diabetes. There is also evidence that psoriasis may be triggered by certain infections or stress.<br><br>Q4. How should I treat it? What medicine should I use?<br><br>A4. Treatment for psoriasis depends on the severity of the condition and the type of psoriasis. The most common treatments for psoriasis include:<br>1. Topical creams and ointments: These are applied directly to the skin and are used to treat mild to moderate psoriasis. They may include corticosteroids, vitamin D analogues, and coal tar.<br>2. Phototherapy: This involves exposing the skin to ultraviolet light, either in a specialized clinic or at home using a phototherapy device. Phototherapy is effective for treating moderate to severe psoriasis.<br>3. Systemic medications: These are taken by mouth or injection and are used to treat moderate to severe psoriasis. They may include methotrexate, biologic agents, and immunosuppressive drugs.<br>4. Combination therapy: This involves using a combination of topical creams, phototherapy, and systemic medications to treat severe psoriasis.<br><br>The best treatment for psoriasis will depend on the individual, and it may take some trial and error to find the most effective treatment. It is important to work with a healthcare provider to develop a treatment plan that is tailored to your needs and preferences.	distribution. Psoriasis is a systemic disease process in which up to 20–30% of the patients have or will develop psoriatic arthritis. In addition, in patients with moderate to severe psoriasis, there is an increased relative risk for metabolic syndrome and atherosclerotic cardiovascular disease. The current treatments, although often effective, do not provide a satisfactory long-term solution.<br>Typical histologic findings include acanthosis with elongated rete ridges, hypogranulosis, hyper- and parakeratosis, dilated blood vessels and a perivascular infiltrate of lymphocytes with neutrophils singly or within aggregates in the epidermis. Phototherapy, methotrexate, cyclosporine and “biologic” therapies that target key immune effector cells and cytokines lead to significant clinical improvement.<br><br>Q4. How should I treat it? What medicine should I use?<br><br>A4. The type of treatment depends on the severity of the patient's condition as well as their willingness to undergo treatment and their financial situation. In this case, *topical Vitamin D3 analogues*, *Corticosteroids* and *Topical retinoids* (*tazarotene*) are the first line medications. Biologic drugs, such as adalimumab, ustekinumab, and secukinumab, which target specific molecules in the immune system are more effective to reduce inflammation.
Evaluation of Physician	
Evaluation Item	Score (1-5, the higher the better)
SkinGPT-4’s diagnosis is correct or relevant.	5
SkinGPT-4’s description is informative.	5
SkinGPT-4’s suggestions are useful.	5

SkinGPT-4 can help doctors with diagnosis.	5
SkinGPT-4 can help patients to understand their disease better.	5
If SkinGPT-4 can be deployed locally, it protects patients' privacy.	5
Willingness to use SkinGPT-4	5

Case Psoriasis 4

<table>
<tr><td>Image ID: Psoriasis4</td><td>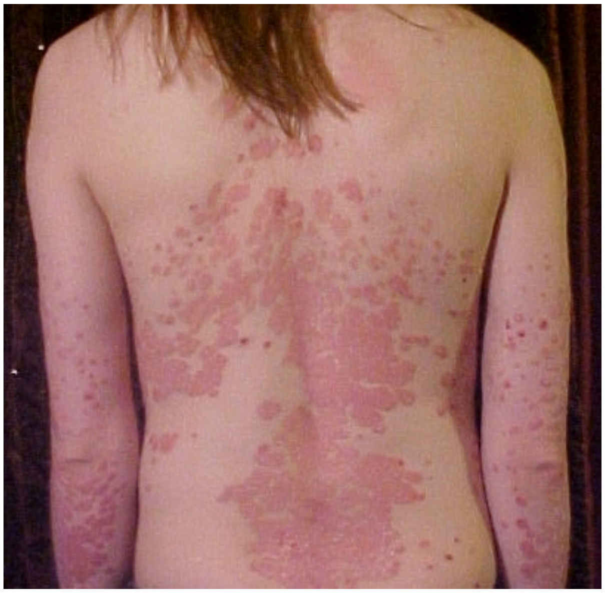</td></tr>
<tr><th>SkinGPT-4</th><th>Dermatology Physician</th></tr>
<tr><td>Q1. Could you describe the skin disease in this image for me?

A1. This could be a skin disease image of psoriasis.

Q2. You should write a paragraph to list more features you observed in the image.

A2. The image shows a person with psoriasis, an autoimmune skin condition characterized by red, scaly, and sometimes itchy patches on the skin. The lesions can appear on any part of the body and can be mistaken for other skin conditions like eczema or dermatitis. The affected skin can be treated with topical corticosteroids, light therapy, or systemic medications. The underlying cause is not well understood and there is no cure, but treatment can help manage symptoms and improve quality of life

Q3. With previous information, please write a paragraph to explain in detail the cause of this skin disease.

A3. Psoriasis is an autoimmune skin condition in which the immune system attacks healthy cells in the skin, causing them to reproduce rapidly and form the characteristic scaly lesions. The exact</td><td>Q1. Could you describe the skin disease in this image for me?

A1. Psoriasis.

Q2. You should write a paragraph to list more features you observed in the image.

A2. The dark-reddish patches and patches, with the sharp demarcation. The lesions are partly symmetrically distributed.

Q3. With previous information, please write a paragraph to explain in detail the cause of this skin disease.

A3. Psoriasis is an immune-mediated polygenic skin disorder. Various environmental triggering factors(e.g. trauma, infections or medications, may elicit disease in predisposed individuals). The characteristic lesion is a sharply demarcated erythematous plaque with micaceous scale, and the plaques may be localized or widespread in distribution. Psoriasis is a systemic disease process in which up to 20–30% of the patients have or will develop psoriatic arthritis. In addition, in patients with moderate to severe psoriasis, there is an increased relative risk for metabolic syndrome and atherosclerotic cardiovascular</td></tr>
</table>

<table>
<tr><td>cause of psoriasis is not fully understood, but it is thought to be related to an overactive immune system and genetic factors. The condition is not contagious and is not caused by poor hygiene or lifestyle factors. There are several types of psoriasis, including plaque, guttate, inverse, and pustular, each with its own distinct characteristics. Treatment options for psoriasis include topical corticosteroids, vitamin D analogues, biologics, and phototherapy, depending on the severity and type of psoriasis. While there is no cure for psoriasis, treatment can help manage symptoms and improve quality of life for those affected by the condition.

Q4. How should I treat it? What medicine should I use?

A4. Treatment options for psoriasis vary depending on the severity and type of psoriasis, as well as individual factors such as age, medical history, and lifestyle. Some common treatment options for psoriasis include:
* Topical corticosteroids: These are creams or ointments applied directly to the skin to reduce inflammation and suppress the immune system. They can be effective for mild to moderate psoriasis, but may cause side effects such as skin thinning or increased risk of infections.
* Vitamin D analogues: These are creams or ointments that work by slowing down the growth of skin cells. They can be effective for mild to moderate psoriasis and are often used as a first-line treatment.
* Biologics: These are injectable medications that target specific parts of the immune system to reduce inflammation. They are effective for moderate to severe psoriasis and may be used in combination with other treatments.
* Phototherapy: This involves exposing the skin to ultraviolet light, either alone or in combination with topical medications. It is effective for mild to moderate psoriasis and can be administered in a doctor’s office or at home.</td>
<td>disease. The current treatments, although often effective, do not provide a satisfactory long-term solution.
Typical histologic findings include acanthosis with elongated rete ridges, hypogranulosis, hyper- and parakeratosis, dilated blood vessels and a perivascular infiltrate of lymphocytes with neutrophils singly or within aggregates in the epidermis. Phototherapy, methotrexate, cyclosporine and “biologic” therapies that target key immune effector cells and cytokines lead to significant clinical improvement.

Q4. How should I treat it? What medicine should I use?

A4. The type of treatment depends on the severity of the patient's condition as well as their willingness to undergo treatment and their financial situation.
In this case, biologic drugs, such as adalimumab, ustekinumab, and secukinumab, which target specific molecules in the immune system are more effective to reduce inflammation are the first line medications.</td></tr>
<tr><td colspan="2">Evaluation of Physician</td></tr>
<tr><td>Evaluation Item</td><td>Score (1-5, the higher the better)</td></tr>
<tr><td>SkinGPT-4’s diagnosis is correct or relevant.</td><td>5</td></tr>
</table>

SkinGPT-4's description is informative.	5
SkinGPT-4's suggestions are useful.	4
SkinGPT-4 can help doctors with diagnosis.	5
SkinGPT-4 can help patients to understand their disease better.	5
If SkinGPT-4 can be deployed locally, it protects patients' privacy.	5
Willingness to use SkinGPT-4	5

Case Psoriasis 5

<table>
<tr><td>Image ID: Psoriasis5</td><td>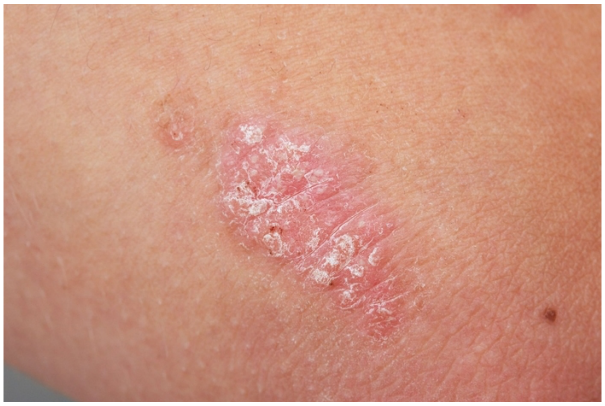</td></tr>
<tr><td>SkinGPT-4</td><td>Dermatology Physician</td></tr>
<tr><td>Q1. Could you describe the skin disease in this image for me?

A1. This could be a skin disease image of lichen planus.

Q2. You should write a paragraph to list more features you observed in the image.

A2. The image shows a chronic inflammatory skin condition called lichen planus. The lesions are erythematous and shiny, with white-gray borders and a depressed center. The disease typically affects middle aged adults and the cause is unknown. It often involves the skin, but can also affect the nails and mucous membranes. The condition is often treated with corticosteroids and immunosuppressive drugs. The disease can be difficult to treat and can take months to years to resolve. The images shows erythema which is a sign of inflammation, shiny skin which is a sign of keratotic plug, and white-gray borders which is a sign of psoriasis.

Q3. With previous information, please write a paragraph to explain in detail the cause of this skin disease.

A3. The cause of lichen planus is not known, but it is thought to be an autoimmune disorder. It is characterized by the presence of T cells and immunoglobulins in the lesions. The disease can also be triggered by certain medications, such as sulfa drugs and thiol-containing drugs. The lesions of lichen planus are often found on the skin, but it</td><td>Q1. Could you describe the skin disease in this image for me?

A1. Psoriasis

Q2. You should write a paragraph to list more features you observed in the image.

A2.

Q3. With previous information, please write a paragraph to explain in detail the cause of this skin disease.

A3. Psoriasis is an immune-mediated polygenic skin disorder. Various environmental triggering factors(e.g. trauma, infections or medications, may elicit disease in predisposed individuals). The characteristic lesion is a sharply demarcated erythematous plaque with micaceous scale, and the plaques may be localized or widespread in distribution. Psoriasis is a systemic disease process in which up to 20–30% of the patients have or will develop psoriatic arthritis. In addition, in patients with moderate to severe psoriasis, there is an increased relative risk for metabolic syndrome and atherosclerotic cardiovascular disease. The current treatments, although often effective, do not provide a satisfactory long-term solution.
Typical histologic findings include acanthosis with elongated rete ridges, hypogranulosis, hyper- and parakeratosis, dilated blood vessels and a perivascular infiltrate of lymphocytes with neutrophils singly or within aggregates in the</td></tr>
</table>

can also affect the nails and mucous membranes. The lesions are usually asymptomatic but can be itchy or painful. The disease is more common in individuals with a family history of autoimmune disorders, such as rheumatoid arthritis, lupus, and psoriasis. The lesions of lichen planus are usually treated with corticosteroids, but there is no cure for the disease. The disease can take months to years to resolve.<br><br>Q4. How should I treat it? What medicine should I use?<br><br>A4. The treatment of lichen planus is aimed at reducing symptoms and inflammation, and may include:<br>* Corticosteroids: Topical or or oral corticosteroids may be prescribed to reduce inflammation and itching.<br>* Immunomodulators: Medications such as methotrexate, cyclosporine, and mycophenolate mofetil may be used to suppress the immune system and reduce inflammation.<br>* Phototherapy: Ultraviolet light therapy may be used to improve skin symptoms.<br>* Emollients: Moisturizing creams or ointments may be used to soothe the skin and reduce itching.<br>It is important to note that the treatment options for lichen planus are not always effective, and the disease may take months to years to resolve on its own.	epidermis. Phototherapy, methotrexate, cyclosporine and "biologic" therapies that target key immune effector cells and cytokines lead to significant clinical improvement.<br><br>Q4. How should I treat it? What medicine should I use?<br><br>A4. The type of treatment depends on the severity of the patient's condition as well as their willingness to undergo treatment and their financial situation. In this case, *topical Vitamin D3 analogues*, *Corticosteroids* and *Topical retinoids* (*tazarotene*) are the first line medications. Biologic drugs, such as adalimumab, ustekinumab, and secukinumab, which target specific molecules in the immune system are more effective to reduce inflammation.
Evaluation of Physician	
Evaluation Item	Score (1-5, the higher the better)
SkinGPT-4's diagnosis is correct or relevant.	1
SkinGPT-4's description is informative.	5
SkinGPT-4's suggestions are useful.	5
SkinGPT-4 can help doctors with diagnosis.	5
SkinGPT-4 can help patients to understand their disease better.	5

If SkinGPT-4 can be deployed locally, it protects patients' privacy.	5
Willingness to use SkinGPT-4	1

Case Psoriasis 6

Image ID: Psoriasis6	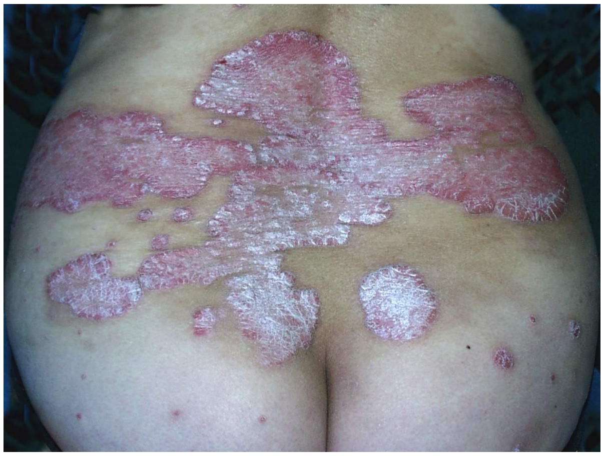
**SkinGPT-4**	**Dermatology Physician**
Q1. Could you describe the skin disease in this image for me?<br><br>A1. This could be a skin disease image of psoriasis.<br><br>Q2. You should write a paragraph to list more features you observed in the image.<br><br>A2. This image shows psoriasis on the skin. It appears as red, scaly patches with white flakes on top. The patches can be itchy and sometimes painful.<br><br>Q3. With previous information, please write a paragraph to explain in detail the cause of this skin disease.<br><br>A3. Psoriasis is caused by an overactive immune system that causes the skin cells to reproduce too quickly and build up on the skin in the form of red, scaly patches. The exact cause of psoriasis is not fully understood, but it is believed to be related to genetics, the environment, and the immune system. There are several genetic markers that have been identified in people with psoriasis, suggesting a genetic predisposition to the disease. The immune system in people with psoriasis produces certain types of white blood cells that attack the skin cells and cause inflammation, which can lead to the development of psoriasis lesions. Environmental factors such as stress, infections, and skin injuries can also trigger	Q1. Could you describe the skin disease in this image for me?<br><br>A1. Psoriasis<br><br>Q2. You should write a paragraph to list more features you observed in the image.<br><br>A2. Red patches on the back with thicken silvery scales.<br><br>Q3. With previous information, please write a paragraph to explain in detail the cause of this skin disease.<br><br>A3. Psoriasis is an immune-mediated polygenic skin disorder. Various environmental triggering factors(e.g. trauma, infections or medications, may elicit disease in predisposed individuals). The characteristic lesion is a sharply demarcated erythematous plaque with micaceous scale, and the plaques may be localized or widespread in distribution. Psoriasis is a systemic disease process in which up to 20–30% of the patients have or will develop psoriatic arthritis. In addition, in patients with moderate to severe psoriasis, there is an increased relative risk for metabolic syndrome and atherosclerotic cardiovascular disease. The current treatments, although often effective, do not provide a satisfactory long-term solution.<br>Typical histologic findings include acanthosis with elongated rete ridges, hypogranulosis, hyper- and

psoriasis flares.<br><br>Q4. How should I treat it? What medicine should I use?<br><br>A4. There are several treatment options available for psoriasis, depending on the severity and location of the symptoms. The most common treatments include:<br>1. Topical creams and ointments: These are applied directly to the skin and can help reduce inflammation and slow down the growth of skin cells. They may contain ingredients such as corticosteroids, vitamin D analogs, or coal tar.<br>2. Phototherapy: This involves exposure to ultraviolet light, either in a specialized clinic or at home using a sun lamp. Phototherapy can help slow down the growth of skin cells and reduce inflammation.<br>3. Systemic medications: These are oral medications that can help control the immune system and reduce inflammation throughout the body. They may include immunosuppressants, biologic drugs, or retinoids.<br>4. Combination therapy: This involves using a combination of topical creams and systemic medications to achieve better results.<br>It is important to work with a healthcare professional to determine the best treatment plan for your specific case of psoriasis. Treatment options may also depend on the severity of the symptoms, the location of the psoriasis, and any related health conditions you may have.	parakeratosis, dilated blood vessels and a perivascular infiltrate of lymphocytes with neutrophils singly or within aggregates in the epidermis. Phototherapy, methotrexate, cyclosporine and "biologic" therapies that target key immune effector cells and cytokines lead to significant clinical improvement.<br><br>Q4. How should I treat it? What medicine should I use?<br><br>A4. The type of treatment depends on the severity of the patient's condition as well as their willingness to undergo treatment and their financial situation. In this case, *topical Vitamin D3 analogues*, *Corticosteroids* and *Topical Retinoids* (*tazarotene*) are the first line medications. If the rash covers a large area, biologic drugs, such as *Adalimumab*, *Ustekinumab*, and *Secukinumab*, which target specific molecules in the immune system are more effective to reduce inflammation.
Evaluation of Physician	
Evaluation Item	Score (1-5, the higher the better)
SkinGPT-4's diagnosis is correct or relevant.	5
SkinGPT-4's description is informative.	5
SkinGPT-4's suggestions are useful.	5
SkinGPT-4 can help doctors with diagnosis.	5
SkinGPT-4 can help patients to understand their disease better.	5

If SkinGPT-4 can be deployed locally, it protects patients' privacy.	5
Willingness to use SkinGPT-4	5

Case Psoriasis 7

<table>
<tr><td>Image ID: Psoriasis7</td><td>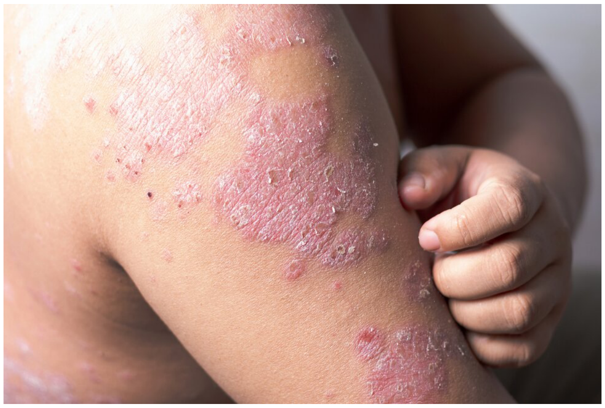</td></tr>
<tr><td>SkinGPT-4</td><td>Dermatology Physician</td></tr>
<tr><td>Q1. Could you describe the skin disease in this image for me?

A1. psoriasis and lichen planus and lupus and impetigo and erythema

Q2. You should write a paragraph to list more features you observed in the image.

A2. I observed several features in the image, including the presence of pustules and papules on the arm and chest, as well as erythema and desquamation of the skin. I also noticed the presence of vesicles on the scalp and the face, which are characteristic of scalp psoriasis. The skin lesions appear to be well demarcated and the skin is heavily scaled, which is a common feature of chronic plaque psoriasis. Additionally, there is an absence of nail pitting, which is a characteristic feature of psoriatic arthritis. Overall, the image depicts a patient with a diagnosis of chronic plaque psoriasis, which is a long-lasting and recurrent autoimmune disorder that affects the skin and nails.

Q3. With previous information, please write a paragraph to explain in detail the cause of this skin disease.

A3. The cause of this skin disease is not clear. It is believed to be an autoimmune disorder, which occurs when the body's immune system mistakenly attacks healthy cells and tissues. This can lead to inflammation, redness, and the</td><td>Q1. Could you describe the skin disease in this image for me?

A1. Psoriasis

Q2. You should write a paragraph to list more features you observed in the image.

A2. Red patches on the arm with thicken silvery scales.

Q3. With previous information, please write a paragraph to explain in detail the cause of this skin disease.

A3. Psoriasis is an immune-mediated polygenic skin disorder. Various environmental triggering factors(e.g. trauma, infections or medications, may elicit disease in predisposed individuals). The characteristic lesion is a sharply demarcated erythematous plaque with micaceous scale, and the plaques may be localized or widespread in distribution. Psoriasis is a systemic disease process in which up to 20–30% of the patients have or will develop psoriatic arthritis. In addition, in patients with moderate to severe psoriasis, there is an increased relative risk for metabolic syndrome and atherosclerotic cardiovascular disease. The current treatments, although often effective, do not provide a satisfactory long-term solution.
Typical histologic findings include acanthosis with elongated rete ridges, hypogranulosis, hyper- and parakeratosis, dilated blood vessels and a perivascular infiltrate of lymphocytes with</td></tr>
</table>

formation of lesions on the skin. The disease is also thought to have a genetic component, as it can run in families. The exact triggers that cause the immune system to attack healthy cells are not well understood, but it is believed to be related to environmental factors, such as stress, infections, and changes in temperature or humidity. The skin lesions can be treated with various topical and systemic therapies, including creams, ointments, and oral medications, but there is currently no cure for the disease. The goal of treatment is to alleviate symptoms and improve the appearance of the skin. In severe cases, treatment may also involve managing any associated symptoms, such as pain, itching, or inflammation, and preventing any complications from occurring.<br>It is important to note that the skin condition in the image is a fictional representation and the cause of the skin condition in the image is not specified. The description of the skin condition is used for illustrative purposes only.<br><br>Q4. How should I treat it? What medicine should I use?<br><br>A4. The treatment for this skin disease will depend on the specific diagnosis and the severity of the symptoms. A dermatologist, a doctor who specializes in skin conditions, will be able to make the proper diagnosis and recommend the appropriate treatment.<br>In general, the most common treatment options for this skin disease are topical treatments, such as creams and ointments, which are applied directly to the skin. These treatments can help to reduce inflammation, redness, and the formation of lesions.<br>In addition to topical treatments, systemic medications, such as oral antibiotics, steroids, or immunosuppressive drugs, may be prescribed to treat the underlying condition. Phototherapy, which involves exposing the skin to ultraviolet light, may also be used to treat the skin condition.<br><br>It is important to note that the skin condition in the image is a fictional representation and the cause of the skin condition in the image is not specified. The description of the skin condition is used for illustrative purposes only. The best treatment for the skin condition will depend on the specific diagnosis and the severity of the symptoms. It is	neutrophils singly or within aggregates in the epidermis. Phototherapy, methotrexate, cyclosporine and “biologic” therapies that target key immune effector cells and cytokines lead to significant clinical improvement.<br><br>Q4. How should I treat it? What medicine should I use?<br><br>A4. The type of treatment depends on the severity of the patient's condition as well as their willingness to undergo treatment and their financial situation. In this case, *topical Vitamin D3 analogues*, *Corticosteroids* and *Topical Retinoids* (*tazarotene*) are the first line medications. If the rash covers a large area, biologic drugs, such as *Adalimumab*, *Ustekinumab*, and *Secukinumab*, which target specific molecules in the immune system are more effective to reduce inflammation.

recommended to consult a dermatologist to get a proper diagnosis and treatment plan.	
Evaluation of Physician	
Evaluation Item	Score (1-5, the higher the better)
SkinGPT-4's diagnosis is correct or relevant.	3
SkinGPT-4's description is informative.	4
SkinGPT-4's suggestions are useful.	2
SkinGPT-4 can help doctors with diagnosis.	3
SkinGPT-4 can help patients to understand their disease better.	2
If SkinGPT-4 can be deployed locally, it protects patients' privacy.	5
Willingness to use SkinGPT-4	1

Case Psoriasis 8

Image ID: Psoriasis8	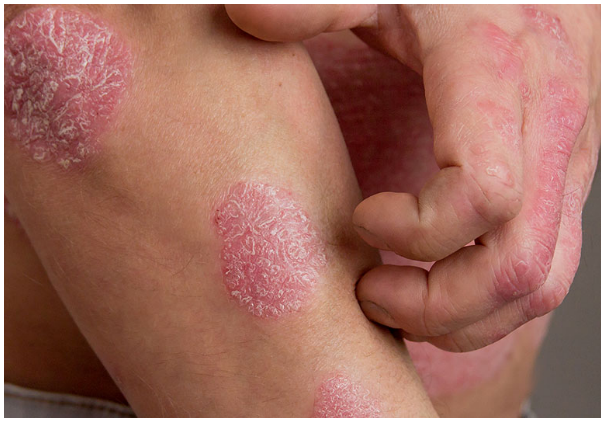
**SkinGPT-4**	**Dermatology Physician**
Q1. Could you describe the skin disease in this image for me?<br><br>A1. psoriasis and lichen planus<br><br>Q2. You should write a paragraph to list more features you observed in the image.<br><br>A2. In the image, there are several features that can be observed. Firstly, the man has psoriasis on his leg, which is characterized by red, scaly, and raised patches on the skin. Secondly, he has lichen planus on his wrist, which is a chronic autoimmune disease that causes rough, brownish or white bumps on the skin. Thirdly, there are several pustules on his arms and legs, which are small, pus - filled blisters that occur in many diseases, including acne and psoriasis. Lastly, there is a rash on his torso, which could be caused by various factors, such as an allergic reaction or eczema.<br><br>Q3. With previous information, please write a paragraph to explain in detail the cause of this skin disease.<br><br>A3. The cause of the skin disease shown in the image is likely to be a combination of genetic and environmental factors. Psoriasis is a genetic autoimmune disorder that causes the rapid growth and accumulation of skin cells, resulting in the formation of red, scaly, and raised patches on the skin. Lichen planus is also an autoimmune disease that causes the immune system to attack	Q1. Could you describe the skin disease in this image for me?<br><br>A1. Psoriasis<br><br>Q2. You should write a paragraph to list more features you observed in the image.<br><br>A2. Red patches on the arm with silvery white scales<br><br>Q3. With previous information, please write a paragraph to explain in detail the cause of this skin disease.<br><br>A3. Psoriasis is an immune-mediated polygenic skin disorder. Various environmental triggering factors(e.g. trauma, infections or medications, may elicit disease in predisposed individuals). The characteristic lesion is a sharply demarcated erythematous plaque with micaceous scale, and the plaques may be localized or widespread in distribution. Psoriasis is a systemic disease process in which up to 20–30% of the patients have or will develop psoriatic arthritis. In addition, in patients with moderate to severe psoriasis, there is an increased relative risk for metabolic syndrome and atherosclerotic cardiovascular disease. The current treatments, although often effective, do not provide a satisfactory long-term solution.<br>Typical histologic findings include acanthosis with elongated rete ridges, hypogranulosis, hyper- and parakeratosis, dilated blood vessels and a

healthy cells in the skin, leading to the formation of rough, brownish or white bumps. Both psoriasis and lichen planus are believed to be triggered by environmental factors such as stress, injury, and infection, although the exact triggers are not yet fully understood. The pustules on the arms and legs may be caused by various factors such as acne, impetigo, or psoriasis, while the rash on the torso could be caused by an allergic reaction or eczema. It is important to consult a dermatologist for an accurate diagnosis and appropriate treatment.<br><br>Q4. How should I treat it? What medicine should I use?<br><br>A4. The treatment for the skin disease shown in the image will depend on the specific diagnosis and the severity of the symptoms. However, here are some general treatments that may be recommended for the different conditions shown in the image:<br>For psoriasis:<br>* Topical creams and ointments, such as corticosteroids, vitamin D analogs, and immunomodulatory agents, can be used to treat mild to moderate psoriasis.<br>* Phototherapy, which involves exposure to ultraviolet light, may be recommended for more severe cases.<br>* Systemic medications, such as biologics and immunosuppressants, may be prescribed for severe or widespread psoriasis that does not respond to topical treatments.<br>For lichen planus:<br>* Topical creams and ointments, such as corticosteroids, can be used to treat mild to moderate lichen planus.<br>* Phototherapy may be recommended for more severe cases.<br>* Systemic medications, such as retinoids and immunosuppressants, may be prescribed for severe or widespread lichen planus that does not respond to topical treatments.	perivascular infiltrate of lymphocytes with neutrophils singly or within aggregates in the epidermis. Phototherapy, methotrexate, cyclosporine and “biologic” therapies that target key immune effector cells and cytokines lead to significant clinical improvement.<br><br>Q4. How should I treat it? What medicine should I use?<br><br>A4. The type of treatment depends on the severity of the patient's condition as well as their willingness to undergo treatment and their financial situation. In this case, *topical Vitamin D3 analogues*, *Corticosteroids* and *Topical Retinoids* (*tazarotene*) are the first line medications. If the rash covers a large area, biologic drugs, such as *Adalimumab*, *Ustekinumab*, and *Secukinumab*, which target specific molecules in the immune system are more effective to reduce inflammation.
Evaluation of Physician	
Evaluation Item	Score (1-5, the higher the better)
SkinGPT-4’s diagnosis is correct or relevant.	3

SkinGPT-4's description is informative.	2
SkinGPT-4's suggestions are useful.	4
SkinGPT-4 can help doctors with diagnosis.	4
SkinGPT-4 can help patients to understand their disease better.	3
If SkinGPT-4 can be deployed locally, it protects patients' privacy.	5
Willingness to use SkinGPT-4	5

Case Psoriasis 9

<table>
<tr><td>Image ID: Psoriasis9</td><td>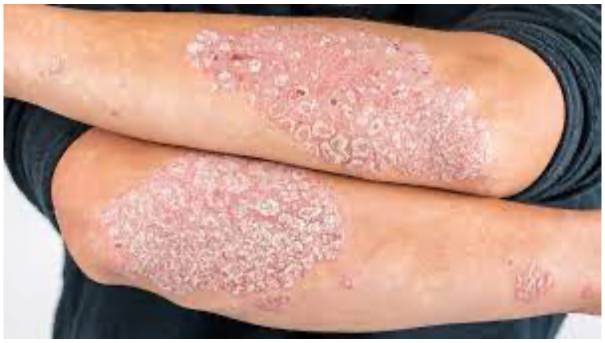</td></tr>
<tr><th>SkinGPT-4</th><th>Dermatology Physician</th></tr>
<tr><td>Q1. Could you describe the skin disease in this image for me?

A1. psoriasis

Q2. You should write a paragraph to list more features you observed in the image.

A2. The image shows a person with a rash on their arm. The rash appears to be red and inflamed, with small bumps and blisters visible on the skin. It looks painful and uncomfortable. The person in the image is holding their arm and looking at it, which suggests that they are aware of the rash and its symptoms. The image also shows a white background, which could indicate that it was taken in a medical setting. Overall, the image shows a case of psoriasis, a chronic autoimmune condition that causes skin redness and itching.

Q3. With previous information, please write a paragraph to explain in detail the cause of this skin disease.

A3. The cause of this skin disease is not specified in the image. However, it is likely to be a chronic autoimmune condition such as psoriasis, which is caused by an overactive immune system. In psoriasis, the immune system mistakenly attacks healthy cells in the skin, leading to inflammation and the formation of red, scaly patches. The exact trigger for psoriasis is not known, but it can be caused by a variety of factors, including stress, infections, and certain medications. In some cases, psoriasis may be associated with other health conditions, such as psoriatic arthritis and</td><td>Q1. Could you describe the skin disease in this image for me?

A1. Psoriasis

Q2. You should write a paragraph to list more features you observed in the image.

A2. Red patches on the elbow with thicken silvery white scales

Q3. With previous information, please write a paragraph to explain in detail the cause of this skin disease.

A3. Psoriasis is an immune-mediated polygenic skin disorder. Various environmental triggering factors(e.g. trauma, infections or medications, may elicit disease in predisposed individuals). The characteristic lesion is a sharply demarcated erythematous plaque with micaceous scale, and the plaques may be localized or widespread in distribution. Psoriasis is a systemic disease process in which up to 20–30% of the patients have or will develop psoriatic arthritis. In addition, in patients with moderate to severe psoriasis, there is an increased relative risk for metabolic syndrome and atherosclerotic cardiovascular disease. The current treatments, although often effective, do not provide a satisfactory long-term solution.
Typical histologic findings include acanthosis with elongated rete ridges, hypogranulosis, hyper- and parakeratosis, dilated blood vessels and a perivascular infiltrate of lymphocytes with neutrophils singly or within aggregates in the epidermis. Phototherapy, methotrexate,</td></tr>
</table>

an increased risk of cardiovascular disease. The treatment options for psoriasis include topical creams and ointments, phototherapy, and systemic medications. The image shows a person with a rash on their arm, which could be caused by psoriasis.<br><br>Q4. How should I treat it? What medicine should I use?<br><br>A4. Treatment for this skin disease depends on the specific condition that is causing the symptoms. In the case of psoriasis, the most common treatment options include topical creams and ointments, phototherapy, and systemic medications. Topical treatments, such as corticosteroids, coal tar, and vitamin D analogs, work by reducing inflammation and slowing the growth of skin cells. Phototherapy, which involves exposing the skin to ultraviolet light, can also be effective in reducing symptoms of psoriasis. Systemic medications, such as biologics, methotrexate, and cyclosporine, are taken orally or by injection and work by targeting specific components of the immune system. It is important to consult a dermatologist or other healthcare provider to determine the best treatment options for your specific condition. Over-the-counter creams and ointments may also be recommended to alleviate symptoms.	cyclosporine and “biologic” therapies that target key immune effector cells and cytokines lead to significant clinical improvement.<br><br>Q4. How should I treat it? What medicine should I use?<br><br>A4. The type of treatment depends on the severity of the patient's condition as well as their willingness to undergo treatment and their financial situation. In this case, *topical Vitamin D3 analogues*, *Corticosteroids* and *Topical Retinoids* (*tazarotene*) are the first line medications. If the rash covers a large area, biologic drugs, such as *Adalimumab*, *Ustekinumab*, and *Secukinumab*, which target specific molecules in the immune system are more effective to reduce inflammation.
Evaluation of Physician	
Evaluation Item	Score (1-5, the higher the better)
SkinGPT-4’s diagnosis is correct or relevant.	5
SkinGPT-4’s description is informative.	5
SkinGPT-4’s suggestions are useful.	5
SkinGPT-4 can help doctors with diagnosis.	5
SkinGPT-4 can help patients to understand their disease better.	5
If SkinGPT-4 can be deployed locally, it protects patients’ privacy.	5

Willingness to use SkinGPT-4	5

Case Psoriasis 10

Image ID: Psoriasis10	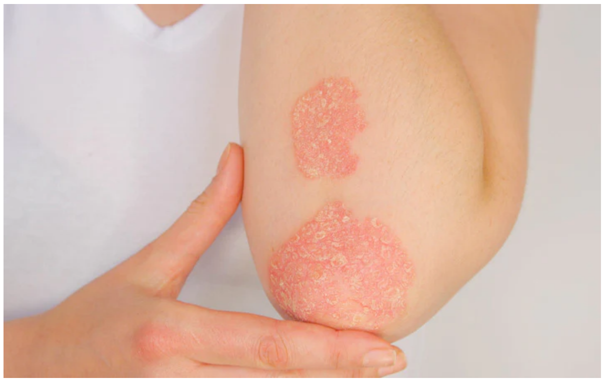
**SkinGPT-4**	**Dermatology Physician**
Q1. Could you describe the skin disease in this image for me?<br><br>A1. psoriasis and lichen planus<br><br>Q2. You should write a paragraph to list more features you observed in the image.<br><br>A2. The image shows various features of psoriasis, a chronic autoimmune skin condition. The most notable feature is the presence of thick, scaly, red plaques on the skin. The skin lesions are often found on the elbows, knees, and scalp, but can appear anywhere on the body. Another feature is the silvery white scales that flake off and accumulate on the skin, as well as the skin's inflammation and itching. In this image, the person is holding their arm and showing the red, scaly patches on their skin.<br><br>Q3. With previous information, please write a paragraph to explain in detail the cause of this skin disease.<br><br>A3. Psoriasis is an autoimmune skin condition caused by an overactive immune system. The immune system mistakenly attacks healthy cells in the skin, causing the rapid growth of skin cells. This leads to the formation of thick, scaly, red plaques on the skin's surface. The exact cause of psoriasis is not fully understood, but it is believed to be a combination of genetic and environmental factors. Triggers such as stress, infections, and certain medications can worsen symptoms, but the condition is not contagious. In some cases,	Q1. Could you describe the skin disease in this image for me?<br><br>A1. Psoriasis<br><br>Q2. You should write a paragraph to list more features you observed in the image.<br><br>A2. Red patches on the elbow with silvery scales.<br><br>Q3. With previous information, please write a paragraph to explain in detail the cause of this skin disease.<br><br>A3. Psoriasis is an immune-mediated polygenic skin disorder. Various environmental triggering factors(e.g. trauma, infections or medications, may elicit disease in predisposed individuals). The characteristic lesion is a sharply demarcated erythematous plaque with micaceous scale, and the plaques may be localized or widespread in distribution. Psoriasis is a systemic disease process in which up to 20–30% of the patients have or will develop psoriatic arthritis. In addition, in patients with moderate to severe psoriasis, there is an increased relative risk for metabolic syndrome and atherosclerotic cardiovascular disease. The current treatments, although often effective, do not provide a satisfactory long-term solution.<br>Typical histologic findings include acanthosis with elongated rete ridges, hypogranulosis, hyper- and parakeratosis, dilated blood vessels and a perivascular infiltrate of lymphocytes with neutrophils singly or within aggregates in the

psoriasis may be triggered by a specific event, such as a severe sunburn or strep throat infection. The condition often runs in families, and people with a family history of psoriasis are more likely to develop the condition.<br><br>Q4. How should I treat it? What medicine should I use?<br><br>A4. Treatment for psoriasis depends on the severity and type of the condition. Mild to moderate cases can be treated with topical creams and ointments, while severe cases may require systemic medications.<br>Topical treatments include:<br>* Corticosteroids to reduce inflammation and suppress the immune system<br>* Vitamin D analogues, such as calcipotriol, to slow down the growth of skin cells<br>* Moisturizers to keep the skin hydrated and prevent dryness<br>Systemic medications include:<br>* Biologic drugs, such as adalimumab and ustekinumab, which target specific proteins in the immune system<br>* Retinoids, such as acitretin and isotretinoin, which slow down the growth of skin cells<br>* Methotrexate, a chemotherapy drug used to treat cancer, psoriasis and rheumatoid arthritis.	epidermis. Phototherapy, methotrexate, cyclosporine and “biologic” therapies that target key immune effector cells and cytokines lead to significant clinical improvement.<br><br>Q4. How should I treat it? What medicine should I use?<br><br>A4. A4. The type of treatment depends on the severity of the patient's condition as well as their willingness to undergo treatment and their financial situation. In this case, *topical Vitamin D3 analogues*, *Corticosteroids* and *Topical retinoids* (*tazarotene*) are the first line medications. Furthermore, biologic drugs, such as *Adalimumab*, *Ustekinumab*, and *Secukinumab*, which target specific molecules in the immune system are more effective to reduce inflammation.
Evaluation of Physician	
Evaluation Item	Score (1-5, the higher the better)
SkinGPT-4’s diagnosis is correct or relevant.	5
SkinGPT-4’s description is informative.	5
SkinGPT-4’s suggestions are useful.	5
SkinGPT-4 can help doctors with diagnosis.	5
SkinGPT-4 can help patients to understand their disease better.	5
If SkinGPT-4 can be deployed locally, it protects patients’ privacy.	5

Willingness to use SkinGPT-4	5

Case Infestations and Bites 1

<table>
<tr><td>Image ID: Infestations and Bites1</td><td>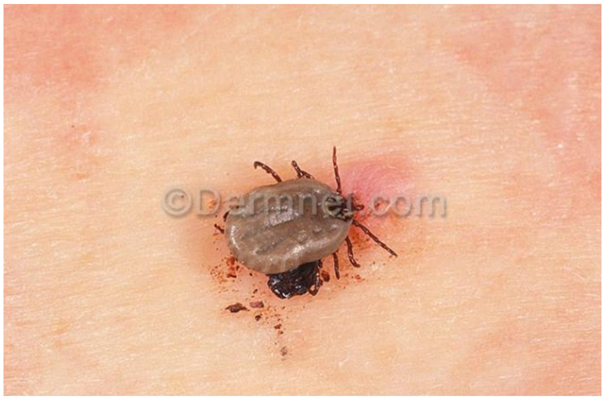
</td></tr>
<tr><td>SkinGPT-4</td><td>Dermatology Physician</td></tr>
<tr><td>Q1. Could you describe the skin disease in this image for me?

A1. This could be a skin disease image of tick bite.

Q2. You should write a paragraph to list more features you observed in the image.

A2. This could be a skin disease image of tick bite. The tick bite is a common tick-borne disease caused by the bite of a tick, which can lead to the transmission of diseases such as Lyme disease, Anaplasmosis, and Ehrlichiosis. The tick bite can present as a red, painless, flat or slightly elevated, small (rarely > 5 mm in diameter) lesion at the site of the bite. The tick bite is usually asymptomatic, but a local inflammatory reaction may occur at the site of the bite, with erythema, induration, or swelling. The tick tick bite can be diagnosed by clinical presentation, and confirmed by serologic testing or PCR. The tick bite can be treated with antibiotics, and the tick can be removed by using tweezers, applying ice or using tick removers.

Q3. With previous information, please write a paragraph to explain in detail the cause of this skin disease.

A3. The tick bite is caused by the bite of a tick, which can lead to the transmission of diseases such as Lyme disease, Anaplasmosis, and Ehrlichiosis. Ticks are small, blood-sucking parasites that attach themselves to animals, including humans, and feed on their blood. The</td><td>Q1. Could you describe the skin disease in this image for me?

A1. This could be a skin disease image of tick bite.

Q2. You should write a paragraph to list more features you observed in the image.

A2. The tick attaches itself to the skin by sticking its proboscis into the flesh to suck blood from the superficial vessels. The insertion of the hypostome is generally unnoticed by the subject. The attached tick may be mistaken by the patient for a new mole. The parasite slowly becomes engorged and then falls off. During this time, which may last for 7–12 days, the patient may have fever, chills, headache, abdominal pain, and vomiting (tick bite pyrexia). Removal of the engorged tick causes a subsidence of the general symptoms in 12–36 hours.

Q3. With previous information, please write a paragraph to explain in detail the cause of this skin disease.

A3. Correct identification of an attached tick provides the physician with an opportunity to discuss tick control measures and symptoms of tickborne disease. Most hard ticks have a three-host life cycle. Larval (seed) ticks have six legs and feed on small animals in nature . The nymphs and adults feed on larger animals. Nymphs resemble small adult ticks, with eight legs, but are sexually</td></tr>
</table>

tick bite can occur when a tick, which is infected with a tick-borne disease, bites a human. The tick bite can cause the transmission of the tick-borne disease, which can cause various symptoms and health problems, depending on the type of tick and the disease it carries. The tick bite can be prevented by using tick repellents, wearing protective clothing, and performing tick checks after being outdoors in tick-endemic areas. The tick bite can be diagnosed by clinical presentation, and confirmed by serologic testing or PCR. The tick bite can be treated with antibiotics, and the tick can be removed by using tweezers, applying ice or using tick removers. The tick-borne disease can be prevented by using tick repellents, wearing protective clothing, and performing tick checks after being outdoors in tick-endemic areas.<br><br>Q4. How should I treat it? What medicine should I use?<br><br>A4. The treatment of tick bite depends on the type of tick and the disease it carries. If the tick is infected with a tick-borne disease, the tick bite can be treated with antibiotics, such as doxycycline, amoxicillin, or ciprofloxacin. The antibiotics are used to prevent or treat the tick-borne disease, such as Lyme disease, Anaplasmosis, and Ehrlichiosis. The antibiotics can be prescribed by a healthcare provider, and the patient should follow the instructions for taking the medicine, such as the dosage and duration of the treatment. The healthcare provider may also recommend other treatment options, such as pain relievers, if needed.<br><br>It is important to note that tick-borne diseases can be prevented by using tick repellents, wearing protective clothing, and performing tick checks after being outdoors in tick-endemic areas.	immature. Because they attach for one long blood meal during each phase of the life cycle and search for a new host for each blood meal, ticks are efficient disease vectors. Hard ticks have a hard dorsal plate (scutum) and account for most medically important ticks, transmitting human diseases including Lyme borreliosis, ehrlichiosis, granulocytotropic anaplasmosis and a variety of rickettsial infections (including RockyMountain spotted fever; RMSF) . Soft ticks such as Ornithodoros (the vector of borrelial relapsing fever) lack a scutum and have retroverted mouthparts. These ticks tend to live in close association with a single host.<br><br>Q4. How should I treat it? What medicine should I use?<br><br>A4. Milder tick bite reactions may respond to application of a potent (class 1 or 2) topical corticosteroid; lesions may fail to improve unless the agent is occluded. More intense reactions often require intralesional corticosteroid injection, and some only respond to surgical excision. Often, the pruritus is so severe that patients express little concern about the discomfort and scar associated with the surgical procedure.Most tick-borne diseases respond readily to tetracyclines. Empiric treatment of suspected RMSF is always appropriate, and therapy should never be delayed until confirmatory tests are available. The consequences of delayed diagnosis and treatment of RMSF are evidenced by reports of deaths in children. Doxycycline is the drug of choice for children as well as adults with RMSF. Staining of teeth is unlikely with a short course of doxycycline, and chloramphenicol is less effective for RMSF.
Evaluation of Physician	
Evaluation Item	Score (1-5, the higher the better)
SkinGPT-4's diagnosis is correct or relevant.	5
SkinGPT-4's description is informative.	4

SkinGPT-4's suggestions are useful.	5
SkinGPT-4 can help doctors with diagnosis.	5
SkinGPT-4 can help patients to understand their disease better.	5
If SkinGPT-4 can be deployed locally, it protects patients' privacy.	5
Willingness to use SkinGPT-4	5

Case Infestations and Bites 2

<table>
<tr><td>Image ID: Infestations and Bites2</td><td>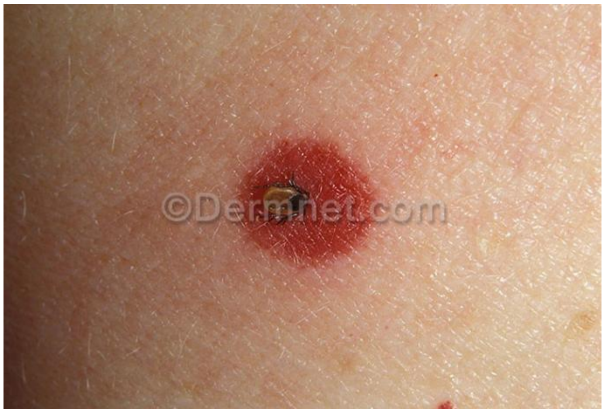
</td></tr>
<tr><th>SkinGPT-4</th><th>Dermatology Physician</th></tr>
<tr><td>Q1. Could you describe the skin disease in this image for me?

A1. This could be a skin disease image of tick bite.

Q2. You should write a paragraph to list more features you observed in the image.

A2. This image shows a skin lesion on the arm of a patient. The lesion is well demarcated, circular in shape, and has a central depression. The lesion is also erythematous and has a scaling papillomatous surface. The lesion is surrounded by a faintly inflamed and reactive border. There is no crusting or oozing observed. No pigment change or telangiectasia is seen in the lesion. The surface is smooth and shiny. The lesion is approximately 5 mm in diameter. This could be a skin cancer, a seborrheic keratosis, or a dermatofibroma.

Q3. The cause of this skin disease is unknown. It is a common, benign, circumscribed nodular lesion consisting of a central papule surrounded by a vascular halo. The lesion is also called “lentigo simplicity” or “benign lentigo.” It is a type of skin growth that is seen most commonly in elderly people, particularly those with fair skin. It is often found on the face, neck, and arms, but can occur anywhere on the body. The cause of lentigo simplicity is unknown, but it is thought to be related to aging and sun exposure. It is considered a type of age spot or solar lentigo. It is</td><td>Q1. Could you describe the skin disease in this image for me?

A1. This could be a skin disease image of tick bite.

Q2. You should write a paragraph to list more features you observed in the image.

A2. The tick attaches itself to the skin by sticking its proboscis into the flesh to suck blood from the superficial vessels. The insertion of the hypostome is generally unnoticed by the subject. The attached tick may be mistaken by the patient for a new mole. The parasite slowly becomes engorged and then falls off. During this time, which may last for 7–12 days, the patient may have fever, chills, headache, abdominal pain, and vomiting (tick bite pyrexia). Removal of the engorged tick causes a subsidence of the general symptoms in 12–36 hours.

Q3. With previous information, please write a paragraph to explain in detail the cause of this skin disease.

A3. Correct identification of an attached tick provides the physician with an opportunity to discuss tick control measures and symptoms of tickborne disease. Most hard ticks have a three-host life cycle. Larval (seed) ticks have six legs and feed on small animals in nature . The nymphs and adults feed on larger animals. Nymphs resemble small adult ticks, with eight legs, but are sexually</td></tr>
</table>

usually asymptomatic, but some people may experience itching or discomfort. Lentigo simplicity is usually not dangerous and does not require treatment. However, it can be removed if desired with various methods such as cryotherapy, curettage, or electrodessication.<br><br>Q4. How should I treat it? What medicine should I use?<br><br>A4. The treatment of lentigo simplicity (age spot) is usually not necessary, as it is considered a benign condition. However, if it is causing cosmetic concerns, there are several options for treatment, including:<br>* Cryotherapy: This involves freezing the lesion with liquid nitrogen, which causes it to blister and eventually fall off.<br>* Curettage: This involves scraping the lesion with a curette, which removes the lesion and surrounding tissue.<br>* Electrodessication: This involves using a needle to remove the lesion by causing an electric current to pass through it, which causes it to burn and eventually fall off.<br>If you want to remove it then you can use 10-25% salicylic acid or 10-20% glycolic acid or 20% Jessner's solution. These medications are applied topically to the lesion and they help to remove the lesion by exfoliating the skin. But, before using any of the above medication, you should consult a dermatologist.	immature. Because they attach for one long blood meal during each phase of the life cycle and search for a new host for each blood meal, ticks are efficient disease vectors. Hard ticks have a hard dorsal plate (scutum) and account for most medically important ticks, transmitting human diseases including Lyme borreliosis, ehrlichiosis, granulocytotropic anaplasmosis and a variety of rickettsial infections (including RockyMountain spotted fever; RMSF) . Soft ticks such as Ornithodoros (the vector of borrelial relapsing fever) lack a scutum and have retroverted mouthparts. These ticks tend to live in close association with a single host.<br><br>Q4. How should I treat it? What medicine should I use?<br><br>A4. Milder tick bite reactions may respond to application of a potent (class 1 or 2) topical corticosteroid; lesions may fail to improve unless the agent is occluded. More intense reactions often require intralesional corticosteroid injection, and some only respond to surgical excision. Often, the pruritus is so severe that patients express little concern about the discomfort and scar associated with the surgical procedure.Most tick-borne diseases respond readily to tetracyclines. Empiric treatment of suspected RMSF is always appropriate, and therapy should never be delayed until confirmatory tests are available. The consequences of delayed diagnosis and treatment of RMSF are evidenced by reports of deaths in children. Doxycycline is the drug of choice for children as well as adults with RMSF. Staining of teeth is unlikely with a short course of doxycycline, and chloramphenicol is less effective for RMSF.
Evaluation of Physician	
Evaluation Item	Score (1-5, the higher the better)
SkinGPT-4's diagnosis is correct or relevant.	5
SkinGPT-4's description is informative.	1
SkinGPT-4's suggestions are useful.	1

SkinGPT-4 can help doctors with diagnosis.	5
SkinGPT-4 can help patients to understand their disease better.	1
If SkinGPT-4 can be deployed locally, it protects patients' privacy.	1
Willingness to use SkinGPT-4	1

Case Infestations and Bites 3

Image ID: Infestations and Bites3	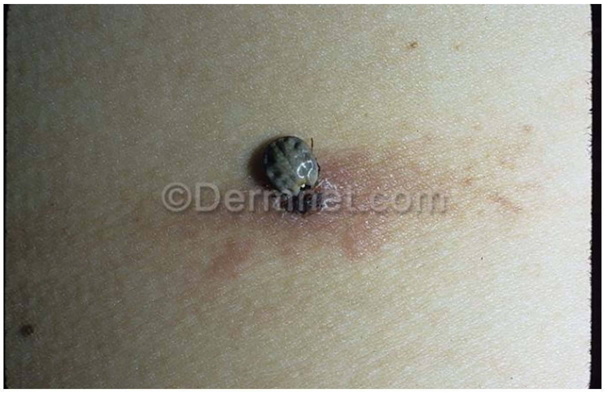
SkinGPT-4	Dermatology Physician
Q1. Could you describe the skin disease in this image for me?<br><br>A1. This could be a skin disease image of tick bite.<br><br>Q2. You should write a paragraph to list more features you observed in the image.<br><br>A2. This image shows a tick bite, which is a common occurrence during the summer months. The tick is attached to the skin and has a flat, oval shape with a dark body and light colored legs. Thetick is embedded in the skin, and the tick's head is embedded in the skin, while the body is visible. The tick is firmly attached to the skin and has a wide attachment site. The tick is still attached to the skin, and the tick's body is not swollen. No redness, pus, or bruising is seen around the attachment site. The tick is attached to the skin at the lower part of the back.<br><br>Q3. With previous information, please write a paragraph to explain in detail the cause of this skin disease.<br><br>A3. The cause of this skin disease is a tick bite. Ticks are small, bloodsucking parasites that attach themselves to animals, including humans, to feed. They are most commonly found in wooded areas, tall grass, and close to bushes. Ticks can carry various diseases, including Lyme disease, which is caused by the bacterium Borrelia burgdorferi. The tick bite leads to the transmission of the	Q1. Could you describe the skin disease in this image for me?<br><br>A1. This could be a skin disease image of tick bite.<br><br>Q2. You should write a paragraph to list more features you observed in the image.<br><br>A2. The tick attaches itself to the skin by sticking its proboscis into the flesh to suck blood from the superficial vessels. The insertion of the hypostome is generally unnoticed by the subject. The attached tick may be mistaken by the patient for a new mole. The parasite slowly becomes engorged and then falls off. During this time, which may last for 7–12 days, the patient may have fever, chills, headache, abdominal pain, and vomiting (tick bite pyrexia). Removal of the engorged tick causes a subsidence of the general symptoms in 12–36 hours.<br><br>Q3. With previous information, please write a paragraph to explain in detail the cause of this skin disease.<br><br>A3. Correct identification of an attached tick provides the physician with an opportunity to discuss tick control measures and symptoms of tickborne disease. Most hard ticks have a three-host life cycle. Larval (seed) ticks have six legs and feed on small animals in nature . The nymphs and adults feed on larger animals. Nymphs resemble small adult ticks, with eight legs, but are sexually

<table>
<tr><td>bacteria into the bloodstream, causing the body's immune system to respond to the infection. The symptoms of Lyme disease can vary from fever, headache, and fatigue to a skin rash called erythema migrans. If left untreated, the infection can spread to the joints, heart, and nervous system, leading to more severe symptoms. In this image, the tick bite has caused a skin lesion which is a sign of Lyme disease. The tick bite is a common occurrence during the summer months, and it is essential to check the body for ticks after being in tick-endemic areas.

Q4. How should I treat it? What medicine should I use?

A4. The treatment of tick bites and the associated diseases depends on the symptoms and the tick species. If you suspect a tick bite and develop fever, headache, fatigue, or a skin rash, it is essential to see a healthcare provider. Your healthcare provider can confirm the diagnosis and recommend appropriate treatment.

For tick bites that are infected with Lyme disease, antibiotics are the primary treatment. The most commonly used antibiotics are doxycycline, amoxicillin, and cefuroxime. These medications are effective in killing the bacteria that cause Lyme disease. It is important to complete the full course of antibiotics as prescribed by your healthcare provider to ensure that the infection is fully treated.
Over-the-counter topical tick repellents or insecticides can also be used to prevent tick bites. These products contain ingredients such as DEET, permethrin, or picaridin that repel or kill ticks. They should be applied to exposed skin and clothing before going into tick-endemic areas.
It is also essential to remove the tick as soon as possible by using tweezers or a tick removal tool. The tick should be pulled away from the skin with steady, upward pressure without crushing it.</td>
<td>immature. Because they attach for one long blood meal during each phase of the life cycle and search for a new host for each blood meal, ticks are efficient disease vectors. Hard ticks have a hard dorsal plate (scutum) and account for most medically important ticks, transmitting human diseases including Lyme borreliosis, ehrlichiosis, granulocytotropic anaplasmosis and a variety of rickettsial infections (including RockyMountain spotted fever; RMSF) . Soft ticks such as Ornithodoros (the vector of borrelial relapsing fever) lack a scutum and have retroverted mouthparts. These ticks tend to live in close association with a single host.

Q4. How should I treat it? What medicine should I use?

A4. Milder tick bite reactions may respond to application of a potent (class 1 or 2) topical corticosteroid; lesions may fail to improve unless the agent is occluded. More intense reactions often require intralesional corticosteroid injection, and some only respond to surgical excision. Often, the pruritus is so severe that patients express little concern about the discomfort and scar associated with the surgical procedure.Most tick-borne diseases respond readily to tetracyclines. Empiric treatment of suspected RMSF is always appropriate, and therapy should never be delayed until confirmatory tests are available. The consequences of delayed diagnosis and treatment of RMSF are evidenced by reports of deaths in children. Doxycycline is the drug of choice for children as well as adults with RMSF. Staining of teeth is unlikely with a short course of doxycycline, and chloramphenicol is less effective for RMSF.</td></tr>
<tr><td colspan="2">Evaluation of Physician</td></tr>
<tr><td>Evaluation Item</td><td>Score (1-5, the higher the better)</td></tr>
<tr><td>SkinGPT-4's diagnosis is correct or relevant.</td><td>5</td></tr>
</table>

SkinGPT-4's description is informative.	4
SkinGPT-4's suggestions are useful.	4
SkinGPT-4 can help doctors with diagnosis.	5
SkinGPT-4 can help patients to understand their disease better.	5
If SkinGPT-4 can be deployed locally, it protects patients' privacy.	5
Willingness to use SkinGPT-4	5

Case Infestations and Bites 4

<table>
<tr><td>Image ID: Infestations and Bites4</td><td>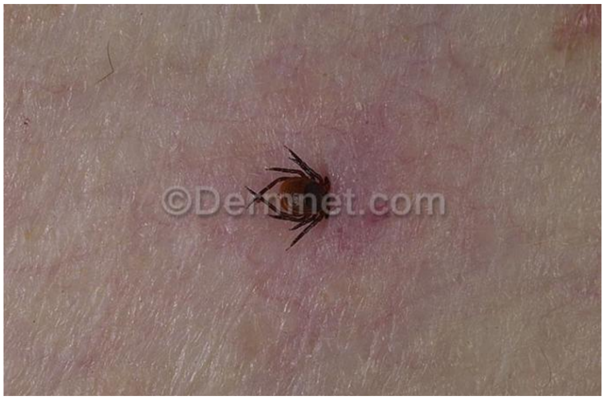
</td></tr>
<tr><th>SkinGPT-4</th><th>Dermatology Physician</th></tr>
<tr><td>Q1. Could you describe the skin disease in this image for me?

A1. This could be a skin disease image of tick bite.

Q2. You should write a paragraph to list more features you observed in the image.

A2. This image shows a tick, which is a small parasitic insect that feeds on the blood of humans and animals. The tick is placed on the skin, with its body attached to the skin and its legs spread out. The tick is a reddish brown color and has a round body with eight legs. This tick is a disease vector and can transmit tick borne diseases such as Lyme disease, Ehrlichiosis and Anaplasmosis.

Q3. With previous information, please write a paragraph to explain in detail the cause of this skin disease.

A3. The cause of this skin disease is an infestation of ticks. Ticks are small, external parasites that attach themselves to the skin of their host and feed on their blood. They are most commonly found in grassy or wooded areas and are most active during the spring and summer months. There are many species of ticks, but they all have the same life cycle and habits. Ticks can transmit diseases such as Lyme disease, Rocky Mountain spotted fever, ehrlichiosis, and anaplasmosis. The tick in the image is most likely to be a deer tick, which is the main tick that</td><td>Q1. Could you describe the skin disease in this image for me?

A1. This could be a skin disease image of tick bite.

Q2. You should write a paragraph to list more features you observed in the image.

A2. The tick attaches itself to the skin by sticking its proboscis into the flesh to suck blood from the superficial vessels. The insertion of the hypostome is generally unnoticed by the subject. The attached tick may be mistaken by the patient for a new mole. The parasite slowly becomes engorged and then falls off. During this time, which may last for 7–12 days, the patient may have fever, chills, headache, abdominal pain, and vomiting (tick bite pyrexia). Removal of the engorged tick causes a subsidence of the general symptoms in 12–36 hours.

Q3. With previous information, please write a paragraph to explain in detail the cause of this skin disease.

A3. Correct identification of an attached tick provides the physician with an opportunity to discuss tick control measures and symptoms of tickborne disease. Most hard ticks have a three-host life cycle. Larval (seed) ticks have six legs and feed on small animals in nature . The nymphs and adults feed on larger animals. Nymphs resemble small adult ticks, with eight legs, but are sexually immature. Because they attach for one long blood</td></tr>
</table>

transmits Lyme disease. It is important to remove a tick as soon as possible to prevent the transmission of any disease.<br><br>Q4. How should I treat it? What medicine should I use?<br><br>A4. The treatment of tick bites depends on the symptoms and the type of tick. If you have been bitten by a tick and develop a rash, fever, headache, or muscle or joint pain, you should see a doctor. Your doctor may prescribe antibiotics to prevent infection, such as doxycycline or amoxicillin. If you have been bitten by a tick and develop symptoms such as fever, headache, and fatigue, your doctor may prescribe antibiotics to treat the infection. If you have been bitten by a tick and develop symptoms such as fever, headache, and fatigue, your doctor may prescribe antibiotics to treat the infection. If you have been bitten by a tick and develop symptoms such as a rash, your doctor may prescribe a topical cream or ointment to treat the rash.	meal during each phase of the life cycle and search for a new host for each blood meal, ticks are efficient disease vectors. Hard ticks have a hard dorsal plate (scutum) and account for most medically important ticks, transmitting human diseases including Lyme borreliosis, ehrlichiosis, granulocytotropic anaplasmosis and a variety of rickettsial infections (including RockyMountain spotted fever; RMSF) . Soft ticks such as Ornithodoros (the vector of borrelial relapsing fever) lack a scutum and have retroverted mouthparts. These ticks tend to live in close association with a single host.<br><br>Q4. How should I treat it? What medicine should I use?<br><br>A4. Milder tick bite reactions may respond to application of a potent (class 1 or 2) topical corticosteroid; lesions may fail to improve unless the agent is occluded. More intense reactions often require intralesional corticosteroid injection, and some only respond to surgical excision. Often, the pruritus is so severe that patients express little concern about the discomfort and scar associated with the surgical procedure.Most tick-borne diseases respond readily to tetracyclines. Empiric treatment of suspected RMSF is always appropriate, and therapy should never be delayed until confirmatory tests are available. The consequences of delayed diagnosis and treatment of RMSF are evidenced by reports of deaths in children. Doxycycline is the drug of choice for children as well as adults with RMSF. Staining of teeth is unlikely with a short course of doxycycline, and chloramphenicol is less effective for RMSF.
Evaluation of Physician	
Evaluation Item	Score (1-5, the higher the better)
SkinGPT-4's diagnosis is correct or relevant.	5
SkinGPT-4's description is informative.	4
SkinGPT-4's suggestions are useful.	4
SkinGPT-4 can help doctors with diagnosis.	5

SkinGPT-4 can help patients to understand their disease better.	4
If SkinGPT-4 can be deployed locally, it protects patients' privacy.	5
Willingness to use SkinGPT-4	5

Case Infestations and Bites 5

Image ID: Infestations and Bites5	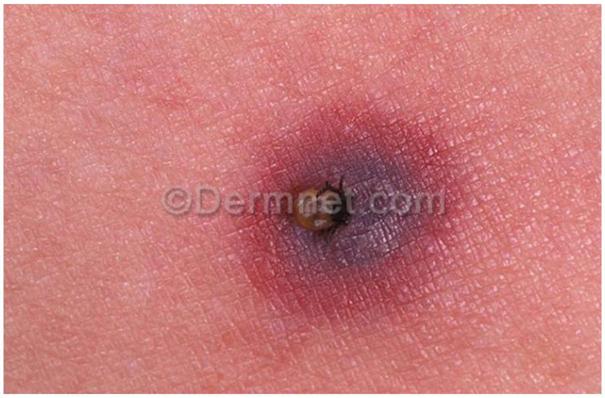
SkinGPT-4	Dermatology Physician
Q1. Could you describe the skin disease in this image for me?<br><br>A1. This could be a skin disease image of epidermal cyst.<br><br>Q2. You should write a paragraph to list more features you observed in the image.<br><br>A2. The image shows a pinkish-red, shiny, smooth, flat, round or oval plaque with a central depression on a smooth skin area. The surrounding skin appears normal. The lesion has a clean surgical margin. It is not flaccid. The lesion is on the right upper arm. No skin tags are seen. The skin is healthy. The patient is a 66 year old woman. The skin lesion is a skin tags.<br><br>Q3. With previous information, please write a paragraph to explain in detail the cause of this skin disease.<br><br>A3. The cause of this skin disease is not known. It could be a congenital anomaly or a benign proliferation of epidermal cells. The age of the patient and the location of the lesion suggest that it may be a skin tag. Skin tags are common, benign skin growths that are usually found on the neck, armpits, and groin. They are typically smooth, rounded, and small, and are often found on skin that is frequently rubbed or chafed. Skin tags are thought to be caused by friction or skin rubbing against skin tags, although the exact cause is not known. They are more common in people who are overweight, have diabetes, or	Q1. Could you describe the skin disease in this image for me?<br><br>A1. This could be a skin disease image of tick bite.<br><br>Q2. You should write a paragraph to list more features you observed in the image.<br><br>A2. The tick attaches itself to the skin by sticking its proboscis into the flesh to suck blood from the superficial vessels. The insertion of the hypostome is generally unnoticed by the subject. The attached tick may be mistaken by the patient for a new mole. The parasite slowly becomes engorged and then falls off. During this time, which may last for 7–12 days, the patient may have fever, chills, headache, abdominal pain, and vomiting (tick bite pyrexia). Removal of the engorged tick causes a subsidence of the general symptoms in 12–36 hours.<br><br>Q3. With previous information, please write a paragraph to explain in detail the cause of this skin disease.<br><br>A3. Correct identification of an attached tick provides the physician with an opportunity to discuss tick control measures and symptoms of tickborne disease. Most hard ticks have a three-host life cycle. Larval (seed) ticks have six legs and feed on small animals in nature . The nymphs and adults feed on larger animals. Nymphs resemble small adult ticks, with eight legs, but are sexually immature. Because they attach for one

have high cholesterol levels. They are also more common in people who have a lot of skin tags, a condition called acrochordons.<br><br>Q4. How should I treat it? What medicine should I use?<br><br>A4. The treatment of skin tags is usually not necessary, as they are usually harmless and do not cause any symptoms. However, if the skin tag is causing discomfort or irritation, it can be removed by a dermatologist or a doctor. The most common method of removal is by cutting or burning the tag off. There is no medication or cream that can remove a skin tag. If the skin tag is causing problems such as bleeding, pain or itching, a doctor should be consulted. The doctor may also recommend removing the skin tag if it is changing in size, shape, or color. If you have many skin tags, it is important to see a doctor as it could be a sign of an underlying medical condition.	long blood meal during each phase of the life cycle and search for a new host for each blood meal, ticks are efficient disease vectors. Hard ticks have a hard dorsal plate (scutum) and account for most medically important ticks, transmitting human diseases including Lyme borreliosis, ehrlichiosis, granulocytotropic anaplasmosis and a variety of rickettsial infections (including RockyMountain spotted fever; RMSF) . Soft ticks such as Ornithodoros (the vector of borrelial relapsing fever) lack a scutum and have retroverted mouthparts. These ticks tend to live in close association with a single host.<br><br>Q4. How should I treat it? What medicine should I use?<br><br>A4. Milder tick bite reactions may respond to application of a potent (class 1 or 2) topical corticosteroid; lesions may fail to improve unless the agent is occluded. More intense reactions often require intralesional corticosteroid injection, and some only respond to surgical excision. Often, the pruritus is so severe that patients express little concern about the discomfort and scar associated with the surgical procedure.Most tick-borne diseases respond readily to tetracyclines. Empiric treatment of suspected RMSF is always appropriate, and therapy should never be delayed until confirmatory tests are available. The consequences of delayed diagnosis and treatment of RMSF are evidenced by reports of deaths in children. Doxycycline is the drug of choice for children as well as adults with RMSF. Staining of teeth is unlikely with a short course of doxycycline, and chloramphenicol is less effective for RMSF.
Evaluation of Physician	
Evaluation Item	Score (1-5, the higher the better)
SkinGPT-4's diagnosis is correct or relevant.	1
SkinGPT-4's description is informative.	1
SkinGPT-4's suggestions are useful.	1

SkinGPT-4 can help doctors with diagnosis.	1
SkinGPT-4 can help patients to understand their disease better.	1
If SkinGPT-4 can be deployed locally, it protects patients' privacy.	1
Willingness to use SkinGPT-4	1

Case Infestations and Bites 6

<table>
<tr><td>Image ID: Infestations and Bites6</td><td>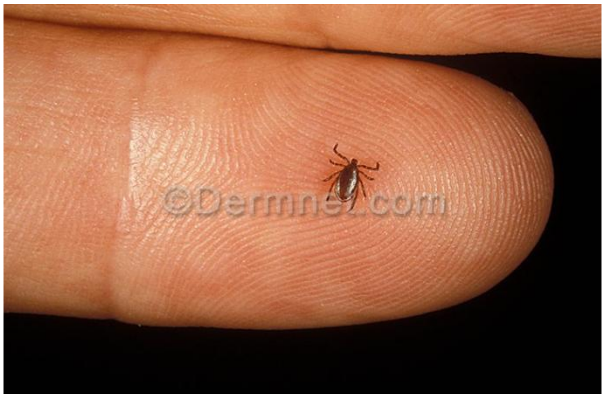
</td></tr>
<tr><th>SkinGPT-4</th><th>Dermatology Physician</th></tr>
<tr><td>Q1. Could you describe the skin disease in this image for me?

A1. This could be a skin disease image of tick bite.

Q2. You should write a paragraph to list more features you observed in the image.

A2. The tick bite image shows a tick tick bite, a tick is a small arachnid that attaches itself to the skin and feeds on blood. The tick is visible on the skin, it has a white back and a black body, it is also engorged. The tick bite is round in shape and has a reddish color.

Q3. With previous information, please write a paragraph to explain in detail the cause of this skin disease.

A3. The tick bite is caused by a tick, which is a small arachnid that attaches itself to the skin and feeds on blood. The tick has a white back and a black body and is visible on the skin. It is also engorged, which means it is swollen from the blood it has consumed. The tick bite is round in shape and has a reddish color, which is a sign of inflammation. The tick bite can cause a variety of symptoms, such as itching, pain, and swelling. It can also transmit diseases, such as Lyme disease and tick-borne meningoencephalitis. It is important to remove the tick as soon as possible to prevent further symptoms and complications.

Q4. How should I treat it? What medicine should I</td><td>Q1. Could you describe the skin disease in this image for me?

A1. This could be a skin disease image of tick bite.

Q2. You should write a paragraph to list more features you observed in the image.

A2. The tick attaches itself to the skin by sticking its proboscis into the flesh to suck blood from the superficial vessels. The insertion of the hypostome is generally unnoticed by the subject. The attached tick may be mistaken by the patient for a new mole. The parasite slowly becomes engorged and then falls off. During this time, which may last for 7–12 days, the patient may have fever, chills, headache, abdominal pain, and vomiting (tick bite pyrexia). Removal of the engorged tick causes a subsidence of the general symptoms in 12–36 hours.

Q3. With previous information, please write a paragraph to explain in detail the cause of this skin disease.

A3. Correct identification of an attached tick provides the physician with an opportunity to discuss tick control measures and symptoms of tickborne disease. Most hard ticks have a three-host life cycle. Larval (seed) ticks have six legs and feed on small animals in nature . The nymphs and adults feed on larger animals. Nymphs resemble small adult ticks, with eight legs, but are</td></tr>
</table>

use?<br><br>A4. The treatment for a tick bite depends on the severity of the symptoms and the tick-borne disease that the tick is carrying. Generally, the tick should be removed as soon as possible with tweezers or a tick remover, being careful not to leave the head or body of the tick in the skin. Antibiotics may be prescribed to prevent or treat tick-borne diseases. Over-the-counter antihistamines and calamine lotion can help relieve itching and reduce inflammation. If the tick was engorged, the person may need to be treated with antibiotics and the tick removed by a healthcare professional. If the person has a history of allergic reactions to tick bites, they should see a healthcare professional immediately.	sexually immature. Because they attach for one long blood meal during each phase of the life cycle and search for a new host for each blood meal, ticks are efficient disease vectors. Hard ticks have a hard dorsal plate (scutum) and account for most medically important ticks, transmitting human diseases including Lyme borreliosis, ehrlichiosis, granulocytotropic anaplasmosis and a variety of rickettsial infections (including RockyMountain spotted fever; RMSF) . Soft ticks such as Ornithodoros (the vector of borrelial relapsing fever) lack a scutum and have retroverted mouthparts. These ticks tend to live in close association with a single host.<br><br>Q4. How should I treat it? What medicine should I use?<br><br>A4. Milder tick bite reactions may respond to application of a potent (class 1 or 2) topical corticosteroid; lesions may fail to improve unless the agent is occluded. More intense reactions often require intralesional corticosteroid injection, and some only respond to surgical excision. Often, the pruritus is so severe that patients express little concern about the discomfort and scar associated with the surgical procedure.Most tick-borne diseases respond readily to tetracyclines. Empiric treatment of suspected RMSF is always appropriate, and therapy should never be delayed until confirmatory tests are available. The consequences of delayed diagnosis and treatment of RMSF are evidenced by reports of deaths in children. Doxycycline is the drug of choice for children as well as adults with RMSF. Staining of teeth is unlikely with a short course of doxycycline, and chloramphenicol is less effective for RMSF.

Evaluation of Physician	
Evaluation Item	Score (1-5, the higher the better)
SkinGPT-4’s diagnosis is correct or relevant.	5
SkinGPT-4’s description is informative.	5
SkinGPT-4’s suggestions are useful.	4

SkinGPT-4 can help doctors with diagnosis.	5
SkinGPT-4 can help patients to understand their disease better.	4
If SkinGPT-4 can be deployed locally, it protects patients' privacy.	5
Willingness to use SkinGPT-4	5

Case Infestations and Bites 7

Image ID: Infestations and Bites7	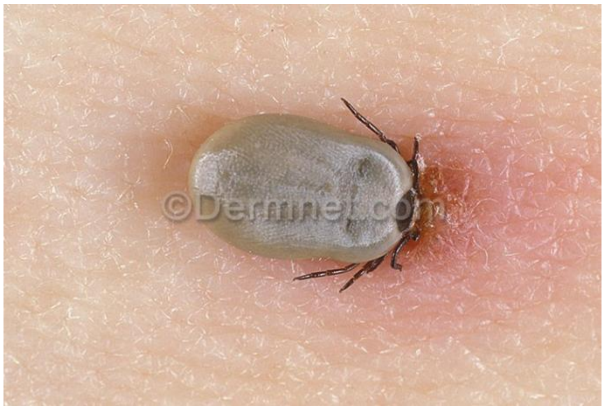
SkinGPT-4	Dermatology Physician
Q1. Could you describe the skin disease in this image for me?<br><br>A1. This could be a skin disease image of tick bite.<br><br>Q2. You should write a paragraph to list more features you observed in the image.<br><br>A2. This image shows a typical appearance of tick bite, which is caused by the bite of an tick. The tick bite is a flat, round, or oval lesion with a pale center and a darker margin. The tick bite is usually less than 1 cm in diameter and may have a white, wavy pattern in the center. The tick bite is usually asymptomatic and does not require any treatment. However, the tick may carry and transmit diseases, such as Lyme disease or tick borne illness. Therefore, it is important to monitor the tick bite and seek medical attention if any symptoms, such as fever, headache, or skin rash, develop.<br><br>Q3. With previous information, please write a paragraph to explain in detail the cause of this skin disease.<br><br>A3. The cause of this skin disease is the bite of a tick. Ticks are small, bloodsucking parasites that attach themselves to animals, including humans, and feed on their blood. There are many different species of ticks, and they can be found in a variety of environments, including forests, grasslands, and urban areas. Ticks can transmit diseases, such as Lyme disease, Ehrlichiosis, and Anaplasmosis, to humans through their bite.	Q1. Could you describe the skin disease in this image for me?<br><br>A1. This could be a skin disease image of tick bite.<br><br>Q2. You should write a paragraph to list more features you observed in the image.<br><br>A2. The tick attaches itself to the skin by sticking its proboscis into the flesh to suck blood from the superficial vessels. The insertion of the hypostome is generally unnoticed by the subject. The attached tick may be mistaken by the patient for a new mole. The parasite slowly becomes engorged and then falls off. During this time, which may last for 7–12 days, the patient may have fever, chills, headache, abdominal pain, and vomiting (tick bite pyrexia). Removal of the engorged tick causes a subsidence of the general symptoms in 12–36 hours.<br><br>Q3. With previous information, please write a paragraph to explain in detail the cause of this skin disease.<br><br>A3. Correct identification of an attached tick provides the physician with an opportunity to discuss tick control measures and symptoms of tickborne disease. Most hard ticks have a three-host life cycle. Larval (seed) ticks have six legs and feed on small animals in nature . The nymphs and adults feed on larger animals. Nymphs resemble small adult ticks, with eight legs, but are

These diseases can cause a variety of symptoms, such as fever, headache, and skin rash. The tick bite is usually asymptomatic and does not require any treatment. However, it is important to monitor the tick bite and seek medical attention if any symptoms develop. The tick can be removed by using tweezers, applying alcohol or a tick remover, or by covering the tick with petroleum jelly or nail polish to kill it. After the tick is removed, the bite should be cleaned with soap and water.<br><br>Q4. How should I treat it? What medicine should I use?<br><br>A4. The tick bite is usually asymptomatic and does not require any treatment. However, if you experience any symptoms, such as fever, headache, or skin rash, it is important to seek medical attention. Your doctor may prescribe antibiotics to prevent or treat tick-borne diseases, such as Lyme disease or Ehrlichiosis. The type and duration of antibiotic treatment will depend on the specific disease and its severity. In addition, your doctor may recommend over-the-counter pain relievers, such as acetaminophen or ibuprofen, to help relieve symptoms. It is important to follow your doctor's instructions and complete the full course of antibiotics, even if you start to feel better. You should also monitor the bite and watch for any signs of infection, such as redness, swelling, or pus. If you experience any of these symptoms, you should seek medical attention immediately.	sexually immature. Because they attach for one long blood meal during each phase of the life cycle and search for a new host for each blood meal, ticks are efficient disease vectors. Hard ticks have a hard dorsal plate (scutum) and account for most medically important ticks, transmitting human diseases including Lyme borreliosis, ehrlichiosis, granulocytotropic anaplasmosis and a variety of rickettsial infections (including RockyMountain spotted fever; RMSF) . Soft ticks such as Ornithodoros (the vector of borrelial relapsing fever) lack a scutum and have retroverted mouthparts. These ticks tend to live in close association with a single host.<br><br>Q4. How should I treat it? What medicine should I use?<br><br>A4. Milder tick bite reactions may respond to application of a potent (class 1 or 2) topical corticosteroid; lesions may fail to improve unless the agent is occluded. More intense reactions often require intralesional corticosteroid injection, and some only respond to surgical excision. Often, the pruritus is so severe that patients express little concern about the discomfort and scar associated with the surgical procedure.Most tick-borne diseases respond readily to tetracyclines. Empiric treatment of suspected RMSF is always appropriate, and therapy should never be delayed until confirmatory tests are available. The consequences of delayed diagnosis and treatment of RMSF are evidenced by reports of deaths in children. Doxycycline is the drug of choice for children as well as adults with RMSF. Staining of teeth is unlikely with a short course of doxycycline, and chloramphenicol is less effective for RMSF.
Evaluation of Physician	
Evaluation Item	Score (1-5, the higher the better)
SkinGPT-4's diagnosis is correct or relevant.	5
SkinGPT-4's description is informative.	5
SkinGPT-4's suggestions are useful.	4

SkinGPT-4 can help doctors with diagnosis.	5
SkinGPT-4 can help patients to understand their disease better.	4
If SkinGPT-4 can be deployed locally, it protects patients' privacy.	5
Willingness to use SkinGPT-4	5

Case Infestations and Bites 8

Image ID: Infestations and Bites8	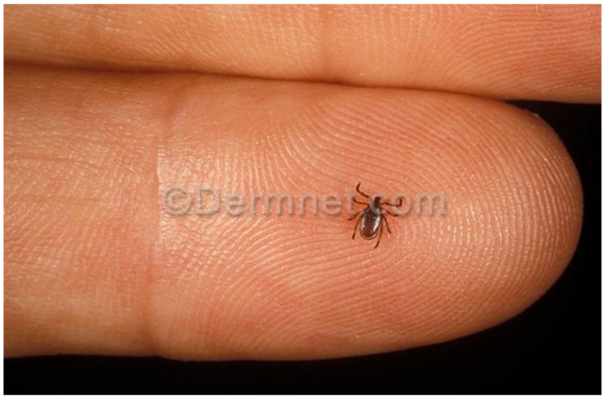
**SkinGPT-4**	**Dermatology Physician**
Q1. Could you describe the skin disease in this image for me?<br><br>A1. This could be a skin disease image of tick bite.<br><br>Q2. You should write a paragraph to list more features you observed in the image.<br><br>A2. The tick bite image depicts a tick bite disease, which is caused by the tick's bite. The tick attaches itself to the skin and injects its saliva into the skin, which can cause an allergic reaction and transmit diseases. The tick bite can cause a wheal and flare reaction, which appears as a red and swollen area around the bite. The tick bite can also cause a dark center or black dot in the center, which is a sign of bleeding. The tick bite can also cause a blister, which is a clear fluid filled bubble that appears on the skin. The tick bite can also cause a rash, which is a skin condition characterized by redness and irritation of the skin. The tick bite can also cause a tick bite fever, which is a type of tick borne disease that causes fever, headache, and muscle aches. The tick bite can also cause a tick disease, which is a condition caused by the tick's saliva and can cause an allergic reaction, fever, and fatigue. The tick bite can also cause a tick borne illness, which is a condition caused by tick bites and can cause fever, headache, and muscle aches. The tick bite can also cause a tick fever, which is a type of tick borne disease.<br><br>Q3. With previous information, please write a paragraph to explain in detail the cause of this skin	Q1. Could you describe the skin disease in this image for me?<br><br>A1. This could be a skin disease image of tick bite.<br><br>Q2. You should write a paragraph to list more features you observed in the image.<br><br>A2. The tick attaches itself to the skin by sticking its proboscis into the flesh to suck blood from the superficial vessels. The insertion of the hypostome is generally unnoticed by the subject. The attached tick may be mistaken by the patient for a new mole. The parasite slowly becomes engorged and then falls off. During this time, which may last for 7–12 days, the patient may have fever, chills, headache, abdominal pain, and vomiting (tick bite pyrexia). Removal of the engorged tick causes a subsidence of the general symptoms in 12–36 hours.<br><br>Q3. With previous information, please write a paragraph to explain in detail the cause of this skin disease.<br><br>A3. Correct identification of an attached tick provides the physician with an opportunity to discuss tick control measures and symptoms of tickborne disease. Most hard ticks have a three-host life cycle. Larval (seed) ticks have six legs and feed on small animals in nature . The nymphs and adults feed on larger animals. Nymphs resemble small adult ticks, with eight legs, but are sexually immature. Because they attach for one long blood meal during each phase of the life

disease.<br><br>A3. The cause of this skin disease is a tick bite. Ticks are small, blood-sucking parasites that attach themselves to the skin and feed on blood. When a tick bites, it injects saliva into the skin, which can cause an allergic reaction, inflammation, and other reactions. The tick can also carry diseases, such as Lyme disease, which can be transmitted through its bite. The tick in this image appears to be engorged, meaning it has fed on blood, and is attached to the skin. The redness and inflammation around the bite is a sign of an allergic reaction to the tick's saliva.<br><br>Q4. How should I treat it? What medicine should I use?<br><br>A4. The treatment for tick bites depends on the severity of the reaction and any underlying tick-borne diseases. Mild reactions can be treated with over-the-counter antihistamines and hydrocortisone cream to reduce itching and inflammation. More severe reactions, such as a large local reaction or anaphylaxis, may require prescription medications, including oral antihistamines, corticosteroids, and antibiotics for tick-borne diseases. In cases of Lyme disease, antibiotics such as doxycycline or amoxicillin may be prescribed. It is important to seek medical advice for proper diagnosis and treatment.	cycle and search for a new host for each blood meal, ticks are efficient disease vectors. Hard ticks have a hard dorsal plate (scutum) and account for most medically important ticks, transmitting human diseases including Lyme borreliosis, ehrlichiosis, granulocytotropic anaplasmosis and a variety of rickettsial infections (including RockyMountain spotted fever; RMSF) . Soft ticks such as Ornithodoros (the vector of borrelial relapsing fever) lack a scutum and have retroverted mouthparts. These ticks tend to live in close association with a single host.<br><br>Q4. How should I treat it? What medicine should I use?<br><br>A4. Milder tick bite reactions may respond to application of a potent (class 1 or 2) topical corticosteroid; lesions may fail to improve unless the agent is occluded. More intense reactions often require intralesional corticosteroid injection, and some only respond to surgical excision. Often, the pruritus is so severe that patients express little concern about the discomfort and scar associated with the surgical procedure.Most tick-borne diseases respond readily to tetracyclines. Empiric treatment of suspected RMSF is always appropriate, and therapy should never be delayed until confirmatory tests are available. The consequences of delayed diagnosis and treatment of RMSF are evidenced by reports of deaths in children. Doxycycline is the drug of choice for children as well as adults with RMSF. Staining of teeth is unlikely with a short course of doxycycline, and chloramphenicol is less effective for RMSF.
Evaluation of Physician	
Evaluation Item	Score (1-5, the higher the better)
SkinGPT-4's diagnosis is correct or relevant.	5
SkinGPT-4's description is informative.	4
SkinGPT-4's suggestions are useful.	4
SkinGPT-4 can help doctors with diagnosis.	5

SkinGPT-4 can help patients to understand their disease better.	4
If SkinGPT-4 can be deployed locally, it protects patients' privacy.	5
Willingness to use SkinGPT-4	5

Case Infestations and Bites 9

Image ID: Infestations and Bites9	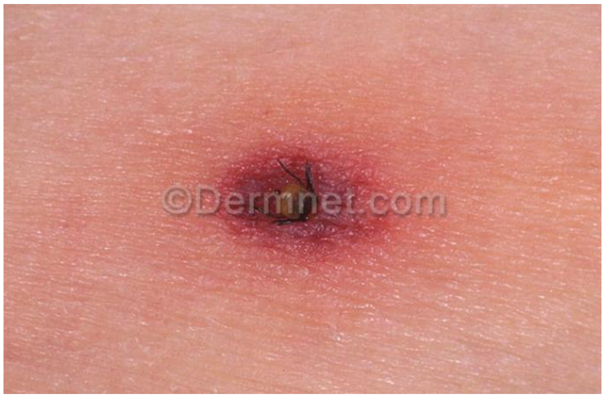
SkinGPT-4	Dermatology Physician
Q1. Could you describe the skin disease in this image for me?<br><br>A1. This could be a skin disease image of tick bite.<br><br>Q2. You should write a paragraph to list more features you observed in the image.<br><br>A2. The image shows a tick bite on the skin. The tick is attached to the skin and has a dark body with a white dot on its back. The bite is red and swollen, with surrounding erythema and no pustules or papules. The tick is crawling on the skin and is attached at the edge of the bite. The tick is embedded in the skin, with the legs embedded in the skin and the body in the dermis. The tick is pointed, with a pointed tip and a broad base. The tick is a tick bite a tick bite. The tick is attached to the skin and has a dark body with a white dot on its back. The tick is crawling on the skin and is attached at the edge of the bite. The tick is embedded in the skin, with the legs embedded in the skin and the body in the dermis. The tick is pointed, with a pointed tip and a broad base.<br><br>Q3. With previous information, please write a paragraph to explain in detail the cause of this skin disease.<br><br>A3. The cause of this skin disease is an insect bite from a tick. The tick is a small, eight-legged parasite that feeds on the blood of animals, including humans. Ticks are most commonly found in wooded areas, tall grass, and leaf litter.	Q1. Could you describe the skin disease in this image for me?<br><br>A1. his could be a skin disease image of tick bite.<br><br>Q2. You should write a paragraph to list more features you observed in the image.<br><br>A2. The tick attaches itself to the skin by sticking its proboscis into the flesh to suck blood from the superficial vessels. The insertion of the hypostome is generally unnoticed by the subject. The attached tick may be mistaken by the patient for a new mole. The parasite slowly becomes engorged and then falls off. During this time, which may last for 7–12 days, the patient may have fever, chills, headache, abdominal pain, and vomiting (tick bite pyrexia). Removal of the engorged tick causes a subsidence of the general symptoms in 12–36 hours.<br><br>Q3. With previous information, please write a paragraph to explain in detail the cause of this skin disease.<br><br>A3. Correct identification of an attached tick provides the physician with an opportunity to discuss tick control measures and symptoms of tickborne disease. Most hard ticks have a three-host life cycle. Larval (seed) ticks have six legs and feed on small animals in nature . The nymphs and adults feed on larger animals. Nymphs resemble small adult ticks, with eight legs, but are sexually immature. Because they attach for one long blood meal during each phase of the life cycle and search

They attach themselves to the skin and feed on blood for several days before dropping off. The tick bite can cause a variety of skin reactions, including redness, swelling, itching, and a localized rash. In some cases, a tick can carry and transmit diseases, such as Lyme disease, to humans through its bite. The tick in this image is likely a deer tick, which is the primary vector for Lyme disease. To prevent tick bites, it is important to wear protective clothing and use tick repellents when spending time in tick-endemic areas. Additionally, it is important to perform daily tick checks and remove any attached ticks as soon as possible to reduce the risk of tick-borne diseases.<br><br>Q4. How should I treat it? What medicine should I use?<br><br>A4. The treatment of tick bites depends on the severity of the reaction and the presence of any tick-borne diseases. Generally, tick bites are treated symptomatically with over-the-counter antihistamines and corticosteroids to reduce itching, swelling, and inflammation. In cases of Lyme disease, antibiotics are prescribed to prevent the progression of the disease.<br>For the itch and rash, calamine lotion, hydrocortisone cream, or antihistamines such as Benadryl can be used.<br>For the bite itself, it can be washed with soap and water and then applied with an antibiotic ointment such as Neosporin.<br><br>It is also important to monitor the bite for any signs of tick-borne illnesses such as Lyme disease, which may cause a fever, headache, and fatigue. If symptoms persist or worsen, medical attention should be sought.	for a new host for each blood meal, ticks are efficient disease vectors. Hard ticks have a hard dorsal plate (scutum) and account for most medically important ticks, transmitting human diseases including Lyme borreliosis, ehrlichiosis, granulocytotropic anaplasmosis and a variety of rickettsial infections (including RockyMountain spotted fever; RMSF) . Soft ticks such as Ornithodoros (the vector of borrelial relapsing fever) lack a scutum and have retroverted mouthparts. These ticks tend to live in close association with a single host.<br><br>Q4. How should I treat it? What medicine should I use?<br><br>A4. Milder tick bite reactions may respond to application of a potent (class 1 or 2) topical corticosteroid; lesions may fail to improve unless the agent is occluded. More intense reactions often require intralesional corticosteroid injection, and some only respond to surgical excision. Often, the pruritus is so severe that patients express little concern about the discomfort and scar associated with the surgical procedure.Most tick-borne diseases respond readily to tetracyclines. Empiric treatment of suspected RMSF is always appropriate, and therapy should never be delayed until confirmatory tests are available. The consequences of delayed diagnosis and treatment of RMSF are evidenced by reports of deaths in children. Doxycycline is the drug of choice for children as well as adults with RMSF. Staining of teeth is unlikely with a short course of doxycycline, and chloramphenicol is less effective for RMSF.

Evaluation of Physician

Evaluation Item	Score (1-5, the higher the better)
SkinGPT-4’s diagnosis is correct or relevant.	5
SkinGPT-4’s description is informative.	4
SkinGPT-4’s suggestions are useful.	5

SkinGPT-4 can help doctors with diagnosis.	5
SkinGPT-4 can help patients to understand their disease better.	4
If SkinGPT-4 can be deployed locally, it protects patients' privacy.	5
Willingness to use SkinGPT-4	5

Case Infestations and Bites 10

Image ID: Infestations and Bites10	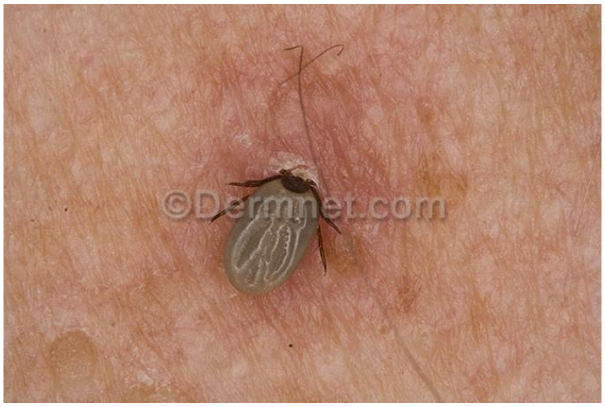
SkinGPT-4	Dermatology Physician
Q1. Could you describe the skin disease in this image for me?<br><br>A1. This could be a skin disease image of tick bite.<br><br>Q2. You should write a paragraph to list more features you observed in the image.<br><br>A2. The tick bite image shows a red patch with central white scar. The tick bite is located on the scalp. The tick bite is circular in shape with a central white scar in the middle. The tick bite is reddish brown in color with visible white scarring. The tick bite is a skin lesion caused by a tick bite.<br><br>Q3. With previous information, please write a paragraph to explain in detail the cause of this skin disease.<br><br>A3. The cause of tick bite is from the bite of an tick, which is a small parasitic arachnid that feeds on the blood of mammals, birds, and sometimes reptiles. Ticks are typically found in grassy areas, bushes, and woodlands, and they attach themselves to the skin of their host while feeding. The tick bite can cause a tick-borne disease such as Lyme disease, tick-borne fever, or tick-borne relapsing fever. The tick bite results in a red patch with central white scar as seen in the image. The tick bite is circular in shape with a central white scar in the middle. The tick bite is reddish brown in color with visible white scarring. The tick bite is a skin lesion caused by a tick bite and can be a sign of a tick-borne disease. It is important to seek medical attention if a tick bite is suspected,	Q1. Could you describe the skin disease in this image for me?<br><br>A1. This could be a skin disease image of tick bite.<br><br>Q2. You should write a paragraph to list more features you observed in the image.<br><br>A2. The tick attaches itself to the skin by sticking its proboscis into the flesh to suck blood from the superficial vessels. The insertion of the hypostome is generally unnoticed by the subject. The attached tick may be mistaken by the patient for a new mole. The parasite slowly becomes engorged and then falls off. During this time, which may last for 7–12 days, the patient may have fever, chills, headache, abdominal pain, and vomiting (tick bite pyrexia). Removal of the engorged tick causes a subsidence of the general symptoms in 12–36 hours.<br><br>Q3. With previous information, please write a paragraph to explain in detail the cause of this skin disease.<br><br>A3. Correct identification of an attached tick provides the physician with an opportunity to discuss tick control measures and symptoms of tickborne disease. Most hard ticks have a three-host life cycle. Larval (seed) ticks have six legs and feed on small animals in nature . The nymphs and adults feed on larger animals. Nymphs resemble small adult ticks, with eight legs, but are sexually immature. Because they attach for one long blood

<table>
<tr><td>especially if there are symptoms such as fever, headache, fatigue, or muscle and joint pain, as prompt treatment can prevent serious complications.

Q4. How should I treat it? What medicine should I use?

A4. The treatment for tick bite depends on the severity and the symptoms associated with it. Generally, tick bites are not serious and usually heal on their own within a few weeks without any treatment. However, if there are any signs of infection, such as increasing redness, swelling, or pain, or if you develop a fever or other symptoms, you should seek medical attention. In some cases, antibiotics may be prescribed to prevent infection or to treat an existing infection. The type of antibiotic and the duration of treatment will depend on the specific diagnosis and the severity of the symptoms. It is important to follow the instructions provided by your healthcare provider regarding the use of any medication.</td><td>meal during each phase of the life cycle and search for a new host for each blood meal, ticks are efficient disease vectors. Hard ticks have a hard dorsal plate (scutum) and account for most medically important ticks, transmitting human diseases including Lyme borreliosis, ehrlichiosis, granulocytotropic anaplasmosis and a variety of rickettsial infections (including RockyMountain spotted fever; RMSF) . Soft ticks such as Ornithodoros (the vector of borrelial relapsing fever) lack a scutum and have retroverted mouthparts. These ticks tend to live in close association with a single host.

Q4. How should I treat it? What medicine should I use?

A4. Milder tick bite reactions may respond to application of a potent (class 1 or 2) topical corticosteroid; lesions may fail to improve unless the agent is occluded. More intense reactions often require intralesional corticosteroid injection, and some only respond to surgical excision. Often, the pruritus is so severe that patients express little concern about the discomfort and scar associated with the surgical procedure.Most tick-borne diseases respond readily to tetracyclines. Empiric treatment of suspected RMSF is always appropriate, and therapy should never be delayed until confirmatory tests are available. The consequences of delayed diagnosis and treatment of RMSF are evidenced by reports of deaths in children. Doxycycline is the drug of choice for children as well as adults with RMSF. Staining of teeth is unlikely with a short course of doxycycline, and chloramphenicol is less effective for RMSF.</td></tr>
<tr><td colspan="2">Evaluation of Physician</td></tr>
<tr><td>Evaluation Item</td><td>Score (1-5, the higher the better)</td></tr>
<tr><td>SkinGPT-4’s diagnosis is correct or relevant.</td><td>5</td></tr>
<tr><td>SkinGPT-4’s description is informative.</td><td>4</td></tr>
<tr><td>SkinGPT-4’s suggestions are useful.</td><td>4</td></tr>
</table>

SkinGPT-4 can help doctors with diagnosis.	5
SkinGPT-4 can help patients to understand their disease better.	4
If SkinGPT-4 can be deployed locally, it protects patients' privacy.	5
Willingness to use SkinGPT-4	5

Case Hives 1

Image ID: Hives 1	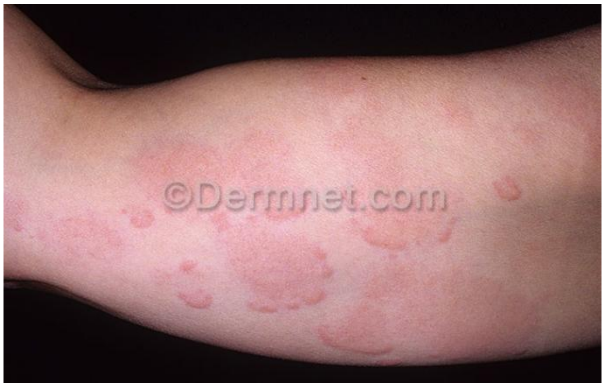
SkinGPT-4	Dermatology Physician
Q1. Could you describe the skin disease in this image for me?<br><br>A1. This could be a skin disease image of hives Urticaria Acute.<br><br>Q2. You should write a paragraph to list more features you observed in the image.<br><br>A2. This image shows the skin manifestation of hives or urticaria. The rash consists of raised, red, itchy bumps on the skin. The bumps are erythematous in color and have a white center. They are spread throughout the body and can be found on various body parts such as the arms, legs, and trunk. The rash is caused by an allergic reaction to a drug or food and can also be associated with other conditions such as angioedema and laryngeal edema. The rash can be treated with antihistamines and corticosteroids.<br><br>Q3. With previous information, please write a paragraph to explain in detail the cause of this skin disease.<br><br>A3. The cause of this skin disease is likely an allergic reaction to a drug or food. The drug or food triggers an immune response in the body, leading to the release of histamine and other inflammatory mediators. These substances cause the symptoms of hives, including the appearance of raised, red, itchy bumps on the skin. The rash can also be associated with other symptoms such as angioedema (swelling of the face, lips, and throat) and laryngeal edema (swelling of the voice	Q1. Could you describe the skin disease in this image for me?<br><br>A1. This could be a skin disease image of hives Urticaria Acute.<br><br>Q2. You should write a paragraph to list more features you observed in the image.<br><br>A2. Urticaria is a vascular reaction of the skin characterized by the appearance of wheals, generally surrounded by a red halo or flare and associated with severe itching, stinging, or pricking sensations. These wheals are caused by localized edema. Clearing of the central region may occur, and lesions may coalesce, producing an annular or polycyclic pattern. Subcutaneous swellings (angioedema) may or may not accompany the wheals. When angioedema is not present, and fever, malaise, and joint/bone pain coexist, an autoinflammatory or autoimmune condition should be considered.<br><br>Q3. With previous information, please write a paragraph to explain in detail the cause of this skin disease.<br><br>A3. In general, infections, ingestants, inhalants, and injections should be considered as possible underlying causes of urticaria. In acute spontaneous cases, URIs and viral infections are the most common etiologies in children. Drugs (e.g., NSAIDs, antibiotics) and foods are other common causes in both adults and children. Clues suggesting physical urticaria as both a primary

box). The rash can be treated with antihistamines and corticosteroids to reduce inflammation and alleviate symptoms. It is important to identify the trigger responsible for the allergy and avoid it to prevent future episodes of hives.<br><br>Q4. How should I treat it? What medicine should I use?<br><br>A4. The treatment of hives or urticaria depends on the severity and duration of symptoms. Mild cases can be treated with over-the-counter antihistamines such as loratadine or cetirizine. For more severe cases, a prescription strength antihistamine may be necessary. In addition, corticosteroids may be prescribed to reduce inflammation and alleviate symptoms. Other treatments such as anti-inflammatory medications and immunomodulatory drugs may also be considered. It is important to follow the instructions of a healthcare provider for proper treatment and to avoid self-medication.	cause and as a coexistent second etiology should be sought historically.<br><br>Q4. How should I treat it? What medicine should I use?<br><br>A4. The mainstay of treatment of acute urticaria is administration of antihistamines. In adults, nonsedating antihistamines pose a lower risk of psychomotor impairment. If the cause of the acute episode can be identified, avoiding that trigger should be stressed. In patients with acute urticaria that does not respond to antihistamines, systemic corticosteroids are generally effective, buthistaminenonresponsive urticaria should prompt consideration for whether there is an underlying disease or continuing trigger present.

Evaluation of Physician	
Evaluation Item	Score (1-5, the higher the better)
SkinGPT-4's diagnosis is correct or relevant.	5
SkinGPT-4's description is informative.	4
SkinGPT-4's suggestions are useful.	5
SkinGPT-4 can help doctors with diagnosis.	5
SkinGPT-4 can help patients to understand their disease better.	4
If SkinGPT-4 can be deployed locally, it protects patients' privacy.	5
Willingness to use SkinGPT-4	5

Case Hives 2

Image ID: Hives2	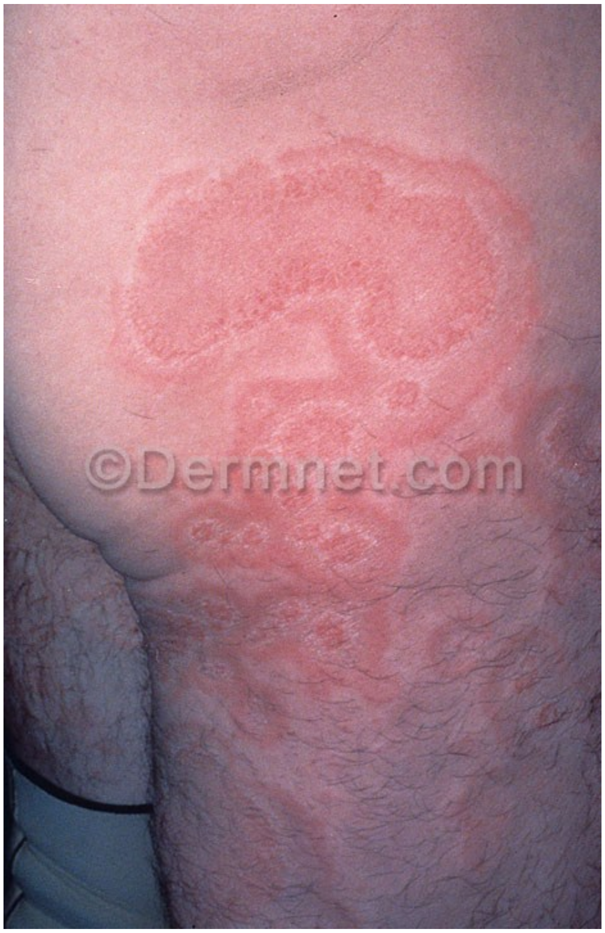
SkinGPT-4	Dermatology Physician
Q1. Could you describe the skin disease in this image for me?<br><br>A1. This could be a skin disease image of granuloma annulare.<br><br>Q2. You should write a paragraph to list more features you observed in the image.<br><br>A2. This image represents a skin condition called granuloma annulare. The lesion is erythematous and indurated with clear borders. The annular plaque is approximately 1.5 cm in diameter and is located on the lower back. Granuloma annulare is a relatively uncommon benign skin disease that affects both children and adults. It is characterized by the development of small, red, raised bumps that often form a ring or circle on the skin. The	Q1. Could you describe the skin disease in this image for me?<br><br>A1. This could be a skin disease image of hives Urticaria Acute.<br><br>Q2. You should write a paragraph to list more features you observed in the image.<br><br>A2. Urticaria is a vascular reaction of the skin characterized by the appearance of wheals, generally surrounded by a red halo or flare and associated with severe itching, stinging, or pricking sensations. These wheals are caused by localized edema. Clearing of the central region may occur, and lesions may coalesce, producing an annular or polycyclic pattern. Subcutaneous swellings (angioedema) may or may not accompany the

cause of granuloma annulare is unknown, but it may be triggered by skin trauma, inflammation, or infection. The condition is usually treated with steroids or surgery, and it tends to recur in about 10 percent of patients.<br><br>Q3. With previous information, please write a paragraph to explain in detail the cause of this skin disease.<br><br>A3. Granuloma annulare is a benign skin condition characterized by the formation of small, raised bumps that often form a ring or circle on the skin. The exact cause of granuloma annulare is not known, but it is thought to be an immune-mediated disorder. It is often triggered by skin trauma, inflammation, or infection, and it tends to occur more frequently in individuals with weakened immune systems. The lesions of granuloma annulare are usually painless and do not itch, but they can be slightly inflamed. The borders of the lesions are usually clear and well-defined, and the center is usually more erythematous. The condition is more common in males than in females and usually affects people of all ages, but it is most frequently seen in children and adolescents. Treatment options for granuloma annulare include topical steroids, intralesional corticosteroids, and surgical excision. Recurrence is common, and treatment may need to be repeated to achieve long-term resolution of the lesions.<br><br>Q4. How should I treat it? What medicine should I use?<br><br>A4. Treatment options for granuloma annulare include topical steroids, intralesional corticosteroids, and surgical excision. The choice of treatment depends on the size, number, and location of the lesions, as well as the patient's age, medical history, and overall health.<br>Topical steroids are commonly used to treat granuloma annulare. They are applied directly to the skin in the form of creams, ointments, or gels. Steroid-containing creams or ointments are usually applied once or twice a day to the affected areas for several weeks to several months.<br>Intralesional corticosteroids involve injecting a steroid medication directly into the center of the lesion. This is usually done in a dermatologist's office using a small needle. Intralesional	wheals. When angioedema is not present, and fever, malaise, and joint/bone pain coexist, an autoinflammatory or autoimmune condition should be considered.<br><br>Q3. With previous information, please write a paragraph to explain in detail the cause of this skin disease.<br><br>A3. In general, infections, ingestants, inhalants, and injections should be considered as possible underlying causes of urticaria. In acute spontaneous cases, URIs and viral infections are the most common etiologies in children. Drugs (e.g., NSAIDs, antibiotics) and foods are other common causes in both adults and children. Clues suggesting physical urticaria as both a primary cause and as a coexistent second etiology should be sought historically.<br><br>Q4. How should I treat it? What medicine should I use?<br><br>A4. The mainstay of treatment of acute urticaria is administration of antihistamines. In adults, nonsedating antihistamines pose a lower risk of psychomotor impairment. If the cause of the acute episode can be identified, avoiding that trigger should be stressed. In patients with acute urticaria that does not respond to antihistamines, systemic corticosteroids are generally effective, buthistaminenonresponsive urticaria should prompt consideration for whether there is an underlying disease or continuing trigger present.

corticosteroids are effective, but they may be more painful and require more sessions than topical steroids.<br>Surgical excision is a last resort for granuloma annulare. It is usually reserved for lesions that are large, numerous, or recurrent, or for those that are causing functional or cosmetic problems. Surgery involves removing the lesion and a small amount of surrounding tissue to prevent regrowth.<br>It is important to note that granuloma annulare can.	
Evaluation of Physician	
Evaluation Item	Score (1-5, the higher the better)
SkinGPT-4's diagnosis is correct or relevant.	1
SkinGPT-4's description is informative.	1
SkinGPT-4's suggestions are useful.	1
SkinGPT-4 can help doctors with diagnosis.	1
SkinGPT-4 can help patients to understand their disease better.	1
If SkinGPT-4 can be deployed locally, it protects patients' privacy.	1
Willingness to use SkinGPT-4	1

Case Hives 3

<table>
<tr><td>Image ID: Hives3</td><td>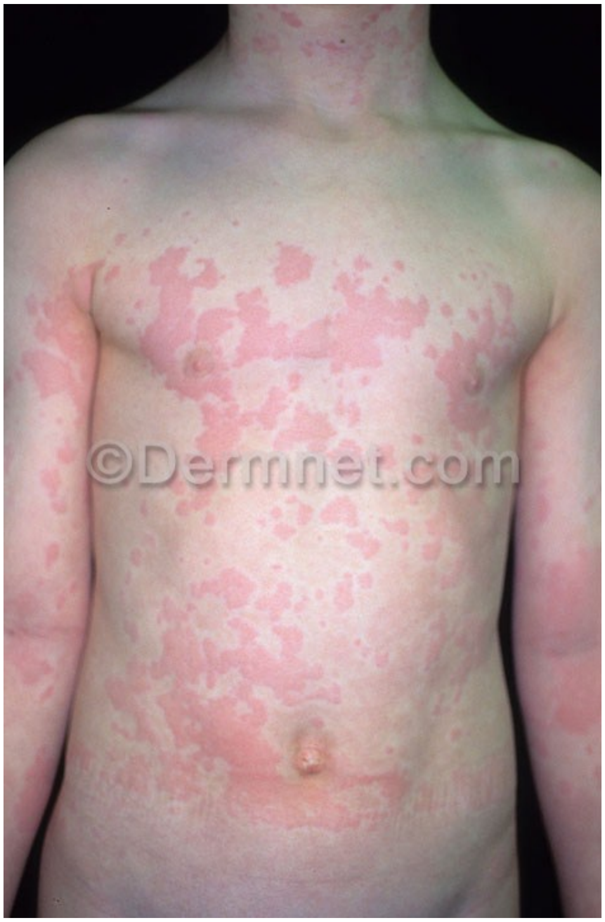
</td></tr>
<tr><th>SkinGPT-4</th><th>Dermatology Physician</th></tr>
<tr><td>Q1. Could you describe the skin disease in this image for me?

A1. This could be a skin disease image of hives Urticaria Acute.

Q2. You should write a paragraph to list more features you observed in the image.

A2. The image shows a child with a rash on the forehead, nose, and cheeks. The rash is erythematous and has a blotchy appearance. The child also has erythema annulare centrifugum, which is a ring shaped eruption on the trunk. The child has a mildly pruritic rash, and there is no blistering or oozing. The eruption is most likely caused by an allergic reaction to a drug or food. The child's medical history is significant for a recent outbreak of hives, which may be related to</td><td>Q1. Could you describe the skin disease in this image for me?

A1. This could be a skin disease image of hives Urticaria Acute.

Q2. You should write a paragraph to list more features you observed in the image.

A2. Urticaria is a vascular reaction of the skin characterized by the appearance of wheals, generally surrounded by a red halo or flare and associated with severe itching, stinging, or pricking sensations. These wheals are caused by localized edema. Clearing of the central region may occur, and lesions may coalesce, producing an annular or polycyclic pattern. Subcutaneous swellings (angioedema) may or may not accompany the wheals. When angioedema is not present, and</td></tr>
</table>

the current eruption. A biopsy of the eruption could be performed to confirm the diagnosis. The child should be advised to avoid any triggering agents and to use antihistamines and corticosteroids as needed. The child should also be referred to a dermatologist for further evaluation and management.<br><br>Q3. With previous information, please write a paragraph to explain in detail the cause of this skin disease.<br><br>A3. The cause of this skin disease is most likely an allergic reaction to a drug or food. The symptoms and appearance of the rash are consistent with erythema annulare, which is a type of allergy-induced skin inflammation. The rash typically affects children and is caused by an immune response to a substance that is otherwise harmless. The rash usually starts as a small red spot that slowly enlarges and forms a ring or circle. The center of the circle tends to clear, giving it a “bull’s-eye” appearance. The rash can be itchy, and blisters may also form. The condition is usually not serious, but it can be difficult to treat. The diagnosis is based on the appearance of the rash and the patient’s history of exposure to a possible allergen. The treatment includes avoiding the allergen and using topical or or oral steroids to reduce inflammation. The rash usually resolves on its own in a few weeks to a few months, but it can take longer in some cases. If the rash does not improve with treatment, a referral to a dermatologist may be necessary.<br><br>Q4. How should I treat it? What medicine should I use?<br><br>A4. The treatment of erythema annulare typically involves avoiding the allergen that caused the allergy and using antihistamines and corticosteroids to reduce inflammation. The rash usually resolves on its own in a few weeks to a few months, but it can take longer in some cases. If the rash does not improve with treatment, a referral to a dermatologist may be necessary.<br><br>For antihistamines, you can use Benadryl or Claritin.<br><br>For corticosteroids, you can use a topical steroid such as hydrocortisone or a systemic steroid like	fever, malaise, and joint/bone pain coexist, an autoinflammatory or autoimmune condition should be considered.<br><br>Q3. With previous information, please write a paragraph to explain in detail the cause of this skin disease.<br><br>A3. In general, infections, ingestants, inhalants, and injections should be considered as possible underlying causes of urticaria. In acute spontaneous cases, URIs and viral infections are the most common etiologies in children. Drugs (e.g., NSAIDs, antibiotics) and foods are other common causes in both adults and children. Clues suggesting physical urticaria as both a primary cause and as a coexistent second etiology should be sought historically.<br><br>Q4. How should I treat it? What medicine should I use?<br><br>A4. The mainstay of treatment of acute urticaria is administration of antihistamines. In adults, nonsedating antihistamines pose a lower risk of psychomotor impairment. If the cause of the acute episode can be identified, avoiding that trigger should be stressed. In patients with acute urticaria that does not respond to antihistamines, systemic corticosteroids are generally effective, buthistaminenonresponsive urticaria should prompt consideration for whether there is an underlying disease or continuing trigger present.

prednisone, if prescribed by a doctor.<br>It is important to follow the instructions of your doctor and use the medication as prescribed.	
Evaluation of Physician	
Evaluation Item	Score (1-5, the higher the better)
SkinGPT-4's diagnosis is correct or relevant.	5
SkinGPT-4's description is informative.	4
SkinGPT-4's suggestions are useful.	3
SkinGPT-4 can help doctors with diagnosis.	5
SkinGPT-4 can help patients to understand their disease better.	4
If SkinGPT-4 can be deployed locally, it protects patients' privacy.	5
Willingness to use SkinGPT-4	5

Case Hives 4

Image ID: Hives4	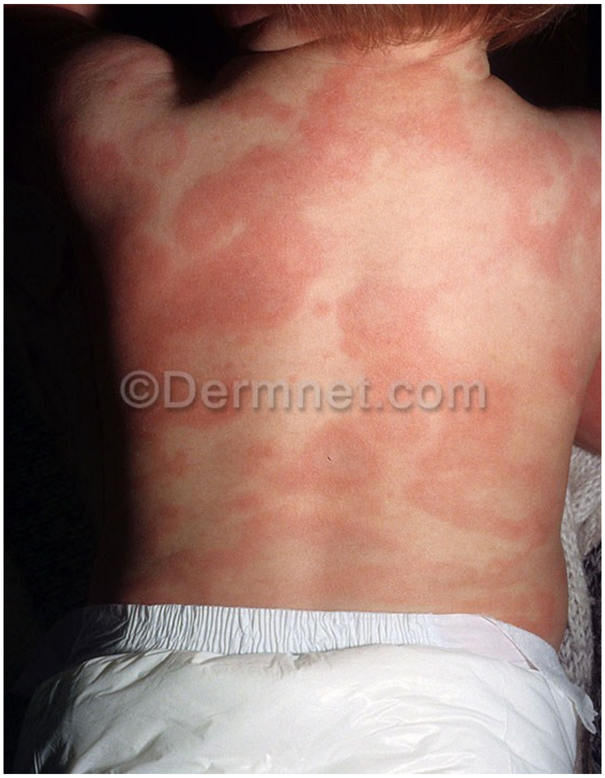
SkinGPT-4	Dermatology Physician
Q1. Could you describe the skin disease in this image for me?<br><br>A1. This could be a skin disease image of angioedema.<br><br>Q2. You should write a paragraph to list more features you observed in the image.<br><br>A2. The child has a smooth, erythematous, central patch on the upper body, and peripheral edema. There is a clear boundary between the central and peripheral patches. The edema involves the hands, feet, and face. The eyelids are involved, with swelling of the upper eyelids and lids with a thick, white plaque on the lower lid. The lips are also swollen, with a white plaque on the upper lip. The child has a clear, fluid -filled blister on the thumb. There is no pitting edema. The child has a rash on the trunk and extremities. The rash is erythematous and does not blanch. The child has no other symptoms, such as fever, vomiting, or diarrhea. This could be a picture of a child with	Q1. Could you describe the skin disease in this image for me?<br><br>A1. This could be a skin disease image of hives Urticaria Acute.<br><br>2. You should write a paragraph to list more features you observed in the image.<br><br>A2. Urticaria is a vascular reaction of the skin characterized by the appearance of wheals, generally surrounded by a red halo or flare and associated with severe itching, stinging, or pricking sensations. These wheals are caused by localized edema. Clearing of the central region may occur, and lesions may coalesce, producing an annular or polycyclic pattern. Subcutaneous swellings (angioedema) may or may not accompany the wheals. When angioedema is not present, and fever, malaise, and joint/bone pain coexist, an autoinflammatory or autoimmune condition should be considered.

angioedema.<br><br>Q3. With previous information, please write a paragraph to explain in detail the cause of this skin disease.<br><br>A3. This skin disease is caused by an allergic reaction to the venom of the Lone Star tick. The tick is found in certain parts of the United States and Canada, and the allergy is most common in children. The tick bite can cause a variety of symptoms, including erythema migrans, fever, fatigue, and muscle and joint pain. The tick bite can also cause a more severe reaction known as tick-borne disease, which can cause symptoms such as facial palsy, meningitis, and even death. In this case, the child is experiencing angioedema, which is a rare but serious allergic reaction that causes swelling of the face and extremities. The reaction can be triggered by a variety of factors, including infection, medications, and insect bites or stings. In this case, the child is experiencing angioedema due to the tick bite. The tick bite can cause a severe allergic reaction that leads to the release of various chemical mediators, including histamine, which causes blood vessels to leak fluid and lead to swelling. The tick bite can also cause the formation of antibodies against various tick proteins, which can lead to the development of an allergy to the tick bite.<br><br>Q4. How should I treat it? What medicine should I use?<br><br>A4. The treatment of angioedema caused by a tick bite will depend on the severity of the symptoms. Mild cases of angioedema can be treated with over-the-counter antihistamines, which can help reduce swelling and itching. More severe cases may require prescription medications, such as corticosteroids, which can help reduce inflammation and swelling. In some cases, a plasma expander, such as fresh frozen plasma or platelets, may be given to help reduce the amount of histamine and other chemical mediators in the body. In cases where the tick bite has caused an allergic reaction, the person may be prescribed an epinephrine auto-injector to help alleviate severe symptoms such as difficulty breathing or a rapid heartbeat. It is important to seek medical attention if you experience symptoms of angioedema, as prompt treatment can help prevent serious	Q3. With previous information, please write a paragraph to explain in detail the cause of this skin disease.<br><br>A3. In general, infections, ingestants, inhalants, and injections should be considered as possible underlying causes of urticaria. In acute spontaneous cases, URIs and viral infections are the most common etiologies in children. Drugs (e.g., NSAIDs, antibiotics) and foods are other common causes in both adults and children. Clues suggesting physical urticaria as both a primary cause and as a coexistent second etiology should be sought historically.<br><br>Q4. How should I treat it? What medicine should I use?<br><br>A4. The mainstay of treatment of acute urticaria is administration of antihistamines. In adults, nonsedating antihistamines pose a lower risk of psychomotor impairment. If the cause of the acute episode can be identified, avoiding that trigger should be stressed. In patients with acute urticaria that does not respond to antihistamines, systemic corticosteroids are generally effective, buthistaminenonresponsive urticaria should prompt consideration for whether there is an underlying disease or continuing trigger present.

complications.	
Evaluation of Physician	
Evaluation Item	Score (1-5, the higher the better)
SkinGPT-4's diagnosis is correct or relevant.	2
SkinGPT-4's description is informative.	1
SkinGPT-4's suggestions are useful.	1
SkinGPT-4 can help doctors with diagnosis.	1
SkinGPT-4 can help patients to understand their disease better.	1
If SkinGPT-4 can be deployed locally, it protects patients' privacy.	1
Willingness to use SkinGPT-4	1

Case Hives 5

Image ID: Hives5	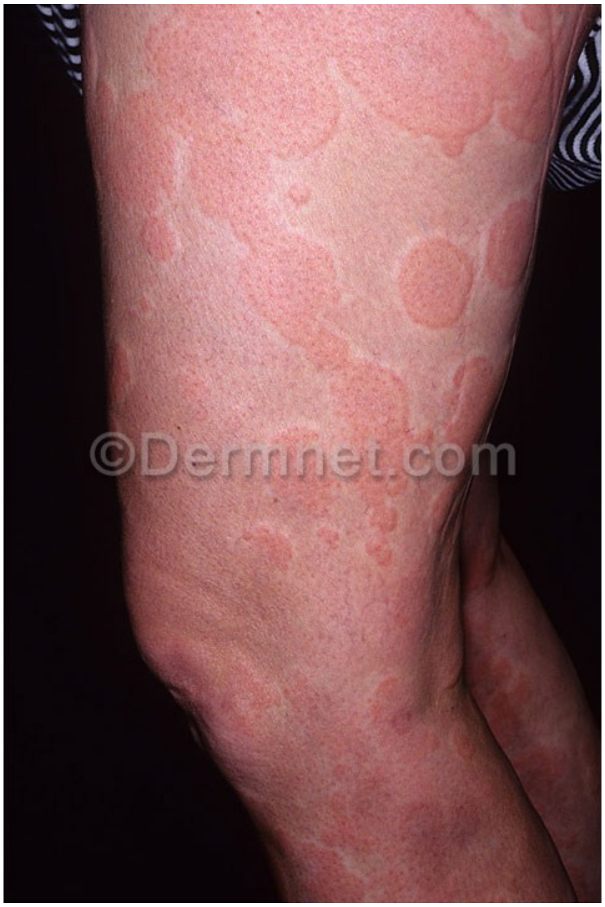
SkinGPT-4	Dermatology Physician
Q1. Could you describe the skin disease in this image for me?<br><br>A1. This could be a skin disease image of hives Urticaria Acute.<br><br>Q2. You should write a paragraph to list more features you observed in the image.<br><br>A2. This image shows a skin disease called hives or urticaria. The patient has red, raised, itchy bumps on the skin that are erythematous in color. The bumps are usually transient and can appear anywhere on the body. The eruption can be solitary or generalized and can be accompanied by angioedema. The disease can be acute or chronic and can be caused by an underlying autoimmune disorder or allergy. The diagnosis is mainly clinical and laboratory tests are not usually	Q1. Could you describe the skin disease in this image for me?<br><br>A1. This could be a skin disease image of hives Urticaria Acute.<br><br>Q2. You should write a paragraph to list more features you observed in the image.<br><br>A2. Urticaria is a vascular reaction of the skin characterized by the appearance of wheals, generally surrounded by a red halo or flare and associated with severe itching, stinging, or pricking sensations. These wheals are caused by localized edema. Clearing of the central region may occur, and lesions may coalesce, producing an annular or polycyclic pattern. Subcutaneous swellings (angioedema) may or may not accompany the wheals. When angioedema is not present, and

needed. Treatment is symptomatic and includes antihistamines, corticosteroids, and epinephrine for severe reactions. The image shows a macroscopic view of the skin, which is the main organ of the immune system, and it is responsible for protecting the body against external factors such as infections, trauma, and UV radiation. The skin is a complex organ composed of several layers, including the epidermis, dermis, and subcutaneous tissue, which work together to maintain the integrity of the skin barrier and protect the body against infection and dehydration.<br><br>Q3. With previous information, please write a paragraph to explain in detail the cause of this skin disease.<br><br>A3. This image shows a skin condition known as hives or urticaria. Hives are red, itchy bumps that appear on the skin, usually as a result of an allergic reaction or an immune system response. They can be caused by a variety of factors, including foods, medications, insect bites, and underlying medical conditions such as thyroid disease or cancer. In some cases, hives can also be caused by viral or bacterial infections. The hives in this image are likely to be caused by an immune system response, as they are erythematous in color and appear as individual bumps on the skin. The symptoms of hives can be treated with antihistamines and other medications to reduce inflammation and itching. The exact cause of hives in this image is not visible, but it could be caused by any of the factors mentioned above. It is important to note that hives are generally not a serious condition and resolve on their own within a few weeks. However, if hives are accompanied by difficulty breathing, swelling of the face and lips, or a rapid heartbeat, medical attention should be sought immediately.<br><br>Q4. How should I treat it? What medicine should I use?<br><br>A4. The treatment of hives or urticaria depends on the underlying cause of the condition. In most cases, hives are caused by an allergic reaction or an immune system response and can be treated symptomatically with antihistamines and other medications. The most common symptoms of hives are itching and redness of the skin, which	fever, malaise, and joint/bone pain coexist, an autoinflammatory or autoimmune condition should be considered.<br><br>Q3. With previous information, please write a paragraph to explain in detail the cause of this skin disease.<br><br>A3. In general, infections, ingestants, inhalants, and injections should be considered as possible underlying causes of urticaria. In acute spontaneous cases, URIs and viral infections are the most common etiologies in children. Drugs (e.g., NSAIDs, antibiotics) and foods are other common causes in both adults and children. Clues suggesting physical urticaria as both a primary cause and as a coexistent second etiology should be sought historically.<br><br>Q4. How should I treat it? What medicine should I use?<br><br>A4. The mainstay of treatment of acute urticaria is administration of antihistamines. In adults, nonsedating antihistamines pose a lower risk of psychomotor impairment. If the cause of the acute episode can be identified, avoiding that trigger should be stressed. In patients with acute urticaria that does not respond to antihistamines, systemic corticosteroids are generally effective, buthistaminenonresponsive urticaria should prompt consideration for whether there is an underlying disease or continuing trigger present.

can be treated with over-the-counter antihistamines such as Benadryl or Claritin. Cold compresses or baths can also be used to relieve itching and reduce inflammation. If the hives are severe or accompanied by other symptoms such as difficulty breathing, swelling of the face and lips, or a rapid heartbeat, medical attention should be sought immediately. In cases where hives are caused by an infection, antibiotics or antivirals may be prescribed. In some cases, hives may be caused by an underlying medical condition, such as thyroid disease or cancer, and treatment may involve managing the underlying condition. It is important to consult with a healthcare provider for proper diagnosis and treatment of hives.	
Evaluation of Physician	
Evaluation Item	Score (1-5, the higher the better)
SkinGPT-4's diagnosis is correct or relevant.	5
SkinGPT-4's description is informative.	3
SkinGPT-4's suggestions are useful.	3
SkinGPT-4 can help doctors with diagnosis.	5
SkinGPT-4 can help patients to understand their disease better.	4
If SkinGPT-4 can be deployed locally, it protects patients' privacy.	5
Willingness to use SkinGPT-4	5

Case Hives 6

Image ID: Hives6	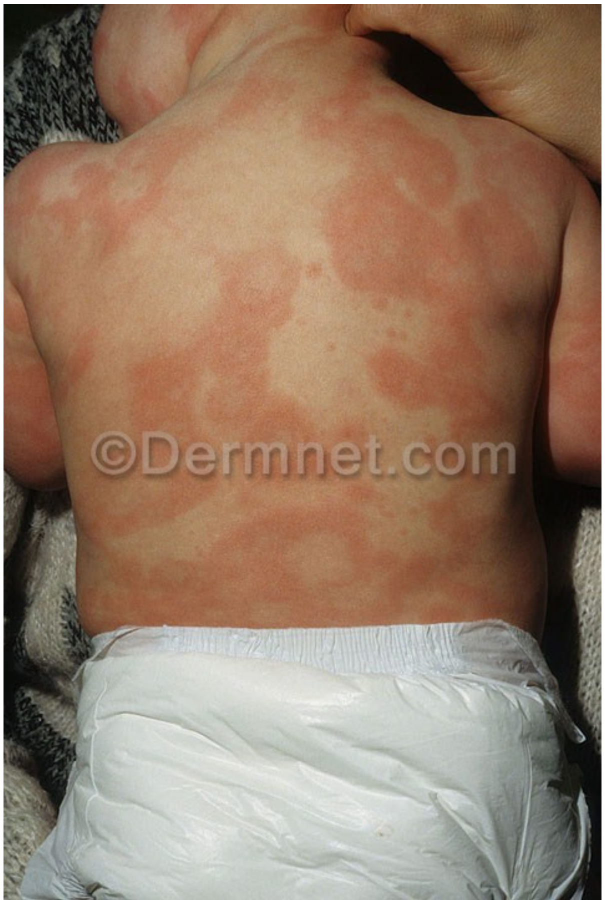
**SkinGPT-4**	**Dermatology Physician**
Q1. Could you describe the skin disease in this image for me?<br><br>A1. This could be a skin disease image of angioedema.<br><br>Q2. You should write a paragraph to list more features you observed in the image.<br><br>A2. The image shows a baby with angioedema, a condition characterized by severe swelling of the face, extremities, and genitalia. The affected areas are flat and indurated, and the skin is erythematous. The swelling is so severe that it obscures the child's facial features, making it difficult to identify the race. The condition is likely caused by a primary immunodeficiency disorder or a reaction to medication. It can be life threatening if airway and respiratory involvement is present,	Q1. Could you describe the skin disease in this image for me?<br><br>A1. This could be a skin disease image of hives Urticaria Acute.<br><br>Q2. You should write a paragraph to list more features you observed in the image.<br><br>A2. Urticaria is a vascular reaction of the skin characterized by the appearance of wheals, generally surrounded by a red halo or flare and associated with severe itching, stinging, or pricking sensations. These wheals are caused by localized edema. Clearing of the central region may occur, and lesions may coalesce, producing an annular or polycyclic pattern. Subcutaneous swellings (angioedema) may or may not accompany the

and requires prompt medical attention.<br><br>Q3. With previous information, please write a paragraph to explain in detail the cause of this skin disease.<br><br>A3. This child has neonatal lupus, a rare autoimmune disorder that occurs in newborns of women with systemic lupus erythematosus (SLE). It is caused by the transfer of maternal antibodies across the placenta, which can lead to hemolytic anemia, thrombocytopenia, and hepatitis in the newborn. The skin rash is a characteristic feature of neonatal lupus and is due to the activation of the immune system, resulting in the production of autoantibodies that attack the skin cells. The rash is typically observed on the face, hands, and feet, and is accompanied by fever and lethargy. Neonatal lupus is usually self-limiting and resolves within a few months without any specific treatment, but affected newborns should be closely monitored for any signs of more severe symptoms. It is important to note that the mother has systemic lupus erythematosus, which is an autoimmune disease that can affect multiple organ systems, including the skin, joints, kidneys, and nervous system.<br><br>Q4. How should I treat it? What medicine should I use?<br><br>A4. The treatment of neonatal lupus depends on the severity of the symptoms. Mild cases may not require any specific treatment, as the condition is usually self-limiting and resolves within a few months. However, newborns with more severe symptoms may require supportive care, such as phototherapy for jaundice, blood transfusions for anemia, and antibiotics for infections. Corticosteroids may also be used to suppress the immune system and reduce inflammation. It is important to consult with a pediatrician or a pediatric rheumatologist for proper diagnosis and treatment.<br>Please note that the use of corticosteroids in neonatal lupus is controversial, as it may have side effects such as growth retardation, hyperglycemia, and adrenal suppression. The benefits and risks of corticosteroid therapy should be carefully weighed on a case-by-case basis.	wheals. When angioedema is not present, and fever, malaise, and joint/bone pain coexist, an autoinflammatory or autoimmune condition should be considered.<br><br>Q3. With previous information, please write a paragraph to explain in detail the cause of this skin disease.<br><br>A3. In general, infections, ingestants, inhalants, and injections should be considered as possible underlying causes of urticaria. In acute spontaneous cases, URIs and viral infections are the most common etiologies in children. Drugs (e.g., NSAIDs, antibiotics) and foods are other common causes in both adults and children. Clues suggesting physical urticaria as both a primary cause and as a coexistent second etiology should be sought historically.<br><br>Q4. How should I treat it? What medicine should I use?<br><br>A4. The mainstay of treatment of acute urticaria is administration of antihistamines. In adults, nonsedating antihistamines pose a lower risk of psychomotor impairment. If the cause of the acute episode can be identified, avoiding that trigger should be stressed. In patients with acute urticaria that does not respond to antihistamines, systemic corticosteroids are generally effective, buthistaminenonresponsive urticaria should prompt consideration for whether there is an underlying disease or continuing trigger present.
Evaluation of Physician	

Evaluation Item	Score (1-5, the higher the better)
SkinGPT-4's diagnosis is correct or relevant.	1
SkinGPT-4's description is informative.	1
SkinGPT-4's suggestions are useful.	1
SkinGPT-4 can help doctors with diagnosis.	1
SkinGPT-4 can help patients to understand their disease better.	1
If SkinGPT-4 can be deployed locally, it protects patients' privacy.	1
Willingness to use SkinGPT-4	1

Case Hives 7

<table>
<tr><td>Image ID: Hives7</td><td>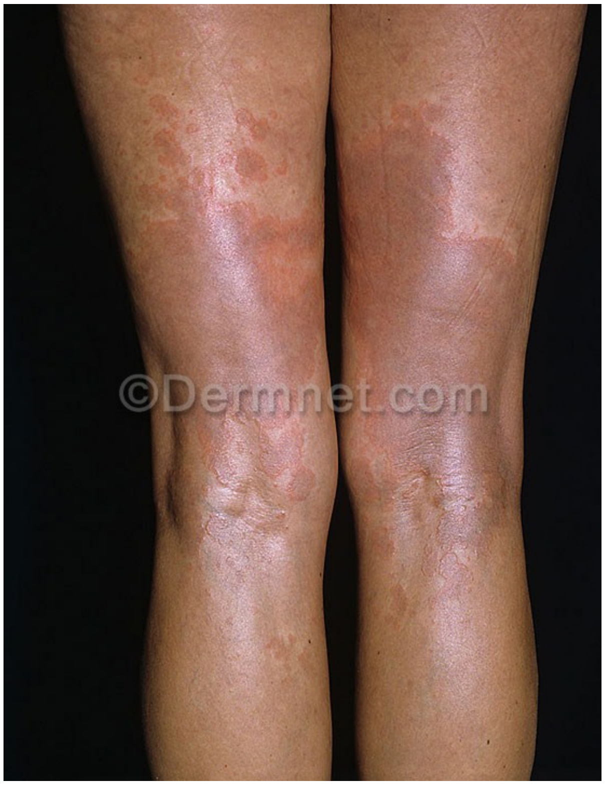
</td></tr>
<tr><th>SkinGPT-4</th><th>Dermatology Physician</th></tr>
<tr><td>Q1. Could you describe the skin disease in this image for me?

A1. This could be a skin disease image of hives Urticaria Acute.

Q2. You should write a paragraph to list more features you observed in the image.

A2. This image depicts a skin condition known as hives or urticaria acute. The skin is covered with raised, red, itchy bumps that have a white center. The bumps are various sizes and shapes and can be found anywhere on the body. The condition is caused by an allergic reaction to certain foods or medications, or by an underlying autoimmune disorder. The lesions are usually transient and resolve within a few weeks, but they can also be chronic and require treatment with antihistamines or steroids. The affected area is usually limited to a specific part of the body. This condition is not contagious and does not leave a scar. It is important to identify the triggers and avoid them to</td><td>Q1. Could you describe the skin disease in this image for me?

A1. This could be a skin disease image of hives Urticaria Acute.

Q2. You should write a paragraph to list more features you observed in the image.

A2. Urticaria is a vascular reaction of the skin characterized by the appearance of wheals, generally surrounded by a red halo or flare and associated with severe itching, stinging, or pricking sensations. These wheals are caused by localized edema. Clearing of the central region may occur, and lesions may coalesce, producing an annular or polycyclic pattern. Subcutaneous swellings (angioedema) may or may not accompany the wheals. When angioedema is not present, and fever, malaise, and joint/bone pain coexist, an autoinflammatory or autoimmune condition should be considered.</td></tr>
</table>

prevent future episodes.<br><br>Q3. With previous information, please write a paragraph to explain in detail the cause of this skin disease.<br><br>A3. The cause of this skin disease is not clearly known. It is believed to be an autoimmune disorder, in which the immune system attacks the body's own tissues, leading to inflammation and damage. The condition is characterized by the presence of patches on the skin that are dry, scaly, and flaky. The patches can appear on various parts of the body, including the scalp, elbows, knees, and soles of the feet. The condition is not contagious and is usually not itchy. It is commonly seen in people with a family history of autoimmune disorders and in those with a fair skin tone. The condition can be managed with topical corticosteroids, moisturizers, and avoiding harsh soaps and detergents. In severe cases, oral corticosteroids or other immunosuppressive medications may be prescribed.<br><br>Q4. How should I treat it? What medicine should I use?<br><br>A4. The treatment of psoriasis depends on the severity and location of the disease, as well as individual preferences and medical history. Treatment options include topical creams and ointments, phototherapy, and systemic medications.<br>Topical treatments: Topical creams and ointments are commonly used to treat mild to moderate psoriasis. They include corticosteroids, vitamin D analogs, and moisturizers.<br>Phototherapy: Phototherapy involves exposure to ultraviolet light to slow the growth of skin cells and reduce inflammation. It can be done at home or in a doctor's office.<br>Systemic medications: For severe or widespread psoriasis, systemic medications such as immunosuppressants, biologics, and immunomodulatory agents may be prescribed. These medications work to slow the growth of skin cells and reduce inflammation throughout the body.<br>It is important to note that treatment options should be discussed with a dermatologist or other healthcare provider to determine the best course	Q3. With previous information, please write a paragraph to explain in detail the cause of this skin disease.<br><br>A3. In general, infections, ingestants, inhalants, and injections should be considered as possible underlying causes of urticaria. In acute spontaneous cases, URIs and viral infections are the most common etiologies in children. Drugs (e.g., NSAIDs, antibiotics) and foods are other common causes in both adults and children. Clues suggesting physical urticaria as both a primary cause and as a coexistent second etiology should be sought historically.<br><br>Q4. How should I treat it? What medicine should I use?<br><br>A4. The mainstay of treatment of acute urticaria is administration of antihistamines. In adults, nonsedating antihistamines pose a lower risk of psychomotor impairment. If the cause of the acute episode can be identified, avoiding that trigger should be stressed. In patients with acute urticaria that does not respond to antihistamines, systemic corticosteroids are generally effective, buthistaminenonresponsive urticaria should prompt consideration for whether there is an underlying disease or continuing trigger present.

of treatment for individual cases.	
Evaluation of Physician	
Evaluation Item	Score (1-5, the higher the better)
SkinGPT-4's diagnosis is correct or relevant.	5
SkinGPT-4's description is informative.	2
SkinGPT-4's suggestions are useful.	2
SkinGPT-4 can help doctors with diagnosis.	5
SkinGPT-4 can help patients to understand their disease better.	2
If SkinGPT-4 can be deployed locally, it protects patients' privacy.	3
Willingness to use SkinGPT-4	3

Case Hives 8

Image ID: Hives8	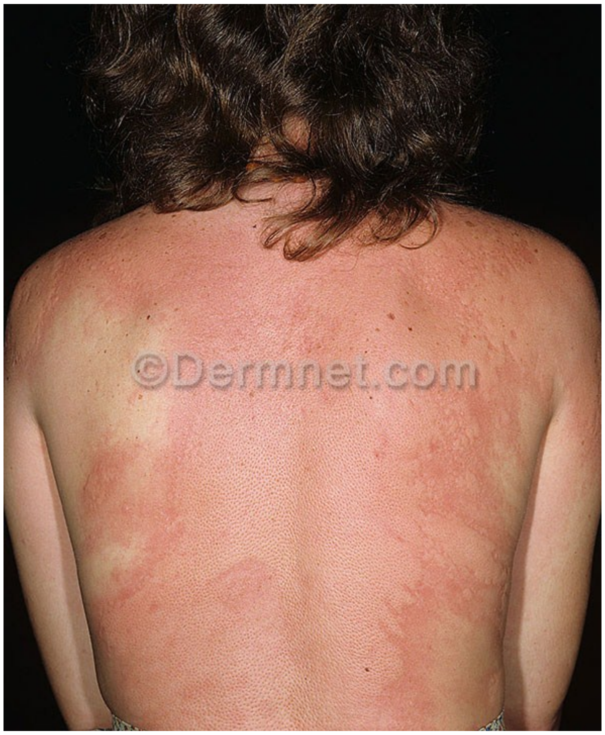
SkinGPT-4	Dermatology Physician
Q1. Could you describe the skin disease in this image for me?<br><br>A1. This could be a skin disease image of phototoxic reactions.<br><br>Q2. You should write a paragraph to list more features you observed in the image.<br><br>A2. The image shows a person's back with a large red rash, which is likely a reaction to a medication. The rash consists of small, red, inflamed bumps that are closely grouped together, with some clear fluid in the center of each bump. The rash is accompanied by blistering and weeping. There is also erythema, which is a general redness of the skin, and excoriation, which is skin damage caused by scratching. The affected area is likely extremely painful and itchy, making it difficult for the person to avoid scratching.<br><br>Q3. With previous information, please write a paragraph to explain in detail the cause of this skin disease.	Q1. Could you describe the skin disease in this image for me?<br><br>A1. This could be a skin disease image of hives Urticaria Acute.<br><br>Q2. You should write a paragraph to list more features you observed in the image.<br><br>A2. Urticaria is a vascular reaction of the skin characterized by the appearance of wheals, generally surrounded by a red halo or flare and associated with severe itching, stinging, or pricking sensations. These wheals are caused by localized edema. Clearing of the central region may occur, and lesions may coalesce, producing an annular or polycyclic pattern. Subcutaneous swellings (angioedema) may or may not accompany the wheals. When angioedema is not present, and fever, malaise, and joint/bone pain coexist, an autoinflammatory or autoimmune condition should be considered.<br><br>Q3. With previous information, please write a

A3. The cause of this skin disease is likely an allergic reaction to a medication. The rash is a type of contact dermatitis, which is caused by exposure to an irritant or allergen that triggers an immune response in the skin. The medication causing the reaction is likely one that the person has been prescribed, and the rash is a result of the body's immune system reacting to the medication. The symptoms of the rash, such as the small red bumps, blistering, weeping, and skin damage, are characteristic of an allergic reaction. The presence of erythema and excoriation further supports the diagnosis of contact dermatitis. The fact that the rash is on the back, which is a common site for people to wear clothing that may come into contact with the skin, also supports the diagnosis of contact dermatitis.<br><br>Q4. How should I treat it? What medicine should I use?<br><br>A4. The treatment of this skin disease will depend on the severity and duration of the rash. The rash may be treated with over-the-counter hydrocortisone cream or calamine lotion to help reduce itching and inflammation. If the rash is severe or does not improve with over-the-counter medication, a prescription steroid cream or or oral antihistamine may be necessary. It is important to avoid scratching the rash, as this can lead to infection and further skin damage. The best course of treatment will depend on the individual case and should be determined by a healthcare professional.	paragraph to explain in detail the cause of this skin disease.<br><br>A3. In general, infections, ingestants, inhalants, and injections should be considered as possible underlying causes of urticaria. In acute spontaneous cases, URIs and viral infections are the most common etiologies in children. Drugs (e.g., NSAIDs, antibiotics) and foods are other common causes in both adults and children. Clues suggesting physical urticaria as both a primary cause and as a coexistent second etiology should be sought historically.<br><br>Q4. How should I treat it? What medicine should I use?<br><br>A4. The mainstay of treatment of acute urticaria is administration of antihistamines. In adults, nonsedating antihistamines pose a lower risk of psychomotor impairment. If the cause of the acute episode can be identified, avoiding that trigger should be stressed. In patients with acute urticaria that does not respond to antihistamines, systemic corticosteroids are generally effective, buthistaminenonresponsive urticaria should prompt consideration for whether there is an underlying disease or continuing trigger present.
Evaluation of Physician	
Evaluation Item	Score (1-5, the higher the better)
SkinGPT-4's diagnosis is correct or relevant.	1
SkinGPT-4's description is informative.	3
SkinGPT-4's suggestions are useful.	4
SkinGPT-4 can help doctors with diagnosis.	1

SkinGPT-4 can help patients to understand their disease better.	1
If SkinGPT-4 can be deployed locally, it protects patients' privacy.	1
Willingness to use SkinGPT-4	1

Case Hives 9

Image ID: Hives9	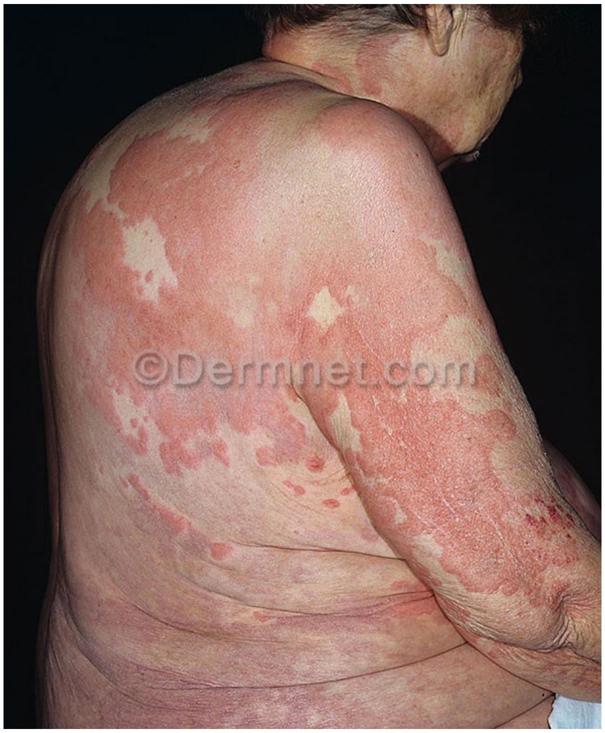
SkinGPT-4	Dermatology Physician
Q1. Could you describe the skin disease in this image for me?<br><br>A1. This could be a skin disease image of hives Urticaria Acute.<br><br>Q2. You should write a paragraph to list more features you observed in the image.<br><br>A2. The image shows a person’s skin with a rash present all over the body. The rash is made up of small, itchy bumps that are red and have white centers. The rash is spread out evenly over the skin and looks quite bad. The skin looks very dry and rough, and there are areas of peeling skin. The rash is also present on the mucous membranes, especially on the tongue and lips. The patient is also experiencing difficulty breathing, wheezing, and coughing. This could be a skin condition such as angioedema or a drug eruption.<br><br>Q3. With previous information, please write a paragraph to explain in detail the cause of this skin	Q1. Could you describe the skin disease in this image for me?<br><br>A1. This could be a skin disease image of hives Urticaria Acute.<br><br>Q2. You should write a paragraph to list more features you observed in the image.<br><br>A2. Urticaria is a vascular reaction of the skin characterized by the appearance of wheals, generally surrounded by a red halo or flare and associated with severe itching, stinging, or pricking sensations. These wheals are caused by localized edema. Clearing of the central region may occur, and lesions may coalesce, producing an annular or polycyclic pattern. Subcutaneous swellings (angioedema) may or may not accompany the wheals. When angioedema is not present, and fever, malaise, and joint/bone pain coexist, an autoinflammatory or autoimmune condition should be considered.<br><br>Q3. With previous information, please write a

disease.<br><br>A3. This skin disease is caused by an allergic reaction to a drug, most likely a beta-lactam antibiotic such as penicillin or cephalosporin. The rash is a classic feature of a drug eruption caused by these antibiotics. The rash usually starts as small, red, itchy bumps that later become blisters, then scab over and fall off, leaving behind dry, rough skin. The rash can also spread to other parts of the body, including the mucous membranes, and can be accompanied by other allergic reactions such as angioedema, which causes swelling of the face, lips, tongue, and throat. The patient may also experience difficulty breathing, wheezing, and coughing. The condition is usually self-limiting and resolves within a week or two after stopping the offending medication.<br><br>Q4. How should I treat it? What medicine should I use?<br><br>A4. The treatment of this skin disease caused by a drug eruption is primarily to discontinue the offending medication. The rash usually resolves within a week or two after stopping the medication. If the rash is severe or accompanied by other symptoms such as difficulty breathing, the patient may need to be hospitalized and treated with corticosteroids and antihistamines. In some cases, an epinephrine injection may be necessary to treat severe reactions. Once the medication is stopped, the skin lesions will begin to improve, and the symptoms will resolve. It is important to consult a dermatologist or a healthcare professional for proper diagnosis and treatment.<br><br>Please confirm if the information provided is accurate and if you have any further questions.	paragraph to explain in detail the cause of this skin disease.<br><br>A3. In general, infections, ingestants, inhalants, and injections should be considered as possible underlying causes of urticaria. In acute spontaneous cases, URIs and viral infections are the most common etiologies in children. Drugs (e.g., NSAIDs, antibiotics) and foods are other common causes in both adults and children. Clues suggesting physical urticaria as both a primary cause and as a coexistent second etiology should be sought historically.<br><br>Q4. How should I treat it? What medicine should I use?<br><br>A4. The mainstay of treatment of acute urticaria is administration of antihistamines. In adults, nonsedating antihistamines pose a lower risk of psychomotor impairment. If the cause of the acute episode can be identified, avoiding that trigger should be stressed. In patients with acute urticaria that does not respond to antihistamines, systemic corticosteroids are generally effective, buthistaminenonresponsive urticaria should prompt consideration for whether there is an underlying disease or continuing trigger present.
Evaluation of Physician	
Evaluation Item	Score (1-5, the higher the better)
SkinGPT-4’s diagnosis is correct or relevant.	5
SkinGPT-4’s description is informative.	2

SkinGPT-4's suggestions are useful.	2
SkinGPT-4 can help doctors with diagnosis.	5
SkinGPT-4 can help patients to understand their disease better.	2
If SkinGPT-4 can be deployed locally, it protects patients' privacy.	2
Willingness to use SkinGPT-4	2

Case Hives 10

Image ID: Hives10	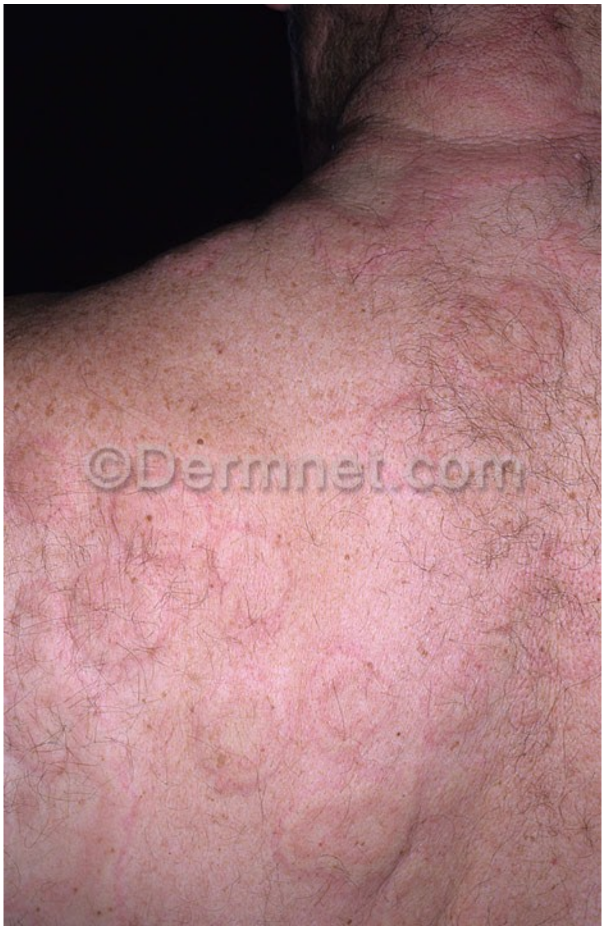
**SkinGPT-4**	**Dermatology Physician**
Q1. Could you describe the skin disease in this image for me?<br><br>A1. This could be a skin disease image of angioedema.<br><br>Q2. You should write a paragraph to list more features you observed in the image.<br><br>A2. The image shows erythema angioedema, a disease characterized by edema and erythema of the skin, caused by an allergic reaction to certain substances. The affected areas are intensely itchy and can be accompanied by other symptoms such as fever, urticaria, and angioedema. The edema usually affects the face, lips, and ears, and can spread to other parts of the body. The erythema is characterized by redness and warmth of the skin,	Q1. Could you describe the skin disease in this image for me?<br><br>A1. This could be a skin disease image of hives Urticaria Acute.<br><br>Q2. You should write a paragraph to list more features you observed in the image.<br><br>A2. Urticaria is a vascular reaction of the skin characterized by the appearance of wheals, generally surrounded by a red halo or flare and associated with severe itching, stinging, or pricking sensations. These wheals are caused by localized edema. Clearing of the central region may occur, and lesions may coalesce, producing an annular or polycyclic pattern. Subcutaneous swellings (angioedema) may or may not accompany the

and can also spread to other parts of the body. The disease can be treated with antihistamines, steroids, and epinephrine.<br><br>Q3. With previous information, please write a paragraph to explain in detail the cause of this skin disease.<br><br>A3. Erythema angioedema is caused by an allergic reaction to certain substances, often medications, food, or insect bites. The reaction leads to the release of histamine and other inflammatory mediators, causing edema, erythema, and itching of the skin. The edema is caused by the increased permeability of the blood vessels, which allows fluid to leak into the tissues. The erythema is caused by the dilation of blood vessels, leading to increased blood flow and redness of the skin. The itching is caused by the activation of itch receptors on the skin, which are stimulated by histamine and other inflammatory mediators. The disease can be treated with antihistamines, steroids, and epinephrine to relieve symptoms and prevent further reactions.<br><br>Q4. How should I treat it? What medicine should I use?<br><br>A4. The treatment of erythema angioedema depends on the severity of symptoms and the underlying cause of the allergy. The following steps can be taken to manage the symptoms:<br><br>* Avoiding triggers: Identifying and avoiding the triggers that cause the allergic reaction can help prevent future episodes.<br>* Antihistamines: Over-the-counter antihistamines, such as Benadryl or Claritin, can help relieve symptoms such as itching and hives.<br>* Cool compresses: Applying a cold compress to the affected area can help reduce swelling and itching.<br>* Steroids: Prescription-strength steroids, such as prednisone, can help reduce inflammation and swelling.<br>* Epinephrine: Epinephrine injection can be used in emergency situations to relieve severe symptoms, such as difficulty breathing or a drop in blood pressure.<br><br>It's important to consult a doctor for proper diagnosis and treatment, as the underlying cause	wheals. When angioedema is not present, and fever, malaise, and joint/bone pain coexist, an autoinflammatory or autoimmune condition should be considered.<br><br>Q3. With previous information, please write a paragraph to explain in detail the cause of this skin disease.<br><br>A3. In general, infections, ingestants, inhalants, and injections should be considered as possible underlying causes of urticaria. In acute spontaneous cases, URIs and viral infections are the most common etiologies in children. Drugs (e.g., NSAIDs, antibiotics) and foods are other common causes in both adults and children. Clues suggesting physical urticaria as both a primary cause and as a coexistent second etiology should be sought historically.<br><br>Q4. How should I treat it? What medicine should I use?<br><br>A4. The mainstay of treatment of acute urticaria is administration of antihistamines. In adults, nonsedating antihistamines pose a lower risk of psychomotor impairment. If the cause of the acute episode can be identified, avoiding that trigger should be stressed. In patients with acute urticaria that does not respond to antihistamines, systemic corticosteroids are generally effective, buthistaminenonresponsive urticaria should prompt consideration for whether there is an underlying disease or continuing trigger present.<br><br>A4.

and severity of symptoms can vary.	
Evaluation of Physician	
Evaluation Item	Score (1-5, the higher the better)
SkinGPT-4's diagnosis is correct or relevant.	2
SkinGPT-4's description is informative.	4
SkinGPT-4's suggestions are useful.	4
SkinGPT-4 can help doctors with diagnosis.	2
SkinGPT-4 can help patients to understand their disease better.	3
If SkinGPT-4 can be deployed locally, it protects patients' privacy.	4
Willingness to use SkinGPT-4	3

Case Vascular Tumors 1

Image ID: Vascular tumor1	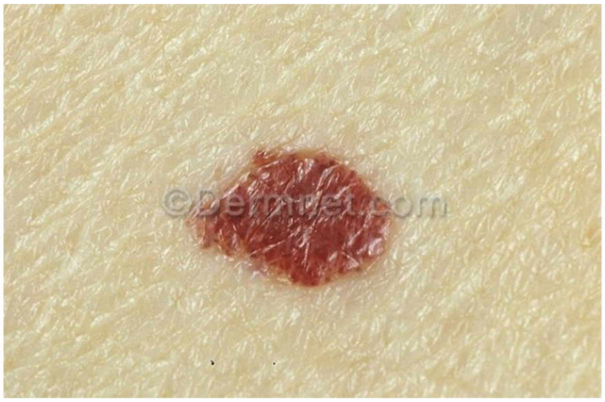
**SkinGPT-4**	**Dermatology Physician**
Q1. Could you describe the skin disease in this image for me?<br><br>A1. This could be a skin disease image of melanocytic nevi.<br><br>Q2. You should write a paragraph to list more features you observed in the image.<br><br>A2. The image is a skin disease image of melanocytic nevus. The lesion is well defined, smooth, and pigmented with a reddish brown color. The nevus is located on the head and neck in 10 percent of cases. It is a congenital nevus and can be present at birth. The nevus can be unilateral or bilateral and can have a size variation. The lesion is a benign tumor and does not metastasize. It is important to monitor the nevus for any changes in size, color, or symptoms.<br><br>Q3. With previous information, please write a paragraph to explain in detail the cause of this skin disease.<br><br>A3. The cause of this skin disease is not clear. It could be due to genetic, environmental or a combination of both. Research has suggested that the development of moles may be influenced by exposure to sunlight, as moles are more common in people with fair skin. However, the relationship between sun exposure and the development of moles is not fully understood. Some studies have suggested that moles may be caused by a genetic predisposition. Some people may have a higher	Q1. Could you describe the skin disease in this image for me?<br><br>A1. This could be a skin disease image of hemangioma.<br><br>Q2. You should write a paragraph to list more features you observed in the image.<br><br>A2. IHs can present anywhere on the body and can be divided based on multiple features. Superficial IHs are easily seen on top of the skin and often have a bright red or maroon look when proliferating. Deep hemangiomas are under the skin and often have a blue hue but are soft. Mixed hemangiomas have features of superficial and deep hemangiomas. Localized hemangiomas are round or oval and have well-defined margins. Segmental or patterned hemangiomas have less distinct borders and appear to grow in embryologic segments. Segmental hemangiomas can be associated with multiple syndromes.<br><br>Q3. With previous information, please write a paragraph to explain in detail the cause of this skin disease.<br><br>A3. IHs go through a characteristic growth pattern with rapid growth occurring most commonly in the first 6 months, although they can grow up to 12 months. The most rapid growth of IH is between 5.5 and 7.5 weeks of life (no matter what the age of gestation) and 90% are finished significantly growing by 4 months, although larger lesions can continue to grow up to a year of life and very rarely

number of moles due to inherited genetic factors. It is also thought that moles may be caused by a combination of genetic and environmental factors. Regardless of the cause, moles are benign tumors of melanocytes and do not spread to other parts of the body. They can be removed for cosmetic reasons or if they are causing symptoms such as itching, bleeding or pain.<br><br>Q4. How should I treat it? What medicine should I use?<br><br>A4. The treatment of congenital nevi, also known as moles, is typically not necessary if the lesion is stable and not causing any symptoms. However, if the nevus is causing symptoms such as itching, bleeding, or pain, or if there are concerns about the lesion being cancerous, then excision may be recommended. The surgical excision is typically done by a dermatologist or a plastic surgeon.<br><br>If the nevus is small and asymptomatic, observation is recommended. However, it is important to monitor the nevus for any changes in size, color, or symptoms. It is also important to perform monthly self-skin examinations to check for any new or changing lesions. If you notice any changes in your nevus, it is important to consult with a dermatologist.	longer. Therefore therapy, when needed, is ideally started early in life to prevent the rapid growth that can lead to tissue distortion. IHs then regress and although the majority of the regression happens by age 4, continued improvement can occur up to and past age 10.<br><br>Q4. How should I treat it? What medicine should I use?<br><br>A4. Simple observation may be appropriate for many hemangiomas, allowing the lesions to regress spontaneously. Parents should be informed of the appropriate time frame and the pros and cons should be explained. For therapy, β-blockers are used most frequently, but systemic treatment can rarely be complicated by bradycardia or hypoglycemia, and regular feedings are critical before and during treatment. Topical timolol, although off label, is extremely effective for superficial hemangiomas and can help heal ulcerations. Oral prednisolone at a dose of 2–4 mg/kg/day has also been used for IH but has mostly been replaced by propranolol.
Evaluation of Physician	
Evaluation Item	Score (1-5, the higher the better)
SkinGPT-4's diagnosis is correct or relevant.	1
SkinGPT-4's description is informative.	4
SkinGPT-4's suggestions are useful.	5
SkinGPT-4 can help doctors with diagnosis.	1
SkinGPT-4 can help patients to understand their disease better.	1
If SkinGPT-4 can be deployed locally, it protects patients' privacy.	1

Willingness to use SkinGPT-4	1

Case Vascular Tumors 2

Image ID: Vascular tumor2	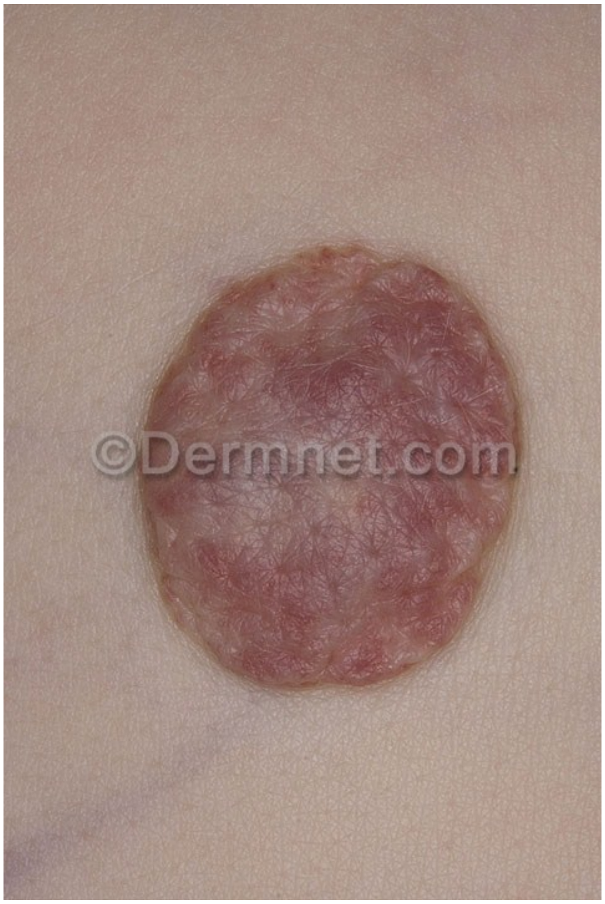
SkinGPT-4	Dermatology Physician
Q1. Could you describe the skin disease in this image for me?<br><br>A1. This could be a skin disease image of hemangioma.<br><br>Q2. You should write a paragraph to list more features you observed in the image.<br><br>A2. The lesion has a smooth, well defined border and is uniform in color. The surface is dome shaped and has a central depression. The lesion measures approximately 1.5 cm in diameter. It is located on the upper back. The patient is a 40 year old male who presents with a new skin lesion. He reports that it has been slowly growing in size over the past several months. He has no other skin lesions or medical problems. The lesion is a hemangioma.	Q1. Could you describe the skin disease in this image for me?<br><br>A1. This could be a skin disease image of hemangioma.<br><br>Q2. You should write a paragraph to list more features you observed in the image.<br><br>A2. IHs can present anywhere on the body and can be divided based on multiple features. Superficial IHs are easily seen on top of the skin and often have a bright red or maroon look when proliferating. Deep hemangiomas are under the skin and often have a blue hue but are soft. Mixed hemangiomas have features of superficial and deep hemangiomas. Localized hemangiomas are round or oval and have well-defined margins. Segmental or patterned hemangiomas have less distinct

Q3. With previous information, please write a paragraph to explain in detail the cause of this skin disease.<br><br>A3. Hemangiomas are caused by an abnormal growth of blood vessels in the skin. They can occur at any time during life, but most commonly occur during the first few months of life. The exact cause of hemangiomas is not known, but it is believed that they may be related to a combination of genetic and environmental factors. Some research suggests that hemangiomas may be triggered by certain genetic mutations, while others propose that exposure to certain environmental toxins, such as alcohol, may play a role in their development. Despite the lack of a clear understanding of the cause, it is generally agreed that hemangiomas do not spread from one person to another, and they do not result from anything the mother did or did not do during pregnancy.<br><br>Q4. How should I treat it? What medicine should I use?<br><br>A4. The treatment of a hemangioma depends on its location, size, and severity. Small hemangiomas that are not causing any symptoms may not require treatment. However, larger hemangiomas or those located in sensitive areas, such as the face or eyes, may need to be treated to improve appearance and prevent vision loss. There are several options for treating hemangiomas, including:<br>* Observation: For small, stable hemangiomas that are not causing any problems, observation may be the best option. The patient should be monitored for any changes in size or symptoms.<br>* Corticosteroids: Corticosteroids are medications that can reduce inflammation and slow down the growth of hemangiomas. They can be applied directly to the skin or given systemically.<br>* Vaporization or laser surgery: These techniques can be used to remove the hemangioma and are often done in a dermatologist's office.<br>* Surgery: For larger hemangiomas or those that are causing symptoms, surgery may be necessary. The surgical method used will depend on the size, location, and severity of the hemangioma.	borders and appear to grow in embryologic segments. Segmental hemangiomas can be associated with multiple syndromes.<br><br>Q3. With previous information, please write a paragraph to explain in detail the cause of this skin disease.<br><br>A3. IHs go through a characteristic growth pattern with rapid growth occurring most commonly in the first 6 months, although they can grow up to 12 months. The most rapid growth of IH is between 5.5 and 7.5 weeks of life (no matter what the age of gestation) and 90% are finished significantly growing by 4 months, although larger lesions can continue to grow up to a year of life and very rarely longer. Therefore therapy, when needed, is ideally started early in life to prevent the rapid growth that can lead to tissue distortion. IHs then regress and although the majority of the regression happens by age 4, continued improvement can occur up to and past age 10.<br><br>Q4. How should I treat it? What medicine should I use?<br><br>A4. Simple observation may be appropriate for many hemangiomas, allowing the lesions to regress spontaneously. Parents should be informed of the appropriate time frame and the pros and cons should be explained. For therapy, β-blockers are used most frequently, but systemic treatment can rarely be complicated by bradycardia or hypoglycemia, and regular feedings are critical before and during treatment. Topical timolol, although off label, is extremely effective for superficial hemangiomas and can help heal ulcerations. Oral prednisolone at a dose of 2–4 mg/kg/day has also been used for IH but has mostly been replaced by propranolol.

Evaluation of Physician	
Evaluation Item	Score (1-5, the higher the better)
SkinGPT-4's diagnosis is correct or relevant.	5
SkinGPT-4's description is informative.	3
SkinGPT-4's suggestions are useful.	4
SkinGPT-4 can help doctors with diagnosis.	4
SkinGPT-4 can help patients to understand their disease better.	4
If SkinGPT-4 can be deployed locally, it protects patients' privacy.	4
Willingness to use SkinGPT-4	4

Case Vascular Tumors 3

Image ID: Vascular tumor3	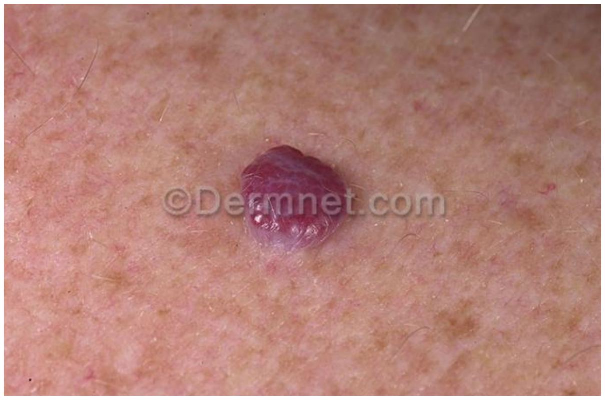
SkinGPT-4	Dermatology Physician
Q1. Could you describe the skin disease in this image for me?<br><br>A1. This could be a skin disease image of hemangioma.<br><br>Q2. You should write a paragraph to list more features you observed in the image.<br><br>A2. The image shows a hemangioma, which is a benign tumor composed of abnormal blood vessels. It is red, circular, and flat. The lesion is well demarcated and is located on the lower third of the face. The overlying skin appears normal. No pigmentation or ulceration is seen. No cervical or regional lymph nodes are palpable. The lesion is not painful or itchy. It has a smooth border and is approximately 5 mm in diameter. The color is red and the surface is shiny.<br><br>Q3. With previous information, please write a paragraph to explain in detail the cause of this skin disease.<br><br>A3. Hemangiomas are the most common tumors of childhood and are characterized by abnormal proliferation of endothelial cells that form blood vessels. The cause of hemangiomas is not completely understood, but it is thought to be related to a combination of genetic and environmental factors. Hemangiomas are more common in females and occur more frequently in Caucasians than in other races. The lesions typically appear during the first few weeks of life	Q1. Could you describe the skin disease in this image for me?<br><br>A1. This could be a skin disease image of hemangioma.<br><br>Q2. You should write a paragraph to list more features you observed in the image.<br><br>A2. IHs can present anywhere on the body and can be divided based on multiple features. Superficial IHs are easily seen on top of the skin and often have a bright red or maroon look when proliferating. Deep hemangiomas are under the skin and often have a blue hue but are soft. Mixed hemangiomas have features of superficial and deep hemangiomas. Localized hemangiomas are round or oval and have well-defined margins. Segmental or patterned hemangiomas have less distinct borders and appear to grow in embryologic segments. Segmental hemangiomas can be associated with multiple syndromes.<br><br>Q3. With previous information, please write a paragraph to explain in detail the cause of this skin disease.<br><br>A3. IHs go through a characteristic growth pattern with rapid growth occurring most commonly in the first 6 months, although they can grow up to 12 months. The most rapid growth of IH is between 5.5 and 7.5 weeks of life (no matter what the age of gestation) and 90% are finished significantly growing by 4 months, although larger lesions can

and grow rapidly during the first several months. Most hemangiomas reach their maximum size by 6 to 9 months of age and then begin to involute, which is the process of shrinkage and involution. By the time a child reaches 5 years of age, most hemangiomas have completely involuted. Some hemangiomas may disappear on their own, while others may require medical intervention such as corticosteroids or surgery.<br><br>Q4. How should I treat it? What medicine should I use?<br><br>A4. The treatment of hemangiomas depends on the size, location, and severity of the lesion. Small hemangiomas that are not causing any symptoms or problems may not require treatment and can be observed for change. Larger or more complex hemangiomas may require medical intervention, such as corticosteroids, to help shrink the lesion and reduce symptoms.<br>Corticosteroids work by decreasing inflammation and suppressing the immune system, which can help to shrink the hemangioma. The most common side effects of corticosteroids are related to the skin, such as skin thinning, bruising, and increased susceptibility to infections.<br>Surgical excision is another treatment option for hemangiomas that are large, causing problems, or do not shrink on their own. Surgery involves removing the hemangioma and closing the wound with stitches. This method is mostly used for large and complex hemangiomas.<br>Laser therapy is also an option for treating hemangiomas. Laser therapy uses a beam of light to remove the hemangioma. This method is less invasive and has fewer side effects than surgery, but it may require multiple treatments.<br>It's important to consult a dermatologist for proper evaluation and treatment plan.	continue to grow up to a year of life and very rarely longer. Therefore therapy, when needed, is ideally started early in life to prevent the rapid growth that can lead to tissue distortion. IHs then regress and although the majority of the regression happens by age 4, continued improvement can occur up to and past age 10.<br><br>Q4. How should I treat it? What medicine should I use?<br><br>A4. Simple observation may be appropriate for many hemangiomas, allowing the lesions to regress spontaneously. Parents should be informed of the appropriate time frame and the pros and cons should be explained. For therapy, β-blockers are used most frequently, but systemic treatment can rarely be complicated by bradycardia or hypoglycemia, and regular feedings are critical before and during treatment. Topical timolol, although off label, is extremely effective for superficial hemangiomas and can help heal ulcerations. Oral prednisolone at a dose of 2–4 mg/kg/day has also been used for IH but has mostly been replaced by propranolol.
Evaluation of Physician	
Evaluation Item	Score (1-5, the higher the better)
SkinGPT-4's diagnosis is correct or relevant.	5
SkinGPT-4's description is informative.	5

SkinGPT-4's suggestions are useful.	4
SkinGPT-4 can help doctors with diagnosis.	5
SkinGPT-4 can help patients to understand their disease better.	4
If SkinGPT-4 can be deployed locally, it protects patients' privacy.	5
Willingness to use SkinGPT-4	5

Case Vascular Tumors 4

Image ID: Vascular tumor4	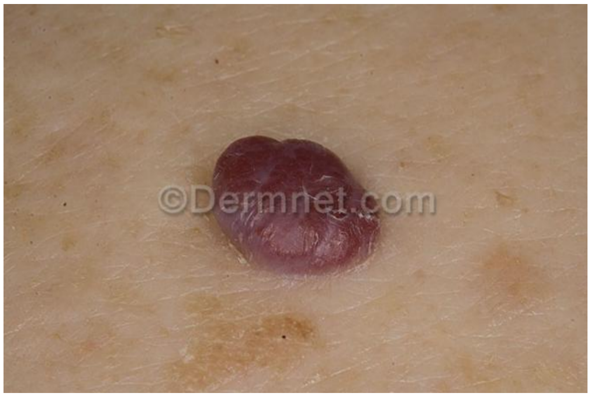
SkinGPT-4	Dermatology Physician
Q1. Could you describe the skin disease in this image for me?	Q1. Could you describe the skin disease in this image for me?
A1. This could be a skin disease image of hemangioma.	A1. This could be a skin disease image of hemangioma.
Q2. You should write a paragraph to list more features you observed in the image.	Q2. You should write a paragraph to list more features you observed in the image.
A2. The image shows a hemangioma, which is a benign tumor composed of blood vessels. It presents as a well defined, circular, raised lesion with smooth surfaces and a central depression. The color is cherry red, and it appears to be protruding from the skin. This type of hemangioma is also known as a strawberry hemangioma due to its red color. It can occur anywhere on the skin and is more common in infants and children. Hemangiomas are usually benign and do not require treatment unless they are causing symptoms or cosmetic concerns. They tend to involute over time and often leave a residual scar.	A2. IHs can present anywhere on the body and can be divided based on multiple features. Superficial IHs are easily seen on top of the skin and often have a bright red or maroon look when proliferating. Deep hemangiomas are under the skin and often have a blue hue but are soft. Mixed hemangiomas have features of superficial and deep hemangiomas. Localized hemangiomas are round or oval and have well-defined margins. Segmental or patterned hemangiomas have less distinct borders and appear to grow in embryologic segments. Segmental hemangiomas can be associated with multiple syndromes.
Q3. With previous information, please write a paragraph to explain in detail the cause of this skin disease.	Q3. With previous information, please write a paragraph to explain in detail the cause of this skin disease.
A3. Hemangiomas are caused by abnormal proliferation of endothelial cells, which line the blood vessels. These cells produce blood vessels that do not mature and infiltrate the surrounding tissue, leading to the formation of a hemangioma. The exact cause of hemangioma formation is not known, but it is thought to be related to genetic and environmental factors. They can occur	A3. IHs go through a characteristic growth pattern with rapid growth occurring most commonly in the first 6 months, although they can grow up to 12 months. The most rapid growth of IH is between 5.5 and 7.5 weeks of life (no matter what the age of gestation) and 90% are finished significantly growing by 4 months, although larger lesions can continue to grow up to a year of life and very rarely

sporadically or as part of a genetic syndrome, such as Hemangioma-Kostmann-Thompson syndrome. The development of a hemangioma is usually preceded by a period of rapid growth, known as the proliferative phase, followed by a period of slow growth or involution. During the involution phase, the hemangioma shrinks and becomes soft and flat, and often leaves a residual scar. In this image, the hemangioma appears to be protruding, which suggests that it is in the proliferative phase.<br><br>Q4. How should I treat it? What medicine should I use?<br><br>A4. The treatment of hemangiomas depends on the size, location, and severity of the lesion. Most hemangiomas do not require treatment unless they are causing symptoms or cosmetic concerns.<br><br>For small hemangiomas, observation and monitoring is the recommended treatment. This is because most hemangiomas will involute (shrink) over time and disappear on their own.<br>For larger or more symptomatic hemangiomas, medical treatment or surgery may be recommended. Medical treatment includes oral corticosteroids, which help to reduce inflammation and slow down the growth of the hemangioma. Surgery is also an option and involves excision of the hemangioma.<br>In the case of this image, it appears to be a small hemangioma on the skin, which is in the proliferative phase. The recommended treatment would be observation and monitoring, as it is likely to involute over time and disappear on its own. However, if the hemangioma causes any symptoms such as pain, itching or bleeding, medical treatment may be necessary.	longer. Therefore therapy, when needed, is ideally started early in life to prevent the rapid growth that can lead to tissue distortion. IHs then regress and although the majority of the regression happens by age 4, continued improvement can occur up to and past age 10.<br><br>Q4. How should I treat it? What medicine should I use?<br><br>A4. Simple observation may be appropriate for many hemangiomas, allowing the lesions to regress spontaneously. Parents should be informed of the appropriate time frame and the pros and cons should be explained. For therapy, β-blockers are used most frequently, but systemic treatment can rarely be complicated by bradycardia or hypoglycemia, and regular feedings are critical before and during treatment. Topical timolol, although off label, is extremely effective for superficial hemangiomas and can help heal ulcerations. Oral prednisolone at a dose of 2–4 mg/kg/day has also been used for IH but has mostly been replaced by propranolol.
Evaluation of Physician	
Evaluation Item	Score (1-5, the higher the better)
SkinGPT-4's diagnosis is correct or relevant.	5
SkinGPT-4's description is informative.	5
SkinGPT-4's suggestions are useful.	4

SkinGPT-4 can help doctors with diagnosis.	5
SkinGPT-4 can help patients to understand their disease better.	4
If SkinGPT-4 can be deployed locally, it protects patients' privacy.	5
Willingness to use SkinGPT-4	5

Case Vascular Tumors 5

Image ID: Vascular tumor5	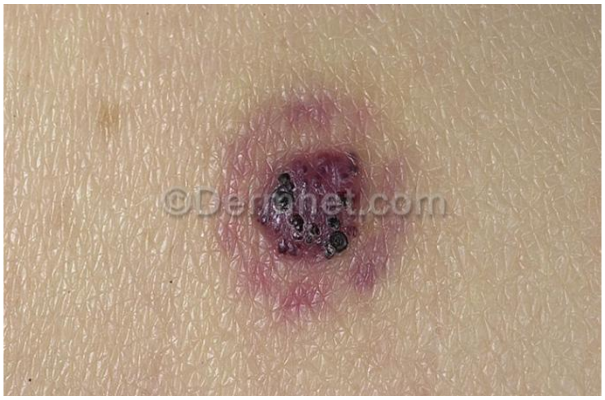
SkinGPT-4	Dermatology Physician
Q1. Could you describe the skin disease in this image for me?<br><br>A1. This could be a skin disease image of hemangioma.<br><br>Q2. You should write a paragraph to list more features you observed in the image.<br><br>A2. The image shows a hemangioma, which is a benign tumor composed of blood vessels. It presents as a raised, smooth, round or oval-shaped mass with a central ulceration. It can be found on the skin or mucosa and can be congenital or acquired. Hemangiomas can be found on any part of the body, but most commonly on the head and neck. They can be treated with various methods, such as surgery, cryotherapy, or laser therapy. This image shows a hemangioma on the skin.<br><br>Q3. With previous information, please write a paragraph to explain in detail the cause of this skin disease.<br><br>A3. The cause of hemangioma is not entirely understood, but it is believed to be related to abnormal proliferation of endothelial cells, which are responsible for forming blood vessels. Hemangiomas are most commonly seen in infancy and childhood, and they tend to grow rapidly in the first few months of life. After reaching their maximum size, they often begin to regress and can disappear completely by adulthood. Some hemangiomas can be treated with propranolol, a	Q1. Could you describe the skin disease in this image for me?<br><br>A1. This could be a skin disease image of hemangioma.<br><br>Q2. You should write a paragraph to list more features you observed in the image.<br><br>A2. The image shows a hemangioma, which is a benign tumor composed of blood vessels. It presents as a well defined, circular, raised lesion with smooth surfaces and a central depression. The color is cherry red, and it appears to be protruding from the skin. This type of hemangioma is also known as a strawberry hemangioma due to its red color. It can occur anywhere on the skin and is more common in infants and children. Hemangiomas are usually benign and do not require treatment unless they are causing symptoms or cosmetic concerns. They tend to involute over time and often leave a residual scar.<br><br>Q3. With previous information, please write a paragraph to explain in detail the cause of this skin disease.<br><br>A3. Hemangiomas are caused by abnormal proliferation of endothelial cells, which line the blood vessels. These cells produce blood vessels that do not mature and infiltrate the surrounding tissue, leading to the formation of a hemangioma. The exact cause of hemangioma formation is not known, but it is thought to be related to genetic and environmental factors. They can occur

beta-blocker that helps to slow down the growth of hemangiomas. In this image, the hemangioma is present on the skin and is likely to be congenital or acquired.<br><br>Q4. How should I treat it? What medicine should I use?<br><br>A4. Treatment for hemangioma depends on the size, location, and severity of the lesion. Small hemangiomas may not require any treatment, while larger or more symptomatic lesions can be treated with various options such as:<br>1. Surgery: Surgical excision is the most common treatment for hemangiomas that are large, causing symptoms, or located in a cosmetically sensitive area.<br>2. Cryotherapy: Cryotherapy involves freezing the hemangioma with liquid nitrogen, which causes the lesion to shrink and fall off over time.<br>3. Laser therapy: Laser therapy uses intense light to destroy the blood vessels in the hemangioma, causing it to shrink and fade over time.<br>4. Medications: Propranolol and timolol are beta-blockers that can be used to slow down the growth of hemangiomas. They are usually prescribed for infants and young children.<br>The most appropriate treatment option depends on the size, location, and severity of the hemangioma, as well as the patient's age and overall health. Your dermatologist or pediatrician can provide the best advice on the treatment options.	sporadically or as part of a genetic syndrome, such as Hemangioma-Kostmann-Thompson syndrome. The development of a hemangioma is usually preceded by a period of rapid growth, known as the proliferative phase, followed by a period of slow growth or involution. During the involution phase, the hemangioma shrinks and becomes soft and flat, and often leaves a residual scar. In this image, the hemangioma appears to be protruding, which suggests that it is in the proliferative phase.<br><br>Q4. How should I treat it? What medicine should I use?<br><br>A4. The treatment of hemangiomas depends on the size, location, and severity of the lesion. Most hemangiomas do not require treatment unless they are causing symptoms or cosmetic concerns.<br>For small hemangiomas, observation and monitoring is the recommended treatment. This is because most hemangiomas will involute (shrink) over time and disappear on their own.<br>For larger or more symptomatic hemangiomas, medical treatment or surgery may be recommended. Medical treatment includes oral corticosteroids, which help to reduce inflammation and slow down the growth of the hemangioma. Surgery is also an option and involves excision of the hemangioma.<br>In the case of this image, it appears to be a small hemangioma on the skin, which is in the proliferative phase. The recommended treatment would be observation and monitoring, as it is likely to involute over time and disappear on its own. However, if the hemangioma causes any symptoms such as pain, itching or bleeding, medical treatment may be necessary.

Evaluation of Physician

Evaluation Item	Score (1-5, the higher the better)
SkinGPT-4's diagnosis is correct or relevant.	5
SkinGPT-4's description is informative.	4

SkinGPT-4's suggestions are useful.	4
SkinGPT-4 can help doctors with diagnosis.	5
SkinGPT-4 can help patients to understand their disease better.	4
If SkinGPT-4 can be deployed locally, it protects patients' privacy.	5
Willingness to use SkinGPT-4	5

Case Vascular Tumors 6

Image ID: Vascular tumor6	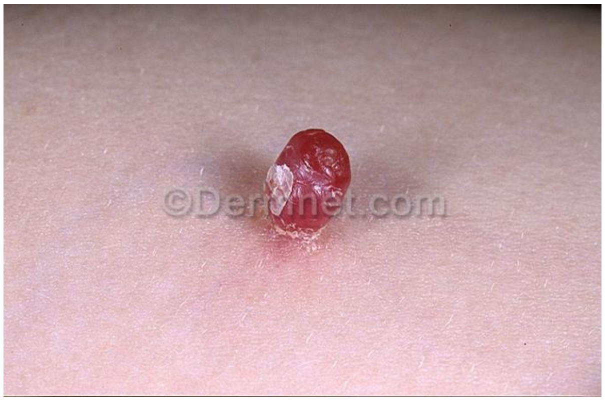
SkinGPT-4	Dermatology Physician
Q1. Could you describe the skin disease in this image for me?<br><br>A1. This could be a skin disease image of pyogenic granuloma.<br><br>Q2. You should write a paragraph to list more features you observed in the image.<br><br>A2. The image shows a hemangioma, which is a benign tumor composed of blood vessels. It is observed on the skin and can be either congenital or acquired. The hemangioma in the image is a pyogenic granuloma, which is a type of hemangioma that appears as a smooth, bright red, and shiny bump on the skin. It is also known as a granulation tissue and can be caused by trauma, surgery, or infection. The lesion is composed of a central vein with a rim of granulation tissue, which is made up of young, actively dividing capillaries. The lesion is highly vascular and may bleed if traumatized. Pyogenic granuloma is a benign tumor that can be treated with intralesional steroids, curettage, or surgical excision.<br><br>Q3. With previous information, please write a paragraph to explain in detail the cause of this skin disease.<br><br>A3. Pyogenic granuloma, the lesion shown in the image, is caused by trauma or irritation to the skin, which triggers the body's immune response to form a granuloma. A granuloma is a type of inflammatory response that leads to the formation	Q1. Could you describe the skin disease in this image for me?<br><br>A1. This could be a skin disease image of pyogenic granuloma.<br><br>Q2. You should write a paragraph to list more features you observed in the image.<br><br>A2. A pyogenic granuloma is a small, eruptive, usually solitary, sessile or pedunculated, friable papule . The lesion is common in children but may occur at any age, especially during pregnancy or with medication use. Pyogenic granuloma occurs most often on an exposed surface: on the hands, forearms, or face, or at sites of trauma. The lesions can also occur in the mouth, especially on the gingiva, most often in pregnant women (granuloma gravidarum). On the sole of the foot or nail bed, it may be mistaken for a melanoma. Pyogenic granulomas bleed easily on the slightest trauma and, if cut off superficially, promptly recur.<br><br>Q3. With previous information, please write a paragraph to explain in detail the cause of this skin disease.<br><br>A3. Pyogenic granulomas bleed easily on the slightest trauma and, if cut off superficially, promptly recur.Recurring lesions may have one or many satellite lesions.Pyogenic granulomas may be seen in patients treated with isotretinoin, capecitabine, vemurafenib, or indinavir. With time, the epidermis becomes thinned, then eroded. Heavy secondary staphylococcal colonization is

of a small, localized nodule. Pyogenic granuloma is caused by a specific type of immune response, known as a Th2 response, which leads to the formation of granulation tissue. This lesion is also known as a “pyo” because of its tendency to bleed, and “granuloma” because of its appearance. The lesion is made up of a central vein with a rim of granulation tissue, which is composed of young, actively dividing capillaries. The cause of this skin disease is not known, but it can be triggered by factors such as insect bites, skin injury, or even psychological stress. Pyogenic granuloma is more common in women and people with fair skin, and it can be treated with intralesional steroids, curettage, or surgical excision.<br><br>Q4. How should I treat it? What medicine should I use?<br><br>A4. Treatment for pyogenic granuloma usually involves intralesional steroids, curettage, or surgical excision. Intralesional steroids involve injecting steroids directly into the lesion to reduce inflammation and slow down the growth of the granuloma. Curettage is a procedure where the lesion is scraped away with a curette, and then treated with steroids. Surgical excision is the removal of the lesion through surgery.<br><br>It is important to consult a dermatologist for the correct diagnosis and treatment plan. The dermatologist will evaluate the lesion and recommend the best treatment based on the size, location, and severity of the pyogenic granuloma.	common. Intravascular pyogenic granuloma appears as a lobular capillary proliferation within a vein.<br><br>Q4. How should I treat it? What medicine should I use?<br><br>A4. Treatment is by curettage or shave excision, followed by destruction of the base by fulguration or aluminum chloride. Silver nitrate alone may be sufficient to treat smaller lesions but may leave a silver tattoo, and it does not allow histopathologic evaluation to rule out a bleeding amelanotic lesion such as a melanoma. Topical timolol, imiquimod under occlusion, and sclerotherapy with monoethanolamine oleate or sodium tetradecyl sulfate have been used successfully. At times, a recalcitrant lesion may require excision or laser ablation. The drug-induced variety will regress after lowering of the dose or discontinuation of the medication. Systemic corticosteroids have been used to treat recurrent giant pyogenic granulomas.
Evaluation of Physician	
Evaluation Item	Score (1-5, the higher the better)
SkinGPT-4’s diagnosis is correct or relevant.	5
SkinGPT-4’s description is informative.	5
SkinGPT-4’s suggestions are useful.	4
SkinGPT-4 can help doctors with diagnosis.	5

SkinGPT-4 can help patients to understand their disease better.	5
If SkinGPT-4 can be deployed locally, it protects patients' privacy.	5
Willingness to use SkinGPT-4	5

Case Vascular Tumors 7

Image ID: Vascular tumor7	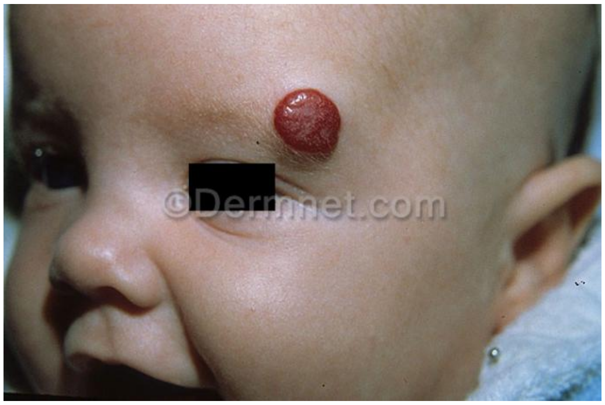
SkinGPT-4	Dermatology Physician
Q1. Could you describe the skin disease in this image for me?<br><br>A1. This could be a skin disease image of hemangioma infancy.<br><br>Q2. You should write a paragraph to list more features you observed in the image.<br><br>A2. The image appears to be a birthmark hemangioma. It is congenital and benign, and commonly present on the head and neck in the first few months of life. It appears as a rubbery, soft, raised, vascular nodule that may be ulcerated or non ulcerated. The color varies from deep red to purple, and it may be accompanied by a stork mark (a paler area surrounding the hemangioma). Hemangiomas are the most common tumors of infancy and almost all of them will involute by 5 years of age.<br><br>Q3. With previous information, please write a paragraph to explain in detail the cause of this skin disease.<br><br>A3. Hemangiomas are caused by abnormal proliferation of endothelial cells, which form blood vessels. They are believed to arise from pluripotent stem cells that differentiate into endothelial cells. The exact etiology of hemangiomas is not known, but they are thought to have a genetic component as they occur more frequently in families with a history of hemangiomas. The development of hemangiomas	Q1. Could you describe the skin disease in this image for me?<br><br>A1. This could be a skin disease image of hemangioma.<br><br>Q2. You should write a paragraph to list more features you observed in the image.<br><br>A2. IHs can present anywhere on the body and can be divided based on multiple features. Superficial IHs are easily seen on top of the skin and often have a bright red or maroon look when proliferating. Deep hemangiomas are under the skin and often have a blue hue but are soft. Mixed hemangiomas have features of superficial and deep hemangiomas. Localized hemangiomas are round or oval and have well-defined margins. Segmental or patterned hemangiomas have less distinct borders and appear to grow in embryologic segments. Segmental hemangiomas can be associated with multiple syndromes.<br><br>Q3. With previous information, please write a paragraph to explain in detail the cause of this skin disease.<br><br>A3. IHs go through a characteristic growth pattern with rapid growth occurring most commonly in the first 6 months, although they can grow up to 12 months. The most rapid growth of IH is between 5.5 and 7.5 weeks of life (no matter what the age of gestation) and 90% are finished significantly growing by 4 months, although larger lesions can continue to grow up to a year of life and very rarely

is regulated by the Sonic Hedgehog signaling pathway, which is a key player in the development of blood vessels. The growth of hemangiomas is rapid during the first few months of life, and they can reach a size of up to 2 cm in diameter. After the age of 2 to 3 months, the growth rate decreases, and by the time the child reaches 5 years of age, the hemangioma has regressed. Most hemangiomas disappear without any treatment, but some may require treatment if they cause complications such as ulceration, bleeding, or scarring.<br><br>Q4. How should I treat it? What medicine should I use?<br><br>A4. Hemangiomas are caused by abnormal proliferation of endothelial cells, which form blood vessels. They are believed to arise from pluripotent stem cells that differentiate into endothelial cells. The exact etiology of hemangiomas is not known, but they are thought to have a genetic component as they occur more frequently in families with a history of hemangiomas. The development of hemangiomas is regulated by the Sonic Hedgehog signaling pathway, which is a key player in the development of blood vessels. The growth of hemangiomas is rapid during the first few months of life, and they can reach a size of up to 2 cm in diameter. After the age of 2 to 3 months, the growth rate decreases, and by the time the child reaches 5 years of age, the hemangioma has regressed. Most hemangiomas disappear without any treatment, but some may require treatment if they cause complications such as ulceration, bleeding, or scarring.<br>How should I treat it? What medicine should I use?<br>The treatment of hemangiomas depends on the size, location, and severity of the lesion. Most hemangiomas do not require treatment and will regress on their own over time. However, if the hemangioma is causing problems such as bleeding, ulceration, or scarring, or if it is causing functional problems such as difficulty feeding or breathing, then treatment may be necessary.<br>The most common treatment for hemangiomas is propranolol, a beta-blocker that decreases blood flow to the hemangioma, causing it to shrink. This medication is usually prescribed as a cream or gel that is applied directly to the hemangioma. Oral	longer. Therefore therapy, when needed, is ideally started early in life to prevent the rapid growth that can lead to tissue distortion. IHs then regress and although the majority of the regression happens by age 4, continued improvement can occur up to and past age 10.<br><br>Q4. How should I treat it? What medicine should I use?<br><br>A4. Simple observation may be appropriate for many hemangiomas, allowing the lesions to regress spontaneously. Parents should be informed of the appropriate time frame and the pros and cons should be explained. For therapy, β-blockers are used most frequently, but systemic treatment can rarely be complicated by bradycardia or hypoglycemia, and regular feedings are critical before and during treatment. Topical timolol, although off label, is extremely effective for superficial hemangiomas and can help heal ulcerations. Oral prednisolone at a dose of 2–4 mg/kg/day has also been used for IH but has mostly been replaced by propranolol.

medication is not recommended as it can cause serious side effects such as bradycardia and hypotension.<br>Another option for treating hemangiomas is corticosteroids, which can be administered as a cream, ointment, or intralesional injection. Corticosteroids work by decreasing inflammation and reducing the growth of blood vessels in the hemangioma.<br><br>Surgery is a last resort for treating hemangiomas and is usually reserved for lesions that are causing significant functional problems or are cosmetically disfiguring.	
Evaluation of Physician	
Evaluation Item	Score (1-5, the higher the better)
SkinGPT-4's diagnosis is correct or relevant.	5
SkinGPT-4's description is informative.	5
SkinGPT-4's suggestions are useful.	4
SkinGPT-4 can help doctors with diagnosis.	5
SkinGPT-4 can help patients to understand their disease better.	5
If SkinGPT-4 can be deployed locally, it protects patients' privacy.	5
Willingness to use SkinGPT-4	5

Case Vascular Tumors 8

Image ID: Vascular tumor8	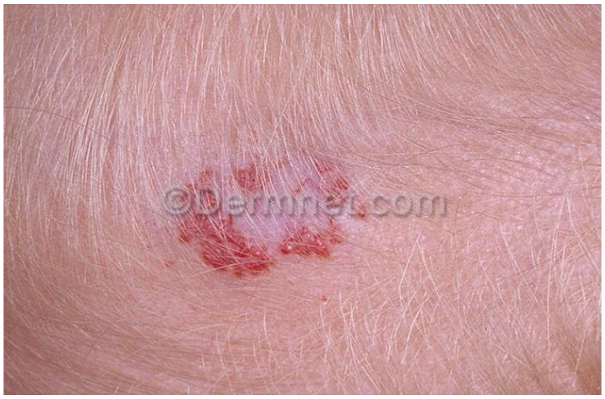
**SkinGPT-4**	**Dermatology Physician**
Q1. Could you describe the skin disease in this image for me?<br><br>A1. This could be a skin disease image of basal cell carcinoma lesion.<br><br>Q2. You should write a paragraph to list more features you observed in the image.<br><br>A2. The lesion appears as a scaly, erythematous plaque with a central ulceration. The borders are indistinct and the lesion is shiny. The skin surrounding the lesion appears normal. No pigmentation or scarring is present. There is no regional lymph node involvement. The size of the lesion is 3 cm in diameter. The diagnosis could be a squamous cell carcinoma.<br><br>Q3. With previous information, please write a paragraph to explain in detail the cause of this skin disease.<br><br>A3. The cause of this skin disease is mostly attributed to chronic sun exposure, as well as other risk factors such as fair skin, blue eyes, and a history of sunburns. The ultraviolet (UV) radiation from the sun damages the skin cells and causes mutations in the DNA, leading to the development of skin cancer. The lesion is a squamous cell carcinoma, which is the second most common form of skin cancer, characterized by the growth of abnormal cells in the squamous cells of the skin. The risk of developing squamous cell carcinoma increases with prolonged sun exposure, especially in individuals with a history of	Q1. Could you describe the skin disease in this image for me?<br><br>A1. This could be a skin disease image of hemangioma.<br><br>Q2. You should write a paragraph to list more features you observed in the image.<br><br>A2. IHs can present anywhere on the body and can be divided based on multiple features. Superficial IHs are easily seen on top of the skin and often have a bright red or maroon look when proliferating. Deep hemangiomas are under the skin and often have a blue hue but are soft. Mixed hemangiomas have features of superficial and deep hemangiomas. Localized hemangiomas are round or oval and have well-defined margins. Segmental or patterned hemangiomas have less distinct borders and appear to grow in embryologic segments. Segmental hemangiomas can be associated with multiple syndromes.<br><br>Q3. With previous information, please write a paragraph to explain in detail the cause of this skin disease.<br><br>A3. IHs go through a characteristic growth pattern with rapid growth occurring most commonly in the first 6 months, although they can grow up to 12 months. The most rapid growth of IH is between 5.5 and 7.5 weeks of life (no matter what the age of gestation) and 90% are finished significantly growing by 4 months, although larger lesions can continue to grow up to a year of life and very rarely

sunburns and fair skin. It is important to avoid prolonged sun exposure and use sun protection measures to prevent the development of skin cancer. Regular skin examinations and early detection can help in the effective treatment of squamous cell carcinoma.<br><br>Q4. How should I treat it? What medicine should I use?<br><br>A4. The treatment of squamous cell carcinoma will depend on the size, location, and stage of the cancer. The most common treatment options for squamous cell carcinoma are surgery, radiation therapy, and chemotherapy. The surgery may involve a wide local excision or Mohs surgery to remove the cancerous lesion, along with some healthy skin surrounding it. Radiation therapy involves the use of high-energy X-rays to destroy the cancer cells. Chemotherapy may be used as a primary treatment or in combination with surgery or radiation therapy to kill the cancer cells.<br>It is important to consult a dermatologist or a medical oncologist to determine the best treatment plan for your specific case, as they will evaluate the cancer size, location, and stage, as well as your overall health and preferences. They will also provide guidance on the appropriate medicine to use.	longer. Therefore therapy, when needed, is ideally started early in life to prevent the rapid growth that can lead to tissue distortion. IHs then regress and although the majority of the regression happens by age 4, continued improvement can occur up to and past age 10.<br><br>Q4. How should I treat it? What medicine should I use?<br><br>A4. Simple observation may be appropriate for many hemangiomas, allowing the lesions to regress spontaneously. Parents should be informed of the appropriate time frame and the pros and cons should be explained. For therapy, β-blockers are used most frequently, but systemic treatment can rarely be complicated by bradycardia or hypoglycemia, and regular feedings are critical before and during treatment. Topical timolol, although off label, is extremely effective for superficial hemangiomas and can help heal ulcerations. Oral prednisolone at a dose of 2–4 mg/kg/day has also been used for IH but has mostly been replaced by propranolol.
Evaluation of Physician	
Evaluation Item	Score (1-5, the higher the better)
SkinGPT-4's diagnosis is correct or relevant.	1
SkinGPT-4's description is informative.	5
SkinGPT-4's suggestions are useful.	5
SkinGPT-4 can help doctors with diagnosis.	1
SkinGPT-4 can help patients to understand their disease better.	1
If SkinGPT-4 can be deployed locally, it protects patients' privacy.	3

Willingness to use SkinGPT-4	3

Case Vascular Tumors 9

Image ID: Vascular tumor9	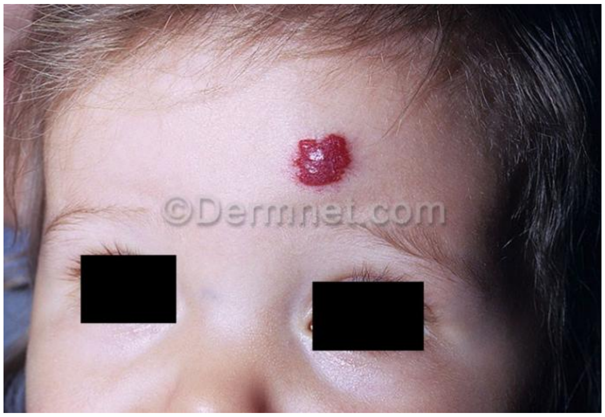
**SkinGPT-4**	**Dermatology Physician**
Q1. Could you describe the skin disease in this image for me?<br><br>A1. This could be a skin disease image of hemangioma infancy.<br><br>Q2. You should write a paragraph to list more features you observed in the image.<br><br>A2. The image provided is a photograph of a hemangioma in an infant. It shows a well circumscribed, superficial hemangioma located on the face. The lesion is composed of a nodular protrusion with a reddish color and a smooth surface. It is causing distress to the infant.<br><br>Q3. With previous information, please write a paragraph to explain in detail the cause of this skin disease.<br><br>A3. The cause of hemangioma in infancy is not fully understood. However, it is believed to be caused by abnormal proliferation of endothelial cells, which form blood vessels. Hemangiomas are benign tumors that can occur anywhere on the body, but they are most commonly found on the head and neck. They tend to grow rapidly during the first few months of life and then involute (get smaller) over time. Most hemangiomas disappear completely by the time the child reaches adulthood, but some may leave residual marks such as redness, pigmentation, or scars. The majority of hemangiomas are asymptomatic and do not require treatment. However, large or	Q1. Could you describe the skin disease in this image for me?<br><br>A1. This could be a skin disease image of hemangioma.<br><br>Q2. You should write a paragraph to list more features you observed in the image.<br><br>A2. IHs can present anywhere on the body and can be divided based on multiple features. Superficial IHs are easily seen on top of the skin and often have a bright red or maroon look when proliferating. Deep hemangiomas are under the skin and often have a blue hue but are soft. Mixed hemangiomas have features of superficial and deep hemangiomas. Localized hemangiomas are round or oval and have well-defined margins. Segmental or patterned hemangiomas have less distinct borders and appear to grow in embryologic segments. Segmental hemangiomas can be associated with multiple syndromes.<br><br>Q3. With previous information, please write a paragraph to explain in detail the cause of this skin disease.<br><br>A3. IHs go through a characteristic growth pattern with rapid growth occurring most commonly in the first 6 months, although they can grow up to 12 months. The most rapid growth of IH is between 5.5 and 7.5 weeks of life (no matter what the age of gestation) and 90% are finished significantly growing by 4 months, although larger lesions can

symptomatic hemangiomas may require medical intervention.<br><br>Q4. How should I treat it? What medicine should I use?<br><br>A4. The treatment of hemangiomas in infancy depends on the size, location, and severity of the lesion. Small hemangiomas that are asymptomatic may not require any treatment, and the lesion will usually disappear on its own within a few years. However, larger or more symptomatic hemangiomas may require medical intervention.<br>The most common treatment for hemangiomas is propranolol, a beta-blocker medication that helps to reduce the blood flow to the hemangioma. This can cause the hemangioma to shrink and become less noticeable. Propranolol is typically given orally and the dosage is adjusted based on the body weight of the child. The treatment usually lasts for several months to a year or more, depending on the size and location of the hemangioma.<br>Another option for treatment is corticosteroids, which can help to reduce inflammation and slow down the growth of the hemangioma. Corticosteroids can be administered topically or systemically, depending on the size and location of the hemangioma.<br>It's important to note that hemangiomas are benign tumors and they will not turn into cancer. However, large hemangiomas may cause complications such as bleeding, ulceration, or obstruction of the airway.	continue to grow up to a year of life and very rarely longer. Therefore therapy, when needed, is ideally started early in life to prevent the rapid growth that can lead to tissue distortion. IHs then regress and although the majority of the regression happens by age 4, continued improvement can occur up to and past age 10.<br><br>Q4. How should I treat it? What medicine should I use?<br><br>A4. Simple observation may be appropriate for many hemangiomas, allowing the lesions to regress spontaneously. Parents should be informed of the appropriate time frame and the pros and cons should be explained. For therapy, β-blockers are used most frequently, but systemic treatment can rarely be complicated by bradycardia or hypoglycemia, and regular feedings are critical before and during treatment. Topical timolol, although off label, is extremely effective for superficial hemangiomas and can help heal ulcerations. Oral prednisolone at a dose of 2–4 mg/kg/day has also been used for IH but has mostly been replaced by propranolol.

Evaluation of Physician

Evaluation Item	Score (1-5, the higher the better)
SkinGPT-4's diagnosis is correct or relevant.	5
SkinGPT-4's description is informative.	5
SkinGPT-4's suggestions are useful.	5
SkinGPT-4 can help doctors with diagnosis.	5
SkinGPT-4 can help patients to understand their disease better.	5

If SkinGPT-4 can be deployed locally, it protects patients’ privacy.	5
Willingness to use SkinGPT-4	5

Case Vascular Tumors 10

<table>
<tr><td>Image ID: Vascular tumor10</td><td>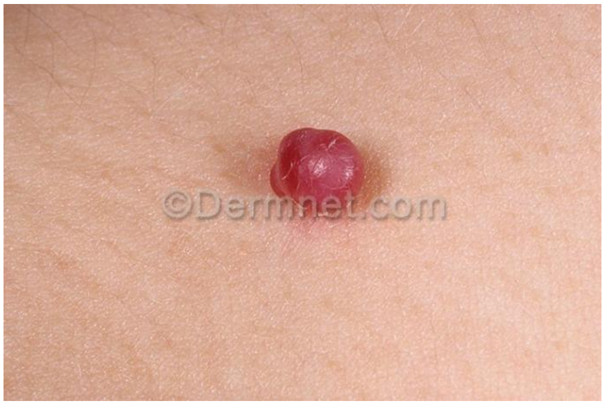
</td></tr>
<tr><th>SkinGPT-4</th><th>Dermatology Physician</th></tr>
<tr><td>Q1. Could you describe the skin disease in this image for me?

A1. This could be a skin disease image of pyogenic granuloma.

Q2. You should write a paragraph to list more features you observed in the image.

A2. This image shows a skin lesion on the surface of the skin. The lesion has a reddish color and the shape of a button. It is circular in shape with a smooth surface. The lesion is raised above the surrounding skin and has a distinct border. The lesion is flat and has no scaliness, bleeding or crusting. The color of the lesion is not white or yellow.

Q3. With previous information, please write a paragraph to explain in detail the cause of this skin disease.

A3. The cause of this skin disease is not clear. However, it is believed to be a form of pyogenic granuloma, which is a benign tumor of the skin that usually arises from the dermis. The exact cause of pyogenic granuloma is not known, but it is thought to be triggered by an injury to the skin, such as trauma, burns, or surgery. Pyogenic granuloma is more common in women and those with darker skin types. It is often seen on the face, neck, and arms. Pyogenic granuloma is characterized by a reddish-purple, dome-shaped nodule with a smooth surface. The lesion is usually solitary, but multiple lesions can occur.</td><td>Q1. Could you describe the skin disease in this image for me?

A1. This could be a skin disease image of pyogenic granuloma.

Q2. You should write a paragraph to list more features you observed in the image.

A2. A pyogenic granuloma is a small, eruptive, usually solitary, sessile or pedunculated, friable papule . The lesion is common in children but may occur at any age, especially during pregnancy or with medication use. Pyogenic granuloma occurs most often on an exposed surface: on the hands, forearms, or face, or at sites of trauma. The lesions can also occur in the mouth, especially on the gingiva, most often in pregnant women (granuloma gravidarum). On the sole of the foot or nail bed, it may be mistaken for a melanoma. Pyogenic granulomas bleed easily on the slightest trauma and, if cut off superficially, promptly recur.

Q3. With previous information, please write a paragraph to explain in detail the cause of this skin disease.

A3. Pyogenic granulomas bleed easily on the slightest trauma and, if cut off superficially, promptly recur.Recurring lesions may have one or many satellite lesions.Pyogenic granulomas may be seen in patients treated with isotretinoin, capecitabine, vemurafenib, or indinavir. With time, the epidermis becomes thinned, then eroded. Heavy secondary staphylococcal colonization is</td></tr>
</table>

The lesion tends to bleed easily and can be tender to the touch. Pyogenic granuloma is a benign tumor and does not spread to other parts of the body. Treatment options include surgical excision, curettage, and cryotherapy.<br><br>Q4. How should I treat it? What medicine should I use?<br><br>A4. The treatment of pyogenic granuloma depends on the size and location of the lesion. Small lesions can be treated with cryotherapy or curettage, while larger lesions may require surgical excision. The choice of treatment depends on the patient's skin type, the location of the lesion, and the patient's desire for scarless healing. Some doctors may also prescribe medication to help reduce the size of the lesion before treatment. The medication used is usually a topical cream or ointment containing a corticosteroid, which helps to reduce inflammation and shrink the lesion. However, it is important to consult a dermatologist for proper diagnosis and treatment.	common. Intravascular pyogenic granuloma appears as a lobular capillary proliferation within a vein.<br><br>Q4. How should I treat it? What medicine should I use?<br><br>A4. Treatment is by curettage or shave excision, followed by destruction of the base by fulguration or aluminum chloride. Silver nitrate alone may be sufficient to treat smaller lesions but may leave a silver tattoo, and it does not allow histopathologic evaluation to rule out a bleeding amelanotic lesion such as a melanoma. Topical timolol, imiquimod under occlusion, and sclerotherapy with monoethanolamine oleate or sodium tetradecyl sulfate have been used successfully. At times, a recalcitrant lesion may require excision or laser ablation. The drug-induced variety will regress after lowering of the dose or discontinuation of the medication. Systemic corticosteroids have been used to treat recurrent giant pyogenic granulomas.

Evaluation of Physician

Evaluation Item	Score (1-5, the higher the better)
SkinGPT-4's diagnosis is correct or relevant.	5
SkinGPT-4's description is informative.	5
SkinGPT-4's suggestions are useful.	4
SkinGPT-4 can help doctors with diagnosis.	5
SkinGPT-4 can help patients to understand their disease better.	5
If SkinGPT-4 can be deployed locally, it protects patients' privacy.	5
Willingness to use SkinGPT-4	5

Case Herpes 1

Image ID: Herpes1	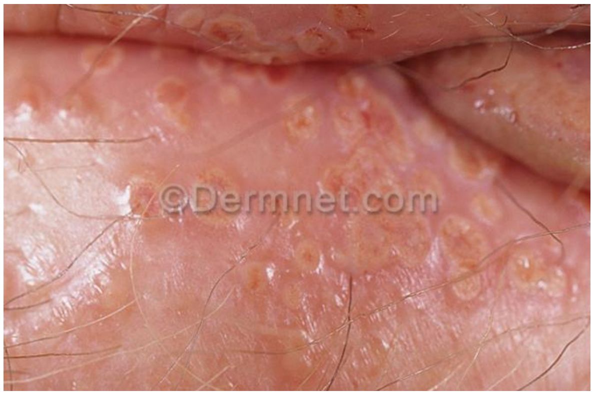
SkinGPT-4	Dermatology Physician
Q1. Could you describe the skin disease in this image for me?<br><br>A1. This could be a skin disease image of herpes type recurrent.<br><br>Q2. You should write a paragraph to list more features you observed in the image.<br><br>A2. This image shows a skin lesion, which could be a herpes simplex type recurrent. The lesion is a fluid filled blister with clear content, which is characteristic of herpes simplex virus infection. The lesion is located on the lip, which is a common site for herpes simplex virus infection. The lesion is round in shape, with a clear fluid content and a white skin border. The border is well defined and smooth. The surrounding skin appears normal.<br><br>Q3. With previous information, please write a paragraph to explain in detail the cause of this skin disease.<br><br>A3. This skin disease is caused by the herpes simplex virus (HSV). HSV is a double stranded DNA virus that belongs to the family Herpesviridae. There are two main types of HSV: type 1 and type 2. Type 1 is the most common cause of herpes simplex disease, while type 2 is more commonly associated with genital herpes. HSV infects the cells of the skin or mucosa and replicates in them, leading to the formation of blisters. The blisters are filled with clear fluid and typically ulcerate, resulting in painful sores. The	Q1. Could you describe the skin disease in this image for me?<br><br>A1. This could be a skin disease image of herpes simplex.<br><br>Q2. You should write a paragraph to list more features you observed in the image.<br><br>A2. The clinical manifestations of HSV infection depend on the site of infection and the immune status of the host. These infections are associated with extensive genital lesions in different stages of evolution, including vesicles, pustules, and erythematous ulcers that may require 2–3 weeks to resolve.<br><br>Q3. With previous information, please write a paragraph to explain in detail the cause of this skin disease.<br><br>A3. HSV-1 and HSV-2 are members of the Herpesviridae family, a group of lipid-enveloped double-stranded DNA viruses. In vivo, HSV infections can be divided into three stages: ① acute infection, ② establishment and maintenance of the latency, and ③ reactivation of virus. Host immunity to HSV influences the risk of acquiring the infection, the severity of disease, and the frequency of recurrences.<br><br>Q4. How should I treat it? What medicine should I use?

virus is highly contagious and can be transmitted through direct contact with the skin lesion or body fluids of an infected individual. Once a person is infected with HSV, the virus remains in the body and can reactivate periodically, leading to recurrent herpes simplex infections. HSV infection can also lead to complications such as neonatal herpes, herpes glossitis, herpes zoster, and herpes encephalitis in immunocompromised individuals.<br><br>Q4. How should I treat it? What medicine should I use?<br><br>A4. The treatment of herpes simplex infection depends on the severity and location of the infection. For localized herpes simplex infections, topical antiviral medications such as acyclovir or penciclovir can be used to help speed up healing and reduce symptoms. These medications work by inhibiting the replication of the virus, thus reducing the duration and severity of the infection.	A4. Many HSV infections require no specific treatment. Keeping the lesions clean and dry while they heal by themselves may be all that is required. Treatment is warranted for infections that are likely to prove protracted, highly symptomatic, or complicated. Acyclovir has a highly favorable therapeutic index because of its preferential activation in infected cells and preferential inhibition of the viral DNA polymerase. Valacyclovir, is an oral prodrug of acyclovir that achieves higher bioavailability after oral administration, thus can be used in a more convenient dosage regimen. Famciclovir is the well-absorbed oral form of the related guanosineanalogue penciclovir.
Evaluation of Physician	
Evaluation Item	Score (1-5, the higher the better)
SkinGPT-4's diagnosis is correct or relevant.	5
SkinGPT-4's description is informative.	4
SkinGPT-4's suggestions are useful.	5
SkinGPT-4 can help doctors with diagnosis.	5
SkinGPT-4 can help patients to understand their disease better.	5
If SkinGPT-4 can be deployed locally, it protects patients' privacy.	5
Willingness to use SkinGPT-4	5

Case Herpes 2

Image ID: Herpes2	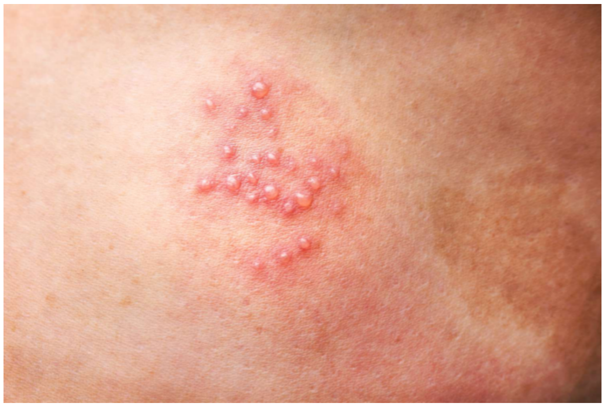
SkinGPT-4	Dermatology Physician
Q1. Could you describe the skin disease in this image for me?<br><br>A1. This could be a skin disease image of herpes zoster.<br><br>Q2. You should write a paragraph to list more features you observed in the image.<br><br>A2. In this image, there is a small herpes zoster (HZ) eruption on the left side of the face, with a single blister on the temple area. The eruption is oval in shape and has clear fluid in the center. The surrounding skin is erythematous and there is mild desquamation. The lesion is slightly raised and has a slightly punched out center. The border is not well defined and the color is white. The surrounding skin is healthy looking. The lesion is asymptomatic and there is no regional lymph node enlargement. There is no evidence of skin atrophy or telangiectasias. The eruption is not itchy or pruritic. The patient has no history of previous herpes zoster infection or immunosuppression. The patient is not currently taking any medications that could be causative.<br><br>Q3. With previous information, please write a paragraph to explain in detail the cause of this skin disease.<br><br>A3. The cause of this skin disease is herpes zoster, also known as shingles. Herpes zoster is a viral infection caused by the varicella-zoster virus (VZV), which also causes chickenpox. After a person recovers from chickenpox, the virus	Q1. Could you describe the skin disease in this image for me?<br><br>A1. This could be a skin disease image of herpes zoster.<br><br>Q2. You should write a paragraph to list more features you observed in the image.<br><br>A2. The most distinctive feature of herpes zoster is the localization and distribution of the rash, which is nearly always unilateral and is generally limited to the area of skin innervated by a single sensory ganglion. Herpes zoster lesions begin as erythematous macules and papules. Vesicles form within 12–24 hours and evolve into pustules by the third day. These dry and crust in 7–10 days. The crusts generally persist for 2–3 weeks. In normal individuals, new lesions continue to appear for 1–4 days (occasionally for as long as 7 days).<br><br>Q3. With previous information, please write a paragraph to explain in detail the cause of this skin disease.<br><br>A3. Zoster is caused by reactivation of VZV. Following primary infection or vaccination, VZV remains latent in the sensory dorsal root ganglion cells. The virus begins to replicate at some later time, traveling down the sensory nerve into the skin. Immunosuppression, including use of tumor necrosis factor (TNF) inhibitors, Janus kinase Janus kinase (JAK)-inhibitors, and some cancer therapies, HIV, and age-related deficiency of cell-mediated immunity are some of the most common

remains dormant in the nerve roots and can reactivate later in life, leading to herpes zoster. The reactivation of VZV causes a localized skin rash, usually on one side of the body, with blisters that typically scab over after a few days. The rash is usually accompanied by pain, burning, or tingling sensations in the affected area. The blisters usually occur in a dermatome, which is a strip of skin supplied by a single spinal nerve. The rash usually lasts for two to four weeks and resolves without any specific treatment. However, antiviral medications can be used to shorten the duration and severity of the illness. Herpes zoster is more common in people with weakened immune systems and those over the age of 50. It is also important to note that people who have had chickenpox are at risk of developing herpes zoster later in life. Prodromal symptoms such as fatigue, headache, and an itchy sensation in the area where the rash<br><br>Q4. How should I treat it? What medicine should I use?<br><br>A4. Treatment of herpes zoster typically involves managing symptoms and supporting the immune system. Antiviral medications such as acyclovir, valaciclovir, or famciclovir can be used to reduce the duration and severity of the illness. These medications work by inhibiting the replication of the virus and are most effective when started within 72 hours of the onset of the rash. Pain relief can be provided by over-the-counter medications such as acetaminophen or ibuprofen. Cool compresses can also be used to reduce pain and itching. In addition, efforts should be made to avoid triggers that can worsen symptoms, such as exposure to sunlight or stress. If the rash is severe, or if complications such as postherpetic neuralgia (PHN) occur, prescription medications may be needed. In such cases, it is best to consult a dermatologist or a healthcare provider for proper evaluation and treatment.	causes of zoster.<br><br>Q4. How should I treat it? What medicine should I use?<br><br>A4. Middle-age and elderly patients with herpes zoster are urged to restrict their physical activities or even stay home in bed for a few days. Bed rest may be of paramount importance in the prevention of neuralgia. The nucleoside analogues acyclovir, famciclovir, valacyclovir, and brivudin and the pyrophosphate analog foscarnet show efficacy in treating VZV infections. Because some of the pain during acute zoster (acute zoster neuritis) may have an inflammatory component, corticosteroids have been used during the acute episode.
Evaluation of Physician	
Evaluation Item	Score (1-5, the higher the better)
SkinGPT-4's diagnosis is correct or relevant.	5

SkinGPT-4's description is informative.	4
SkinGPT-4's suggestions are useful.	4
SkinGPT-4 can help doctors with diagnosis.	5
SkinGPT-4 can help patients to understand their disease better.	5
If SkinGPT-4 can be deployed locally, it protects patients' privacy.	5
Willingness to use SkinGPT-4	5

Case Herpes 3

Image ID: Herpes3	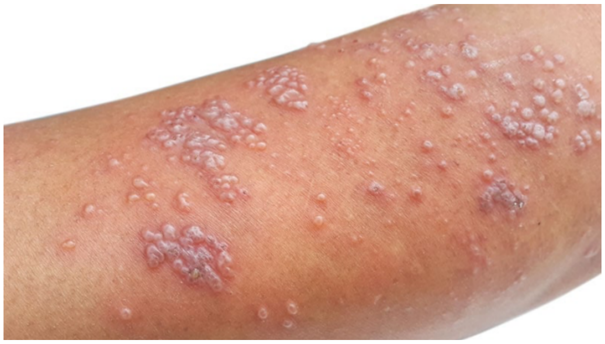
**SkinGPT-4**	**Dermatology Physician**
Q1. Could you describe the skin disease in this image for me?<br><br>A1. This could be a skin disease image of herpes zoster.<br><br>Q2. You should write a paragraph to list more features you observed in the image.<br><br>A2. This could be a herpes zoster image. The features observed in this image include: Papules or vesicles, erythema (inflammation), and induration (hardening of the skin).<br><br>Q3. With previous information, please write a paragraph to explain in detail the cause of this skin disease.<br><br>A3. This could be a herpes zoster image, which is caused by the varicella-zoster virus (VZV). The virus remains dormant in nerve cells after the initial episode of chickenpox and can reactivate later in life, resulting in herpes zoster. The virus causes an immune response leading to the formation of vesicles or papules on one side of the body, often accompanied by symptoms such as pain, itching, and fever. The vesicles eventually crust over and fall off, leaving behind scarring and pigment changes, as seen in this image. The disease can also affect the nerves, leading to abnormal sensations, weakness, or paralysis. Prophylaxis with varicella-zoster immune globulin and vaccination can prevent infection and reduce severity of symptoms.<br><br>Q4. How should I treat it? What medicine should I use?	Q1. Could you describe the skin disease in this image for me?<br><br>A1. This could be a skin disease image of herpes zoster.<br><br>Q2. You should write a paragraph to list more features you observed in the image.<br><br>A2. The most distinctive feature of herpes zoster is the localization and distribution of the rash, which is nearly always unilateral and is generally limited to the area of skin innervated by a single sensory ganglion. Herpes zoster lesions begin as erythematous macules and papules. Vesicles form within 12–24 hours and evolve into pustules by the third day. These dry and crust in 7–10 days. The crusts generally persist for 2–3 weeks. In normal individuals, new lesions continue to appear for 1–4 days (occasionally for as long as 7 days).<br><br>Q3. With previous information, please write a paragraph to explain in detail the cause of this skin disease.<br><br>A3. Zoster is caused by reactivation of VZV. Following primary infection or vaccination, VZV remains latent in the sensory dorsal root ganglion cells. The virus begins to replicate at some later time, traveling down the sensory nerve into the skin. Immunosuppression, including use of tumor necrosis factor (TNF) inhibitors, Janus kinase Janus kinase (JAK)-inhibitors, and some cancer therapies, HIV, and age-related deficiency of cell-mediated immunity are some of the most common causes of zoster.

A4. Treatment of herpes zoster typically involves antiviral medication to shorten the duration and severity of the illness, as well as symptomatic treatment for pain, fever, and itching. Antiviral medications such as acyclovir, valacyclovir, or famciclovir can be prescribed to reduce the viral load and prevent the spread of infection to other people. Pain can be managed with over-the-counter medications such as acetaminophen or ibuprofen, or prescription medications such as gabapentin or lidocaine. Cool compresses and calamine lotion can also help with itching and discomfort. Severe cases or those causing complications may require hospitalization and additional treatment. It is important to follow the advice of a healthcare professional for the appropriate treatment of herpes zoster.	Q4. How should I treat it? What medicine should I use?<br><br>A4. Middle-age and elderly patients with herpes zoster are urged to restrict their physical activities or even stay home in bed for a few days. Bed rest may be of paramount importance in the prevention of neuralgia. The nucleoside analogues acyclovir, famciclovir, valacyclovir, and brivudin and the pyrophosphate analog foscarnet show efficacy in treating VZV infections. Because some of the pain during acute zoster (acute zoster neuritis) may have an inflammatory component, corticosteroids have been used during the acute episode.
Evaluation of Physician	
Evaluation Item	Score (1-5, the higher the better)
SkinGPT-4's diagnosis is correct or relevant.	5
SkinGPT-4's description is informative.	4
SkinGPT-4's suggestions are useful.	4
SkinGPT-4 can help doctors with diagnosis.	5
SkinGPT-4 can help patients to understand their disease better.	4
If SkinGPT-4 can be deployed locally, it protects patients' privacy.	5
Willingness to use SkinGPT-4	5

Case Herpes 4

<table>
<tr><td>Image ID: Herpes4</td><td>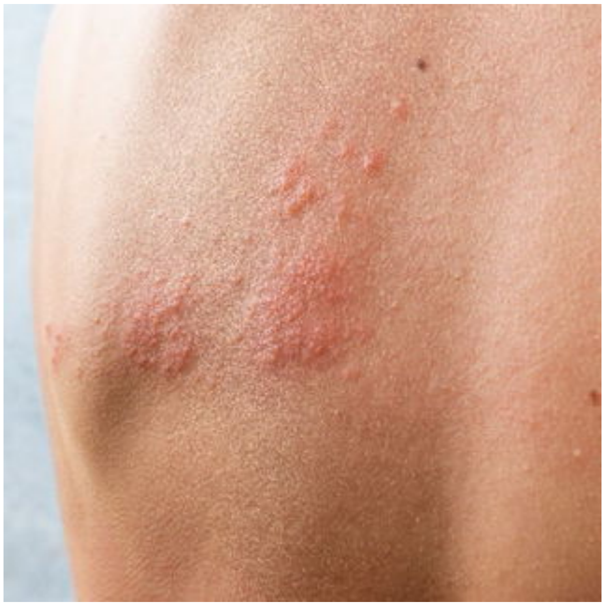</td></tr>
<tr><th>SkinGPT-4</th><th>Dermatology Physician</th></tr>
<tr><td>Q1. Could you describe the skin disease in this image for me?

A1. This could be a skin disease image of herpes simplex.

Q2. You should write a paragraph to list more features you observed in the image.

A2. This image shows a skin disease that affects the moist areas of the skin such as axillary and inguinal regions, but can also involve other parts of the body including the face, neck, and flexural areas. The disease presents as moist, macerated patches with white or grayish center and erythematous periphery. The lesions often have a greasy or shiny appearance and can be itchy. The disease can be painless and can be mistaken for other skin conditions such as eczema or psoriasis. The lesions can progress to become more inflamed and weepy, and can also become secondarily infected with bacteria or fungi, leading to an exudative and suppurative phase.

Q3. With previous information, please write a paragraph to explain in detail the cause of this skin disease.</td><td>Q1. Could you describe the skin disease in this image for me?

A1. This could be a skin disease image of herpes zoster.

Q2. You should write a paragraph to list more features you observed in the image.

A2. The most distinctive feature of herpes zoster is the localization and distribution of the rash, which is nearly always unilateral and is generally limited to the area of skin innervated by a single sensory ganglion. Herpes zoster lesions begin as erythematous macules and papules. Vesicles form within 12–24 hours and evolve into pustules by the third day. These dry and crust in 7–10 days. The crusts generally persist for 2–3 weeks. In normal individuals, new lesions continue to appear for 1–4 days (occasionally for as long as 7 days).

Q3. With previous information, please write a paragraph to explain in detail the cause of this skin disease.

A3. Zoster is caused by reactivation of VZV. Following primary infection or vaccination, VZV remains latent in the sensory dorsal root ganglion</td></tr>
</table>

A3. This skin disease is caused by a deficiency of the enzyme ceramidase, which plays a crucial role in maintaining the balance of lipids in the skin. Without this enzyme, the skin is unable to properly metabolize fatty acids, leading to the accumulation of toxic substances and the formation of waxy white plaques. The deficiency of ceramidase can be caused by mutations in the CDS1 gene, which encodes for the enzyme. This genetic disorder is inherited in an autosomal recessive manner, meaning that individuals must inherit two copies of the mutated gene (one from each parent) to develop the disease. There is currently no cure for this condition, but treatments such as moisturizers and emollients can help manage symptoms and prevent skin infections.<br><br>Q4. How should I treat it? What medicine should I use?<br><br>A4. Treatment for this skin disease typically involves the use of moisturizers and emollients to help manage symptoms and prevent skin infections. Moisturizers help to lock in skin moisture and reduce dryness, while emollients help to soothe and soften the skin. In addition, topical steroids may be used to reduce inflammation and itching.<br>It is important to avoid harsh soaps and detergents, as these can dry out the skin and make symptoms worse. It is also important to keep the skin clean and dry, and to avoid scratching which can lead to skin infections.<br>In cases that are severe or do not improve with topical treatments, medications such as retinoids, vitamin D analogs, and enzyme replacement therapy may be considered.<br>It is important to consult a dermatologist for proper diagnosis and treatment plan.	cells. The virus begins to replicate at some later time, traveling down the sensory nerve into the skin. Immunosuppression, including use of tumor necrosis factor (TNF) inhibitors, Janus kinase Janus kinase (JAK)-inhibitors, and some cancer therapies, HIV, and age-related deficiency of cell-mediated immunity are some of the most common causes of zoster.<br><br>Q4. How should I treat it? What medicine should I use?<br><br>A4. Middle-age and elderly patients with herpes zoster are urged to restrict their physical activities or even stay home in bed for a few days. Bed rest may be of paramount importance in the prevention of neuralgia. The nucleoside analogues acyclovir, famciclovir, valacyclovir, and brivudin and the pyrophosphate analog foscarnet show efficacy in treating VZV infections. Because some of the pain during acute zoster (acute zoster neuritis) may have an inflammatory component, corticosteroids have been used during the acute episode.
Evaluation of Physician	
Evaluation Item	Score (1-5, the higher the better)
SkinGPT-4’s diagnosis is correct or relevant.	5
SkinGPT-4’s description is informative.	1
SkinGPT-4’s suggestions are useful.	1

SkinGPT-4 can help doctors with diagnosis.	5
SkinGPT-4 can help patients to understand their disease better.	1
If SkinGPT-4 can be deployed locally, it protects patients' privacy.	1
Willingness to use SkinGPT-4	1

Case Herpes 5

Image ID: Herpes5	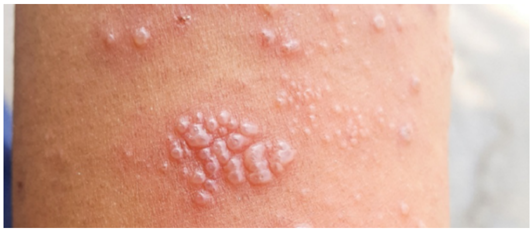
**SkinGPT-4**	**Dermatology Physician**
Q1. Could you describe the skin disease in this image for me?<br><br>A1. This could be a skin disease image of herpes zoster.<br><br>Q2. You should write a paragraph to list more features you observed in the image.<br><br>A2. This image shows a case of herpes zoster, which is also known as shingles. The rash consists of small, fluid filled blisters that typically form a band or patch on one side of the body. The blisters typically scab over and then fall off, leaving behind erythema (inflammation and redness) and exudate (pus or fluid). The skin around the blisters may also be edematous (swollen) and tender. The disease is caused by reactivation of the dormant varicella zoster virus, which causes chickenpox, and typically affects people who have previously had chickenpox. The disease is more common in adults and can be treated with antiviral medications, but there is no cure.<br><br>Q3. With previous information, please write a paragraph to explain in detail the cause of this skin disease.<br><br>A3. This image shows a case of herpes zoster, which is caused by reactivation of the dormant varicella zoster virus (VZV). VZV is a double stranded DNA virus that belongs to the family Herpesviridae. It causes chickenpox, a highly infectious and contagious disease that typically affects children and adolescents. After an attack of chickenpox, the virus remains dormant in nerve cells and can reactivate later in life, leading to herpes zoster. Herpes zoster is also known as shingles and typically affects people who have	Q1. Could you describe the skin disease in this image for me?<br><br>A1. This could be a skin disease image of herpes zoster.<br><br>Q2. You should write a paragraph to list more features you observed in the image.<br><br>A2. The most distinctive feature of herpes zoster is the localization and distribution of the rash, which is nearly always unilateral and is generally limited to the area of skin innervated by a single sensory ganglion. Herpes zoster lesions begin as erythematous macules and papules. Vesicles form within 12–24 hours and evolve into pustules by the third day. These dry and crust in 7–10 days. The crusts generally persist for 2–3 weeks. In normal individuals, new lesions continue to appear for 1–4 days (occasionally for as long as 7 days).<br><br>Q3. With previous information, please write a paragraph to explain in detail the cause of this skin disease.<br><br>A3. Zoster is caused by reactivation of VZV. Following primary infection or vaccination, VZV remains latent in the sensory dorsal root ganglion cells. The virus begins to replicate at some later time, traveling down the sensory nerve into the skin. Immunosuppression, including use of tumor necrosis factor (TNF) inhibitors, Janus kinase Janus kinase (JAK)-inhibitors, and some cancer therapies, HIV, and age-related deficiency of cell-mediated immunity are some of the most common causes of zoster.<br><br>Q4. How should I treat it? What medicine should I use?

previously had chickenpox. The reactivation of VZV is more common in adults and people with weakened immune systems. The disease is characterized by a rash that typically forms a band or patch on one side of the body and is accompanied by symptoms such as pain, burning, and itching. The rash consists of small, fluid filled blisters that typically scab over and then fall off, leaving behind erythema (inflammation and redness) and exudate (pus or fluid). The disease is contagious and can be treated with antiviral medications, but there is no cure.<br><br>Q4. How should I treat it? What medicine should I use?<br><br>A4. Herpes zoster, also known as shingles, is typically treated with antiviral medications, which can help shorten the duration and severity of the disease. The most commonly used antiviral medications for herpes zoster are acyclovir, valaciclovir, and famciclovir. These medications work by inhibiting the replication of the varicella-zoster virus (VZV), which causes herpes zoster. They can be taken orally or applied topically, and are most effective when started within 72 hours of the onset of the rash. Pain relief can also be provided by using over the counter pain medications such as acetaminophen or ibuprofen. It is important to consult a healthcare professional for proper diagnosis and treatment.	A4. Middle-age and elderly patients with herpes zoster are urged to restrict their physical activities or even stay home in bed for a few days. Bed rest may be of paramount importance in the prevention of neuralgia. The nucleoside analogues acyclovir, famciclovir, valacyclovir, and brivudin and the pyrophosphate analog foscarnet show efficacy in treating VZV infections. Because some of the pain during acute zoster (acute zoster neuritis) may have an inflammatory component, corticosteroids have been used during the acute episode.
Evaluation of Physician	
Evaluation Item	Score (1-5, the higher the better)
SkinGPT-4's diagnosis is correct or relevant.	5
SkinGPT-4's description is informative.	5
SkinGPT-4's suggestions are useful.	5
SkinGPT-4 can help doctors with diagnosis.	5
SkinGPT-4 can help patients to understand their disease better.	5
If SkinGPT-4 can be deployed locally, it protects patients' privacy.	5

Willingness to use SkinGPT-4	5

Case Herpes 6

Image ID: Herpes6	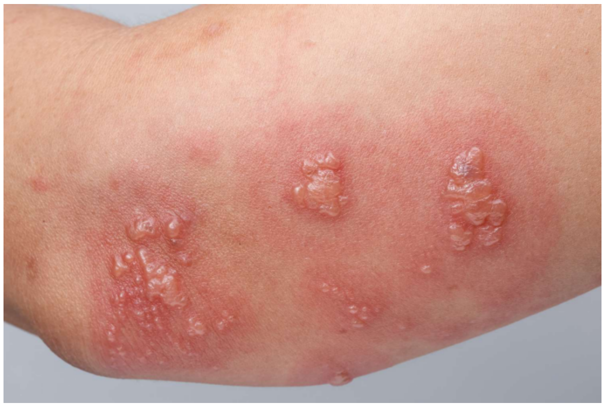
**SkinGPT-4**	**Dermatology Physician**
Q1. Could you describe the skin disease in this image for me?<br><br>A1. This could be a skin disease image of herpes zoster.<br><br>Q2. You should write a paragraph to list more features you observed in the image.<br><br>A2. This could be a herpes zoster image. The features observed in this image include erythema, blistering, and a pruritic rash. The rash is usually in a dermatomal distribution and can involve the face, ears, and neck. The blisters are filled with clear fluid and eventually rupture, leaving erythematous plaques that eventually turn into scabs. The infection is caused by the herpes virus and is more common in immunocompromised individuals.<br><br>Q3. With previous information, please write a paragraph to explain in detail the cause of this skin disease.<br><br>A3. Herpes zoster, also known as shingles, is caused by the reactivation of the varicella-zoster virus, which also causes chickenpox. After an initial infection, the virus remains dormant in the nerve cells and can reactivate later in life, leading to herpes zoster. The exact trigger for the reactivation is not known, but it can be associated with stress, immunosuppression, and advancing age. The infection presents with a characteristic rash involving a single dermatome on one side of the body, which typically involves the face, ears,	Q1. Could you describe the skin disease in this image for me?<br><br>A1. This could be a skin disease image of herpes zoster.<br><br>Q2. You should write a paragraph to list more features you observed in the image.<br><br>A2. The most distinctive feature of herpes zoster is the localization and distribution of the rash, which is nearly always unilateral and is generally limited to the area of skin innervated by a single sensory ganglion. Herpes zoster lesions begin as erythematous macules and papules. Vesicles form within 12–24 hours and evolve into pustules by the third day. These dry and crust in 7–10 days. The crusts generally persist for 2–3 weeks. In normal individuals, new lesions continue to appear for 1–4 days (occasionally for as long as 7 days).<br><br>Q3. With previous information, please write a paragraph to explain in detail the cause of this skin disease.<br><br>A3. Zoster is caused by reactivation of VZV. Following primary infection or vaccination, VZV remains latent in the sensory dorsal root ganglion cells. The virus begins to replicate at some later time, traveling down the sensory nerve into the skin. Immunosuppression, including use of tumor necrosis factor (TNF) inhibitors, Janus kinase Janus kinase (JAK)-inhibitors, and some cancer therapies, HIV, and age-related deficiency of cell-mediated immunity are some of the most common

and neck. The rash starts as a erythema and then progresses to small blisters that eventually rupture, leaving behind a macular rash. The infection is contagious and can be transmitted to immunocompromised individuals, such as those with AIDS, through direct contact with the blisters or rash. Treatment includes antiviral medications, which can reduce the severity and duration of the disease, as well as symptomatic treatment with over the counter medications.<br><br>Q4. How should I treat it? What medicine should I use?<br><br>A4. Treatment of herpes zoster typically involves the use of antiviral medications, such as acyclovir, valacyclovir, or famciclovir. These medications work by inhibiting viral replication and are most effective when started within 72 hours of the onset of the rash. They can shorten the duration of the illness and reduce the severity of the symptoms. Pain relief can be managed with over the counter medications such as acetaminophen or ibuprofen. Cool compresses can also help reduce the pain and inflammation. In addition, antibiotics are not recommended for herpes zoster as they are not effective against a viral infection.	causes of zoster.<br><br>Q4. How should I treat it? What medicine should I use?<br><br>A4. Middle-age and elderly patients with herpes zoster are urged to restrict their physical activities or even stay home in bed for a few days. Bed rest may be of paramount importance in the prevention of neuralgia. The nucleoside analogues acyclovir, famciclovir, valacyclovir, and brivudin and the pyrophosphate analog foscarnet show efficacy in treating VZV infections. Because some of the pain during acute zoster (acute zoster neuritis) may have an inflammatory component, corticosteroids have been used during the acute episode.

Evaluation of Physician	
Evaluation Item	Score (1-5, the higher the better)
SkinGPT-4's diagnosis is correct or relevant.	5
SkinGPT-4's description is informative.	4
SkinGPT-4's suggestions are useful.	5
SkinGPT-4 can help doctors with diagnosis.	5
SkinGPT-4 can help patients to understand their disease better.	5
If SkinGPT-4 can be deployed locally, it protects patients' privacy.	5
Willingness to use SkinGPT-4	5

Case Herpes 7

Image ID: Herpes7	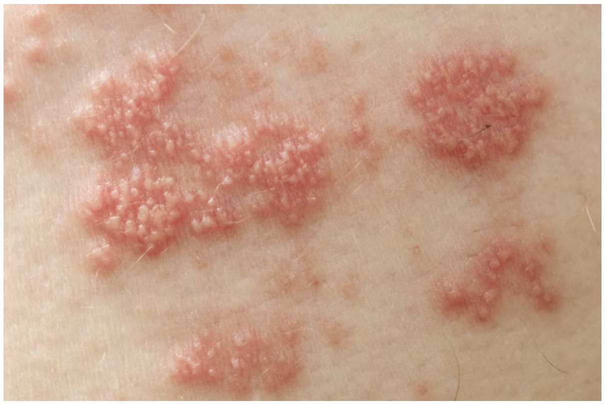
SkinGPT-4	Dermatology Physician
Q1. Could you describe the skin disease in this image for me?<br><br>A1. This could be a skin disease image of herpes simplex.<br><br>Q2. You should write a paragraph to list more features you observed in the image.<br><br>A2. This image is a skin disease image of herpes simplex. The herpes simplex virus causes this condition, which presents as one or more small, fluid-filled blisters on an erythematous base. The blisters typically occur in clusters and can be painful. The skin around the blisters may be erythematous, and there may be some oozing. The lesions typically heal without scarring, but the virus remains dormant in the nerve cells, and recurrences are common.<br><br>Q3. With previous information, please write a paragraph to explain in detail the cause of this skin disease.<br><br>A3. Herpes simplex is caused by the herpes simplex virus (HSV), which is a highly contagious viral infection. The virus is spread through direct contact with the lesions or secretions of an infected person. The virus can be transmitted through skin-to-skin contact, sexual contact, or contact with objects that have come into contact with the virus, such as towels, razors, or toothbrushes. Once the virus enters the body, it replicates and causes the symptoms of herpes simplex. The virus infects the cells of the skin or	Q1. Could you describe the skin disease in this image for me?<br><br>A1. This could be a skin disease image of herpes zoster.<br><br>Q2. You should write a paragraph to list more features you observed in the image.<br><br>A2. The most distinctive feature of herpes zoster is the localization and distribution of the rash, which is nearly always unilateral and is generally limited to the area of skin innervated by a single sensory ganglion. Herpes zoster lesions begin as erythematous macules and papules. Vesicles form within 12–24 hours and evolve into pustules by the third day. These dry and crust in 7–10 days. The crusts generally persist for 2–3 weeks. In normal individuals, new lesions continue to appear for 1–4 days (occasionally for as long as 7 days).<br><br>Q3. With previous information, please write a paragraph to explain in detail the cause of this skin disease.<br><br>A3. Zoster is caused by reactivation of VZV. Following primary infection or vaccination, VZV remains latent in the sensory dorsal root ganglion cells. The virus begins to replicate at some later time, traveling down the sensory nerve into the skin. Immunosuppression, including use of tumor necrosis factor (TNF) inhibitors, Janus kinase Janus kinase (JAK)-inhibitors, and some cancer therapies, HIV, and age-related deficiency of cell-mediated immunity are some of the most common

mucous membranes and can remain dormant in the nerve cells, leading to recurrent episodes of the disease. Herpes simplex can be treated with antiviral medications, but there is no cure for the disease. Prevention measures include good hygiene, avoiding contact with infected individuals, and using barrier protection during sexual contact.<br><br>Q4. How should I treat it? What medicine should I use?<br><br>A4. Treatment of herpes simplex typically involves antiviral medications to reduce the severity and duration of symptoms, as well as to prevent recurrences. The most commonly used antiviral medications for herpes simplex are acyclovir, valacyclovir, and famciclovir. These medications work by inhibiting the replication of the herpes simplex virus and can reduce the severity and duration of symptoms by one to two days.<br>It is important to start treatment as soon as possible after the onset of symptoms to minimize the severity and duration of the disease. Treatment with antiviral medications can also help prevent the spread of the virus to other people.<br><br>It is also important to note that antiviral medications do not cure herpes simplex, and the virus can remain in the body and cause recurrent episodes of the disease. To prevent recurrences, it is recommended to take antiviral medications at the first sign of symptoms, and to avoid triggers such as stress, fatigue, and sun exposure.	causes of zoster.<br><br>Q4. How should I treat it? What medicine should I use?<br><br>A4. Middle-age and elderly patients with herpes zoster are urged to restrict their physical activities or even stay home in bed for a few days. Bed rest may be of paramount importance in the prevention of neuralgia. The nucleoside analogues acyclovir, famciclovir, valacyclovir, and brivudin and the pyrophosphate analog foscarnet show efficacy in treating VZV infections. Because some of the pain during acute zoster (acute zoster neuritis) may have an inflammatory component, corticosteroids have been used during the acute episode.
Evaluation of Physician	
Evaluation Item	Score (1-5, the higher the better)
SkinGPT-4's diagnosis is correct or relevant.	5
SkinGPT-4's description is informative.	5
SkinGPT-4's suggestions are useful.	5
SkinGPT-4 can help doctors with diagnosis.	5
SkinGPT-4 can help patients to understand their disease better.	4

If SkinGPT-4 can be deployed locally, it protects patients' privacy.	5
Willingness to use SkinGPT-4	4

Case Herpes 8

Image ID: Herpes8	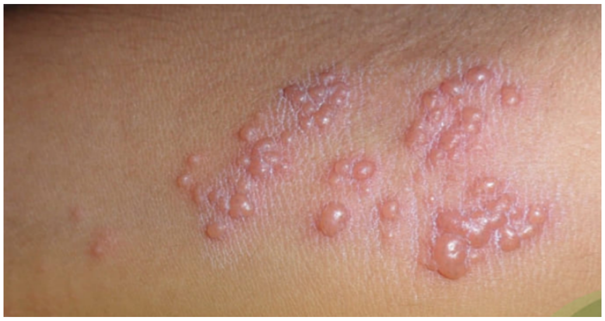
**SkinGPT-4**	**Dermatology Physician**
Q1. Could you describe the skin disease in this image for me?<br><br>A1. This could be a skin disease image of herpes zoster.<br><br>Q2. You should write a paragraph to list more features you observed in the image.<br><br>A2. This image shows a skin lesion on the left side of the face, which is erythematous and indurated with central vesicle. There is a hypopigmented area adjacent to the lesion, which suggests postinflammatory hyperpigmentation. The lesion is surrounded by a circular boundary, which suggests a nevus simplex. The lesion is located on the left side of the face, which is consistent with herpes zoster (shingles).<br><br>Q3. With previous information, please write a paragraph to explain in detail the cause of this skin disease.<br><br>A3. The cause of this skin disease is herpes zoster, also known as shingles. Herpes zoster is a viral infection caused by the varicella-zoster virus, which also causes chickenpox. After a person recovers from chickenpox, the virus remains in their body and can reactivate later in life, causing herpes zoster. The reactivation of herpes zoster typically occurs in people who have weakened immune systems, such as those with HIV/AIDS, or those taking immunosuppressive drugs. Herpes zoster presents as a rash on one side of the body, usually on the trunk, and is often accompanied by symptoms such as pain, burning, and itching. The rash begins as small red bumps, which then blister	Q1. Could you describe the skin disease in this image for me?<br><br>A1. This could be a skin disease image of herpes zoster.<br><br>Q2. You should write a paragraph to list more features you observed in the image.<br><br>A2. The most distinctive feature of herpes zoster is the localization and distribution of the rash, which is nearly always unilateral and is generally limited to the area of skin innervated by a single sensory ganglion. Herpes zoster lesions begin as erythematous macules and papules. Vesicles form within 12–24 hours and evolve into pustules by the third day. These dry and crust in 7–10 days. The crusts generally persist for 2–3 weeks. In normal individuals, new lesions continue to appear for 1–4 days (occasionally for as long as 7 days).<br><br>Q3. With previous information, please write a paragraph to explain in detail the cause of this skin disease.<br><br>A3. Zoster is caused by reactivation of VZV. Following primary infection or vaccination, VZV remains latent in the sensory dorsal root ganglion cells. The virus begins to replicate at some later time, traveling down the sensory nerve into the skin. Immunosuppression, including use of tumor necrosis factor (TNF) inhibitors, Janus kinase Janus kinase (JAK)-inhibitors, and some cancer therapies, HIV, and age-related deficiency of cell-mediated immunity are some of the most common causes of zoster.

and crust over. The lesions usually heal within two to four weeks, but the virus can remain in the nerve roots and cause pain, known as postherpetic neuralgia, which can last for months or even years after the rash has healed.<br><br>Q4. How should I treat it? What medicine should I use?<br><br>A4. Herpes zoster is treated with antiviral medications, such as acyclovir, valaciclovir, or famciclovir, which can reduce the duration and severity of the illness if started within 72 hours of the onset of the rash. Pain relief can be managed with over-the-counter pain medications, such as acetaminophen or ibuprofen. Cool compresses or baths can help reduce itching and inflammation. If the herpes zoster is severe or if there are complications, such as bacterial superinfection, hospitalization and intravenous antiviral therapy may be necessary. It is important to note that antiviral medications do not cure herpes zoster, but they can help to shorten the duration and severity of the illness.	Q4. How should I treat it? What medicine should I use?<br><br>A4. Middle-age and elderly patients with herpes zoster are urged to restrict their physical activities or even stay home in bed for a few days. Bed rest may be of paramount importance in the prevention of neuralgia. The nucleoside analogues acyclovir, famciclovir, valacyclovir, and brivudin and the pyrophosphate analog foscarnet show efficacy in treating VZV infections. Because some of the pain during acute zoster (acute zoster neuritis) may have an inflammatory component, corticosteroids have been used during the acute episode.
Evaluation of Physician	
Evaluation Item	Score (1-5, the higher the better)
SkinGPT-4’s diagnosis is correct or relevant.	5
SkinGPT-4’s description is informative.	3
SkinGPT-4’s suggestions are useful.	5
SkinGPT-4 can help doctors with diagnosis.	5
SkinGPT-4 can help patients to understand their disease better.	4
If SkinGPT-4 can be deployed locally, it protects patients’ privacy.	5
Willingness to use SkinGPT-4	5

Case Herpes 9

<table>
<tr><td>Image ID: Herpes9</td><td>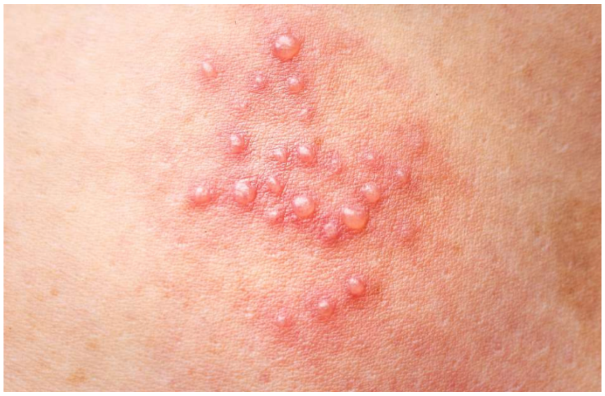</td></tr>
<tr><th>SkinGPT-4</th><th>Dermatology Physician</th></tr>
<tr><td>Q1. Could you describe the skin disease in this image for me?

A1. This could be a skin disease image of herpes simplex.

Q2. You should write a paragraph to list more features you observed in the image.

A2. The image shows a generalized herpes simplex virus infection. The lesions are present on the lower lip, which is a common site for herpes simplex virus to erupt. The lesions are small, white or grayish-white, with a depressed center, and they are surrounded by an inflamed halo. The lesions are sometimes referred to as cold sores or fever blisters. The skin around the lesions appears normal. There is no pustulation, erythema, or edema. The lesions are painful and can be accompanied by symptoms such as itching, tingling, or burning. The disease can be confirmed by PCR or viral culture.

Q3. With previous information, please write a paragraph to explain in detail the cause of this skin disease.

A3. The cause of this skin disease is herpes simplex virus (HSV) infection. HSV is a highly contagious viral infection that can affect any part of the body, including the skin, mucous membranes, and eyes. HSV type 1 is primarily responsible for herpetic whitlow, while HSV type 2 is responsible for genital herpes. HSV infects individuals of all ages and can be transmitted</td><td>Q1. Could you describe the skin disease in this image for me?

A1. This could be a skin disease image of herpes zoster.

Q2. You should write a paragraph to list more features you observed in the image.

A2. The most distinctive feature of herpes zoster is the localization and distribution of the rash, which is nearly always unilateral and is generally limited to the area of skin innervated by a single sensory ganglion. Herpes zoster lesions begin as erythematous macules and papules. Vesicles form within 12–24 hours and evolve into pustules by the third day. These dry and crust in 7–10 days. The crusts generally persist for 2–3 weeks. In normal individuals, new lesions continue to appear for 1–4 days (occasionally for as long as 7 days).

Q3. With previous information, please write a paragraph to explain in detail the cause of this skin disease.

A3. Zoster is caused by reactivation of VZV. Following primary infection or vaccination, VZV remains latent in the sensory dorsal root ganglion cells. The virus begins to replicate at some later time, traveling down the sensory nerve into the skin. Immunosuppression, including use of tumor necrosis factor (TNF) inhibitors, Janus kinase Janus kinase (JAK)-inhibitors, and some cancer therapies, HIV, and age-related deficiency of cell-mediated immunity are some of the most common</td></tr>
</table>

through direct contact with a lesion or secretions from an infected person. The infection occurs through a break in the skin or mucous membranes, such as a cut, scratch, or abrasion. The virus replicates in the epithelial cells and produces small, white or grayish-white lesions on the skin. The lesions are surrounded by an inflamed halo and can be accompanied by symptoms such as itching, tingling, or burning. The virus remains dormant in the nerve cells and can reactivate later in life, causing recurrent episodes of herpes simplex. The disease can be confirmed by PCR or viral culture. Prevention measures include good hygiene, avoiding sharing personal items, and avoiding contact with people who have herpes simplex lesions.<br><br>Q4. How should I treat it? What medicine should I use?<br><br>A4. The treatment of herpes simplex depends on the severity and location of the infection. For simple herpetic whitlow, the following steps can be taken:<br>* Avoiding trauma to the affected area<br>* Keeping the area clean and dry<br>* Applying ice to the affected area to reduce pain and swelling<br>* Taking over-the-counter pain medication, such as acetaminophen or ibuprofen<br>* Antiviral medication, such as acyclovir, valacyclovir, or famciclovir, can be prescribed to reduce viral replication and shorten the duration of symptoms.<br>It is important to seek medical attention if the infection does not improve with home treatment, if there are systemic symptoms, or if the infection is severe. A healthcare professional can prescribe antiviral medication, and if necessary, refer the patient to a dermatologist or other specialist.	causes of zoster.<br><br>Q4. How should I treat it? What medicine should I use?<br><br>A4. Middle-age and elderly patients with herpes zoster are urged to restrict their physical activities or even stay home in bed for a few days. Bed rest may be of paramount importance in the prevention of neuralgia. The nucleoside analogues acyclovir, famciclovir, valacyclovir, and brivudin and the pyrophosphate analog foscarnet show efficacy in treating VZV infections. Because some of the pain during acute zoster (acute zoster neuritis) may have an inflammatory component, corticosteroids have been used during the acute episode.
Evaluation of Physician	
Evaluation Item	Score (1-5, the higher the better)
SkinGPT-4’s diagnosis is correct or relevant.	5
SkinGPT-4’s description is informative.	5

SkinGPT-4's suggestions are useful.	3
SkinGPT-4 can help doctors with diagnosis.	5
SkinGPT-4 can help patients to understand their disease better.	4
If SkinGPT-4 can be deployed locally, it protects patients' privacy.	5
Willingness to use SkinGPT-4	5

Case Herpes 10

Image ID: Herpes10	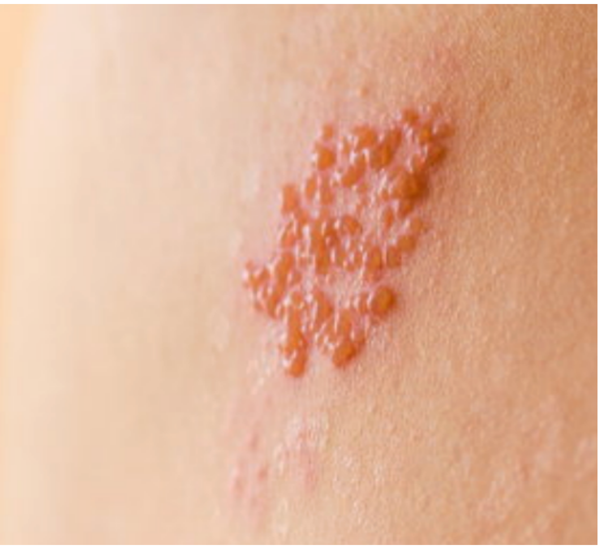
SkinGPT-4	Dermatology Physician
Q1. Could you describe the skin disease in this image for me?<br><br>A1. This could be a skin disease image of herpes zoster (shingles).<br><br>Q2. You should write a paragraph to list more features you observed in the image.<br><br>A2. In this image, the observer can see a small, raised, flesh colored bump with a visible central punctum. The borders are smooth and the bump is not inflamed. The observer can also see nearby normal appearing skin. This image could represent a skin disease such as a seborrheic keratosis.<br><br>Q3. With previous information, please write a paragraph to explain in detail the cause of this skin disease.<br><br>A3. This skin disease is likely caused by the herpes zoster virus, also known as shingles. The virus can reactivate in people who have previously had chickenpox, causing a rash with painful blisters on one side of the body. The virus typically affects people with weakened immune systems, such as those with HIV or cancer. It can also be caused by stress, fatigue, or skin trauma. The virus multiplies in nerve cells and produces an	Q1. Could you describe the skin disease in this image for me?<br><br>A1. This could be a skin disease image of herpes zoster.<br><br>Q2. You should write a paragraph to list more features you observed in the image.<br><br>A2. The most distinctive feature of herpes zoster is the localization and distribution of the rash, which is nearly always unilateral and is generally limited to the area of skin innervated by a single sensory ganglion. Herpes zoster lesions begin as erythematous macules and papules. Vesicles form within 12–24 hours and evolve into pustules by the third day. These dry and crust in 7–10 days. The crusts generally persist for 2–3 weeks. In normal individuals, new lesions continue to appear for 1–4 days (occasionally for as long as 7 days).<br><br>Q3. With previous information, please write a paragraph to explain in detail the cause of this skin disease.<br><br>A3. Zoster is caused by reactivation of VZV. Following primary infection or vaccination, VZV remains latent in the sensory dorsal root ganglion cells. The virus begins to replicate at some later time, traveling down the sensory nerve into the

inflammatory response, leading to the characteristic rash and blisters. After the blisters scab over, they fall off and the skin heals, but scarring may occur. Prevention includes maintaining a healthy immune system, practicing good hygiene, and avoiding contact with people with active herpes infections. Treatment includes antiviral medications and pain relief, and may prevent complications such as postherpetic neuralgia.<br><br>Q4. How should I treat it? What medicine should I use?<br><br>A4. Treatment for herpes zoster, also known as shingles, typically includes antiviral medications to reduce the duration and severity of the illness, as well as pain relief. Oral antiviral medications, such as acyclovir, valaciclovir, or famciclovir, are usually prescribed to help fight the virus and reduce symptoms. Topical medications, such as lidocaine, can also be used to relieve pain. Pain relief can be provided by over-the-counter medications such as acetaminophen or ibuprofen, or by prescription medications such as opioids. In some cases, a healthcare provider may also prescribe postherpetic neuralgia treatment to prevent or manage pain that can occur after the rash has healed. It's important to follow the specific treatment plan and instructions provided by a healthcare provider.	skin. Immunosuppression, including use of tumor necrosis factor (TNF) inhibitors, Janus kinase Janus kinase (JAK)-inhibitors, and some cancer therapies, HIV, and age-related deficiency of cell-mediated immunity are some of the most common causes of zoster.<br><br>Q4. How should I treat it? What medicine should I use?<br><br>A4. Middle-age and elderly patients with herpes zoster are urged to restrict their physical activities or even stay home in bed for a few days. Bed rest may be of paramount importance in the prevention of neuralgia. The nucleoside analogues acyclovir, famciclovir, valacyclovir, and brivudin and the pyrophosphate analog foscarnet show efficacy in treating VZV infections. Because some of the pain during acute zoster (acute zoster neuritis) may have an inflammatory component, corticosteroids have been used during the acute episode.
Evaluation of Physician	
Evaluation Item	Score (1-5, the higher the better)
SkinGPT-4's diagnosis is correct or relevant.	5
SkinGPT-4's description is informative.	1
SkinGPT-4's suggestions are useful.	5
SkinGPT-4 can help doctors with diagnosis.	5
SkinGPT-4 can help patients to understand their disease better.	3
If SkinGPT-4 can be deployed locally, it protects patients' privacy.	5

Willingness to use SkinGPT-4	4
