# Supplementary material for: Pre-trained multimodal large language model enhances dermatological diagnosis using SkinGPT-4
Source: Nat Commun. 2024 Jul 5;15:5649. doi: 10.1038/s41467-024-50043-3 (PMC11226626; doi:10.1038/s41467-024-50043-3)
Supplement: Supplementary file 3 — Description Of Additional Supplementary File [file 41467_2024_50043_MOESM3_ESM.pdf]

Description of Additional supplementary file

Supplementary Movie 1. Running Demo of SkinGPT-4
