# Supplementary material for: Pre-trained multimodal large language model enhances dermatological diagnosis using SkinGPT-4
Source: Nat Commun. 2024 Jul 5;15:5649. doi: 10.1038/s41467-024-50043-3 (PMC11226626; doi:10.1038/s41467-024-50043-3)
Supplement: Supplementary file 5 — Reporting summary [file 41467_2024_50043_MOESM5_ESM.pdf]

nature portfolio

Corresponding author(s): Xin Gao

Last updated by author(s): Feb 28, 2024

Reporting Summary

Nature Portfolio wishes to improve the reproducibility of the work that we publish. This form provides structure for consistency and transparency in reporting. For further information on Nature Portfolio policies, see our Editorial Policies and the Editorial Policy Checklist.

Statistics

For all statistical analyses, confirm that the following items are present in the figure legend, table legend, main text, or Methods section.

n/a	Confirmed	
☐	☒	The exact sample size (*n*) for each experimental group/condition, given as a discrete number and unit of measurement
☐	☒	A statement on whether measurements were taken from distinct samples or whether the same sample was measured repeatedly
☐	☒	The statistical test(s) used AND whether they are one- or two-sided<br>*Only common tests should be described solely by name; describe more complex techniques in the Methods section.*
☒	☐	A description of all covariates tested
☒	☐	A description of any assumptions or corrections, such as tests of normality and adjustment for multiple comparisons
☐	☒	A full description of the statistical parameters including central tendency (e.g. means) or other basic estimates (e.g. regression coefficient) AND variation (e.g. standard deviation) or associated estimates of uncertainty (e.g. confidence intervals)
☐	☒	For null hypothesis testing, the test statistic (e.g. *F*, *t*, *r*) with confidence intervals, effect sizes, degrees of freedom and *P* value noted<br>*Give P values as exact values whenever suitable.*
☒	☐	For Bayesian analysis, information on the choice of priors and Markov chain Monte Carlo settings
☒	☐	For hierarchical and complex designs, identification of the appropriate level for tests and full reporting of outcomes
☒	☐	Estimates of effect sizes (e.g. Cohen's *d*, Pearson's *r*), indicating how they were calculated

Our web collection on statistics for biologists contains articles on many of the points above.

Software and code

Policy information about availability of computer code

Data collection	No software was used for data collection.
Data analysis	The software we used were the following which were all available in github: Python (v3.7), Pytorch (v1.9.1), CUDA (v11.4). The code proposed by SkinGPT-4 is publicly available at https://github.com/JoshuaChou2018/SkinGPT-4.

For manuscripts utilizing custom algorithms or software that are central to the research but not yet described in published literature, software must be made available to editors and reviewers. We strongly encourage code deposition in a community repository (e.g. GitHub). See the Nature Portfolio guidelines for submitting code & software for further information.

Data

Policy information about availability of data

All manuscripts must include a data availability statement. This statement should provide the following information, where applicable:

- Accession codes, unique identifiers, or web links for publicly available datasets
- A description of any restrictions on data availability
- For clinical datasets or third party data, please ensure that the statement adheres to our policy

The data that support the findings of this study are divided into two groups: shared data and restricted data. Shared data include the SKINCON dataset and the Dermnet dataset. The SKINCON dataset can be accessed at https://skincon-dataset.github.io/. The Dermnet dataset can be accessed at https://www.kaggle.com/

datasets/shubhamgoel27/dermnet. The restricted in-house skin disease images used in this study but not displayed are not publicly available due to restrictions in the data-sharing agreement.

Research involving human participants, their data, or biological material

Policy information about studies with human participants or human data. See also policy information about sex, gender (identity/presentation), and sexual orientation and race, ethnicity and racism.

Reporting on sex and gender	Sex and gender were not considered in the study design.
Reporting on race, ethnicity, or other socially relevant groupings	Race, ethnicity and socially relevant groupings were not considered in the study design.
Population characteristics	The anonymization of human participants was ensured. Sensitive information, including patient gender, age, name, and nationality, was completely removed.
Recruitment	Recruitment is not relevant to our study since we used pre-collected data.
Ethics oversight	The ethics vote for this study was held by the Beijing Anzhen Hospital Ethics Committee, Beijing AnZhen Hospital, affiliated with Capital Medical University in Beijing, China, with ethical approval obtained under referencing ID 2024002X. The ethical approval from the Institutional Biosafety and Bioethics Committee (IBEC), King Abdullah University of Science and Technology was obtained under referencing ID 23IBEC100.

Note that full information on the approval of the study protocol must also be provided in the manuscript.

Field-specific reporting

Please select the one below that is the best fit for your research. If you are not sure, read the appropriate sections before making your selection.

☒ Life sciences ☐ Behavioural & social sciences ☐ Ecological, evolutionary & environmental sciences

For a reference copy of the document with all sections, see nature.com/documents/nr-reporting-summary-flat.pdf

Life sciences study design

All studies must disclose on these points even when the disclosure is negative.

Sample size	We have collected an extensive collection of skin disease images (comprising 52,929 publicly available and proprietary images) along with clinical concepts and doctors' notes. The number of datasets is determined by the amount of data we actually have. This volume of data exceeds what is currently used in most related research fields, and according to accepted knowledge, this amount is sufficient for our study.
Data exclusions	No experimental data was generated in our study and no data were excluded from analysis.
Replication	All experiments were replicated at least three times and all attempts were successful.
Randomization	Randomization of samples is not applicable in our study since we did not collect any experimental data.
Blinding	Blinding is not relevant to our study since we report a software development as main finding.

Reporting for specific materials, systems and methods

We require information from authors about some types of materials, experimental systems and methods used in many studies. Here, indicate whether each material, system or method listed is relevant to your study. If you are not sure if a list item applies to your research, read the appropriate section before selecting a response.

Materials & experimental systems

n/a	Involved in the study	
☒	☐	Antibodies
☒	☐	Eukaryotic cell lines
☒	☐	Palaeontology and archaeology
☒	☐	Animals and other organisms
☒	☐	Clinical data
☒	☐	Dual use research of concern
☒	☐	Plants

Methods

n/a	Involved in the study	
☒	☐	ChIP-seq
☒	☐	Flow cytometry
☒	☐	MRI-based neuroimaging

Plants

Seed stocks

Report on the source of all seed stocks or other plant material used. If applicable, state the seed stock centre and catalogue number. If plant specimens were collected from the field, describe the collection location, date and sampling procedures.

Novel plant genotypes

Describe the methods by which all novel plant genotypes were produced. This includes those generated by transgenic approaches, gene editing, chemical/radiation-based mutagenesis and hybridization. For transgenic lines, describe the transformation method, the number of independent lines analyzed and the generation upon which experiments were performed. For gene-edited lines, describe the editor used, the endogenous sequence targeted for editing, the targeting guide RNA sequence (if applicable) and how the editor was applied.

Authentication

Describe any authentication procedures for each seed stock used or novel genotype generated. Describe any experiments used to assess the effect of a mutation and, where applicable, how potential secondary effects (e.g. second site T-DNA insertions, mosiacism, off-target gene editing) were examined.
